# Quinone methide dimers lacking labile hydrogen atoms are surprisingly excellent radical-trapping antioxidants†
†Electronic supplementary information (ESI) available: Additional kinetic and thermodynamic data and analyses, synthesis and characterization data, computed optimized geometries, energies, and Cartesian coordinates. See DOI: 10.1039/d0sc02020f


**DOI:** 10.1039/d0sc02020f

**Published:** 2020-05-06

**Authors:** Mark A. R. Raycroft, Jean-Philippe R. Chauvin, Matthew S. Galliher, Kevin J. Romero, Corey R. J. Stephenson, Derek A. Pratt

**Affiliations:** a Department of Chemistry and Biomolecular Sciences , University of Ottawa , Ottawa , ON K1N 6N5 , Canada . Email: dpratt@uottawa.ca; b Department of Chemistry , University of Michigan , Ann Arbor , MI 48109 , USA . Email: crjsteph@umich.edu

## Abstract

Hydrogen atom transfer (HAT) is the mechanism by which the vast majority of radical-trapping antioxidants (RTAs), such as hindered phenols, inhibit autoxidation. As such, at least one weak O–H bond is the key structural feature which underlies the reactivity of phenolic RTAs. We recently observed that quinone methide dimers (QMDs) synthesized from hindered phenols are significantly more reactive RTAs than the phenols themselves despite lacking O–H bonds. Herein we describe our efforts to elucidate the mechanism by which they inhibit autoxidation. Four possible reaction paths were considered: (1) HAT from the C–H bonds on the carbon atoms which link the quinone methide moieties; (2) tautomerization or hydration of the quinone methide(s) *in situ* followed by HAT from the resultant phenolic O–H; (3) direct addition of peroxyl radicals to the quinone methide(s), and (4) homolysis of the weak central C–C bond in the QMD followed by combination of the resultant persistent phenoxyl radicals with peroxyl radicals. The insensitivity of the reactivity of the QMDs to substituent effects, solvent effects and a lack of kinetic isotope effects rule out the HAT reactions (mechanisms 1 and 2). Simple (monomeric) quinone methides, to which peroxyl radicals add, were found to be *ca.* 100-fold less reactive than the QMDs, ruling out mechanism 3. These facts, combined with the poor RTA activity we observe for a QMD with a stronger central C–C bond, support mechanism 4. The lack of solvent effects on the RTA activity of QMDs suggests that they may find application as additives to materials which contain H-bonding accepting moieties that can dramatically suppress the reactivity of conventional RTAs, such as phenols. This reactivity does not extend to biological membranes owing to the increased microviscosity of the phospholipid bilayer, which suppresses QMD dissociation in favour of recombination. Interestingly, the simple QMs were found to be very good RTAs in phospholipid bilayers – besting even the most potent form of vitamin E.

## Abstract 2

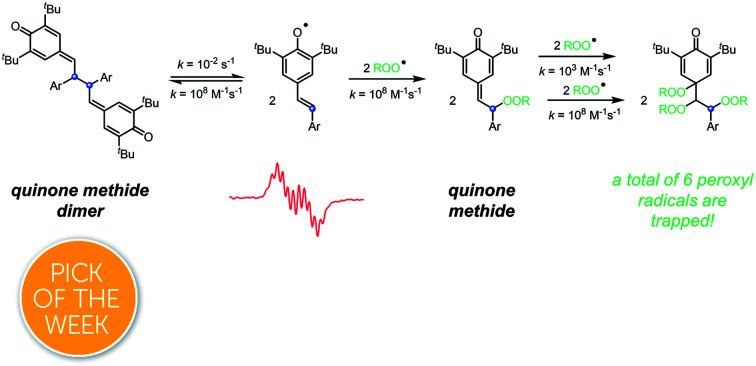
Quinone method dimers, (bio)synthetic intermediates en route to many naturally products derived from resveratrol, are potent radical-trapping antioxidants, besting the phenols from which they are derived and to which they can be converted.

## Introduction

**Fig. 1:**
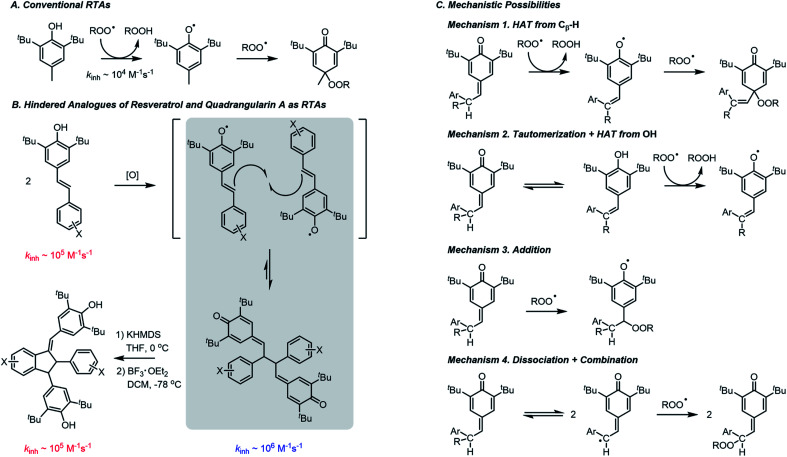
(A) Conventional reactivity of RTAs; (B) oxidation of stilbenoid phenols to form quinone methide dimers, followed by their base-mediated isomerization and Lewis acid-catalyzed Friedel–Crafts cyclization to form quadrangularin A analogues; (C) mechanisms by which QMDs may trap peroxyls. For clarity, half of the dimer is represented as R.

The efficient inhibition of autoxidation continues to be a widely-pursued objective, given the indispensability of hydrocarbon-based products (either petroleum-derived or plant-derived) in day-to-day life and their propensity to undergo autoxidation. Nevertheless, the inhibitors which are commonly added to these products have remained essentially the same for decades.^1^,^2^ The most ubiquitous radical-trapping antioxidants (RTAs) are hindered phenols, such as butylated hydroxytoluene (BHT). BHT and related phenols react with the peroxyl radicals (ROO˙) that propagate the autoxidation chain reaction. The mechanism is well understood to involve hydrogen atom transfer (HAT) from the phenolic O–H to a chain-carrying peroxyl radical followed by rapid combination of the resultant phenoxyl radical with a second peroxyl radical (Fig. 1A).^2^ A vast literature has established that substituents can have a substantial impact on the rate of the initial H-atom transfer, which is characterized by the inhibition rate constant (*k*_inh_).^2^,^3^ Electron-donating groups accelerate the rate as they stabilize the electron-deficient phenoxyl radical^4^ and the transition state leading to its formation, whereas electron-withdrawing groups have the opposite effect.

As part of ongoing efforts to better understand the antioxidant activity of resveratrol and its oligomers,^5^ we found that the dimeric quinone methides which we employed as the key synthetic intermediates en route to resveratrol dimers are significantly better RTAs than their phenolic precursors or products (Fig. 1B).^6^ This was entirely unexpected given that the quinone methide dimers (hereafter QMDs) lack phenolic H-atoms. In our preliminary report, we suggested four possible reaction paths that would account for this observation (Fig. 1C): (1) HAT from the C–H bonds on the carbon atoms which link the quinone methide moieties; (2) tautomerization or hydration of the quinone methide(s) followed by HAT from the resultant phenolic O–H; (3) direct addition of peroxyl radicals to the quinone methide to form persistent phenoxyl radicals, and (4) homolysis of the weak central C–C bond in the QMDs followed by combination of the resultant persistent phenoxyl radicals with peroxyl radicals. Herein, we offer the details of our efforts to elucidate the mechanism.

## Results

### Synthetic procedures

**Chart 1:**
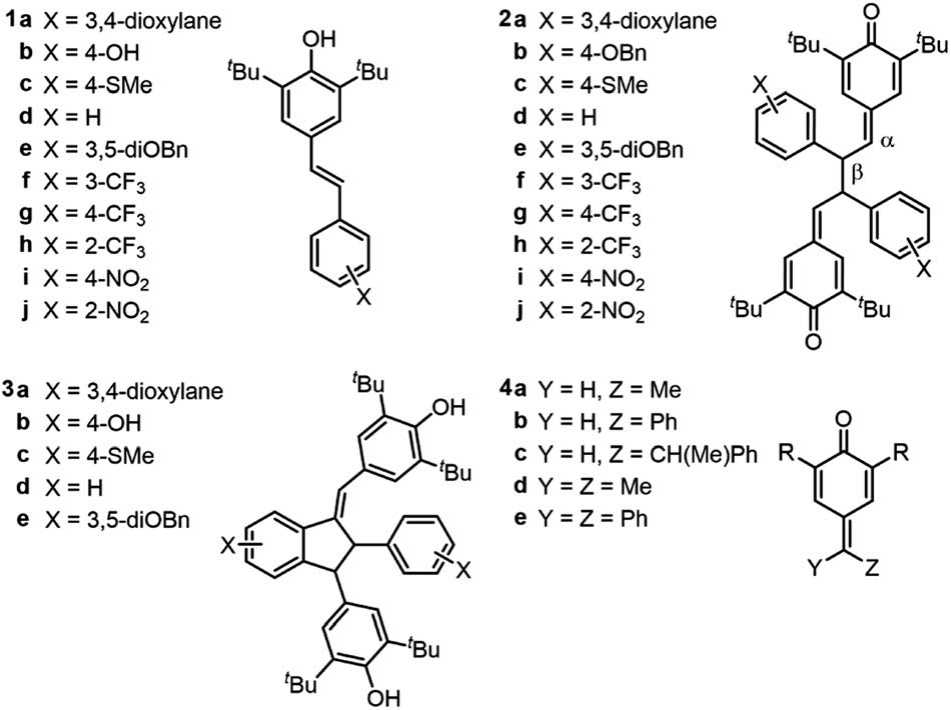
Relevant stilbenoid phenols (**1**), quinone methide dimers (QMDs, **2**), quadrangularin A analogues (**3**), and quinone methides (**4**).

A set of hindered resveratrol analogues (**1a–j**) were synthesized wherein the resorcinol ring was replaced with aryl rings bearing substituents of differing electronics (Chart 1). These phenols were dimerized *via* anodic oxidation in the presence of 2,6-lutidine to produce quinone methide dimers (**2a–j**).^6^ After base-mediated isomerization of one quinone methide, Lewis acid activation and Friedel–Crafts cyclization afforded a series of substituted quadrangularin (quad) A analogues (**3a–e**).

### Inhibited autoxidations

**Fig. 2:**
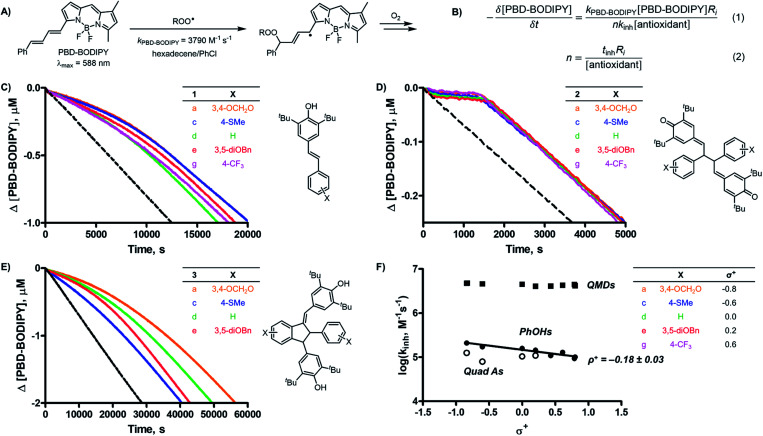
(A) PBD-BODIPY serves as the signal carrier in 1-hexadecene autoxidations; (B) determination of inhibition rate constants (*k*_inh_) and stoichiometries (*n*) for reactions of inhibitors with chain-carrying peroxyl radicals from initial rates and inhibition periods (*t*_inh_). Co-autoxidations of 1-hexadecene (2.9 M) and PBD-BODIPY (10 μM) initiated by AIBN (6 mM) in chlorobenzene at 37 °C (dashed black traces in (C–E)) and inhibited by 5 μM **1** (C), 1 μM **2** (D), and 5 μM **3** (E) (colour traces); (F) linear free energy relationships for **1**, **2**, and **3** at 37 °C.

The reactivities of the QMDs as RTAs were determined by the classical inhibited autoxidation approach^7^ utilizing a co-autoxidation of 1-hexadecene and PBD-BODIPY (Fig. 2A).^8^ Inclusion of the latter enables monitoring of reaction progress *via* conventional spectrophotometry by loss of its absorbance at *λ*_max_ = 588 nm upon addition of peroxyl radicals to its 1-phenylbutadiene moiety. Rate constants for the reaction of peroxyl radicals with the 10 QMDs (**2**), their 10 precursor stilbenoid phenols (**1**), and 5 quadrangularin A analogues derived therefrom (**3**) (*k*_inh_) and corresponding reaction stoichiometries (*n*) were determined from the initial rate and inhibited period (*t*_inh_) of the inhibited autoxidations, respectively, according to the expressions in Fig. 2B. Representative results are shown in Fig. 2C–E for series of equivalently-substituted **1**, **2** and **3**, respectively.

All 10 of the QMDs studied were determined to be excellent RTAs, with *k*_inh_ ∼ 4 × 10^6^ M^–1^ s^–1^ at 37 °C. The striking difference in reactivity between the QMDs and the equivalently-substituted precursor stilbenoid phenols and product quadrangularin A analogues is evident simply upon consideration of the raw inhibited autoxidation reaction progress data (compare the prominent inhibited periods in Fig. 2D to the retarded autoxidations in Fig. 2C and E, respectively), which amount to a difference in *k*_inh_ of >10-fold. This is even more impressive given that the stilbenoid phenols from which the QMDs are derived are already ∼10-fold more reactive than the archetype hindered phenolic RTAs, such as BHT (*k*_inh_ = 2 × 10^4^ M^–1^ s^–1^).^9^ Indeed, the reactivity of the QMDs under these conditions is on par with that of α-tocopherol, the most potent form of vitamin E and among the most reactive RTAs .^9^

The observed inhibition periods correspond to the trapping of ∼2 radicals by the QMDs, which is similar to the precursor phenols, but roughly half that observed for the quadrangularin A analogues. The stoichiometries of the phenols are consistent with precedent (*cf.*Fig. 1A),^9^ and since the quadrangularin A analogues contain two phenolic moieties, it follows that they trap twice as many peroxyl radicals. We also carried out inhibited autoxidations at 70 °C and found that the superiority of QMDs compared to their precursor and product phenols is maintained (see ESI for the raw data†). However, pushing the temperature to 100 °C reveals a noticeable drop in reactivity. The stoichiometry exhibited by the QMDs was also found to attenuate at progressively higher temperatures; see the kinetic data summarized in Table 1.

**Table 1:** Inhibition rate constants (*k*_inh_) and stoichiometries (*n*) for substituted QMDs (**2**), stilbenoid phenols (**1**) and quadrangularin A analogues (**3**) measured from inhibited co-autoxidations of 1-hexadecene (2.9 M) and PBD-BODIPY (10 μM) initiated by AIBN (6 mM) at 37 °C, ^*t*^BuOO^*t*^Bu (87 mM) at 70 °C, and dicumyl peroxide (1 mM) at 100 °C

**QMDs**		37 °C		70 °C		100 °C	
		***k***_**inh**_ **(10** ^**5**^ **M** ^**–1**^ **s** ^**–1**^ **)**	***n***	***k***_**inh**_ **(10** ^**5**^ **M** ^**–1**^ **s** ^**–1**^ **)**	***n***	***k***_**inh**_ **(10** ^**5**^ **M** ^**–1**^ **s** ^**–1**^ **)**	***n***
3,4-Dioxyl	**2a**	48 ± 9	1.8 ± 0.1	59 ± 10	1.6 ± 0.1	9.9 ± 0.6	0.44 ± 0.01
4-OBn	**2b**	48 ± 8	1.7 ± 0.1	44 ± 2	1.6 ± 0.1	ND	ND
4-SMe	**2c**	46 ± 6	1.8 ± 0.1	59 ± 4	1.4 ± 0.1	10 ± 1	0.49 ± 0.01
H	**2d**	45 ± 3	1.8 ± 0.1	86 ± 6	1.7 ± 0.1	17 ± 2	0.52 ± 0.01
3,5-(OBn)_2_	**2e**	40 ± 8	2.0 ± 0.1	120 ± 10	1.9 ± 0.1	22 ± 2	0.61 ± 0.05
3-CF_3_	**2f**	41 ± 4	1.8 ± 0.1	110 ± 10	1.6 ± 0.1	ND	ND
4-CF_3_	**2g**	43 ± 9	1.8 ± 0.1	98 ± 10	1.6 ± 0.1	14 ± 2	0.50 ± 0.02
2-CF_3_	**2h**	45 ± 3	1.9 ± 0.1	62 ± 8	1.4 ± 0.1	ND	ND
4-NO_2_	**2i**	45 ± 6	1.3 ± 0.1	40 ± 10	1.2 ± 0.1	ND	ND
2-NO_2_	**2j**	42 ± 9	1.5 ± 0.1	47 ± 10	1.0 ± 0.1	ND	ND

**Stilbenoid phenols**		***k***_**inh**_ **(10** ^**5**^ **M** ^**–1**^ **s** ^**–1**^ **)**	***n***	***k***_**inh**_ **(10** ^**5**^ **M** ^**–1**^ **s** ^**–1**^ **)**	***n***	***k***_**inh**_ **(10** ^**5**^ **M** ^**–1**^ **s** ^**–1**^ **)**	***n***
3,4-Dioxyl	**1a**	2.1 ± 0.2	2.2 ± 0.1	6.2 ± 0.3	1.6 ± 0.1	2.9 ± 0.1	1.1 ± 0.1
4-OH	**1b**	3.1 ± 0.2	2.1 ± 0.1	11 ± 1	1.9 ± 0.1	ND	ND
4-SMe	**1c**	1.7 ± 0.1	2.2 ± 0.1	5.9 ± 0.7	1.5 ± 0.1	2.8 ± 0.3	1.1 ± 0.1
H	**1d**	1.6 ± 0.1	2.0 ± 0.3	4.6 ± 0.4	1.5 ± 0.1	2.6 ± 0.2	1.0 ± 0.1
3,5-(OBn)_2_	**1e**	1.4 ± 0.1	2.3 ± 0.1	19 ± 5	1.7 ± 0.1	2.5 ± 0.1	1.3 ± 0.1
3-CF_3_	**1f**	1.1 ± 0.2	2.3 ± 0.3	3.7 ± 0.1	1.5 ± 0.1	ND	ND
4-CF_3_	**1g**	1.3 ± 0.1	2.3 ± 0.1	3.5 ± 0.4	1.6 ± 0.2	2.3 ± 0.2	1.2 ± 0.1
2-CF_3_	**1h**	1.0 ± 0.1	2.3 ± 0.3	3.6 ± 0.4	1.3 ± 0.1	ND	ND
4-NO_2_	**1i**	1.0 ± 0.1	2.3 ± 0.1	2.8 ± 0.3	1.6 ± 0.1	ND	ND
2-NO_2_	**1j**	1.0 ± 0.1	2.3 ± 0.1	2.7 ± 0.2	1.4 ± 0.1	ND	ND

**Quad A analogues**		***k***_**inh**_ **(10** ^**5**^ **M** ^**–1**^ **s** ^**–1**^ **)**	***n***	***k***_**inh**_ **(10** ^**5**^ **M** ^**–1**^ **s** ^**–1**^ **)**	***n***	***k***_**inh**_ **(10** ^**5**^ **M** ^**–1**^ **s** ^**–1**^ **)**	***n***
3,4-Dioxyl	**3a**	1.3 ± 0.1	4.1 ± 0.1	2.8 ± 0.3	4.2 ± 0.4	1.9 ± 0.2	3.6 ± 0.1
4-OH	**3b**	2.6 ± 0.4	5.5 ± 0.5	16 ± 1	6.3 ± 0.4	ND	ND
4-SMe	**3c**	0.8 ± 0.1	3.8 ± 0.1	1.2 ± 0.1	3.5 ± 0.3	1.6 ± 0.1	2.0 ± 0.1
H	**3d**	1.0 ± 0.1	4.1 ± 0.3	2.2 ± 0.2	4.0 ± 0.3	1.7 ± 0.1	2.9 ± 0.1
3,5-(OBn)_2_	**3e**	1.1 ± 0.1	3.8 ± 0.3	2.7 ± 0.4	3.5 ± 0.4	1.9 ± 0.1	2.8 ± 0.1

### Mechanistic studies

**Fig. 3:**
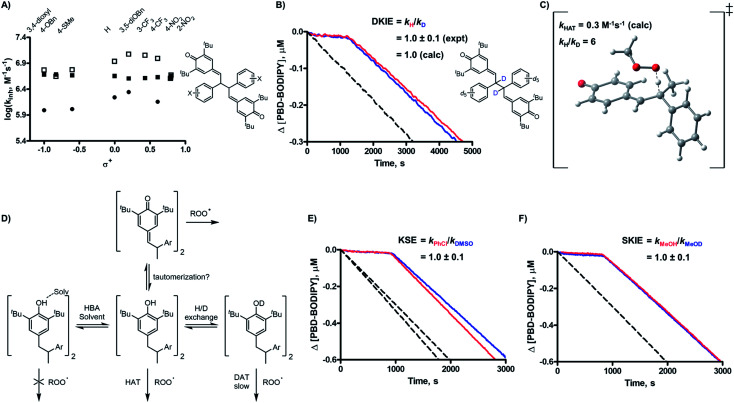
(A) Linear free energy relationships (log(*k*_inh_) *vs. σ*^+^) at 37 (■), 70 (□), and 100 ([black circle]) °C; (B) absence of deuterium kinetic isotope effect (DKIE) on the reactivity of **2d** in hexadecene/PBD-BODIPY co-autoxidations in PhCl at 37 °C, [**2d**] and [**2d**-*d*_12_] = 1 μM; (C) computed TS structure for hydrogen atom transfer and corresponding computed DKIE; (D) scheme depicting the effect of H-bond-accepting solvents (Solv) and deuterated solvent on activity of phenolic RTAs; (E) absence of kinetic solvent effect (KSE) in PhCl *vs.* 2 : 1 PhCl–DMSO (dioxane/PhCl, 37 °C, [**2d**] = 1 μM); (F) absence of solvent kinetic isotope effect (SKIE) in 1% MeOH *vs.* 1% MeOD (dioxane/PhCl, 37 °C, [**2d**] = 1 μM).

Without a labile H-atom, the mechanism by which QMDs act as RTAs was not immediately obvious. The lack of substituent effects on the reactivity of the QMDs (*ρ*^+^ ∼ 0 at 37 °C, Fig. 2F) is in direct contrast to the sensitivity observed for the precursor phenols (*ρ*^+^ = –0.18 at 37 °C), which is consistent with known structure–reactivity relationships for phenolic RTAs, in general (*vide supra*). The reactivities of the quadrangularin A analogues, which are also phenols, are lower than the precursor stilbenoid phenols and essentially independent of their substitution. This difference can be rationalized based upon the reduced conjugation between the substituted phenyl rings and the reactive hindered phenolic moieties in the quadrangularin A analogues relative to the stilbenoid phenols, which are fully conjugated (see ESI for the calculated minimum energy structures†).^10^ Upon increasing the temperature, the trends among the two sets of phenols remain consistent (see ESI for the plots†), but those for the QMDs change – demonstrating a slight positive correlation at 70 °C which increases slightly along with the diminution of reactivity at 100 °C (Fig. 3A).

The first mechanistic possibility we considered involves H-atom transfer from the benzylic positions adjacent to the methide carbons of the QMD. Although HAT from a benzylic carbon to a peroxyl radical is generally a sluggish reaction (*k* ∼ 1 M^–1^ s^–1^),^11^ in this case HAT restores the aromaticity of one of the aryl rings, which suggests it may be significantly enhanced. To probe this mechanism, an analogue of **2d** was synthesized wherein both benzylic H-atoms were replaced with D-atoms (structure shown in Fig. 3B; for synthetic details see Experimental section), and inhibited autoxidations were carried out as above.^12^ The results of this experiment (shown in Fig. 3B) revealed no kinetic isotope effect. Corresponding DFT computations on a model reaction (HAT from an analogous monomeric quinone methide to a methylperoxyl radical, shown in Fig. 3C) yield *k*_HAT_ = 0.3 M^–1^ s^–1^ from Δ*G*^‡^ = 20.9 kcal mol^–1^ and *k*_H_/*k*_D_ = 6.^13^ Clearly, this mechanism fails to account for the reactivity of the QMDs.

The second mechanistic possibility we considered involves *in situ* tautomerization of the QMD to a stilbenoid phenol that can react with peroxyl radicals (Fig. 1C). The simple fact that the *k*_inh_ values for the QMDs exceed that of the precursor stilbenoid phenols by more than an order of magnitude strongly suggests that a phenol is not involved in the mechanism;^6^ however, we sought corroborating evidence from kinetic solvent effect (KSE^14^) and solvent kinetic isotope effect (SKIE) experiments (see Fig. 3D). Thus, we carried out co-autoxidations of dioxane and PBD-BODIPY in chlorobenzene (PhCl) and a 2 : 1 PhCl : DMSO mixture. Previous studies^15^ conducted under identical conditions have shown a predictable attenuation of *k*_inh_ for HAT from X–H groups to peroxyl radicals due to a combination of the H-bond accepting capacities of the substrate (1,4-dioxane; *β*H2 = 0.41) and co-solvent (DMSO; *β*H2 = 0.78).^15^ The results are shown in Fig. 3E, which reveal no suppression in the reactivity of the QMD in the presence of H-bond accepting (HBA) solvents. Furthermore, QMD-inhibited co-autoxidations of dioxane and PBD-BODIPY in PhCl to which 1% v/v MeOH or 1% v/v MeOD were added were indistinguishable (Fig. 3F), suggesting no exchangeable protons are involved in the RTA activity.^16^

**Fig. 4:**
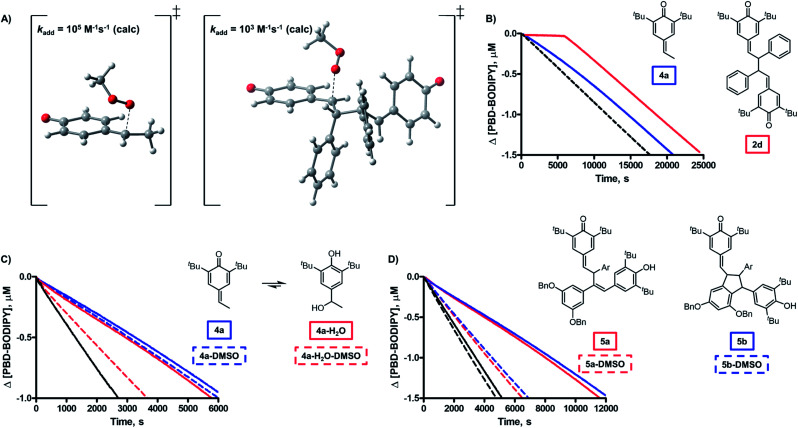
(A) Comparison of the transition state structures for addition to QM **4a** and QMD **2d** (^*t*^Bu groups omitted); (B) experimental evidence for the enhanced reactivity of QMDs over QMs (hexadecene/PhCl, 37 °C, [**2d**] = [**4a**] = 5 μM); (C) kinetic solvent effect (KSE) data in PhCl *vs.* 2 : 1 PhCl–DMSO for **4a** and **4a-H_2_O** (dioxane/PhCl, 37 °C, [**4a**] = [**4a-H_2_O**] = 50 μM); (D) kinetic solvent effect (KSE) data in PhCl *vs.* 2 : 1 PhCl–DMSO for **5a** and **5b** (dioxane/PhCl, 37 °C, [**5a**] = [**5b**] = 50 μM).

The third mechanistic possibility, addition of a peroxyl radical to one of the methide carbons of the QMD (Fig. 1C), has some precedent. Volodkin found quinone methides to be modest antioxidants (*k*_inh_ ∼ 10^3^ M^–1^ s^–1^),^17^ but since the rate constants they determined were much lower than those we found for the QMDs, and they investigated a limited number of structurally similar compounds, it is possible that quinone methide reactivity is enhanced when part of a QMD.^18^ When we calculated the TS for addition of a peroxyl radical to a model QMD (non-*tert*-butylated **2d**, see Fig. 4A), we found a barrier of Δ*G*^‡^ = 16.0 kcal mol^–1^, which corresponds to *k*_add_ = 9 × 10^2^ M^–1^ s^–1^ upon application of transition state theory – close to the reported values for simple QMs, but about 4500-fold lower than the *k*_inh_ values derived from the inhibited autoxidations.^19^ Aside from this discrepancy between theory and experiment, the addition mechanism is fully consistent with the lack of substituent effects on the reactivity at ambient temperature and the absence of kinetic solvent effects and either O–H/O–D or C–H/C–D kinetic isotope effects.

To provide insight on the origin of the possibility of an enhancement of quinone methide reactivity when part of a dimer, we calculated the TS for the addition of a peroxyl radical to a simple QM for comparison to the calculated data for the model QMD (Fig. 4A). To our surprise, the reaction was predicted to be *much faster* (Δ*G*^‡^ = 13.0 kcal mol^–1^, which was used to derive *k*_add_ = 1 × 10^5^ M^–1^ s^–1^). The discrepancy between the calculated rate constant for the reaction of **4a** and the reported experimental values (*e.g.* 1.4 × 10^3^ and 5.8 × 10^2^ M^–1^ s^–1^ at 60 °C in styrene and cumene, respectively)^17^ prompted us to synthesize it to corroborate Volodkin's results using our own approach and under the same conditions that the reactivity of the QMDs had been determined. Inhibited co-autoxidations of PBD-BODIPY in 1-hexadecene and of STY-BODIPY in cumene (both in chlorobenzene) yielded *k*_inh_ = 8.2 × 10^4^ and 7.2 × 10^3^ M^–1^ s^–1^, respectively, at 37 °C (see Fig. 4B for a direct comparison of the reactivity of **4a** and **2d**, tabulated in Table 2).^20^ We believe that these values are slightly higher than those obtained by Volodkin due to differences in the experimental conditions employed.^21^ Nevertheless, these results confirm that simple QMs are much less reactive than the QMDs.^22^

**Table 2:** Inhibition rate constants (*k*_inh_) and stoichiometries (*n*) of QMs and phenols during inhibited co-autoxidations of cumene (3.6 M) and STY-BODIPY (10 μM) or hexadecene (2.9 M) and PBD-BODIPY initiated by AIBN (6 mM) in chlorobenzene at 37 °C alongside calculated addition rate constants (*k*calcadd, gas phase, 37 °C) and literature *k*_inh_ values (*k*litinh, cumene or styrene, 60 °C)

	Y	Z	*k* calc add (10^4^ M^–1^ s^–1^)	*k* lit inh ^*a*^ (10^4^ M^–1^ s^–1^)	*n* ^*a*^	*k* hexadecene inh (10^4^ M^–1^ s^–1^)	*n*	*k* cumene inh (10^4^ M^–1^ s^–1^)	*n*
**4e**	Me	Me	80	1.6	0.9	ND		ND	
**4a**	H	Me	10	0.058	1.1	8.2 ± 0.2	2.0 ± 0.1	0.72 ± 0.03	2.2 ± 0.1
**4a-H_2_O** ^*c*^			NA	ND		8.3 ± 0.6	2.0^*b*^	0.84 ± 0.09	2.1 ± 0.1
**4b**	H	Ph	0.01	0.018	0.5	Retards		0.34 ± 0.03	1.9 ± 0.1
**4f**	Ph	Ph	0.0002	Does not inhibit		ND		ND	
**5a** ^*c*^			ND			8.1 ± 0.2	2.2 ± 0.1	0.75 ± 0.06	2.4 ± 0.1
**5b** ^*c*^			0.01			6.4 ± 0.1	2.4 ± 0.1	1.10 ± 0.03	2.4 ± 0.1

^*a*^Data from ref. 17.

^*b*^Stoichiometry set to *n* = 2.0 to determine *k*_inh_, [**4a-H_2_O**] = 5 μM.

^*c*^The data primarily reflect HAT.

Given that the observed reactivity of **4a** was highly coincident with the corresponding hindered phenols, we considered that the simple QMs undergo hydration *in situ* to produce hindered phenols that are the active RTAs, and that the QMDs are simply more resistant to hydration – making them appear more reactive. Thus, we prepared the hydrated form of **4a** and evaluated its reactivity in a 1-hexadecene/PBD-BODIPY co-autoxidation to find nearly indistinguishable reactivity to that of **4a**. To confirm that **4a** and hydrated **4a** react *via* different mechanisms, we carried out additional experiments wherein DMSO was added as a co-solvent. While the reactivity of **4a** was unchanged, a significant suppression in the reactivity of the hydrated **4a** was observed, consistent with the sequestration of the phenolic H-atom as part of an H-bonded complex with DMSO (Fig. 4C). We were also able to monitor the QM chromophore (*λ*_max_ ∼ 300 nm) by UV-vis spectrophotometry and found that it disappeared steadily during the inhibited period of the autoxidation (Fig. 6D).

The confirmation of the lackluster – but authentic – RTA activity of the quinone methide **4a** suggests that there is something special about the QMD structure which confers greater reactivity compared to monomeric quinone methides. To provide further insight on this point, we carried out autoxidations inhibited by the tautomerized QMD precursor to quadrangularin A analogue **5a** and an isomer thereof (**5b**) – both of which contain one hindered phenol and one quinone methide moiety. Both compounds exhibited kinetics consistent with the phenolic moiety (refer to Table 2 and Fig. 4D), in agreement with the larger *k*_inh_ of the phenols compared to the quinone methides.

**Fig. 5:**
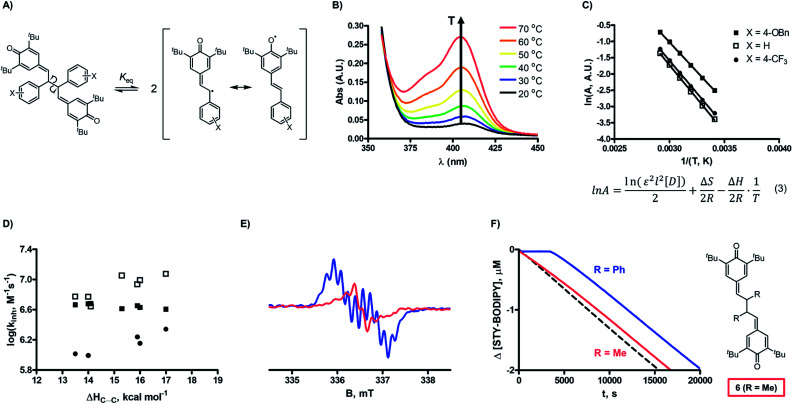
(A) Dissociation equilibrium for substituted QMDs (**2**) and key resonance structures of the resultant radical monomers; (B) UV-visible absorbance spectra of the phenoxyl radical derived from **2d** as a function of temperature; (C) representative Van't Hoff plots used to determine Δ*H*_C–C_; (D) relationship between log(*k*_inh_) and Δ*H*_C–C_ at 37 (■), 70 (□), and 100 ([black circle]) °C for *meta*- and *para*-substituted QMDs; (E) EPR spectra of the radicals present in solutions of **2d** (1 mM, blue) and **6** (10 mM, red) in benzene at 20 °C; (F) comparison of diaryl (**6_R=Ph_**, blue) *vs.* dialkyl (**6_R=Me_**, red) QMDs (5 μM) as inhibitiors of the AIBN-initiated autoxidation of cumene in PhCl at 37 °C.

The fourth mechanistic possibility (Fig. 1C) involves trapping of peroxyl radicals by combination with the small amount of persistent phenoxyl radical that is in equilibrium with the QMD (Fig. 5A).^23^,^24^ We had previously shown that QMD **2e** does not possess the weakest C–C bond (with a BDE of 17.0 *vs.* 6.1 kcal mol^–1^),^25^ it is sufficiently weak that a relevant concentration of phenoxyl radical exists at ambient temperatures. Based upon the equilibrium constant we previously reported for **2e** (*K*_eq_ = 5.5 × 10^–10^ M at 37 °C),^24^ the phenoxyl radical concentration at the beginning of an autoxidation inhibited by 1 μM of the QMD is ∼23 nM. If establishment of this equilibrium is fast relative to propagation of the autoxidation, then *k*_inh_[QMD] = *k*_comb_[phenoxyl], such that *k*_comb_ ∼ 43(4.0 × 10^6^ M^–1^ s^–1^) ∼ 1.7 × 10^8^ M^–1^ s^–1^. This value is in excellent agreement with findings of Jonsson *et al.*, who determined rate constants ranging from 1 × 10^8^ M^–1^ s^–1^ to 5 × 10^8^ M^–1^ s^–1^ for the reactions of various stabilized phenoxyl radicals with peroxyl radicals derived from ^i^PrOH in water (the effects of non-viscous solvents are generally negligible on radical combination reactions).^26^

Since we have no information on how substituents impact the QMD-phenoxyl radical equilibrium (Fig. 5A), we investigated it by UV-vis spectrophotometry at different temperatures (representative plot shown in Fig. 5B), enabling determination of the C–C BDEs (Δ*H*_C–C_, tabulated in the ESI†) from the resultant Van't Hoff plots (*e.g.*Fig. 5C).^27^ The BDEs of a broader series of QMDs were also computed using DFT and dispersion-corrected DFT (see ESI†), and we find that the trend in the sensitivity of Δ*H*_C–C_ to the substituent is similar to that of the experimental values, with derivatives substituted with electron-donating groups possessing weaker bonds. These data imply that electron-donating substituents should enhance RTA activity, but this is not observed. In fact, the relationship between log *k*_inh_ and Δ*H*_C–C_ (Fig. 5D) shows the opposite trend – at elevated temperatures, at least.

Nevertheless, to probe whether this pre-equilibrium is relevant, we synthesized a QMD bearing alkyl substituents *in lieu* of aryl substituents (**6**, structure shown in Fig. 5F) in the anticipation that it would have a stronger central C–C bond. Indeed, we could find no evidence for phenoxyl radicals in the UV/vis spectra of the QMD up to 100 °C even though the predicted spectrum of the radical suggests *λ*_max_ = 325 nm (see ESI†). Furthermore, we were unable to identify crossover products when equivalent amounts of **2d** and **6** were heated together (see ESI for spectral data†). Indeed, only poorly resolved (low intensity) spectra were obtained when concentrated samples of **6** (10 mM) were placed in the cavity of an EPR spectrometer, in contrast to the strong signals observed from samples of **2d** (1 mM, see Fig. 5E), which can be readily identified as being fully consistent with the radicals derived therefrom (see ESI†). Nonetheless, integration of the spectra observed from samples of **6** yields *K*_eq_ = 4.2 × 10^–13^ M at 20 °C. This can be directly compared with a value of *K*_eq_ = 1.7 × 10^–10^ M at 20 °C that we obtained with **2d**, and assuming Δ*S* is similar for C–C bond homolysis in **2d** and **6**, suggests that the difference in their C–C BDEs is at least 3.5 kcal mol^–1^. Most importantly, this compound is a very poor RTA (*k*_inh_ ∼ 2 × 10^3^ M^–1^ s^–1^)^28^ compared to the other QMDs (see Fig. 5F) and is even several-fold worse than the simple QM **4a**.

## Discussion

We recently prepared QMD **2e** as the key intermediate in the total synthesis of pallidol and quadrangularin A – natural products resulting from the oxidative dimerization of resveratrol.^6^ We were very surprised to find that **2e** and related QMDs are potent RTAs (*k*_inh_ ∼ 4 × 10^6^ M^–1^ s^–1^ at 37 °C); *ca.* 10-fold more reactive than the phenols from which they are derived. In fact, the QMDs were similarly reactive to α-tocopherol – the most biologically active form of vitamin E and the standard to which all other RTAs are compared – despite lacking labile H-atoms, the key structural feature of canonical RTAs.

The preparation of **2e** and its subsequent stereoselective conversion to pallidol and quadrangularin A was enabled by its reversible fragmentation to two persistent phenoxyl radicals.^5^,^24^ The foregoing mechanistic investigations suggest that this equilibrium is also responsible for the impressive RTA activity of **2e** and related QMDs. Although the persistent phenoxyl radicals are present in small quantities at equilibrium (*ca.* 1% of the QMD under the conditions which were investigated here), their combination with peroxyl radicals is quite rapid (*k*_inh_ ∼ 2 × 10^8^ M^–1^ s^–1^ at 37 °C). Thus, one molecule of QMD can be expected to trap two peroxyl radicals – consistent with the inhibition times observed in the QMD-inhibited autoxidations, from which *n* ∼ 2 were derived.

**Fig. 6:**
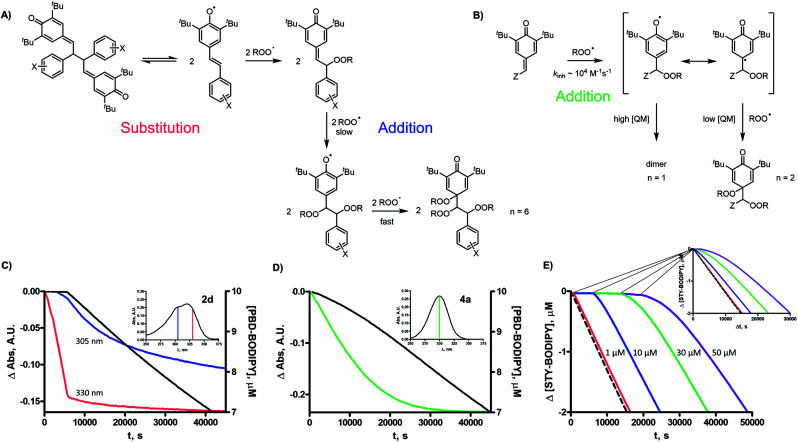
(A) Proposed mechanism for peroxyl radical-trapping by aryl-containing QMDs; (B) mechanism by which QMs trap peroxyls and the dependence of stoichiometry on [QM]; (C) spectral changes associated with inhibitors **2d** (blue and red) and (D) **4a** (green) in the co-autoxidations of 1-hexadecene (2.9 M) and PBD-BODIPY (black traces); (E) co-autoxidations of cumene (3.6 M) and STY-BODIPY (10 μM) initiated by AIBN (6 mM) in chlorobenzene at 37 °C inhibited by **2d** (1–50 μM) with an inset showing the second phase of inhibition translated to the uninhibited trace (broken line).

It is noteworthy that the resultant peroxidic adducts still contain QM moieties which can, in principle, react with additional peroxyl radicals. Indeed, simple QMs such as **4a** react with peroxyl radicals with rate constants similar to those of the hindered phenols from which they are derived (*e.g.***4a-H_2_O**), *k*_inh_ ∼ 8 × 10^4^ M^–1^ s^–1^ at 37 °C. Since the initial addition of a peroxyl radical forms a phenoxyl that can subsequently combine with a peroxyl radical (as do the phenoxyl radicals in equilibrium with the QMDs), the simple QMs were also found to trap 2 peroxyl radicals (see Fig. 6B). Thus, in principle, QMDs should trap a total of 6 peroxyl radicals, as is shown in the overall mechanistic scheme in Fig. 6A.

The inhibited autoxidation data for the isomerized QMD structures **5a** and **5b** (Fig. 4D) provide some insight to why the QMDs only appear to trap 2 peroxyl radicals in our experiments. Each of these compounds, which feature one phenolic moiety and one QM moiety react as if only the phenol were present with *n* ∼ 2 and *k*_inh_ = 8.1 × 10^4^ M^–1^ s^–1^ and 6.4 × 10^4^ M^–1^ s^–1^, respectively, which are subject to the same substantial KSEs. The lack of retardation of oxidation due to the QM moiety implies that it is significantly less reactive to peroxyl radicals than a simple monomeric QM. Indeed, when we monitored the QMD-inhibited autoxidations by looking at the whole spectrum rather than simply *λ*_max_ of PBD-BODIPY at 588 nm, we noticed consumption of the characteristic absorbance of the QM moiety at ∼300 nm only after the initial inhibited period, which coincided with consumption of the characteristic absorbance of the QMD moiety at ∼330 nm (Fig. 6C). This slower phase is consistent with the loss at ∼300 nm in autoxidations of the simple QM **4a** (Fig. 6D). In an attempt to quantify the slower reactivity of the more hindered QM moieties in the peroxyl adducts derived from the QMD (or **5a** or **5b**), we carried out inhibited autoxidations of cumene in PhCl with much higher concentrations of QMD (up to 50 μM). Upon doing so, we could indeed see a retardation of autoxidation beyond the initial inhibited period (Fig. 6E), which yielded *k*_inh_ ∼ 4 × 10^3^ M^–1^ s^–1^ – on par with a *k*_inh_ for a simple QM measured in cumene/PhCl at 37 °C. Ultimately, the most compelling result in support of the dissociation/combination mechanism is the observation that QMD **6**, which has a much stronger central C–C bond than **2**, is >1000-fold less reactive as an RTA than **2**. The reactivity of **6** is even lower than the simple QM **4a**, implying some steric effects on the addition of peroxyl radicals. This is consistent with computational predictions^22^ as well as the slower reactivity of the QM moieties in the tautomerized QMD precursor to quad A (**5a**) and isomer thereof (**5b**) relative to **4a**.

The one piece of mechanistic data that is not, at first glance, fully consistent with the dissociation/combination mechanism is the erosion in RTA activity with increasing temperature. Increasing the temperature shifts the QMD/radical equilibrium toward phenoxyl radical formation, which should improve peroxyl radical-trapping, but instead only a slight increase is observed on going from 37 °C to 70 °C and then a noticeable drop on going from 70 °C to 100 °C. We believe this simply reflects the reversible nature of the phenoxyl-peroxyl radical combination. It is well known that the activities of hindered phenolic RTAs diminish at elevated temperatures and that *n* values erode from 2 to 1 due to the reversible nature of the phenoxyl-peroxyl radical combination step that follows the initial H-atom transfer from phenol to peroxyl radical.^29^ The same phenoxyl-peroxyl combination step features here. The fact that the erosion in reactivity is not as severe for the more electron-poor QMDs (*cf.*Fig. 5D) presumably reflects a stronger C–O bond in the phenoxyl-peroxyl adduct.

The step-wise homolytic substitution mechanism of the QMDs is reminiscent of that by which the dimeric form of the Ciba (now BASF) antioxidant Irganox HP-136 reacts. The HP-136 dimer was determined to have largely solvent-independent RTA kinetics,^30^ with *k*_inh_ = 4.3 × 10^5^ M^–1^ s^–1^ and *n* = 0.8 from styrene autoxidations in chlorobenzene at 30 °C.^31^ The QMDs are therefore roughly 10-fold more reactive than the HP-136 dimer, presumably due to their weaker central C–C bond: 14–17 kcal mol^–1^*vs.* 23 kcal mol^–1^ for (HP-136)_2_. Thus, the QMDs add to a short list of molecules which lack labile H-atoms but are nevertheless reactive RTAs. Two of the few other examples of which we are aware are tetrasulfides^32^ and trisulfide-1-oxides.^33^ These compounds undergo concerted (bimolecular) homolytic substitution by peroxyl radicals to produce stable and persistent perthiyl radicals that do not propagate the autoxidation, but combine to give tetrasulfides. Although these substitution reactions are also insensitive to solvent effects, they are *much* slower at ambient temperatures (*k*_inh_ ∼ 10^3^ and 10^4^ M^–1^ s^–1^ for the tetrasulfides and trisulfide-1-oxides, respectively, compared to *k*_inh_ ∼ 10^6^ M^–1^ s^–1^ for the QMDs at 37 °C), limiting their applications to elevated temperatures.

**Fig. 7:**
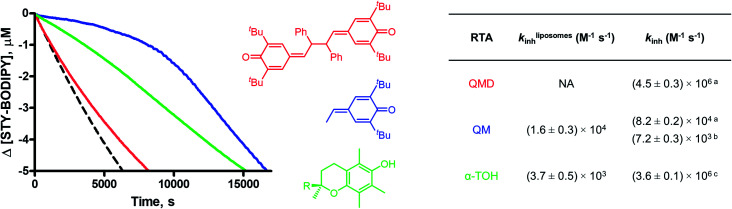
Co-autoxidations of unilammelar (100 nm) liposomes of egg phosphatidylcholine (1 mM) and STY-BODIPY (10 μM) initiated by DTUN (0.2 mM) in PBS buffer (pH = 7.4) at 37 °C (dashed black trace) and inhibited by 10 μM **2d** (red), **4a** (blue), and α-tocopherol (R = C_16_H_33_, green). The table compares *k*_inh_ values obtained from co-autoxidations for **2d** (QMD), **4a** (QM), and α-tocopherol (α-TOH, R = C_16_H_33_) in this system with those determined in hexadecene/PhCl (a), cumene/PhCl (b), and/or styrene/PhCl (c) at 37 °C.

**Fig. 8:**
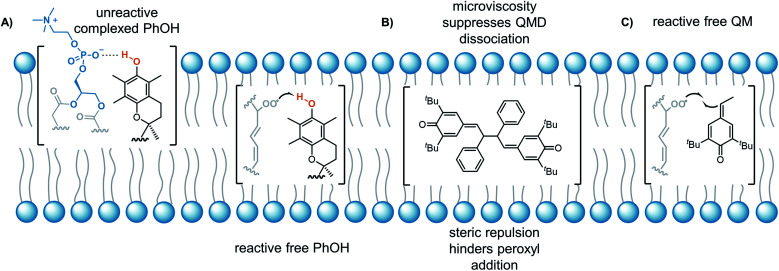
(A) Hydrogen-bonding of the labile H-atom of phenols such as α-TOH to the phosphate diester of phospholipids suppresses their reactivity to HAT; (B) the high microviscosity of the lipid environment promotes in-cage recombination of QMD-derived radicals, thereby suppressing the peroxyl radical substitution pathway. Addition of peroxyls to the QM moieties of the QMD is slowed by steric congestion imposed by the QMD scaffold; (C) simple QMs are more sterically accessible and highly effective RTAs.

The lack of solvent effects and high reactivity of the QMDs at ambient temperatures prompted us to carry out one additional set of experiments (see Fig. 7). It has recently come to light that the poor performance of phenolic antioxidants as inhibitors of lipid peroxidation in biological membranes is the result of strong H-bonding interactions between phenols and the phosphate diester moiety of the membrane phospholipids (Fig. 8A).^34^ Indeed, the reactivity of α-tocopherol (α-TOH), Nature's premier lipophilic RTA, toward peroxyl radicals is suppressed almost 1000-fold on going from chlorobenzene to a phospholipid bilayer (*k*_inh_ ∼ 10^6^*vs.* 10^3^ M^–1^ s^–1^). As such, we wondered if QMDs would be effective RTAs in this context, and evaluated their ability to suppress (phospho)lipid peroxidation using our recently described FENIX assay.34*a* Disappointingly, we found that they were quite poor inhibitors. Alas, the large microviscosity of the phospholipid bilayer^35^ presumably suppresses cage escape and combination of the QMD-derived phenoxyl radicals with lipid peroxyl radicals (Fig. 8B). This result further supports a stepwise substitution by peroxyl radicals on the QMDs in contrast to the concerted substitution favoured for the tetrasulfides and trisulfide-1-oxides, which do not depend on the viscosity of the medium. Alongside the foregoing experiment, we evaluated the RTA activity of a simple QM (**4a**) as a control. In fact, the QM turned out to be a much better RTA under these conditions, even besting α-TOH (see Fig. 7).^36^ The apparent *k*_inh_ value measured for **4a** in the phospholipid bilayer is scarcely different from that obtained in chlorobenzene – reinforcing that addition of peroxyl radicals is operative and medium independent (Fig. 8C). As far as we are aware, this is the first instance of the inhibition of (phospho)lipid peroxidation by a non-canonical (HAT)-like RTA, and is compelling indirect evidence underlining the deleterious role of H-bonding on the reactivity of canonical RTAs.

## Conclusions

This work establishes the mechanistic basis which underpins the surprisingly potent RTA activity of quinone methide dimers (QMDs) of the general formula **2**. These dimers, which are formed by the oxidative coupling of hindered stilbenoid phenols, have previously served as synthetic precursors to resveratrol oligomer natural products. Despite lacking any conventional RTA motif (*e.g.* a phenolic O–H bond), these QMDs are not only reactive in that capacity, but best both the phenolic compounds from which they are derived and to which they are converted. Our work corroborates previous data which shows that monomeric quinone methides are modest RTAs at best, and shows that the diaryl QMD scaffold is privileged in that it opens up a new mode of reactivity *via* its reversible fragmentation. The resultant persistent phenoxyl radicals can combine with peroxyl radicals leaving the second quinone methide moiety to react with additional peroxyl radicals. This mode of reactivity is not influenced by solvent, in contrast to traditional phenolic RTAs, whose H-atom transfer reactions are slowed by H-bonding interactions. This fact suggests that QMDs may be useful RTAs for applications in non-viscous H-bonding media. Experiments in phospholipid bilayers reveal that the high microviscosity of the lipid phase suppresses the reactivity of QMDs, but that the lacklustre reactivity of simple QMs, which undergo addition, is fully consistent with what is observed in solution – making it the only class of RTA reported to date whose solution phase reactivity directly translates to lipid bilayers.

## Experimental section

### General

All chemicals and solvents obtained from commercial suppliers were used as received unless indicated otherwise. 1-Hexadecene and cumene were purified and stored as previously described.^8^,^32^ The synthesis, purification, and characterization of substituted stilbenes (**1**), QMDs (**2**), and quad A analogues (**3**) as well as hybrid phenol–QMs (**5** and **6**) used in this study are described in previous reports.^5^,^6^ The synthesis, purification, and characterization of **4a**,^37^**4a-H_2_O**,^38^**4b** 39 and **S4** 40 were carried out according to literature reports.

#### 4-((1*S*,2*S*)-6-(Benzyloxy)-2-(4-(benzyloxy)phenyl)-3-((*E*)-3,5-di-*tert*-butyl-4-hydroxybenzylidene)-2,3-dihydro-1*H*-inden-1-yl)-2,6-di-*tert*-butylphenol (**S2**)

Compound **S1** (120 mg, 0.145 mmol) was dried down into a flame-dried round bottom flask charged with a stir bar. The atmosphere was evacuated and replaced with N_2_, and the starting material was dissolved in CH_2_Cl_2_ (14 mL, 0.01 M reaction concentration). The solution was cooled to the reaction temperature, and BF_3_·OEt_2_ (0.036 mL, 0.29 mmol, 2 equiv.) was added dropwise. The reaction was stirred for 3 hours, at which point it was raised from the ice bath and quenched *via* the addition of saturated NaHCO_3_. Once the reaction had thawed, it was poured into a separatory funnel, and the layers were separated. The aqueous layer was extracted with additional portions of CH_2_Cl_2_, and the combined organic layers were washed with brine, dried over magnesium sulfate, and concentrated under reduced pressure. The crude product was purified by column chromatography (10% to 50% DCM/hexanes) to afford compound **S2** (100 mg, 83% yield). ^1^H NMR (500 MHz, chloroform-*d*) *δ* 7.43–7.27 (m, 12H), 7.16 (d, *J* = 9.1 Hz, 1H), 7.14 (s, 2H), 7.01 (s, 2H), 6.98 (s, 1H), 6.87 (d, *J* = 8.9 Hz, 2H), 6.77 (m, 2H), 5.04 (s, 2H), 5.01 (s, 2H), 5.01 (s, 1H), 4.97 (s, 1H), 4.12 (s, 1H), 4.04 (s, 1H), 1.34 (s, 18H), 1.29 (s, 18H). ^13^C NMR (176 MHz, chloroform-*d*) *δ* 158.27, 158.05, 152.37, 152.33, 138.22, 137.43, 137.32, 137.20, 136.74, 135.58, 135.55, 134.49, 134.05, 128.70, 128.65, 128.06, 127.97, 127.56, 127.52, 127.17, 124.18, 123.96, 123.05, 116.37, 114.67, 112.94, 70.13, 70.03, 53.62, 50.27, 34.52, 34.47, 30.46, 30.45. IR (Neat): 3634, 3589, 2957, 2870, 1647, 1594, 1507, 1434, 1237, 1158, 1136, 1029, 734, 697 cm^–1^. HRMS (ESI) *m*/*z* calculated for C_58_H_66_O_4_^+^ ([M]^+^) 826.4956, found 826.4952.

#### (2*S*,3*S*)-1-((*E*)-3,5-Di-*tert*-butyl-4-hydroxybenzylidene)-3-(3,5-di-*tert*-butyl-4-hydroxyphenyl)-2-(4-hydroxyphenyl)-2,3-dihydro-1*H*-inden-5-ol (**3b**)

Compound **S2** (73 mg, 0.088 mmol) was dried down in a flame-dried round bottom flask charged with a stir bar. Pentamethylbenzene (134 mg, 0.88 mmol) was added in a single portion, and the solids were dissolved in dichloromethane (9 mL, 0.01 M reaction concentration) under an inert atmosphere. The reaction mixture was cooled to –78 °C, at which point BCl_3_ (0.53 mL, 0.53 mmol, 1.0 M in CH_2_Cl_2_) was added *via* syringe, turning the reaction mixture deep purple. The reaction was stirred at this temperature for 1.5 h, at which point it was lifted from the dry ice bath and quenched with saturated NaHCO_3_. The reaction was stirred vigorously while the ice thawed and until the reaction mixture stopped changing colour. Once the quench was complete (at this point the reaction was a pale-yellow colour), the reaction was poured into a separatory funnel containing DI H_2_O. The layers were separated, and the aqueous layer was extracted with additional CH_2_Cl_2_. The combined organic layers were washed with brine, dried over MgSO_4_ and concentrated under reduced pressure. The crude material was purified by flash chromatography using a 0 to 15% acetone in DCM gradient to afford compound **3b** (49 mg, 86% yield). ^1^H NMR (700 MHz, chloroform-*d*) *δ* 7.31 (d, *J* = 8.3 Hz, 2H), 7.12 (s, 2H), 7.12 (d, *J* = 8.1 Hz, 1H), 7.00 (s, 2H), 6.95 (s, 1H), 6.71 (d, *J* = 8.7 Hz, 2H), 6.63 (dd, *J* = 8.1, 2.8 Hz, 1H), 6.58 (d, *J* = 2.5 Hz, 1H), 5.01 (s, 1H), 4.97 (s, 1H), 4.60 (s, 1H), 4.52 (s, 1H), 4.08 (s, 1H), 4.00 (s, 1H), 1.33 (s, 18H), 1.29 (s, 18H). ^13^C NMR (176 MHz, chloroform-*d*) *δ* 154.79, 154.67, 152.38, 138.41, 137.24, 136.65, 135.60, 135.59, 134.47, 133.95, 127.86, 127.81, 127.36, 124.13, 123.91, 123.00, 116.67, 115.14, 113.55, 53.52, 50.30, 34.51, 34.46, 30.43, 30.41. IR (Neat): 3628, 2959, 1656, 1595, 1558, 1507, 1459, 1361, 1244, 1197, 1024, 878, 836, 668 cm^–1^. HRMS (ESI) *m*/*z* calculated for C_44_H_55_O_4_^+^ ([M + H]^+^) 647.4021, found 647.4029.

#### 2,6-Di-*tert*-butyl-4-(4-(3,5-di-*tert*-butyl-4-hydroxycyclohexa-2,5-dien-1-ylidene)-2,3-dimethylbutylidene)cyclohexa-2,5-dien-1-one (**6**)

Phenol **S4** (25 mg, 0.100 mmol) was added to a 10 mL reaction flask charged with a stir bar and KPF_6_ (74 mg, 0.4 mmol, 4.0 equiv.). The solids were dissolved in MeCN (8 mL) and 2,6-lutidine (12 μL, 0.1 mmol, 1.0 equiv.) was added to the reaction solution. See ESI† for details regarding the electrochemical setup. The reaction was stirred at 750 rpm for 1 h at a constant voltage of 0.8 V. Upon completion of the reaction (as judged by TLC), the electrodes were removed and rinsed into a collection flask with DCM (∼40 mL). The contents of the reaction vial were also rinsed into the collection flask. The solvent was removed under reduced pressure, the crude material was resuspended in DCM, and the electrolyte was filtered away with a plug of Celite. The filtrate was then concentrated and purified by column chromatography (1% to 10% EtOAc/hexanes) to afford compound **6** as a yellow foam (23.8 mg, 95% yield, ∼7 : 1 dr). ^1^H NMR (500 MHz, chloroform-*d*) *δ* 7.22 (d, *J* = 2.4 Hz, 1H, major diastereomer), 7.19 (d, *J* = 2.4 Hz, 1H, minor diastereomer), 6.84 (d, *J* = 2.4 Hz, 1H, minor diastereomer), 6.80 (d, *J* = 2.4 Hz, 1H, diastereomer), 6.10 (d, *J* = 10.1 Hz, 1H, major + minor diastereomers overlapped), 3.02 (ddd, *J* = 9.7, 8.0, 5.2 Hz, 1H, major diastereomer), 2.98–2.91 (m, 1H, minor diastereomer), 1.31 (s, 9H), 1.27 (s, 9H), 1.19 (d, *J* = 6.2 Hz, 3H). ^13^C NMR (176 MHz, chloroform-*d*) *δ* 152.22, 152.21, 145.37, 145.10, 137.79, 137.16, 136.19, 135.45, 134.56, 132.58, 127.44, 126.93, 126.40, 124.26, 124.01, 122.45, 116.78, 115.01, 113.50, 53.72, 50.45, 34.50, 34.45, 30.48, 30.45. HRMS (ESI) *m*/*z* calculated for C_34_H_51_O_2_ ([M + H]^+^) 491.3884, found 491.3879.

#### 2,6-Di-*tert*-butyl-4-(1-hydroxy-2-(phenyl-d_5_)ethyl-2,2-d_2_)phenol (**S6**)^23^

Benzylbromide-*d*_7_ (ref. 41) **S5** (1.00 g, 5.62 mmol) in ether (1.0 mL) was added over the course of 1 hour to a suspension of activated magnesium (115 mg, 4.82 mmol) in ether (5.0 mL) under a nitrogen atmosphere. The solution was heated at reflux for 2 hours to ensure complete formation of the Grignard reagent after which 3,5-di-*tert*-butyl-4-hydroxybenzaldehyde (360 mg, 1.61 mmol) was added dropwise to the reaction as a solution in ether (25 mL). The resulting solution was heated at reflux for 4 hours after which it was cooled to room temperature and quenched with aqueous acetic acid and diluted with ether. The organic phase was washed with water and brine, dried over MgSO_4_, filtered and concentrated *in vacuo*. The crude yellow oil was purified by flash column chromatography using 10% EtOAc in hexanes as the mobile phase to yield the final product as a light beige oil (0.320 g, 60%). ^1^H-NMR (400 MHz; CDCl_3_): *δ* 7.13 (s, 2H), 5.18 (s, 1H), 4.81 (s, 1H), 1.86 (d, *J* = 2.3 Hz, 1H), 1.44 (s, 18H). ^13^C NMR (101 MHz; CDCl_3_): *δ* 153.4, 135.9, 134.5, 122.9, 75.9, 34.5, 30.4. HRMS (EI, [M – H_2_O]^+^): *m*/*z* calcd for C_22_H_21_O_1_D_7_ 315.2580, found 315.2584.

#### (*E*)-2,6-Di-*tert*-butyl-4-(2-(phenyl-d_5_)vinyl-2-d)phenol (**1d**-*d*_6_)

*p*-Toluenesulfonic acid monohydrate (5.7 mg, 0.03 mmol) was added to a solution of 2,6-di-*tert*-butyl-4-(1-hydroxy-2-(phenyl-*d*_5_)ethyl-2,2-*d*_2_)phenol (200 mg, 0.60 mmol) in benzene (12.0 mL) under nitrogen and the solution was heated to reflux (85 °C) for 4 hours. After it was cooled to room temperature, the solution was diluted with ethyl acetate (50 mL) and washed with water and brine, dried over MgSO_4_, filtered and concentrated *in vacuo*. The crude yellow oil was purified by flash column chromatography using hexanes as the eluent to yield the final product as a white solid (0.130 g, 69%). ^1^H-NMR (400 MHz; CDCl_3_): *δ* 7.35 (s, 2H), 7.07 (s, 1H), 5.28 (s, 1H), 1.48 (s, 18H). ^13^C NMR (101 MHz; CDCl_3_): *δ* 154.0, 136.3, 129.6, 128.8, 123.6, 34.5, 30.4. HRMS (EI, [M+]): *m*/*z* calcd for C_22_H_23_OD_6_ 314.2517, found 314.2517.

#### 4,4′-(2,3-Bis(phenyl-d_5_)butane-1,4-diylidene-2,3-d_2_)bis(2,6-di-*tert*-butylcyclohexa-2,5-dien-1-one) (**2d**-*d*_12_)^23^

A solution of stilbene **1d**-*d*_6_ (25 mg, 0.080 mmol) in dry THF (0.9 mL) was cooled to 0 °C in an ice bath and purged with nitrogen for 5 minutes. KHMDS (0.090 mmol, 1 M in THF, 0.09 mL) was added slowly and the resulting bright yellow solution was stirred for 10 minutes at 0 °C. Ferrocenium hexafluorophosphate (28 mg, 0.090 mmol) was added in two 14 mg portions within a 15 minute interval. The resulting orange solution was stirred at 0 °C for 1 hour after which it was filtered through a pad of Celite, concentrated *in vacuo* and then purified by flash column chromatography using a gradient of 95 : 2.5 : 2.5 to 90 : 5:5 hexanes/EtOAc/DCM to obtain the final product as a yellow solid (19 mg, 76%). ^1^H-NMR (600 MHz; CDCl_3_): *δ* 7.15 (d, *J* = 2.4 Hz, 2H), 7.12 (d, *J* = 2.4 Hz, 2H), β-H's of quinone methides: 6.81 (minor diastereomer, d, *J* = 2.4 Hz, 2H), 6.69 (major diastereomer, d, *J* = 2.4 Hz, 2H), δ-H's of quinone methides: 6.49 (minor diastereomer, s, 2H), 6.38 (major diasteromer, s, 2H), ^*t*^Bu signals: 1.25 (s, 18H), 1.24 (s, 18H), 1.23 (s, 18H), 1.21 (s, 18H). ^13^C NMR (151 MHz; CDCl_3_): *δ* 186.6, 186.5, 149.1, 148.9, 147.6, 147.3, 145.4, 144.9, 140.9, 140.3, 134.7, 132.7, 132.0, 126.1, 125.9, 35.5, 35.5, 35.0, 34.9, 29.6, 29.6, 29.5. HRMS (ESI, [M + Na]^+^): *m*/*z* calcd for C_44_H_42_O_2_D_12_Na 649.4775, found 649.4774.

### General procedures for inhibited Co-autoxidations

The inhibited co-autoxidations were carried out following our reported methods.^8^,34a Autoxidations of 1-hexadecene (2.9 M) and PBD-BODIPY (10 μM) in chlorobenzene were initiated by AIBN (6 mM) at 37 °C, ^*t*^BuOO^*t*^Bu (87 mM) at 70 °C, or dicumyl peroxide (1 mM) at 100 °C. A 3.5 mL quartz cuvette was charged with PhCl (0.44 mL) and 1-hexadecene (2.00 mL). The cuvette was preheated to the desired temperature in a thermostated sample holder of a UV-vis spectrophotometer and allowed to equilibrate for approximately 15 min. To the cuvette was added PBD-BODIPY (12.5 μL of a 2.00 mM stock solution in 1,2,4-trichlorobenzene) and initiator (50 μL of a 300 mM stock solution of AIBN in chlorobenzene, 40 μL of neat ^*t*^BuOO^*t*^Bu, or 50 μL of a 50 mM stock solution of dicumyl peroxide in chlorobenzene). The solution was thoroughly mixed prior to monitoring the uninhibited co-autoxidation *via* the disappearance of PBD-BODIPY at 588 nm (37 °C), 587 nm (70 °C), or 586 nm (100 °C) for 5–10 min to ensure the reaction was proceeding at a constant rate. Finally, the antioxidant under investigation was added (5.0–10.0 μL of a 0.25 or 2.5 mM solution in chlorobenzene), the solution was mixed thoroughly, and the absorbance readings were resumed. The resulting Abs *vs.* time data were processed as previously reported.^8^ The rate of initiation (*R*_i_ = 1.30 × 10^–9^ M s^–1^ (37 °C), 1.26 × 10^–9^ M s^–1^ (70 °C), 6.58 × 10^–9^ M s^–1^ (100 °C)) and second order rate constant for propagation of the dye (*k*_PBD-BODIPY_ = 3792 M^–1^ s^–1^ (37 °C), 7633 M^–1^ s^–1^ (70 °C), 8283 M^–1^ s^–1^ (100 °C)) necessary to compute stoichiometric data (*n*) and inhibition rate constants (*k*_inh_) were determined using 2,2,5,7,8-pentamethylchromanol (PMC) as a standard, which has an established stoichiometry of 2.^42^ Similar experiments were conducted at 37 °C employing cumene (3.6 M) and STY-BODIPY (10 μM) initiated by AIBN (6 mM) in chlorobenzene. Reaction progress was monitored at 571 nm. The rate of initiation, also determined using PMC, was measured to be *R*_i_ = 2.28 × 10^–9^ M s^–1^ and the second order rate constant for propagation had been determined previously (*k*_STY-BODIPY_ = 141 M^–1^ s^–1^) (37 °C). Inhibited autoxidation experiments involving liposomes were conducted at 37 °C employing egg-phosphatidylcholine liposomes (1 mM), STY-BODIPY (10 μM), initiated by DTUN34*a* (0.2 mM) in PBS buffer (10 mM). Reaction progress was monitored at 565 nm. The rate of initiation, determined using PMC, was measured to be *R*_i_ = 2.29 × 10^–9^ M s^–1^ and the second order rate constant for propagation had been determined previously (*k*_STY-BODIPY_ = 894 M^–1^ s^–1^) (37 °C).

#### Determination of kinetic solvent effects (KSEs) in inhibited co-autoxidation experiments

Autoxidations of 1,4-dioxane (2.9 M) and PBD-BODIPY (10 μM) at 37 °C were initiated by AIBN (6 mM) in chlorobenzene. A 3.5 mL quartz cuvette was charged with 1,4-dioxane (0.620 mL) and either PhCl (1.790 mL) or PhCl and DMSO (1.180 mL and 0.620 mL, respectively). The proceeding steps are the same as those described above but where only AIBN (50 μL of a 300 mM stock solution in chlorobenzene) is used to initiate the co-autoxidation which was monitored at 588 nm (*ε* = 123 000 M^–1^ cm^–1^ in PhCl, 118 200 M^–1^ cm^–1^ in 2 : 1 PhCl/DMSO). The rate of initiation in PhCl (*R*_i_ = 2.40 × 10^–9^ M s^–1^) and in 2 : 1 PhCl/DMSO were previously standardized by PMC. The second order rate constants for propagation are *k*_PBD-BODIPY_ = 5310 M^–1^ s^–1^ (PhCl) and 5900 M^–1^ s^–1^ (2 : 1 PhCl/DMSO).^15^

#### Determination of solvent kinetic isotope effects (SKIEs) in inhibited co-autoxidation experiments

Autoxidations of 1,4-dioxane were conducted as described above but where the 3.5 mL quartz cuvette was charged with 1,4-dioxane (0.620 mL), PhCl (1.770 mL), and 0.025 mL of MeOL (L = H or D). The experiments containing MeOL were conducted in competition and each set was determined in triplicate.

#### Determination of QMD C–C bond dissociation enthalpies (Δ*H*)^24^

To a 3.5 mL quartz cuvette was added 2.475 mL of 1,2-dichlorobenzene and 0.025 mL of a 5 mM stock of QMD in 1,2-dichorobenzene. The cuvette was affixed with a rubber septum and the contents were purged with N_2_ for 10 min. A scan on the UV-vis spectrophotometer was acquired using a cuvette containing only solvent and used to record baseline-corrected spectra throughout the experiment. The cuvettes containing the QMDs equilibrated to the set temperature for at least 5 min before recording the spectra. The measurements were conducted in triplicate.

### EPR spectra

Electron paramagnetic resonance (EPR) spectra were recorded using a Bruker EMXplus (X-band) spectrometer equipped with an ER 4119HS cavity at 20 °C. The samples were 0.1–10 mM in benzene and degassed (3 cycles of freeze–pump–thaw) and placed under an atmosphere of N_2_ prior to acquisition. The radical concentration was determined using the quantitative EPR package of the Bruker Xenon software. Spectral simulations were performed using EasySpin.^43^

### Calculations

Calculations were conducted using the B3LYP method^44^ (CBSB7 basis set) and CBS-QB3 (complete basis set) method^45^ as implemented in the Gaussian 16 suite of programs.^46^ Rate constants were calculated using transition state theory at 37 °C. Dispersion corrections were applied using Grimme's D3 approach.^47^ Hyperfine coupling constants were predicted by spin density distributions at the B3LYP/TZVP level of theory.^48^

## Conflicts of interest

There are no conflicts to declare.

## Supplementary Material

Supplementary information
