# Supplementary material for: Quinone methide dimers lacking labile hydrogen atoms are surprisingly excellent radical-trapping antioxidants
Source: Chem Sci. 2020 May 6;11(22):5676–89. doi: 10.1039/d0sc02020f (PMC7422964; doi:10.1039/d0sc02020f)
Supplement: Supplementary file 1 [file SC-011-D0SC02020F-s001.pdf]

Electronic Supplementary Material (ESI) for Chemical Science.
This journal is © The Royal Society of Chemistry 2020

Supporting Information for

Quinone Methide Dimers Lacking Labile Hydrogen Atoms Are Surprisingly Excellent Radical-Trapping Antioxidants

Mark A. R. Raycroft, Jean-Philippe R. Chauvin, Matthew S. Galliher, Kevin J. Romero,
Corey R. J. Stephenson* and Derek A. Pratt*

Table of contents

1. Co-autoxidation kinetic experiments in hexadecene/PhCl S2
2. Co-autoxidation kinetic experiments in dioxane/PhCl S14
3. Co-autoxidation kinetic experiments in cumene/PhCl S15
4. Scanning kinetics experiments in hexadecene/PhCl and cumene/PhCl S18
5. Computed frontier molecular orbitals of **4a** and **2d** S25
6. Experimental and computed UV-vis spectra of **4a** and **2d** S26
7. Computed UV-vis spectra of **2d**- and **6**-derived radicals S27
8. Spectral characteristics and thermal stability of **6** S28
9. Cross-over experiment of **2d** with **6** S29
10. EPR spectral data of **2d** and **6** S30
11. Hammett plots for **1** and **3** at 37, 70, 100 °C S31
12. QMD concentration dependences of inhibition rate, time of inhibition, or stoichiometry S32
13. UV-vis spectra and corresponding Van't Hoff plots for **2** S35
14. Comparison of experimental BDEs to DFT and dispersion-corrected DFT values S38
15. Extinction coefficient determinations for **2d**, **4a**, and **5b** S39
16. Characterization data for synthesized compounds S43
17. Computational studies S59
18. Computed structures of **1d**, **5a**, **3d** S64
19. Cartesian coordinates S65
20. References and notes S200

Co-autoxidation kinetic experiments in hexadecene/PhCl

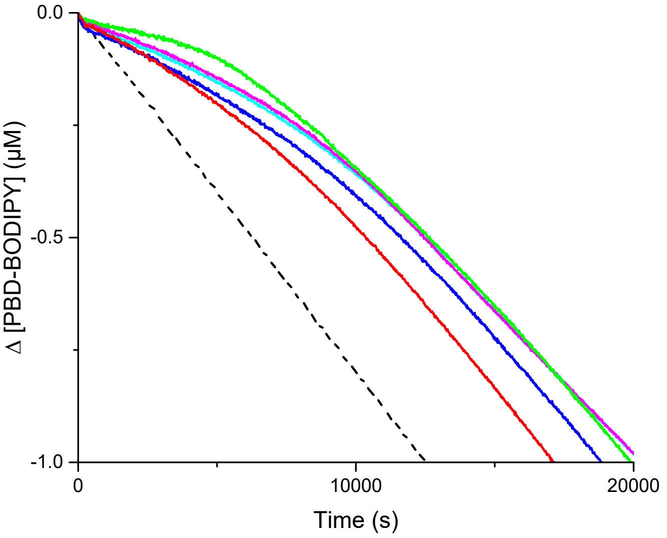

Figure S1. Co-autoxidations of 1-hexadecene (2.9 M) and PBD-BODIPY (10 μM) initiated by AIBN (6 mM) in chlorobenzene at 37 °C (dashed black trace) and inhibited by 5 μM **1a** (magenta), **1b** (green), **1c** (cyan), **1d** (red), **1e** (blue).

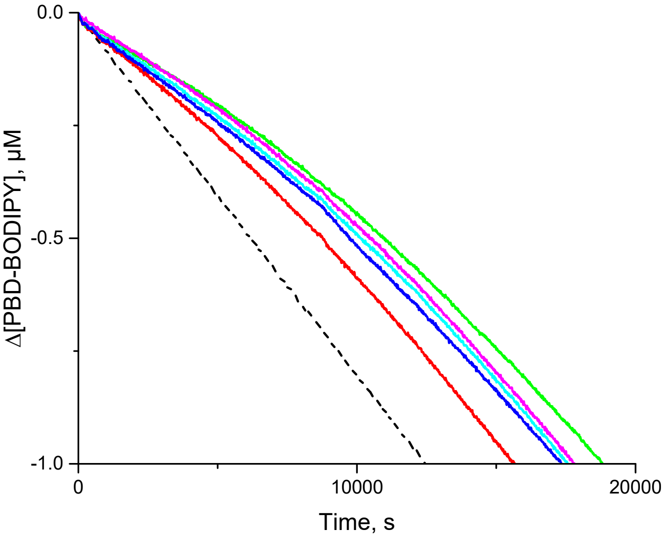

Figure S2. Co-autoxidations of 1-hexadecene (2.9 M) and PBD-BODIPY (10 μM) initiated by AIBN (6 mM) in chlorobenzene at 37 °C (dashed black trace) and inhibited by 5 μM **1f** (magenta), **1g** (green), **1h** (cyan), **1i** (blue), **1j** (red).

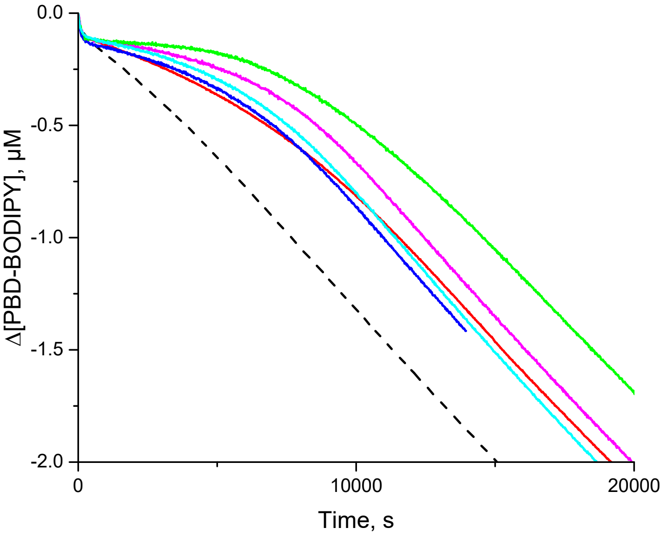

Figure S3. Co-autoxidations of 1-hexadecene (2.9 M) and PBD-BODIPY (10 μM) initiated by $^{t}BuOO^{t}Bu$ (87 mM) in chlorobenzene at 70 °C (dashed black trace) and inhibited by 5 μM **1a** (magenta), **1b** (green), **1c** (red), **1d** (blue), **1e** (cyan).

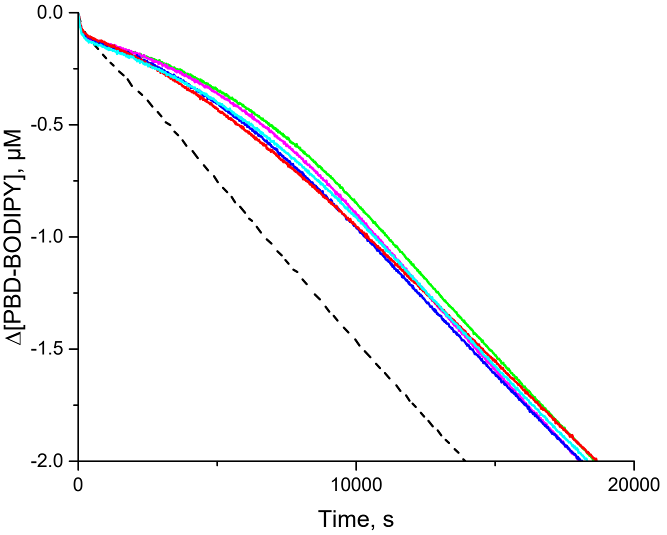

Figure S4. Co-autoxidations of 1-hexadecene (2.9 M) and PBD-BODIPY (10 μM) initiated by $^{t}BuOO^{t}Bu$ (87 mM) in chlorobenzene at 70 °C (dashed black trace) and inhibited by 5 μM **1f** (magenta), **1g** (green), **1h** (blue), **1i** (cyan), **1j** (red).

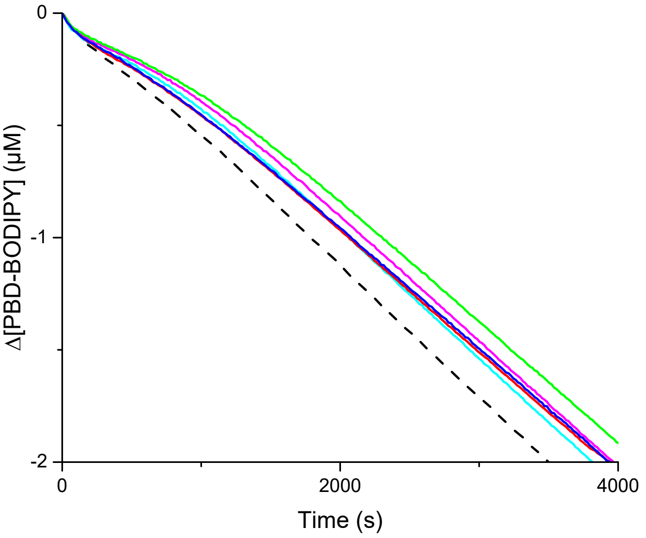

Figure S5. Co-autoxidations of 1-hexadecene (2.9 M) and PBD-BODIPY (10 μM) initiated by dicumyl peroxide (1 mM) in chlorobenzene at 100 °C (dashed black trace) and inhibited by 5 μM **1a** (magenta), **1c** (cyan), **1e** (red), **1e** (green), **1g** (blue).

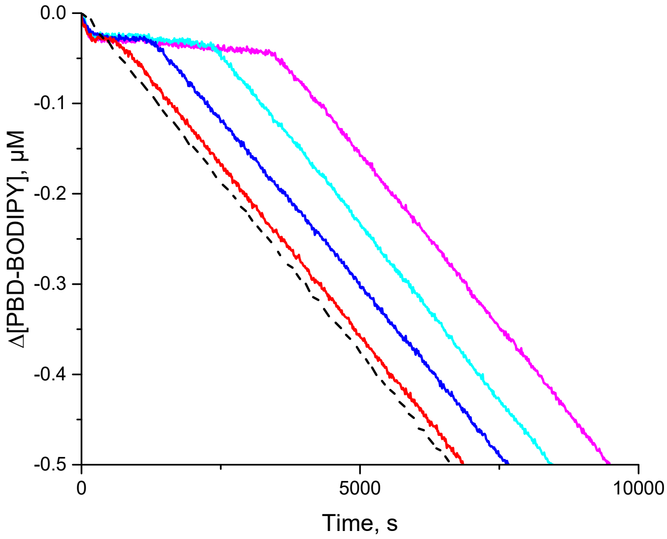

Figure S6. Co-autoxidations of 1-hexadecene (2.9 M) and PBD-BODIPY (10 μM) initiated by AIBN (6 mM) in chlorobenzene at 37 °C (dashed black trace) and inhibited by 0.5 (red), 1 (blue), 2 (cyan), 3 (magenta) μM **2a**.

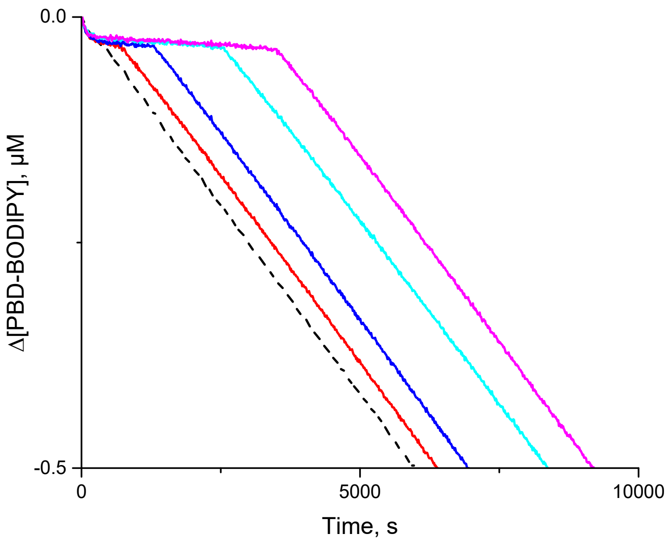

Figure S7. Co-autoxidations of 1-hexadecene (2.9 M) and PBD-BODIPY (10 μM) initiated by AIBN (6 mM) in chlorobenzene at 37 °C (dashed black trace) and inhibited by 0.5 (red), 1 (blue), 2 (cyan), 3 (magenta) μM **2d**.

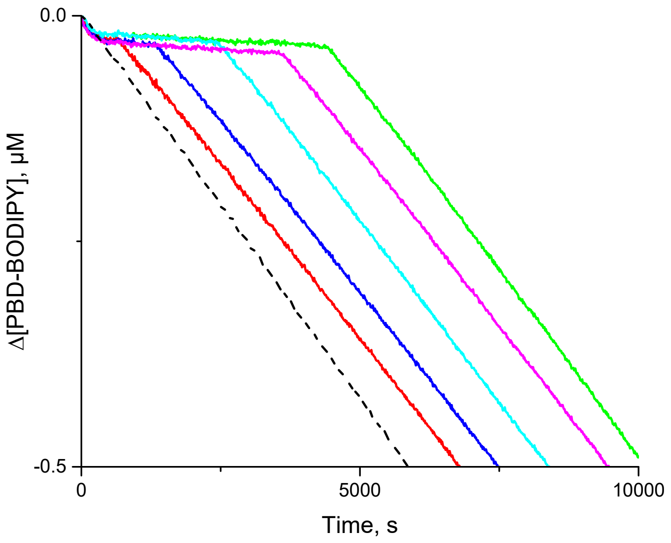

Figure S8. Co-autoxidations of 1-hexadecene (2.9 M) and PBD-BODIPY (10 μM) initiated by AIBN (6 mM) in chlorobenzene at 37 °C (dashed black trace) and inhibited by 0.5 (red), 1 (blue), 2 (cyan), 3 (magenta), 4 (green) μM **2g**.

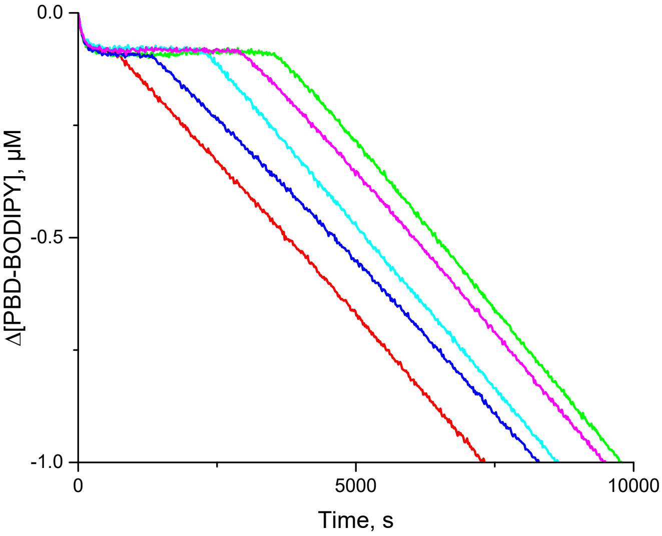

Figure S9. Co-autoxidations of 1-hexadecene (2.9 M) and PBD-BODIPY (10 μM) initiated by AIBN (6 mM) in chlorobenzene at 70 °C (dashed black trace) and inhibited by 0.5 (red), 1 (blue), 2 (cyan), 3 (magenta), 4 (green) μM **2d**.

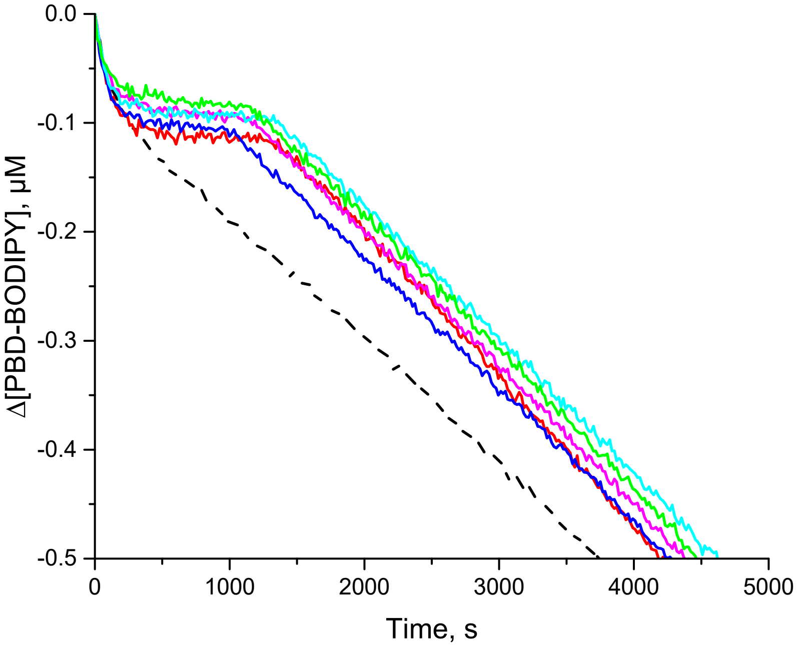

Figure S10. Co-autoxidations of 1-hexadecene (2.9 M) and PBD-BODIPY (10 μM) initiated by $^{t}BuOO^{t}Bu$ (87 mM) in chlorobenzene at 70 °C (dashed black trace) and inhibited by 1 μM **2a** (green), **2b** (magenta), **2c** (blue), **2d** (cyan), **2e** (red).

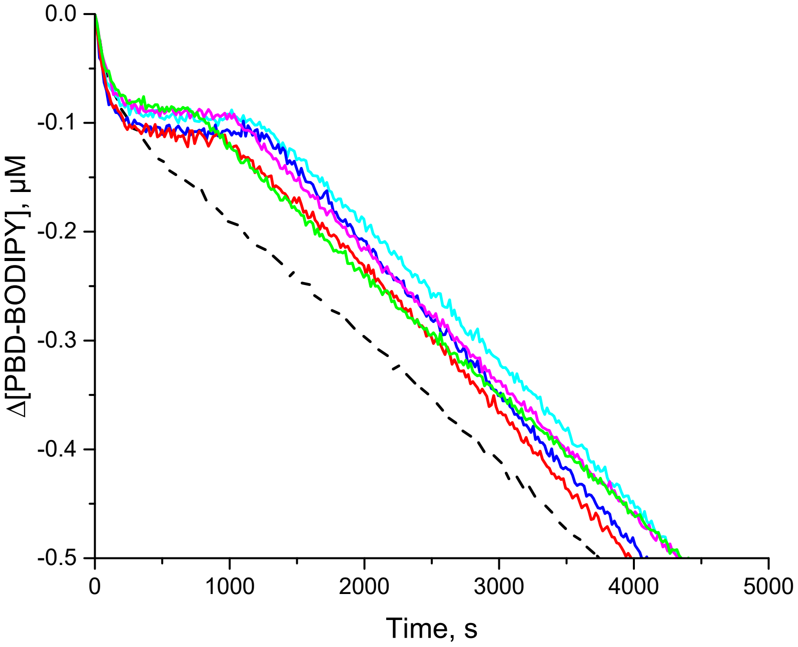

Figure S11. Co-autoxidations of 1-hexadecene (2.9 M) and PBD-BODIPY (10 μM) initiated by $^tBuOO^tBu$ (87 mM) in chlorobenzene at 70 °C (dashed black trace) and inhibited by 1 μM **2f** (blue), **2g** (cyan), **2h** (magenta), **2i** (red), **2j** (green).

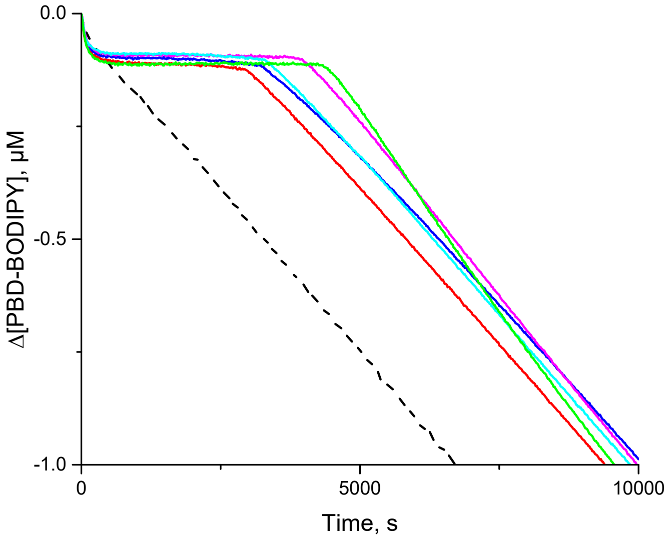

Figure S12. Co-autoxidations of 1-hexadecene (2.9 M) and PBD-BODIPY (10 μM) initiated by $^tBuOO^tBu$ (87 mM) in chlorobenzene at 70 °C (dashed black trace) and inhibited by 5 μM **2a** (red), **2b** (blue), **2c** (cyan), **2d** (magenta), **2e** (green).

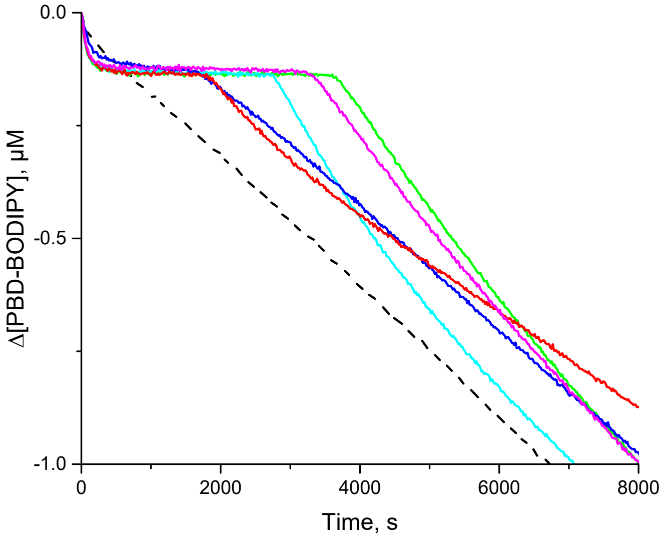

Figure S13. Co-autoxidations of 1-hexadecene (2.9 M) and PBD-BODIPY (10 μM) initiated by $^{t}BuOO^{t}Bu$ (87 mM) in chlorobenzene at 70 °C (dashed black trace) and inhibited by 5 μM **2f** (magenta), **2g** (green), **2h** (cyan), **2i** (blue), **2j** (red).

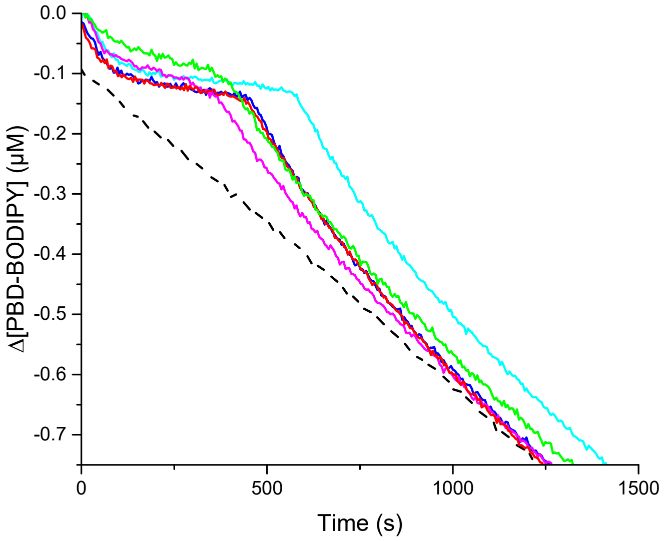

Figure S14. Co-autoxidations of 1-hexadecene (2.9 M) and PBD-BODIPY (10 μM) initiated by dicumyl peroxide (1 mM) in chlorobenzene at 100 °C (dashed black trace) and inhibited by 5 μM **2a** (green), **2c** (magenta), **2d** (blue), **2e** (cyan), **2g** (red).

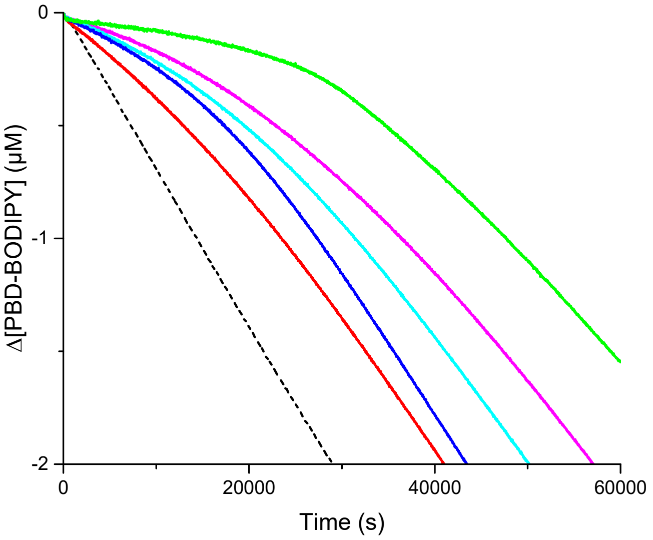

Figure S15. Co-autoxidations of 1-hexadecene (2.9 M) and PBD-BODIPY (10 µM) initiated by AIBN (6 mM) in chlorobenzene at 37 °C (dashed black trace) and inhibited by 5 µM **3a** (magenta), **3b** (green), **3c** (red), **3d** (cyan), **3e** (blue).

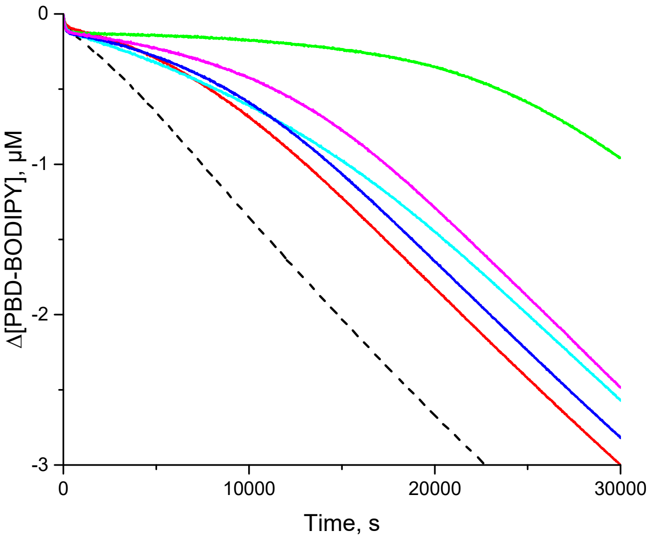

Figure S16. Co-autoxidations of 1-hexadecene (2.9 M) and PBD-BODIPY (10 µM) initiated by $^tBuOO^tBu$ (87 mM) in chlorobenzene at 70 °C (dashed black trace) and inhibited by 5 µM **3a** (magenta), **3b** (green), **3c** (red), **3d** (cyan), **3e** (blue).

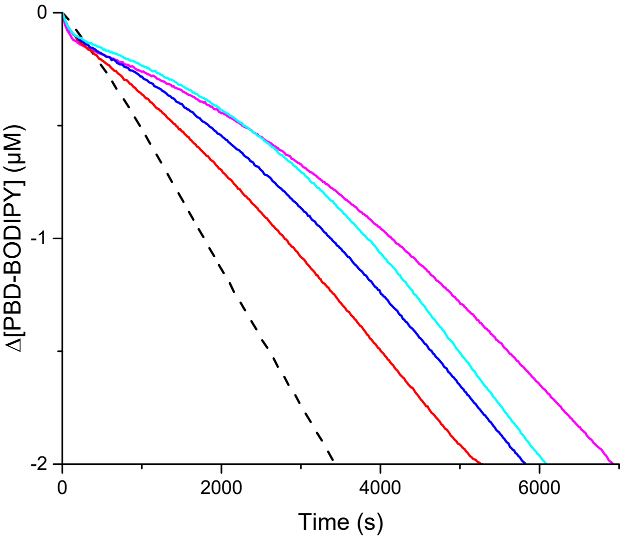

Figure S17. Co-autoxidations of 1-hexadecene (2.9 M) and PBD-BODIPY (10 μM) initiated by dicumyl peroxide (1 mM) in chlorobenzene at 100 °C (dashed black trace) and inhibited by 5 μM **3a** (magenta), **3c** (red), **3d** (blue), **3e** (cyan).

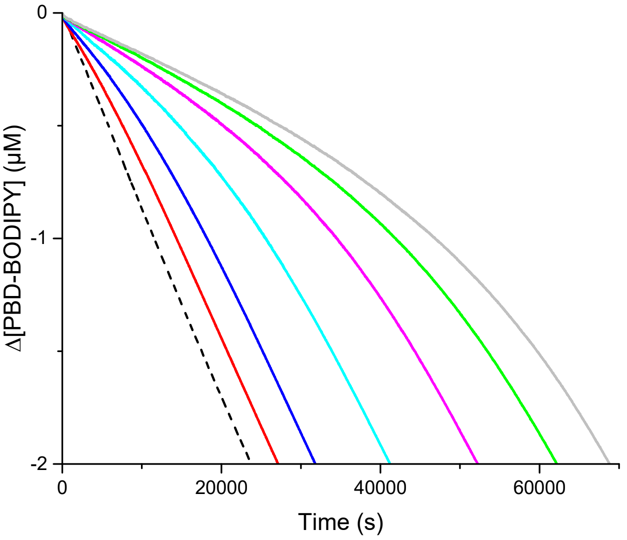

Figure S18. Co-autoxidations of 1-hexadecene (2.9 M) and PBD-BODIPY (10 μM) initiated by AIBN (6 mM) in chlorobenzene at 37 °C (dashed black trace) and inhibited by 5 (red), 10 (blue), 20 (cyan), 30 (magenta), 40 (green), 50 (grey) μM **4a**.

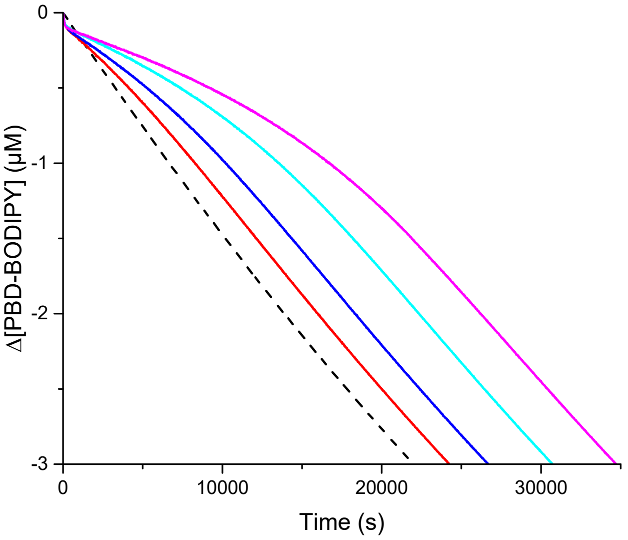

Figure S19. Co-autoxidations of 1-hexadecene (2.9 M) and PBD-BODIPY (10 μM) initiated by $^tBuOO^tBu$ (87 mM) in chlorobenzene at 70 °C (dashed black trace) and inhibited by 5 (red), 10 (blue), 20 (cyan), 30 (magenta) μM **4a**.

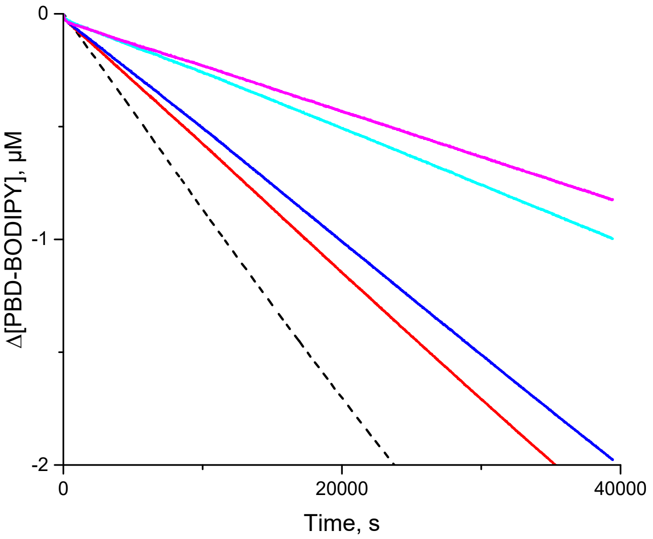

Figure S20. Co-autoxidations of 1-hexadecene (2.9 M) and PBD-BODIPY (10 μM) initiated by AIBN (6 mM) in chlorobenzene at 37 °C (dashed black trace) and inhibited by 50 (red), 100 (blue), 500 (cyan), 1000 (magenta) μM **4b**.

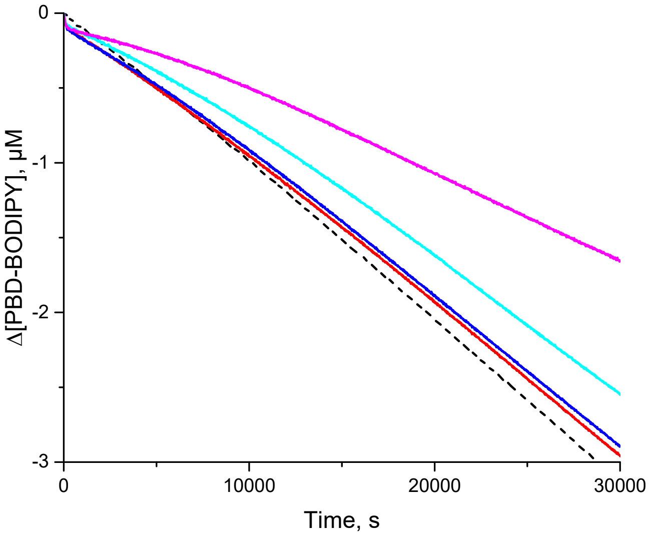

Figure S21. Co-autoxidations of 1-hexadecene (2.9 M) and PBD-BODIPY (10 µM) initiated by $^{t}BuOO^{t}Bu$ (87 mM) in chlorobenzene at 70 °C (dashed black trace) and inhibited by 10 (red), 50 (blue), 100 (cyan), 500 (magenta) µM **4b**.

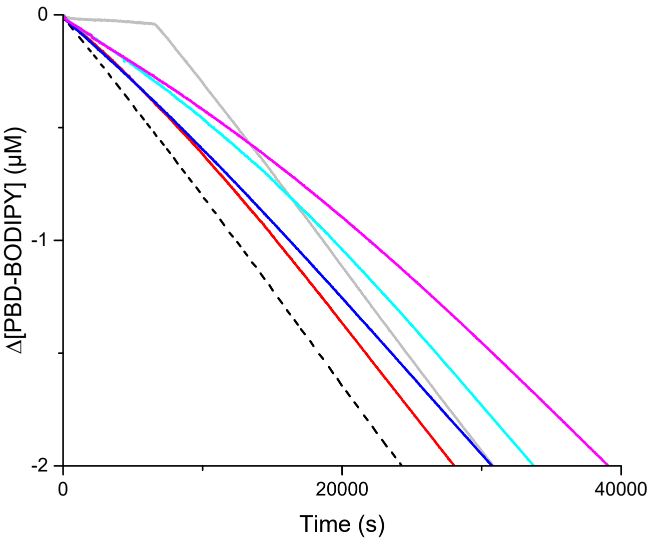

Figure S22. Co-autoxidations of 1-hexadecene (2.9 M) and PBD-BODIPY (10 µM) initiated by AIBN (6 mM) in chlorobenzene at 37 °C (dashed black trace) and inhibited by **5a** at 5 µM (red) or 10 µM (cyan), **5b** at 5 µM (blue or 10 µM (magenta), **2e** at 5 µM (grey).

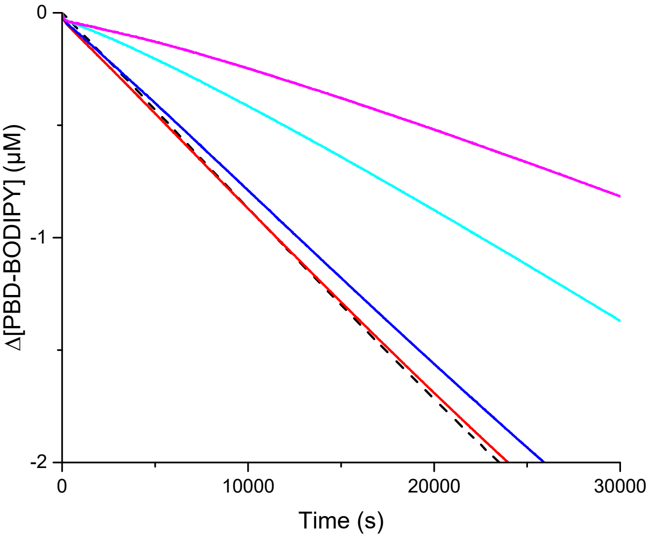

Figure S23. Co-autoxidations of 1-hexadecene (2.9 M) and PBD-BODIPY (10 μM) initiated by AIBN (6 mM) in chlorobenzene at 37 °C (dashed black trace) and inhibited by 5 (red), 10 (blue), 50 (cyan), 100 (magenta) μM **6**.

Table S1. Inhibition rate constants for **2d** and **2d-*d₁₂*** (1 μM) in hexadecene/PhCl initiated by AIBN (6 mM) at 37 °C.

**Inhibitor**	$\boldsymbol{k_{inh}}$	$\boldsymbol{n}$	**DKIE**
**2d**	$(4.4 \pm 0.3) \times 10^6$ $M^{-1}s^{-1}$	1.7 ± 0.1	$k_H/k_D = 1.0 \pm 0.1$
**2d-*d₁₂***	$(4.3 \pm 0.1) \times 10^6$ $M^{-1}s^{-1}$	1.8 ± 0.1	

Co-autoxidation kinetic experiments in dioxane/PhCl

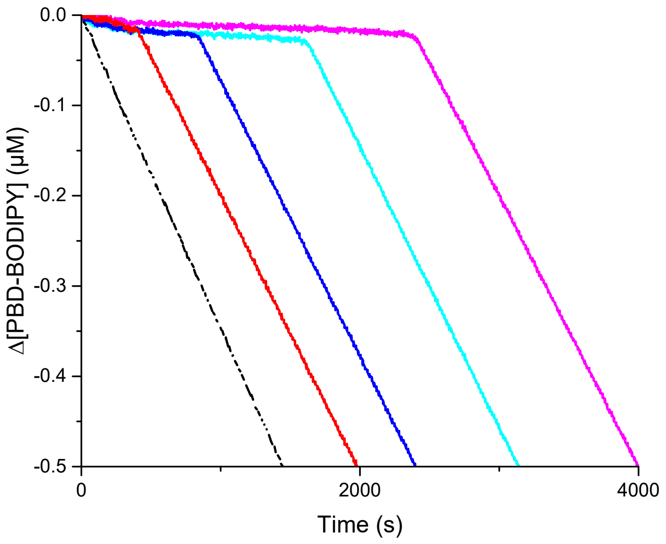

Figure S24. Co-autoxidations of 1,4-dioxane (2.9 M) and PBD-BODIPY (10 µM) initiated by AIBN (6 mM) in chlorobenzene at 37 °C (dashed black trace) and inhibited by 0.5 (red), 1 (blue), 2 (cyan), 3 (magenta) µM **2d**.

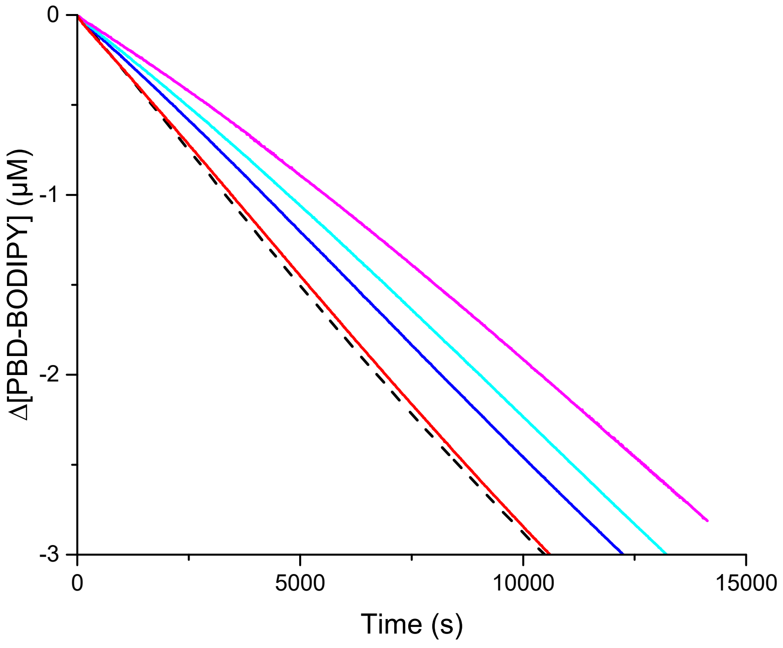

Figure S25. Co-autoxidations of 1,4-dioxane (2.9 M) and PBD-BODIPY (10 µM) initiated by AIBN (6 mM) in chlorobenzene at 37 °C (dashed black trace) and inhibited by 5 (red), 10 (blue), 20 (cyan), 30 (magenta) µM **4a**.

Co-autoxidation kinetic experiments in cumene/PhCl

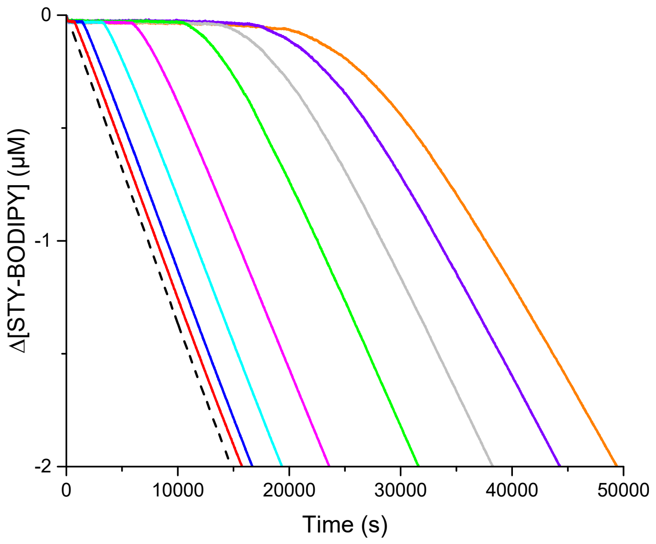

Figure S26. Co-autoxidations of cumene (3.6 M) and STY-BODIPY (10 µM) initiated by AIBN (6 mM) in chlorobenzene at 37 °C (dashed black trace) and inhibited by 1 (red), 2 (blue), 5 (cyan), 10 (magenta), 20 (green), 30 (grey), 40 (violet), 50 (orange) µM **2d**.

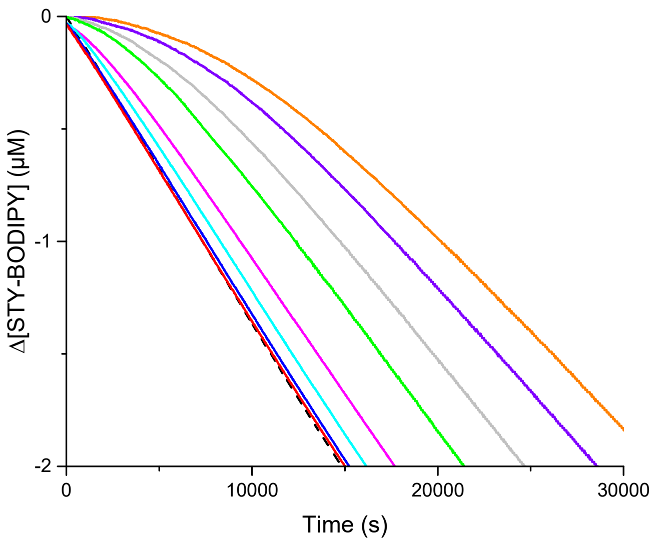

Figure S27. Co-autoxidations of cumene (3.6 M) and STY-BODIPY (10 µM) initiated by AIBN (6 mM) in chlorobenzene at 37 °C (dashed black trace) and inhibited by the intermediate QM formed subsequent to substitution of the QMD 1 (red), 2 (blue), 5 (cyan), 10 (magenta), 20 (green), 30 (grey), 40 (violet), 50 (orange) µM **2d**. The data are transposed to the uninhibited trace.

Table S2. Second order rate constants (k_{inh}) and stoichiometries (n) for the second phase of inhibition by **2d** in the co-autoxidations of cumene (3.6 M) and STY-BODIPY (10 µM) initiated by AIBN (6 mM) in chlorobenzene at 37 °C.

AO	[AO], µM	k_{inh}, $M^{-1}\cdot s^{-1}$ (37 °C)	n (37 °C)	k_{inh}^{avg}, $M^{-1}\cdot s^{-1}$	n
**2d**	20	$(1.0 \pm 0.1) \times 10^4$	0.53 ± 0.05	$(8 \pm 1) \times 10^3$	0.50 ± 0.02
	30	$(8.3 \pm 0.9) \times 10^3$	0.49 ± 0.02		
	40	$(6.5 \pm 0.2) \times 10^3$	0.51 ± 0.01		
	50	$(7.0 \pm 0.9) \times 10^3$	0.48 ± 0.04		

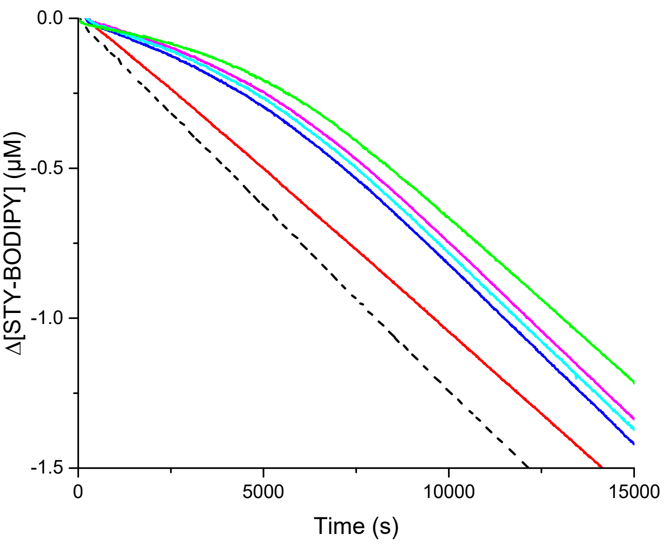

Figure S28. Co-autoxidations of cumene (3.6 M) and STY-BODIPY (10 µM) initiated by AIBN (6 mM) in chlorobenzene at 37 °C (dashed black trace) and inhibited by 5 µM **4a** (cyan), 5 µM **4a-H$_2$O** (blue), 5 µM **4b** (red), 5 µM **5a** (magenta), 5 µM **5b** (green).

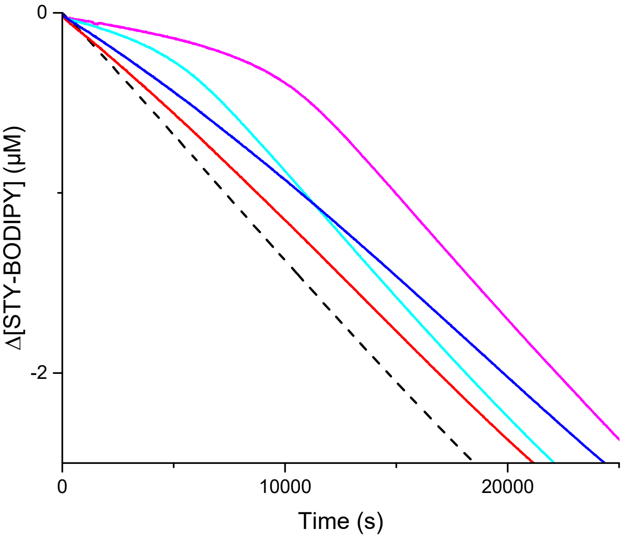

Figure S29. Co-autoxidations of cumene (3.6 M) and STY-BODIPY (10 µM) initiated by AIBN (6 mM) in chlorobenzene at 37 °C (dashed black trace) and inhibited by 5 µM (cyan) or 10 µM (magenta) **4a**, 5 µM (red) or 10 µM (blue) **6**.

Table S3. Second order rate constants (k_{inh}) and stoichiometries (*n*) for **6**-inhibited co-autoxidations of cumene (3.6 M) and STY-BODIPY (10 µM) initiated by AIBN (6 mM) in chlorobenzene at 37 °C.

AO	[AO], µM	Cumene, 37 °C	
		k_{inh}, $M^{-1}\cdot s^{-1}$	n
diMe-QMD	5	(2.8 ± 0.1) × 10^3	2.1 ± 0.1
	10	(2.2 ± 0.2) × 10^3	1.7 ± 0.1
QM-Me	5	(7.2 ± 0.3) × 10^3	2.2 ± 0.1
	10	(6.7 ± 0.3) × 10^3	2.0 ± 0.2

Scanning kinetics experiments in hexadecene/PhCl and cumene/PhCl

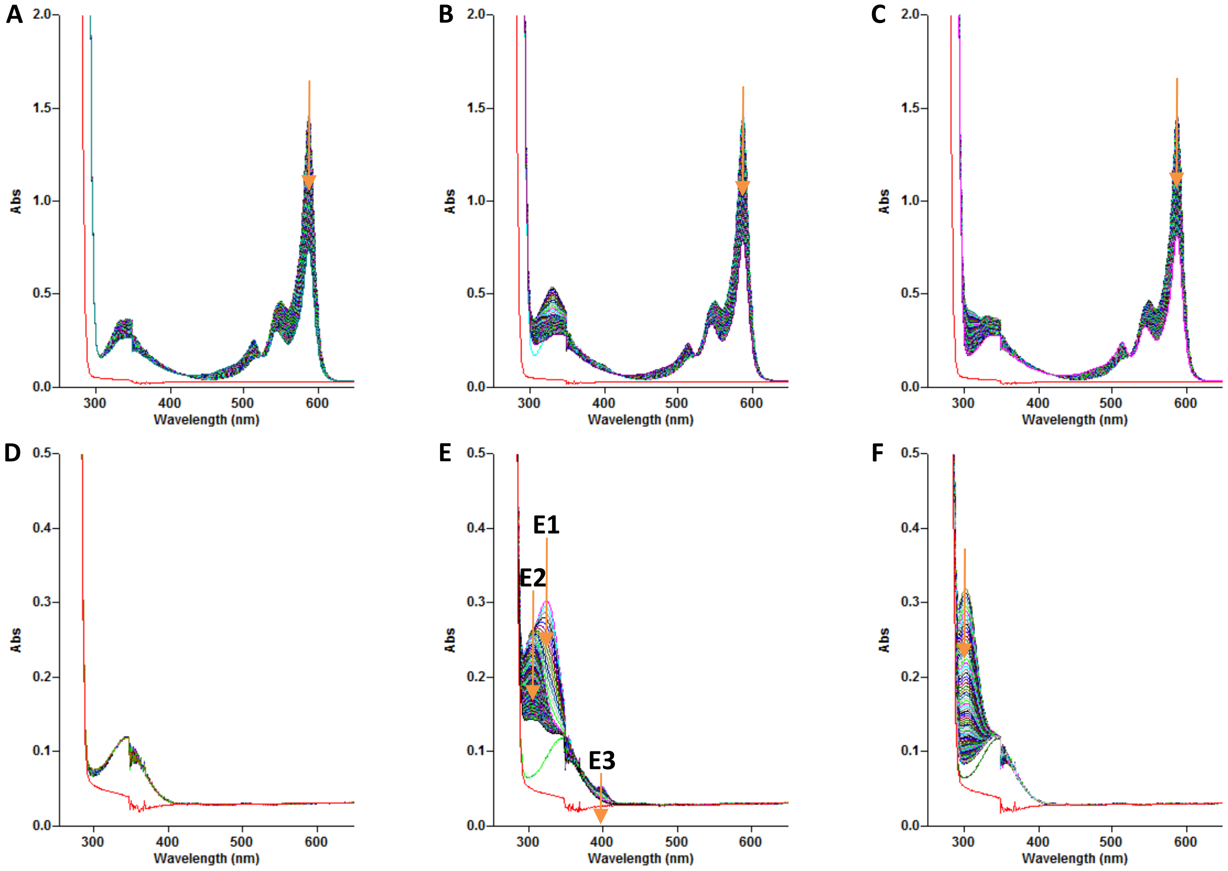

Figure S30. Co-autoxidations of 1-hexadecene (2.9 M) and PBD-BODIPY (10 µM) initiated by AIBN (6 mM) in chlorobenzene at 37 °C (A) inhibited by 5 µM **2d** (B) or 10 µM **4a** (C). Autoxidations of hexadecene (2.9 M) initiated by AIBN (6 mM) in chlorobenzene at 37 °C (D) and inhibited by 5 µM **2d** (E) or 10 µM **4a** (F).

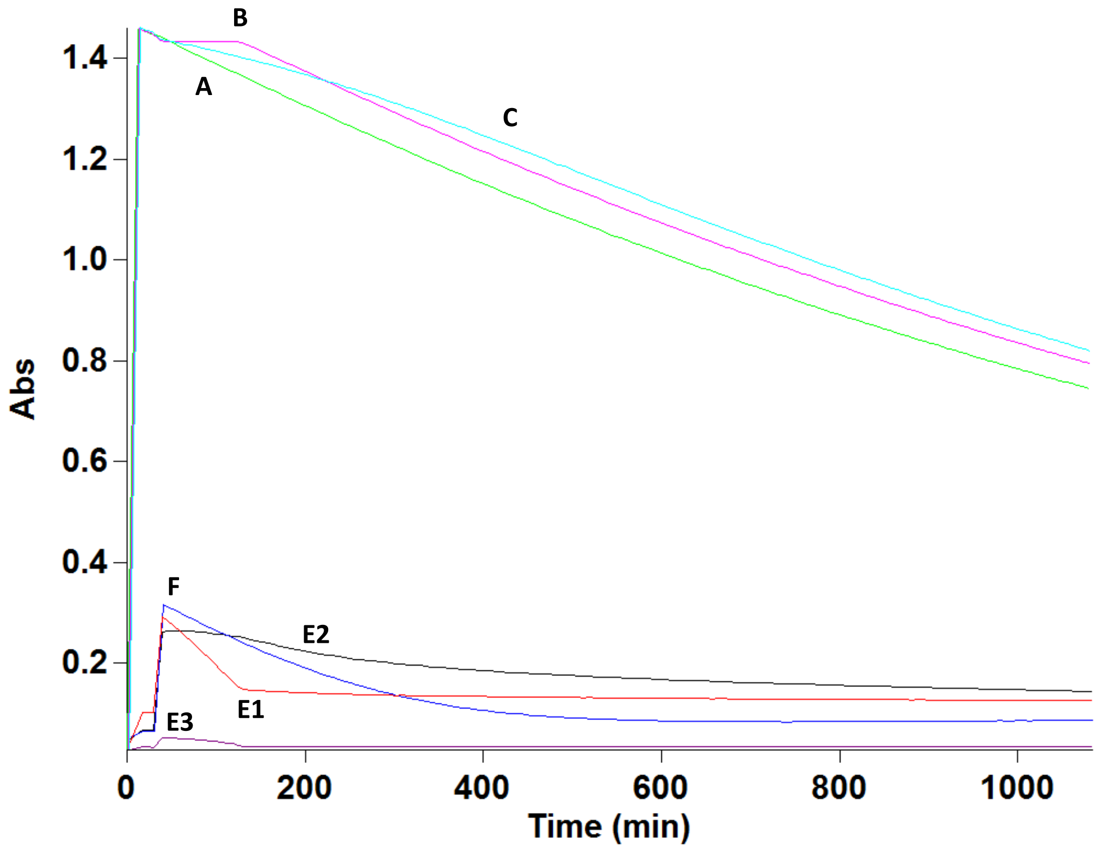

Figure S31. Abs vs time traces derived from the spectra in Figure S30. The co-autoxidations are monitored at 588 nm (uninhibited, green; 5 µM **2d**, magenta; 10 µM **4a**, cyan). The autoxidation inhibited by **2d** is monitored at 330 nm (red, E1), 300 nm (black, E2), 404 nm (violet, E3). The autoxidation inhibited by **4a** is monitored at 305 nm (blue).

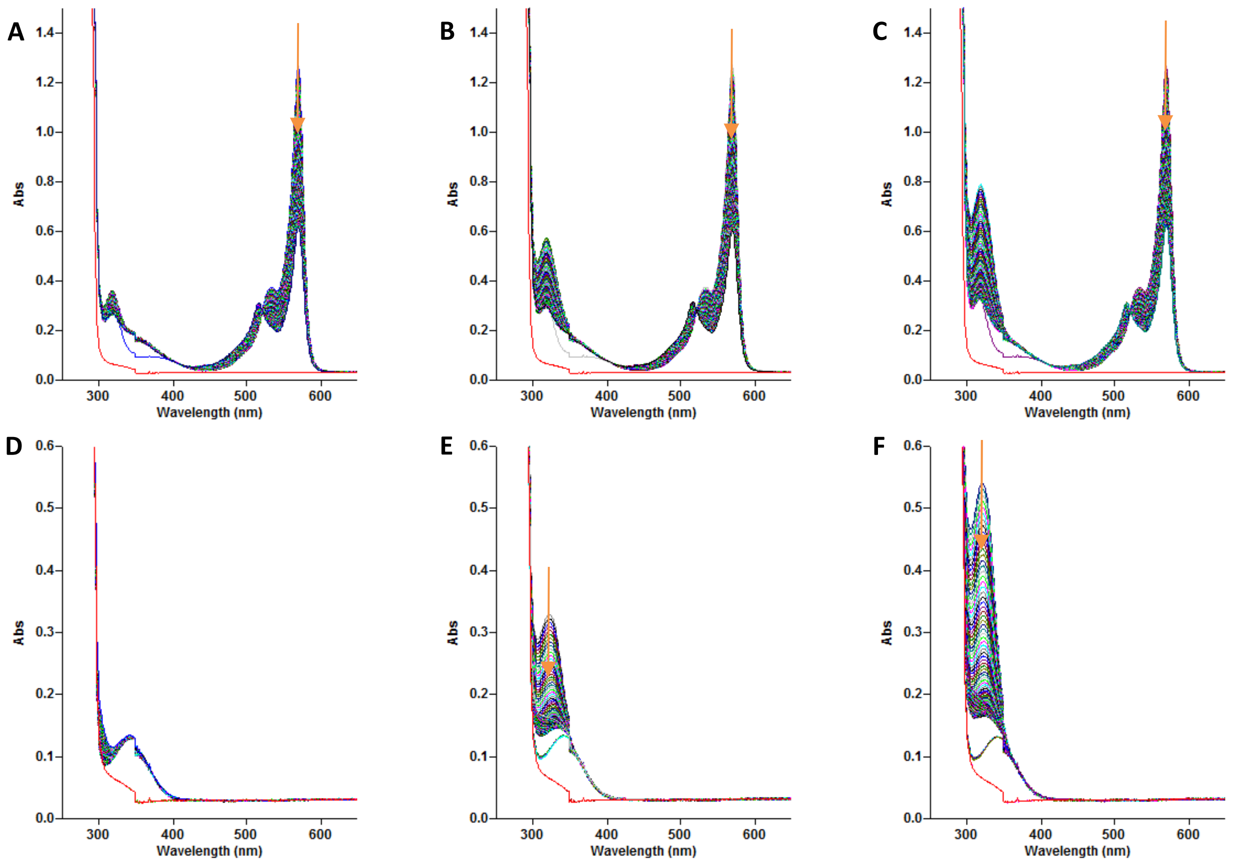

Figure S32. Co-autoxidations of cumene (3.6 M) and STY-BODIPY (10 µM) initiated by AIBN (6 mM) in chlorobenzene at 37 °C (A) inhibited by 5 µM **6** (B) or 10 µM **6** (C). Autoxidations of cumene (3.6 M) initiated by AIBN (6 mM) in chlorobenzene at 37 °C (D) and inhibited by 5 µM **6** (E) or 10 µM **6** (F).

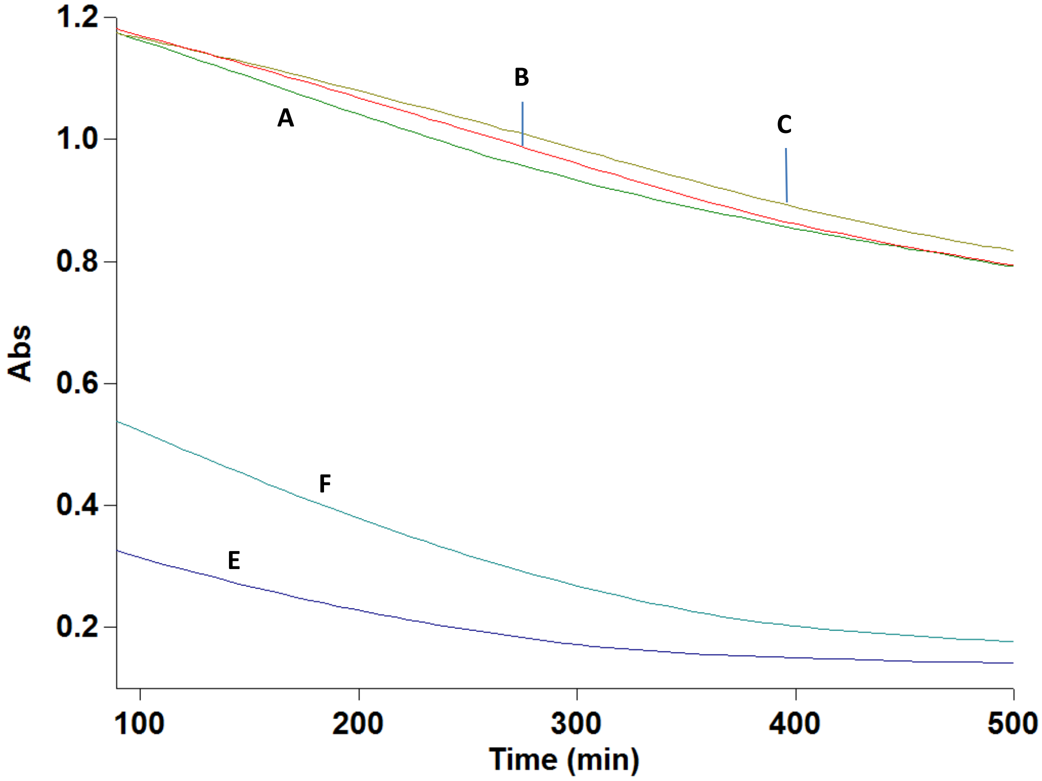

Figure S33. Abs vs time traces derived from the spectra in Figure S32. The co-autoxidations are monitored at 571 nm (uninhibited, green (A); 5 µM **6**, red (B); 10 µM **6,** yellow (C)). The autoxidation inhibited by **6** is monitored at 320 nm (5 µM **6** teal (E), 10 µM **6** blue (F)).

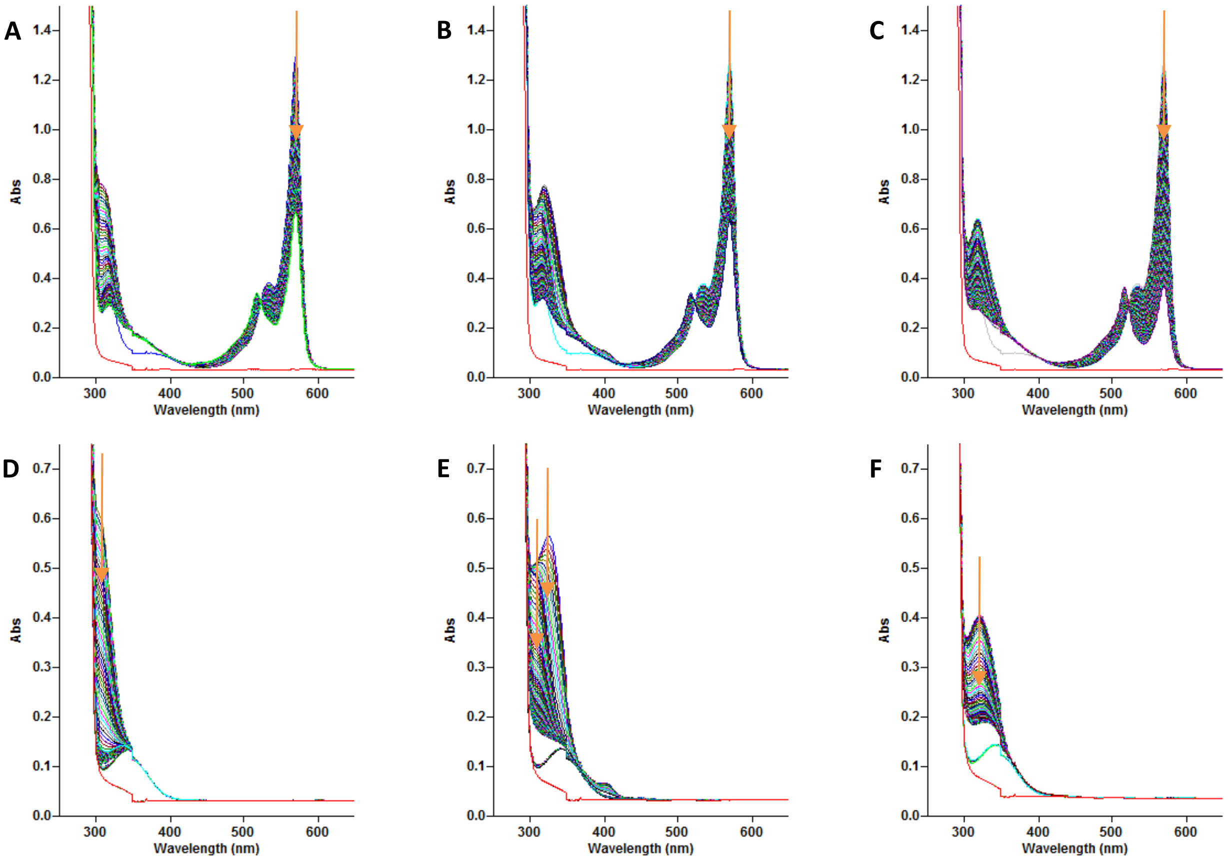

Figure S34. Co-autoxidations of cumene (3.6 M) and STY-BODIPY (10 µM) initiated by AIBN (6 mM) in chlorobenzene at 37 °C inhibited by 20 µM **4a** (A), 10 µM **2d** (C), 10 µM **5b**. Autoxidations of cumene (3.6 M) initiated by AIBN (6 mM) in chlorobenzene at 37 °C (D) and inhibited by 20 µM **4a** µM (D), 10 µM **2d** (E), 10 µM **5b** (F).

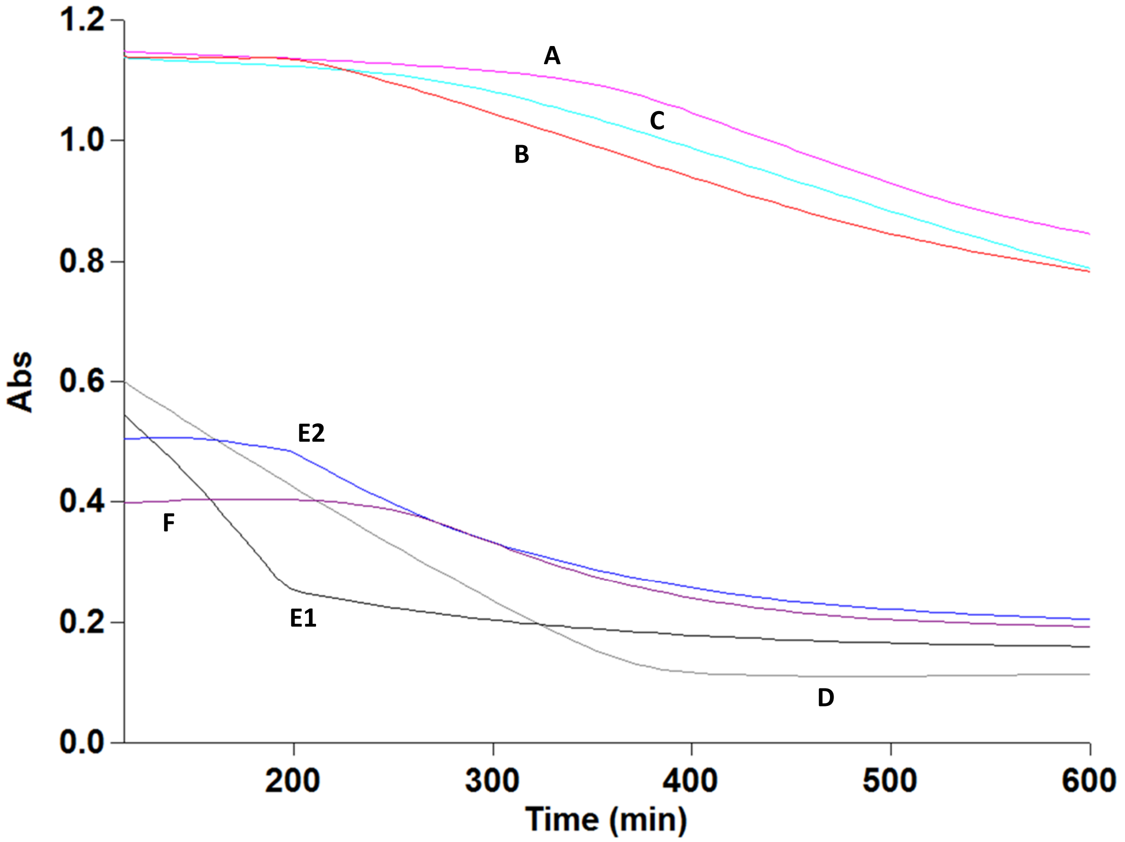

Figure S35. Abs vs time traces derived from the spectra in Figure S34. The co-autoxidations are monitored at 571 nm (20 µM **4a**, magenta; 10 µM **2d**, red; 10 µM **5b**, cyan). The autoxidation inhibited by **4a** is monitored at 305 nm (grey). The autoxidation inhibited by **2d** is monitored at 330 nm (black, E1), 305 nm (blue, E2). The autoxidation inhibited by **5b** is monitored at 320 nm (violet).

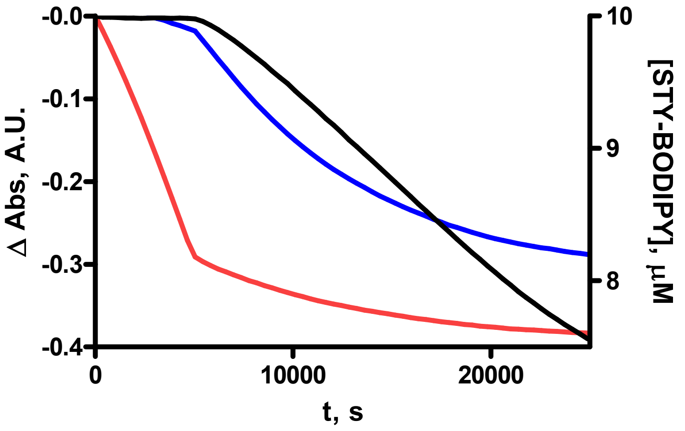

Figure S36. Co-autoxidations of cumene (3.6 M) and STY-BODIPY (10 µM) initiated by AIBN (6 mM) in chlorobenzene at 37 °C inhibited by 10 µM **2d**. The red trace corresponds to the QMD chromophore at 330 nm (left axis); the blue trace corresponds to the QM chromophore at 305 nm (left axis); the black trace corresponds to the STY-BODIPY chromophore at 571 nm (right axis).

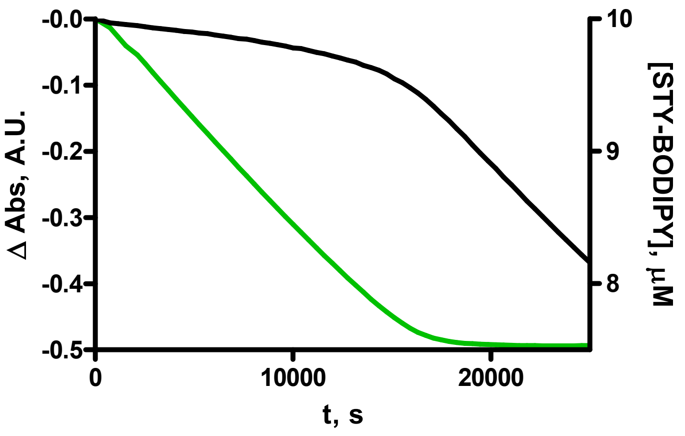

Figure S37. Co-autoxidations of cumene (3.6 M) and STY-BODIPY (10 μM) initiated by AIBN (6 mM) in chlorobenzene at 37 °C inhibited by 20 μM **4a**. The green trace corresponds to the QM chromophore at 305 nm (left axis); the black trace corresponds to the STY-BODIPY chromophore at 571 nm (right axis).

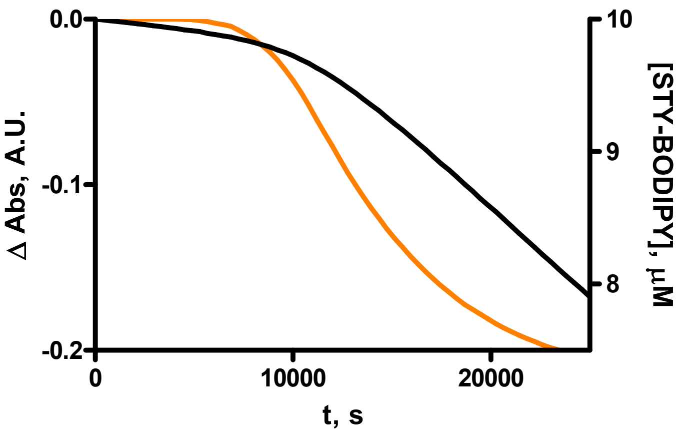

Figure S38. Co-autoxidations of cumene (3.6 M) and STY-BODIPY (10 μM) initiated by AIBN (6 mM) in chlorobenzene at 37 °C inhibited by 10 μM **5b**. The orange trace corresponds to the QM chromophore at 320 nm (left axis); the black trace corresponds to the STY-BODIPY chromophore at 571 nm (right axis).

Computed frontier molecular orbitals of 4a and 2d

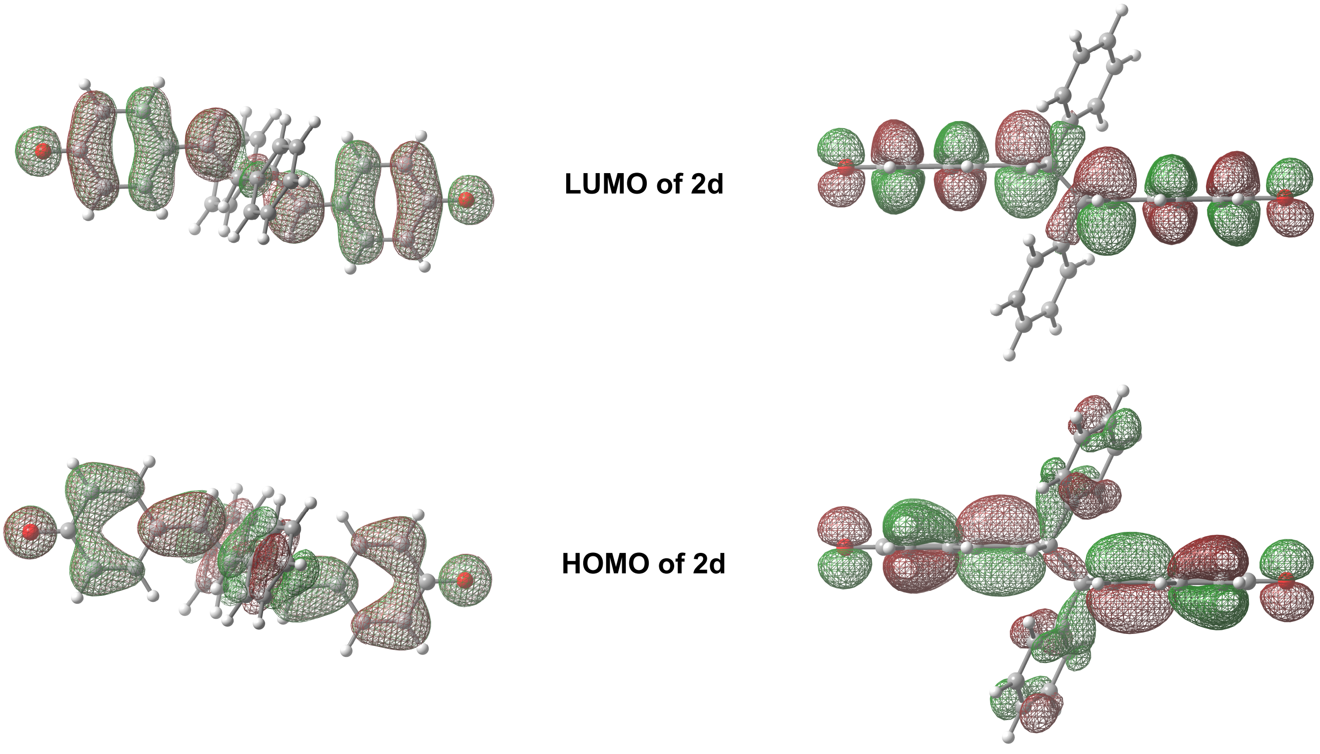

Figure S39. Computed (B3LYP/CBSB7) frontier molecular orbitals for **2d**.

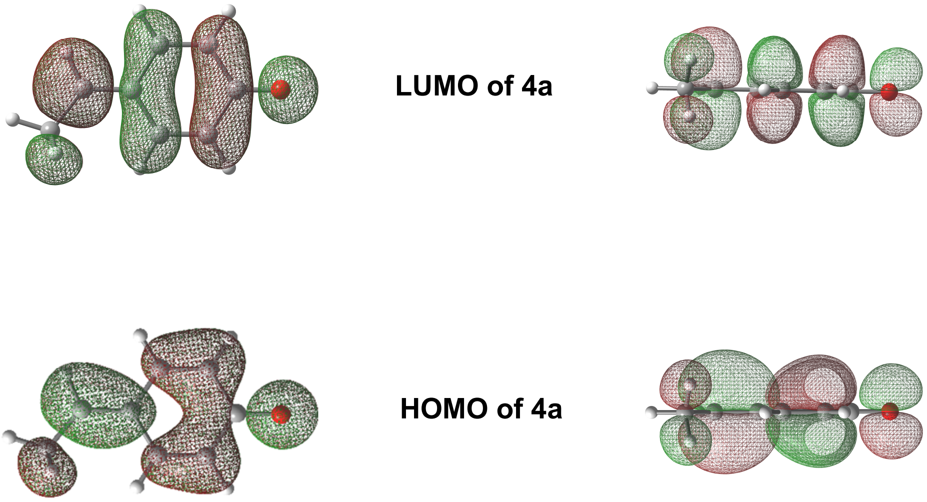

Figure S40. Computed (B3LYP/CBSB7) frontier molecular orbitals for **4a**.

Experimental and computed UV-vis spectra of 4a and 2d

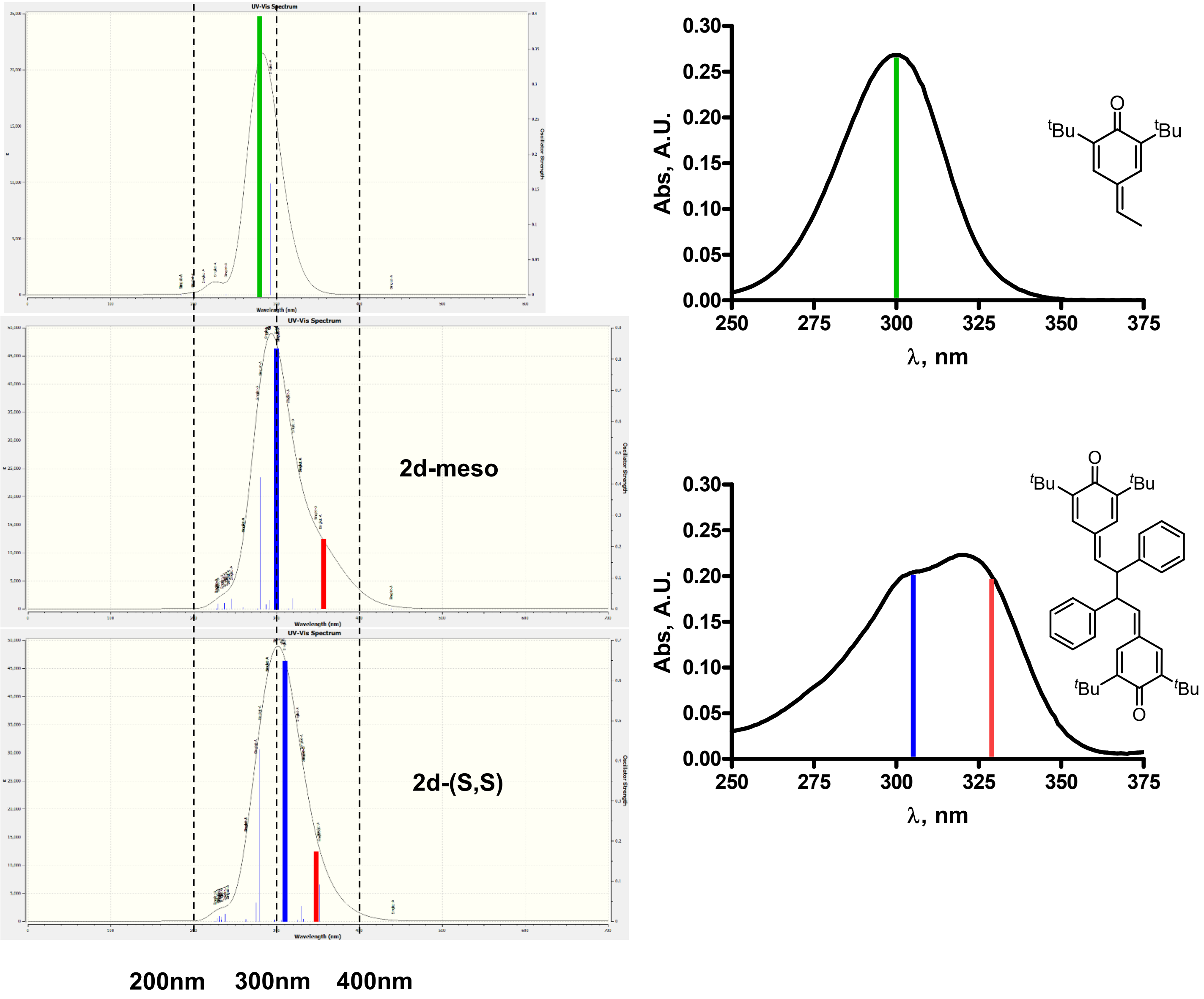

Figure S41. Comparison of computed (B3LYP/CBSB7) UV-visible spectra of **4a**, **2d** (meso, (S,S)) to experimental spectra (hexadecene/PhCl).

Computed UV-vis spectra of 2d- and 6-derived radicals

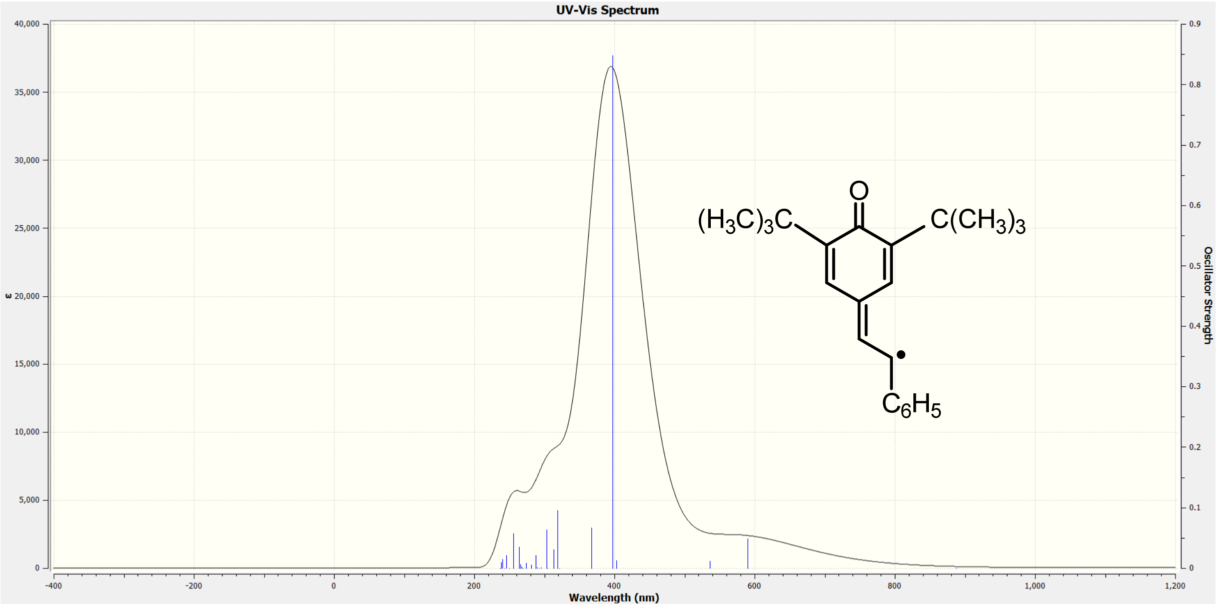

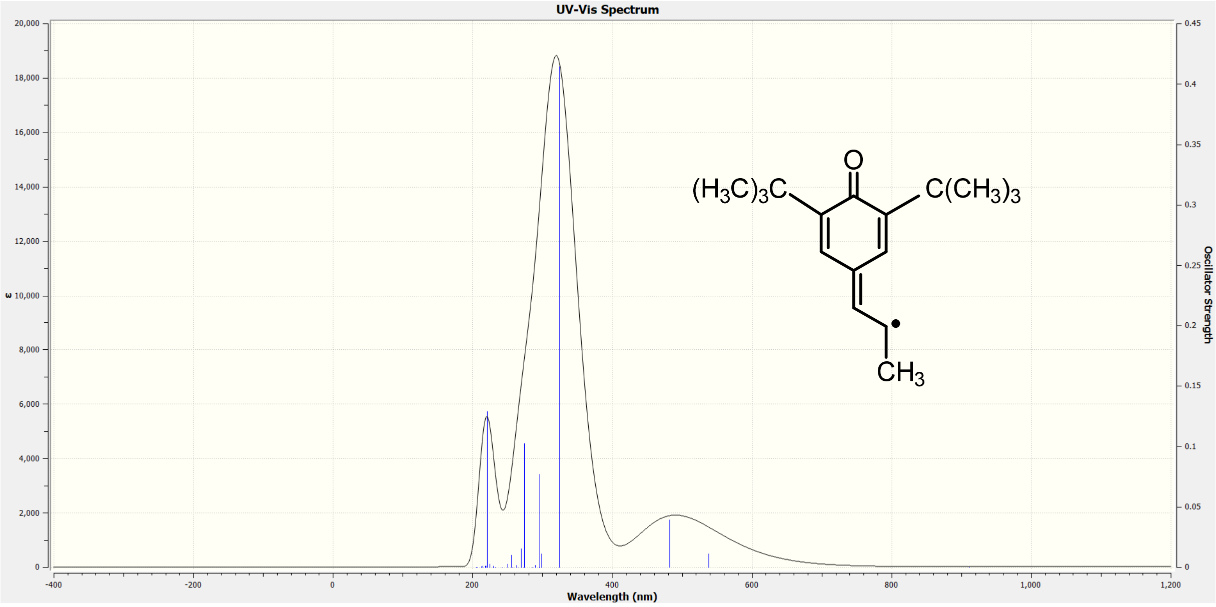

Figure S42. Comparison of computed (B3LYP/CBSB7) UV-visible spectra of **2d**- and **6**-derived radicals.

Spectral characteristics and thermal stability of 6

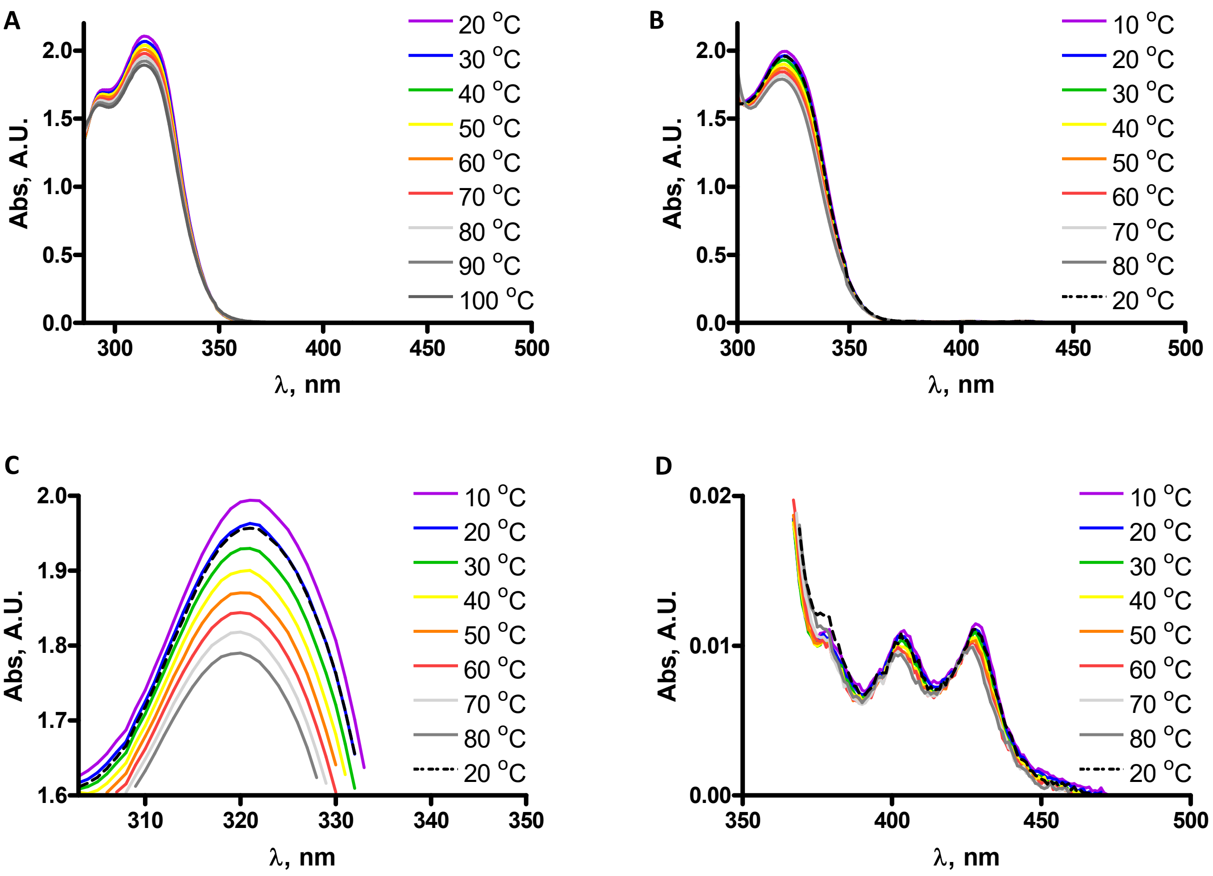

Figure S43. Plots of Abs vs wavelength for **6** in hexadecane (A, 20–100 °C) or $PhCl_2$ (B-D, 10–80 °C). Plots C and D are expanded sections of B. Solid lines refer to spectra collected while increasing temperature and the broken line refers to a spectrum recorded after cooling the sample down to 20 °C from 80 °C.

Cross-over experiment of 2d with 6

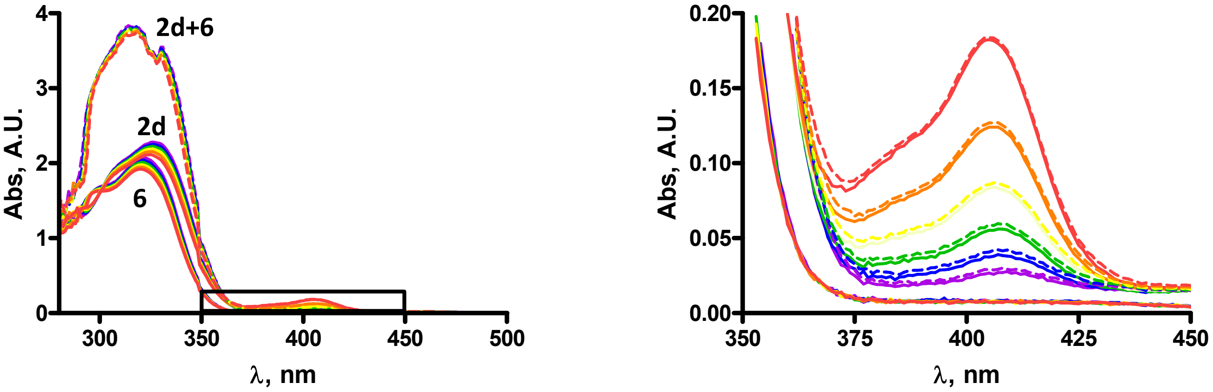

Figure S44. Plots of Abs vs wavelength for **2d** (50 µM), **6** (50 µM), and a combination of **2d** (50 µM) and **6** (50 µM) (broken lines). The scans were collected in $PhCl_2$ at 10 (violet), 20 (blue), 30 (green), 40 (yellow), 50 (orange), 60 (red) °C. The chromophore depicted in the black box is expanded in the plot on the right.

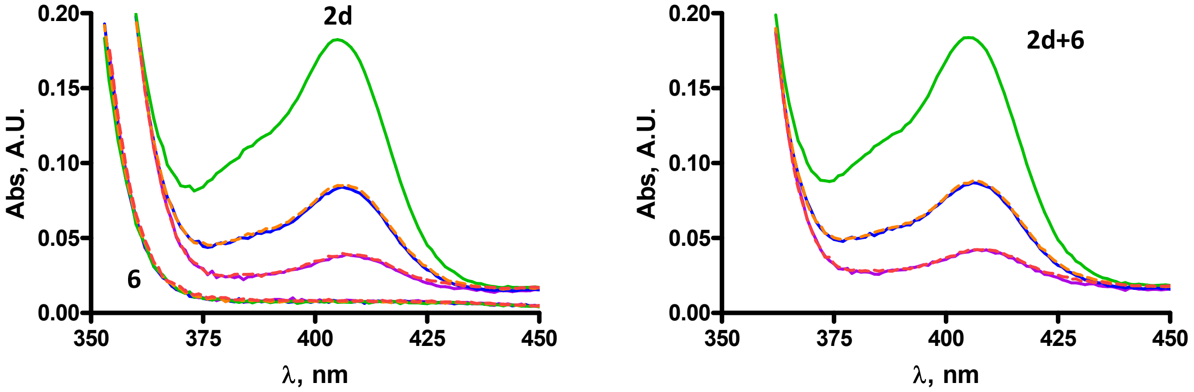

Figure S45. (Left) Plot of Abs vs wavelength for **2d** (prominent absorbance at 404 nm) or **6** (overlaid featureless spectra at bottom). The scans were collected in $PhCl_2$ at 20 (violet), 40 (blue), 60 (green), 40 (orange, dashed), 20 (red, dashed) °C. (Right) Plot of Abs vs wavelength for **2d+6** where the solid traces represent spectra collected while increasing temperature and the dashed lines represent spectra collected while decreasing temperature according to the aforementioned colour scheme. The data shown in both plots were performed as part of the same experiment in a thermostated multi-cell changer.

EPR spectral data for 2d and 6

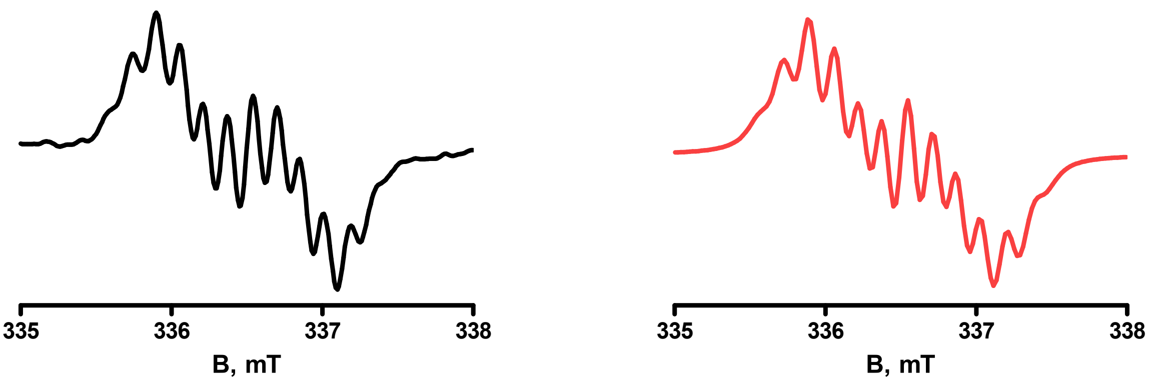

Figure S46. Experimental (black, left) and fitted (red, right) EPR spectra of **2d**-derived radicals in benzene at 20 °C.

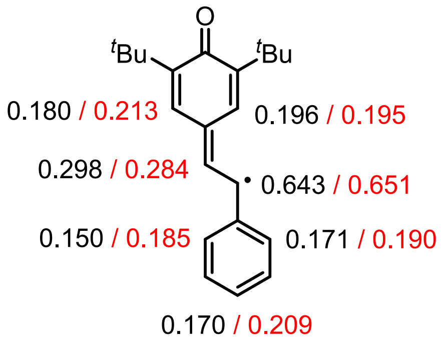

Figure S47. Experimental (black) and calculated (B3LYP/TZVP, red) hyperfine coupling constants for **2d**-derived radicals.

Hammett plots for 1 and 3 at 37, 70, 100 °C

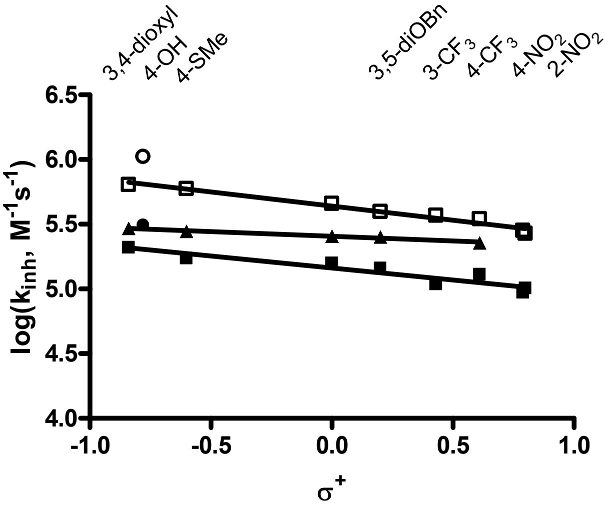

Figure S48. Hammett plot of log(k_{inh}) for the stilbenoid phenol (**1**)-inhibited co-autoxidation of 1-hexadecene (2.9 M) and PBD-BODIPY (10 µM) vs σ^+ in PhCl at 37 (■), 70 (□), and 100 (▲) °C. The data (not including X = 4-OH (●,○) which contains additional phenolic moieties) are fitted to a linear regressions computing ρ^+ = -0.18 ± 0.03, r^2 = 0.8850 (37 °C, ■); ρ^+ = -0.22 ± 0.02, r^2 = 0.9695 (70 °C, □); and ρ^+ = -0.072 ± 0.006, r^2 = 0.9801 (37 °C, ▲), respectively.

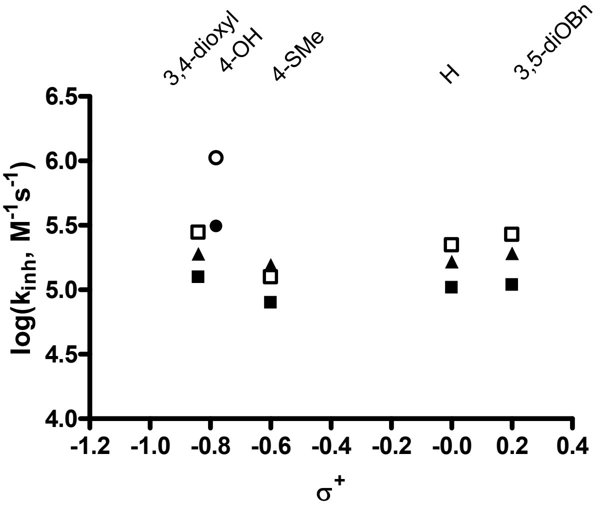

Figure S49. Hammett plot of log(k_{inh}) for the Quad A (**3**)-inhibited co-autoxidation of 1-hexadecene (2.9 M) and PBD-BODIPY (10 µM) vs σ^+ in PhCl at 37 (■), 70 (□), and 100 (▲) °C. The data (not including X = 4-OH (●,○) which contains additional phenolic moieties) exhibit little dependence on the nature of the substituent.

QMD concentration dependences of inhibition rate, time of inhibition, or stoichiometry

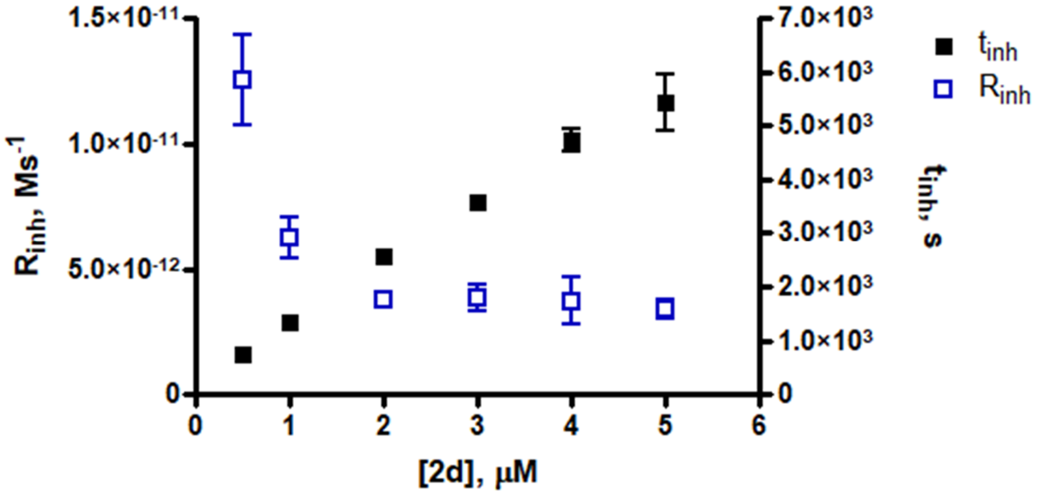

Figure S50. Rate (□) and time (■) of inhibition vs [**2d**] for the QMD-inhibited co-autoxidation of 1-hexadecene (2.9 M) and PBD-BODIPY (10 μM) in chlorobenzene at 37 °C.

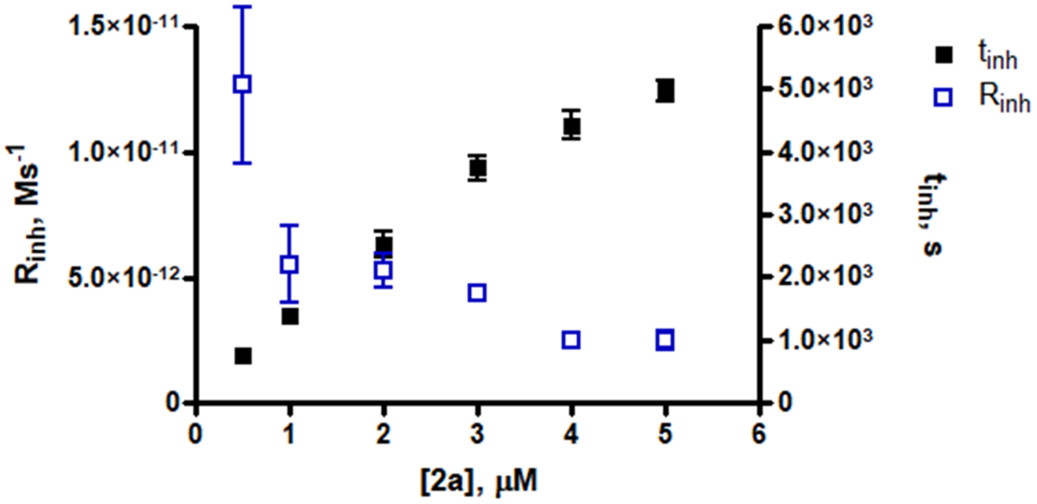

Figure S51. Rate (□) and time (■) of inhibition vs [**2a**] for the QMD-inhibited co-autoxidation of 1-hexadecene (2.9 M) and PBD-BODIPY (10 μM) in chlorobenzene at 37 °C.

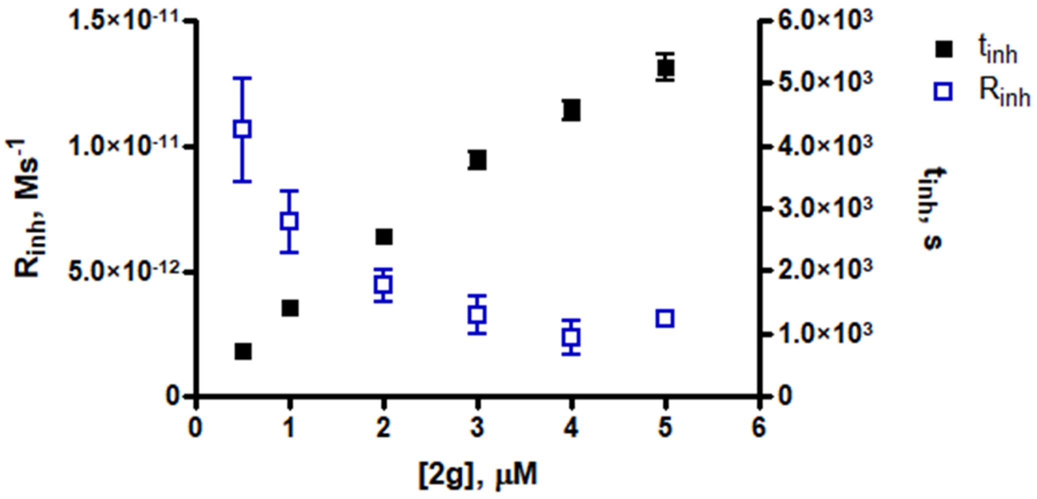

Figure S52. Rate (□) and time (■) of inhibition vs [**2g**] for the QMD-inhibited co-autoxidation of 1-hexadecene (2.9 M) and PBD-BODIPY (10 µM) in chlorobenzene at 37 °C.

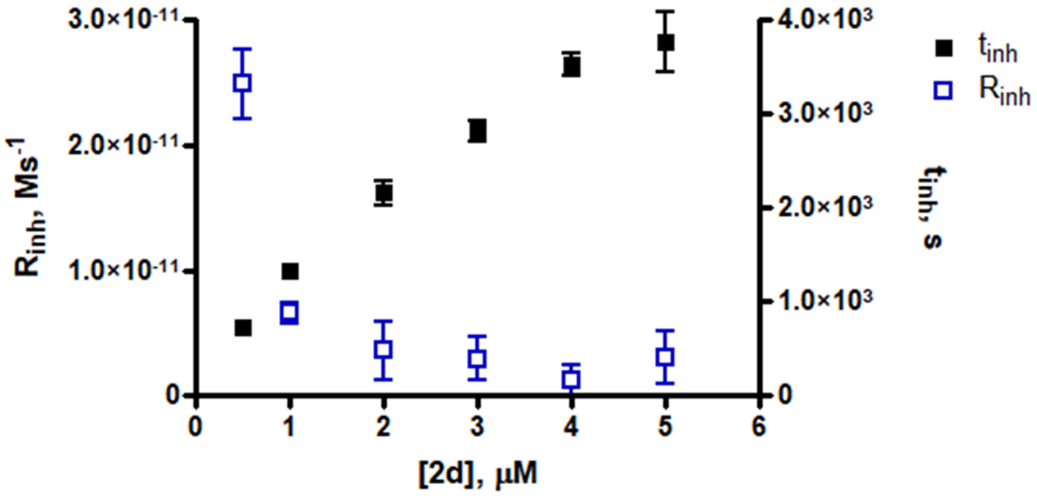

Figure S53. Rate (□) and time (■) of inhibition vs [**2d**] for the QMD-inhibited co-autoxidation of 1-hexadecene (2.9 M) and PBD-BODIPY (10 µM) in chlorobenzene at 70 °C.

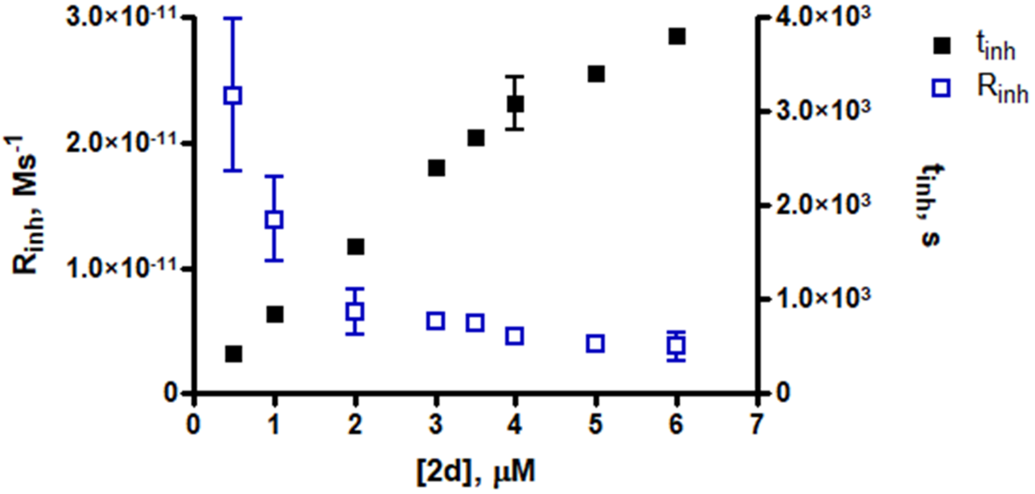

Figure S54. Rate (□) and time (■) of inhibition vs [**2d**] for the QMD-inhibited co-autoxidation of 1,4-dioxane (2.9 M) and PBD-BODIPY (10 μM) in chlorobenzene at 37 °C.

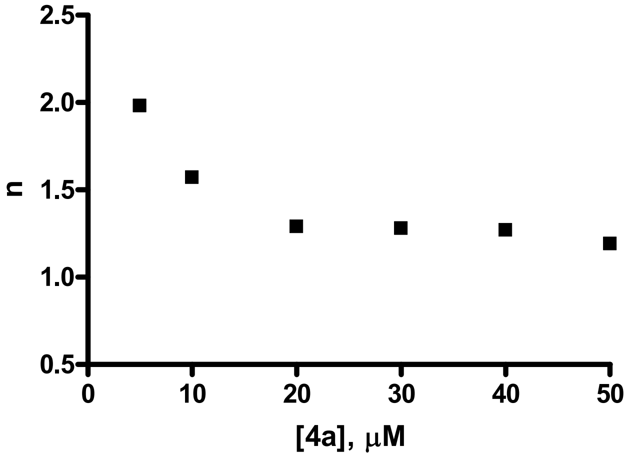

Figure S55. Stoichiometry (n) vs [**4a**] for the QM-inhibited co-autoxidation of 1-hexadecene (2.9 M) and PBD-BODIPY (10 μM) in chlorobenzene at 37 °C.

UV-vis spectra and corresponding Van't Hoff plots for 2

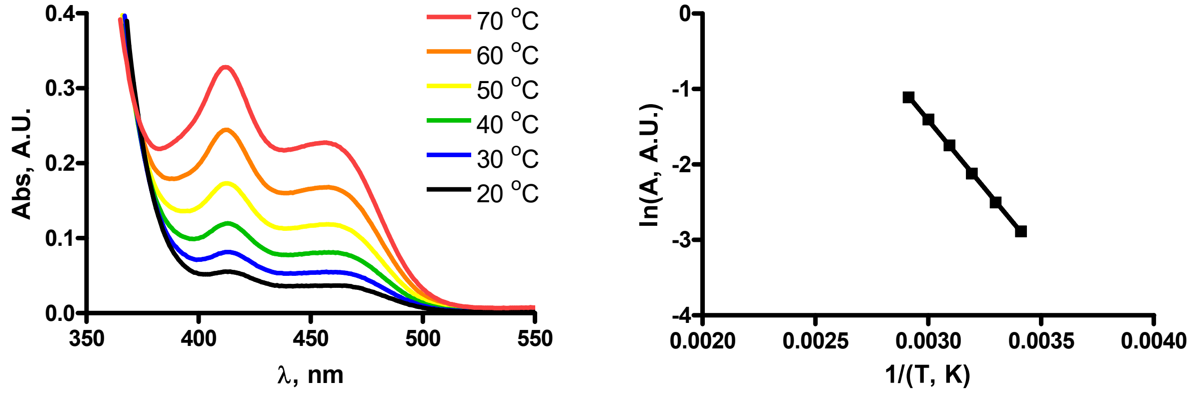

Figure S56. (Left) Absorbance vs wavelength (nm) for **2a** at different temperatures (20–70 °C) in 1,2-dichlorobenzene. (Right) Van't Hoff plot used to determine $\Delta H_{C\text{–}C}$ for **2a** at λ_{max} = 412 nm (ΔH = 14.3 ± 0.1, r^2 = 0.9997).

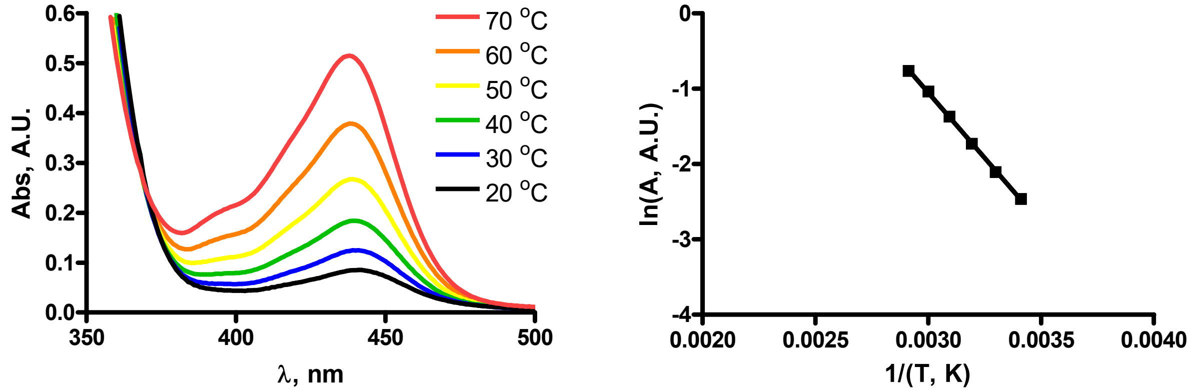

Figure S57. (Left) Absorbance vs wavelength (nm) for **2b** at different temperatures (20–70 °C) in 1,2-dichlorobenzene. (Right) Van't Hoff plot used to determine $\Delta H_{C\text{–}C}$ for **2b** at λ_{max} = 438 nm (ΔH = 13.8 ± 0.2, r^2 = 0.9994).

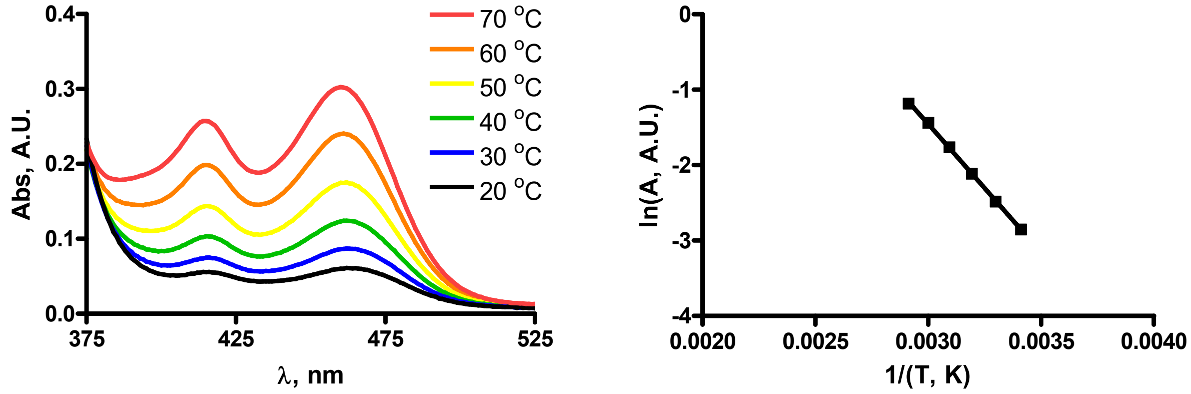

Figure S58. (Left) Absorbance vs wavelength (nm) for **2c** at different temperatures (20–70 °C) in 1,2-dichlorobenzene. (Right) Van't Hoff plot used to determine $\Delta H_{C\text{–}C}$ for **2c** at λ_{max} = 460 nm (ΔH = 13.5 ± 0.2, r^2 = 0.9995).

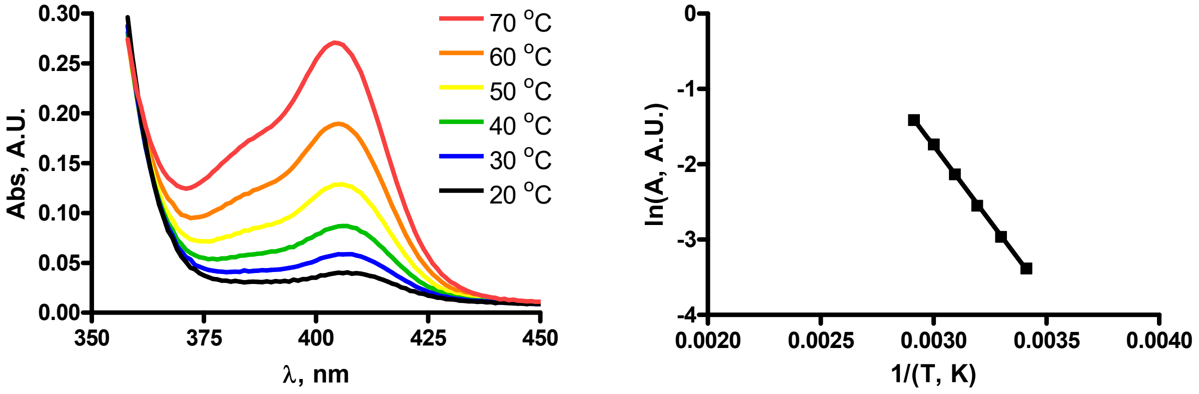

Figure S59. (Left) Absorbance vs wavelength (nm) for **2d** at different temperatures (20–70 °C) in 1,2-dichlorobenzene. (Right) Van't Hoff plot used to determine $\Delta H_{C\text{–}C}$ for **2d** at λ_{max} = 404 nm (ΔH = 15.9 ± 0.2, r^2 = 0.9994).

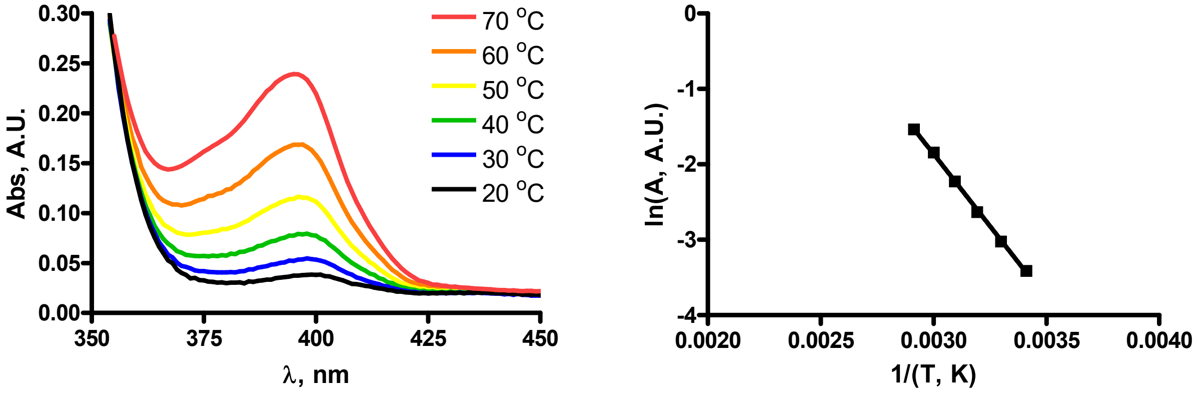

Figure S60. (Left) Absorbance vs wavelength (nm) for **2f** at different temperatures (20–70 °C) in 1,2-dichlorobenzene. (Right) Van't Hoff plot used to determine $\Delta H_{C\text{–}C}$ for **2f** at λ_{max} = 395 nm (ΔH = 15.2 ± 0.2, r^2 = 0.9990).

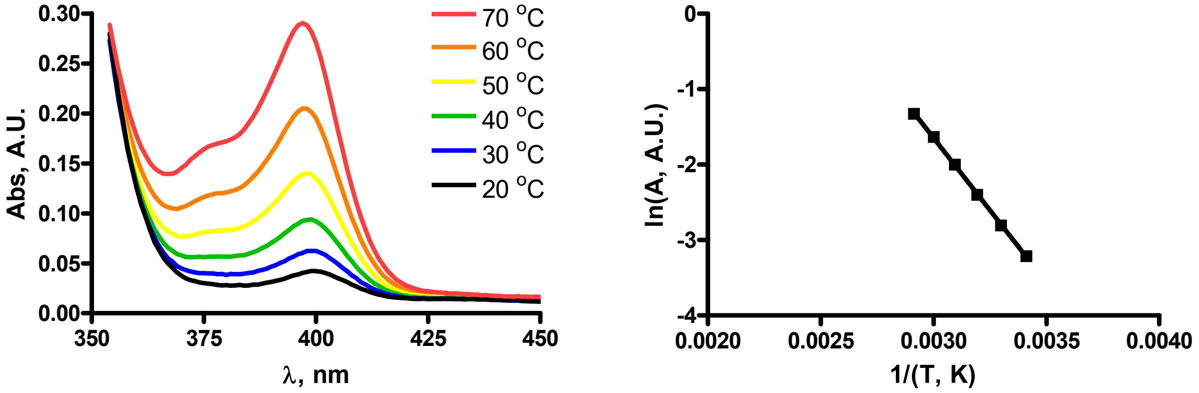

Figure S61. (Left) Absorbance vs wavelength (nm) for **2g** at different temperatures (20–70 °C) in 1,2-dichlorobenzene. (Right) Van't Hoff plot used to determine $\Delta H_{C\text{–}C}$ for **2g** at λ_{max} = 397 nm (ΔH = 15.3 ± 0.2, r^2 = 0.9996).

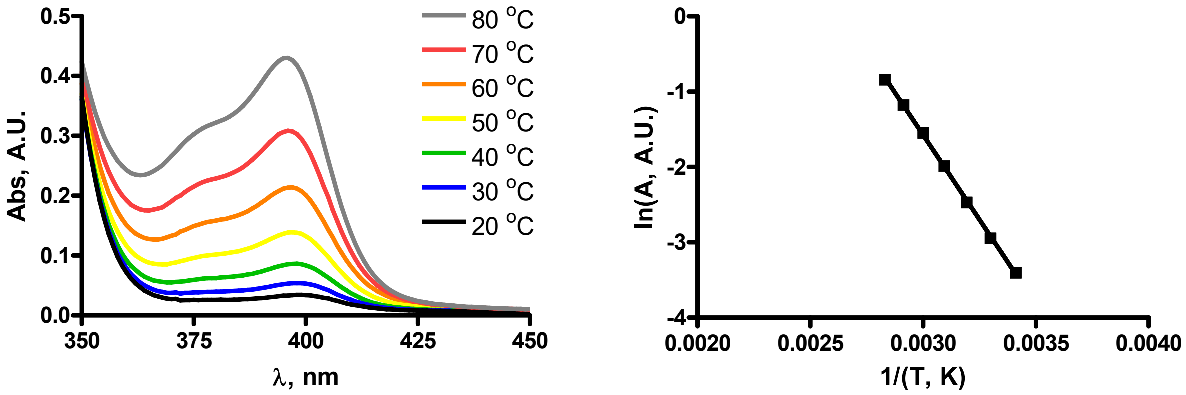

Figure S62. (Left) Absorbance vs wavelength (nm) for **2h** at different temperatures (20–80 °C) in 1,2-dichlorobenzene. (Right) Van't Hoff plot used to determine ΔH_{C-C} for **2h** at λ_{max} = 395 nm (C, ΔH = 17.9 ± 0.2, r^2 = 0.9992).

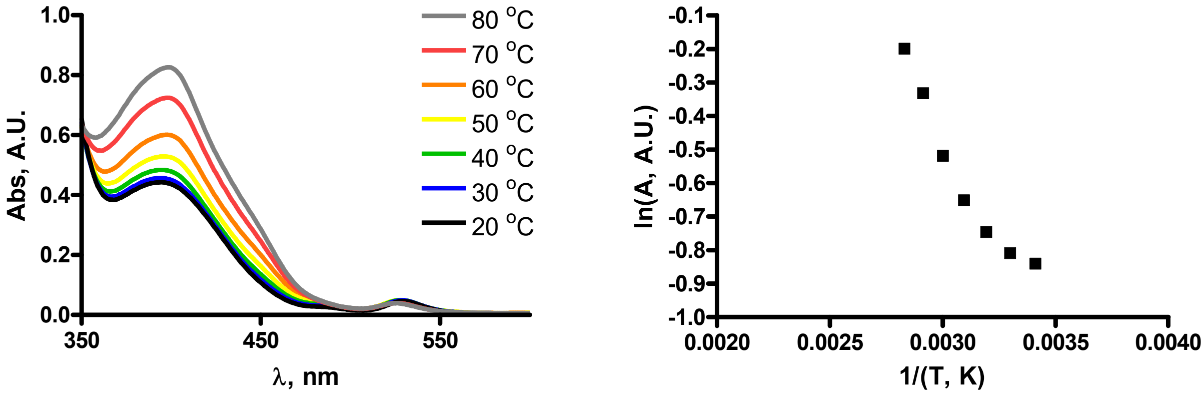

Figure S63. (Left) Absorbance vs wavelength (nm) for **2i** at different temperatures (20–80 °C) in 1,2-dichlorobenzene. (Right) Van't Hoff plot for **2i** at λ_{max} = 402 nm.

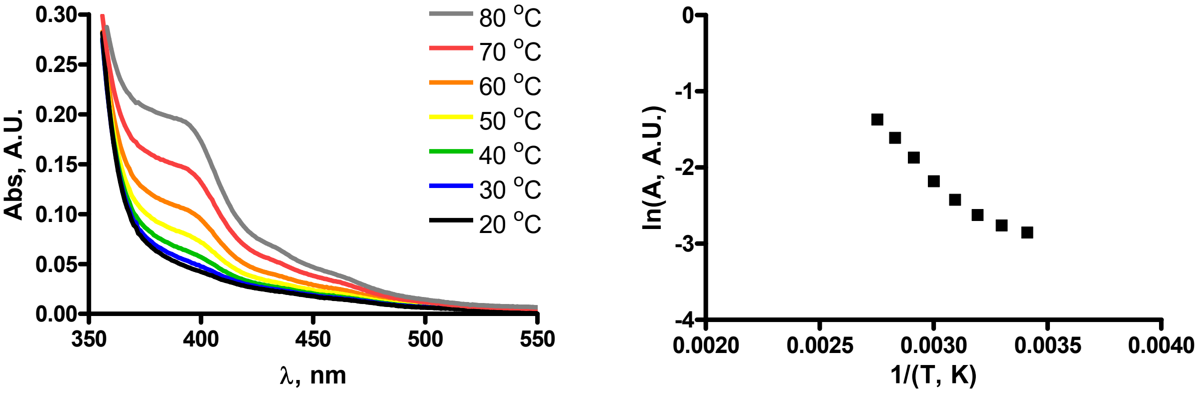

Figure S64. (Left) Absorbance vs wavelength (nm) for **2j** at different temperatures (20–80 °C) in 1,2-dichlorobenzene. (Right) Van't Hoff plot for **2j** at λ_{max} = 384 nm.

Comparison of experimental BDEs to DFT and dispersion-corrected DFT values

Table S4. Summary of computationally (R = H) and experimentally (R = ^{t}Bu) determined bond dissociation enthalpies (kcal·mol^{-1}) associated with substituted quinone methide dimers. The experimental ΔH_{exp} values are the average of triplicate measurements. The computed ΔH_{DFT} values were determined using B3LYP/CBSB7 and the ΔH_{GD3} values were similarly determined including dispersion correction (GD3).

Compound	X	σ^+	ΔH_{DFT}	ΔH_{GD3}	ΔH_{exp}	λ, nm
	4-NO_2	0.79	1.0	13.0		
	4-CF_3	0.61	2.0	14.6	16.0 ± 0.3	397
	4-CO_2H	0.42	1.8	14.0		
	2-CF_3				17.9 ± 0.3	395
	3-CF_3				15.3 ± 0.4	395
diAr-QMD	3,5-diOBn[a]	0.20			17.0 ± 0.7	414
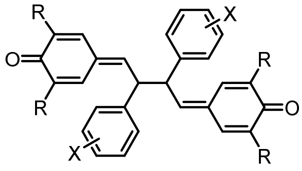	4-Cl	0.11	1.6	13.7		
	H	0.00	2.5	14.4	15.7 ± 0.3	404
	4-SH	-0.03	0.7	12.9		
	4-CH_3	-0.31	1.9	14.6		
	4-SCH_3	-0.60	0.5	13.6	13.5 ± 0.3	460
	4-OCH_3	-0.78	0.9	12.6		
	4-OH	-0.78	0.8	12.5		
	4-OBn	-0.83			14.1 ± 0.3	438
	3,4-dioxyl	-0.84	0.0	14.4	14.0 ± 0.3	412
	4-NH_2	-1.3	-0.9	11.2		
diMe-QMD	NA		14.7	22.0	>19	

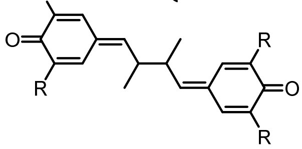

[a] M. H. Keylor, B. S. Matsuura, M. Griesser, J.-P. R. Chauvin, R. A. Harding, M. S. Kirillova, X. Zhu, O. J. Fischer, D. A. Pratt, C. R. J. Stephenson, *Science*, 2016, **354**, 1260.

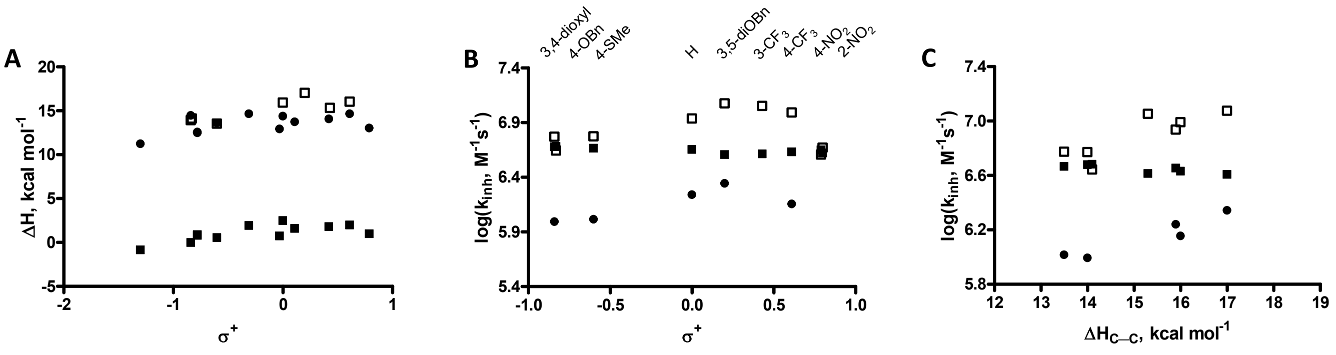

Figure S65. A) Plots of $\Delta H_{C\text{–}C}$ vs σ^+ for experimental (□), DFT (■), and dispersion-corrected DFT (●) C–C values for substituted QMDs. The relationship between $\Delta H_{C\text{–}C}$ and σ^+ exhibits little sensitivity to electron-withdrawing substituents ($\rho^+_{C\text{–}C}$ ~ 0 for $\sigma^+ > 0$); electron-donating substituents exhibit $\rho^+_{C\text{–}C}$ = 1.9 ± 0.5 (DFT), 1.4 ± 0.9 (dispersion-corrected DFT), 1.4 ± 0.7 (experiment). B) Plots of log(k_{inh}) vs σ^+ for **2a-j** at 37 (■), 70 (□), and 100 (●) °C. C) Plots of log(k_{inh}) vs $\Delta H_{C\text{–}C}$ at 37 (■), 70 (□), and 100 (●) °C. The relationship between $\Delta H_{C\text{–}C}$ and σ^+ is reflected in log(k_{inh}) vs σ^+ (at 70 and 100 °C) and depicted in C.

Extinction coefficient determinations for 2d, 4a, and 5b

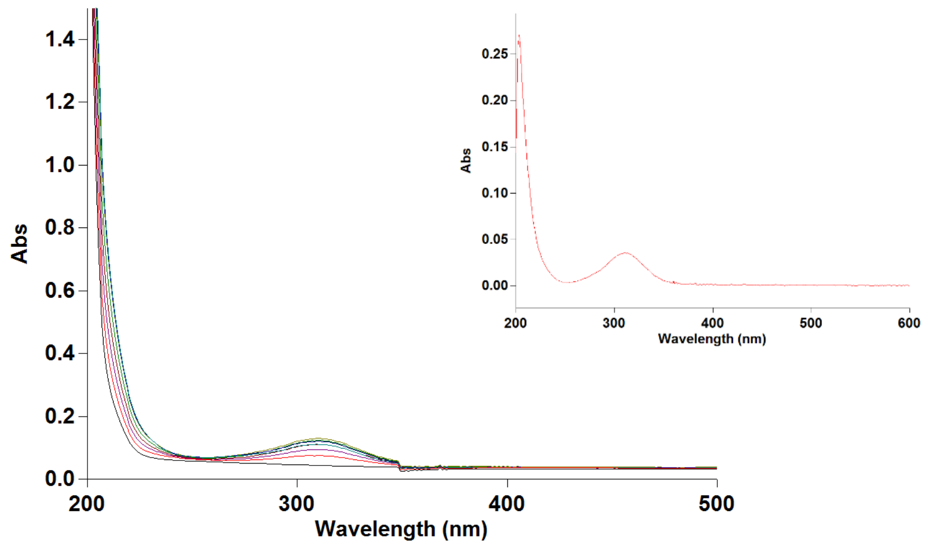

Figure S66. Spectrophotometric titration of **4a** in PBS buffer (pH = 7.4) at 37 °C. The inset spectrum shows the difference between two spectra/concentrations. Due to interferences caused by hydration under these conditions, an approximate extinction coefficient was determined: ε_{310nm} = 8000 ± 500 $M^{-1}cm^{-1}$.

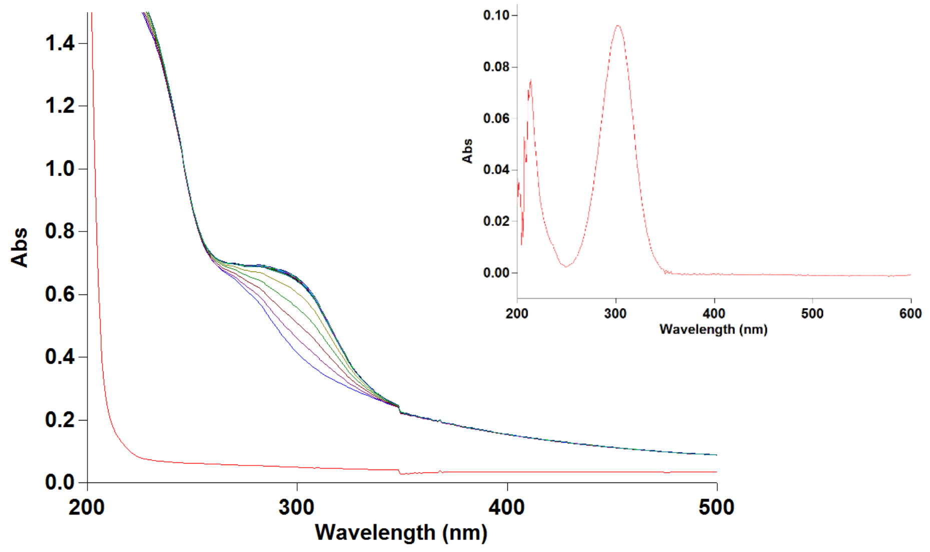

Figure S67. Spectrophotometric titration of **4a** in PBS buffer (pH = 7.4) containing liposomes at 37 °C. The inset spectrum shows the difference between two spectra/concentrations – the shape is similar to that observed in organic solution. Under these conditions, **4a** is not sensitive to hydration, suggesting it is sequestered within the liposome. The extinction coefficient was determined: ε_{302nm} = 24420 ± 80 $M^{-1}cm^{-1}$.

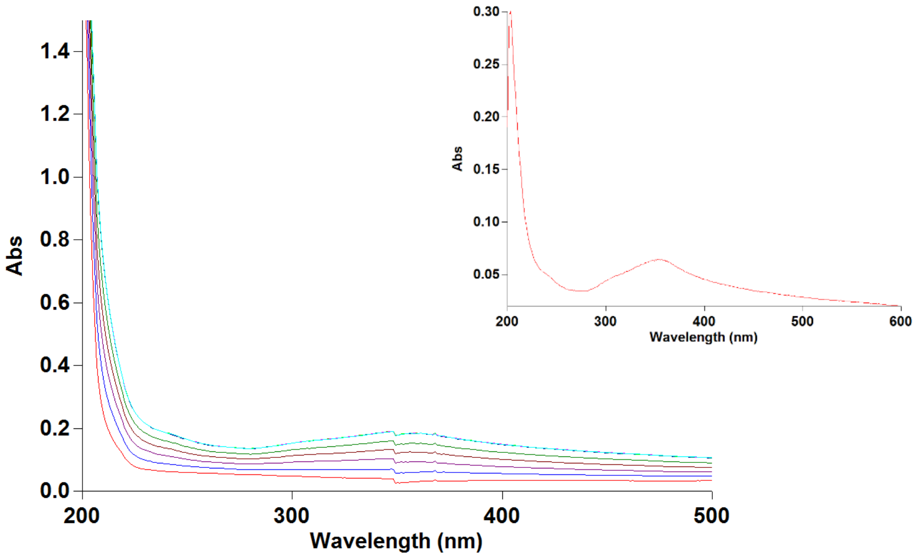

Figure S68. Spectrophotometric titration of **2d** in PBS buffer (pH = 7.4) at 37 °C. The inset spectrum shows the difference between two spectra/concentrations. The extinction coefficient was determined: ε_{353nm} = 15300 ± 300 $M^{-1}cm^{-1}$.

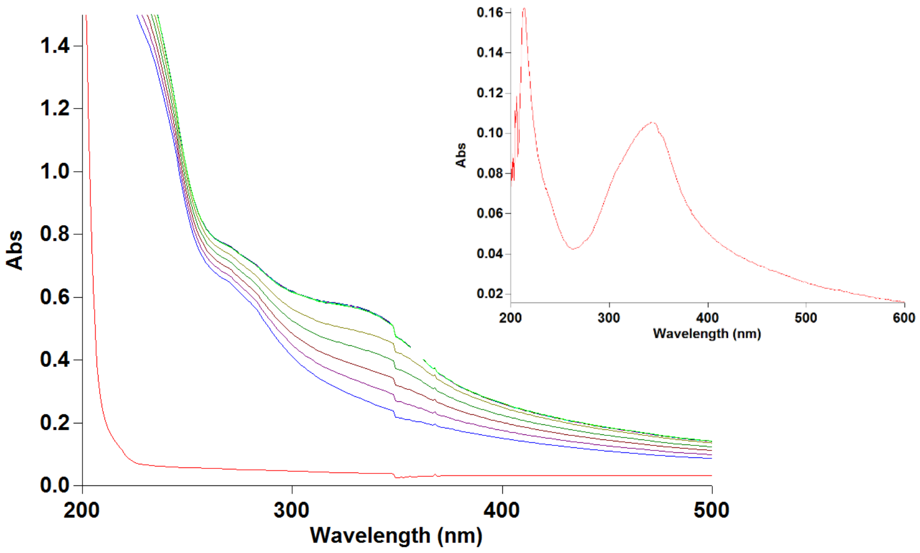

Figure S69. Spectrophotometric titration of **2d** in PBS buffer (pH = 7.4) containing liposomes at 37 °C. The inset spectrum shows the difference between two spectra/concentrations – the shape is dissimilar to that observed in organic solution. Importantly, no spectral features associated with dissociated radical monomers is observed. The extinction coefficient was determined: ε_{340nm} = 29000 ± 1000 $M^{-1}cm^{-1}$.

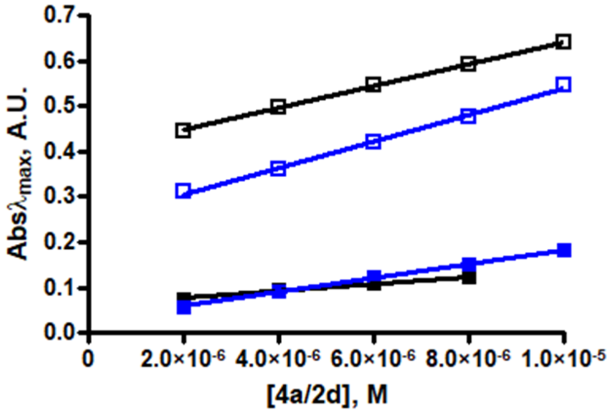

Figure S70. Determination of extinction coefficients for **4a** (PBS buffer, ε_{310nm} = 8000 ± 500 $M^{-1}cm^{-1}$, r^2 = 0.9916, ■; PBS buffer containing liposomes, ε_{302nm} = 24420 ± 80 $M^{-1}cm^{-1}$, r^2 = 1.000, □) and **2d** (PBS buffer, ε_{302nm} = 24420 ± 80 $M^{-1}cm^{-1}$, r^2 = 0.9988, ■; PBS buffer containing liposomes, ε_{340nm} = 29000 ± 1000 $M^{-1}cm^{-1}$, r^2 = 0.9966, □) at 37 °C.

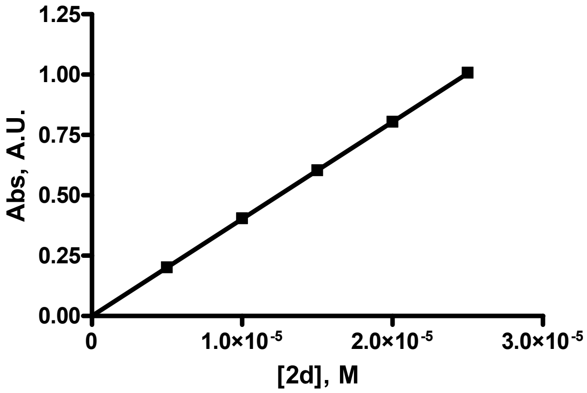

Figure S71. Determination of extinction coefficients for **2d** in 2.9 M hexadecene in chlorobenzene at 37 °C; ε_{330nm} = 40200 ± 100 $M^{-1}cm^{-1}$, r^2 = 1.000.

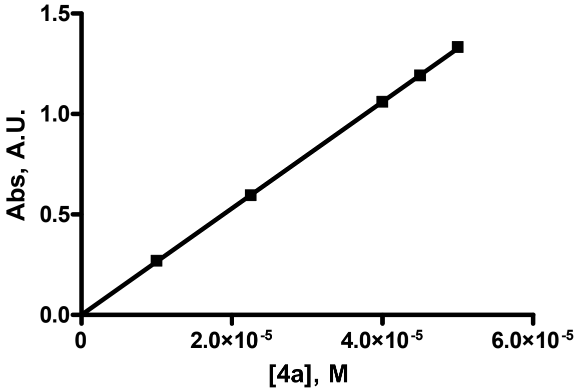

Figure S72. Determination of extinction coefficients for **4a** in 2.9 M hexadecene in chlorobenzene at 37 °C; ε_{205nm} = 26500 ± 100 $M^{-1}cm^{-1}$, r^2 = 0.9999.

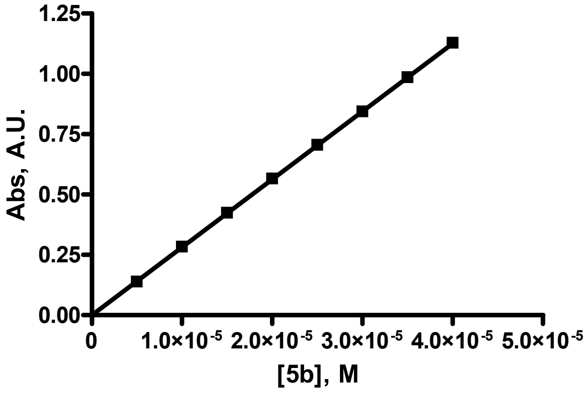

Figure S73. Determination of extinction coefficients for **5b** in 2.9 M hexadecene in chlorobenzene at 37 °C; ε_{317nm} = 28170 ± 60 $M^{-1}cm^{-1}$, r^2 = 1.000.

Characterization data for synthesized compounds

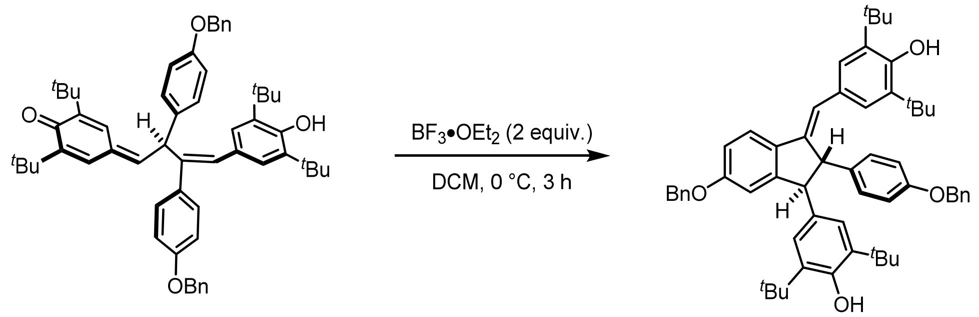

S1 **S2**

(S2) 4-((1*S*,2*S*)-6-(benzyloxy)-2-(4-(benzyloxy)phenyl)-3-((*E*)-3,5-di-*tert*-butyl-4-hydroxybenzylidene)-2,3-dihydro-1H-inden-1-yl)-2,6-di-*tert*-butylphenol

^{1}H NMR (500 MHz, Chloroform-*d*) δ 7.43 – 7.27 (m, 12H), 7.16 (d, *J* = 9.1 Hz, 1H), 7.14 (s, 2H), 7.01 (s, 2H), 6.98 (s, 1H), 6.87 (d, *J* = 8.9 Hz, 2H), 6.77 (m, 2H), 5.04 (s, 2H), 5.01 (s, 2H), 5.01 (s, 1H), 4.97 (s, 1H), 4.12 (s, 1H), 4.04 (s, 1H), 1.34 (s, 18H), 1.29 (s, 18H).

^{13}C NMR (176 MHz, Chloroform-*d*) δ 158.27, 158.05, 152.37, 152.33, 138.22, 137.43, 137.32, 137.20, 136.74, 135.58, 135.55, 134.49, 134.05, 128.70, 128.65, 128.06, 127.97, 127.56, 127.52, 127.17, 124.18, 123.96, 123.05, 116.37, 114.67, 112.94, 70.13, 70.03, 53.62, 50.27, 34.52, 34.47, 30.46, 30.45.

IR (Neat): 3634, 3589, 2957, 2870, 1647, 1594, 1507, 1434, 1237, 1158, 1136, 1029, 734, 697 cm^{-1};

HRMS (ESI) *m/z* calculated for $C_{58}H_{66}O_4^+$ ([M]$^+$) 826.4956, found 826.4952.

^{1}H NMR, 500 MHz, Chloroform-*d*, Compound **S2**

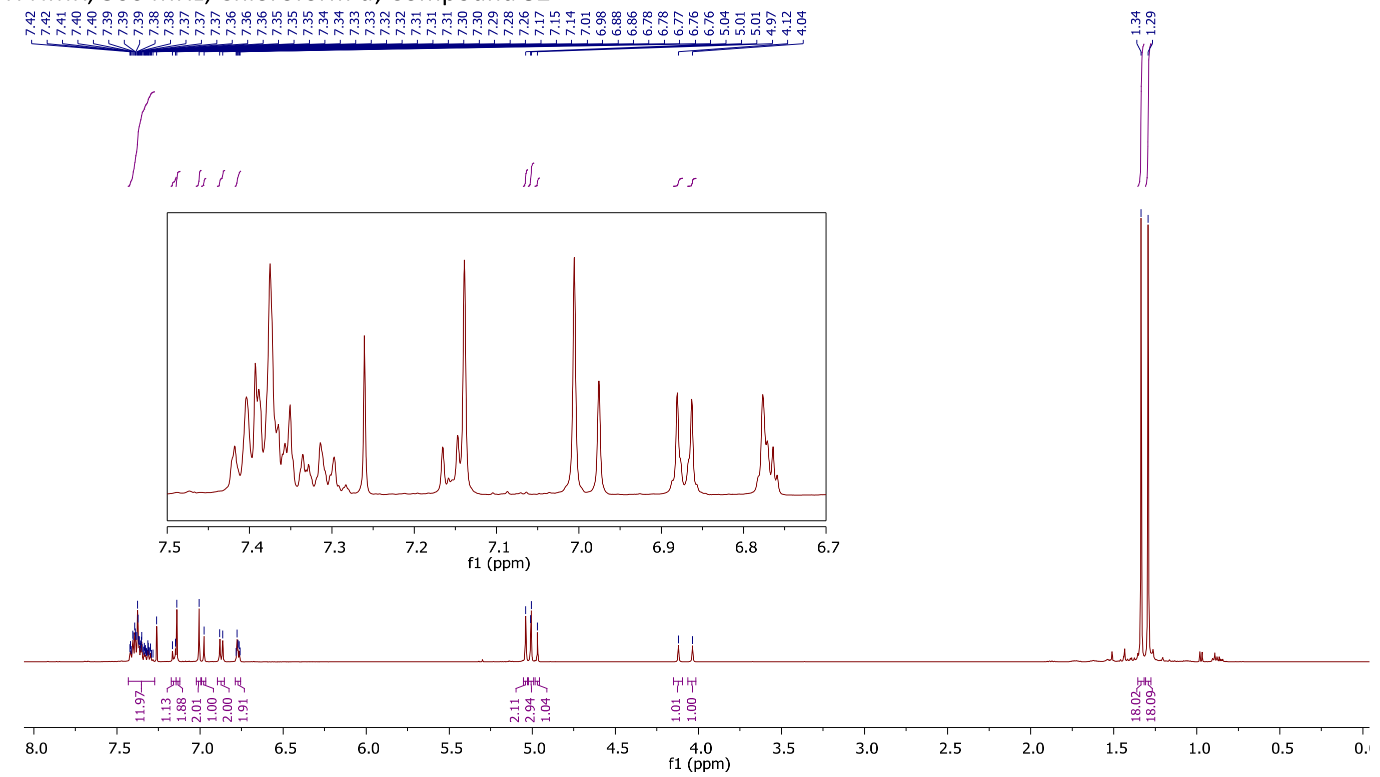

^{13}C NMR, 176 MHz, Chloroform-*d*, Compound **S2**

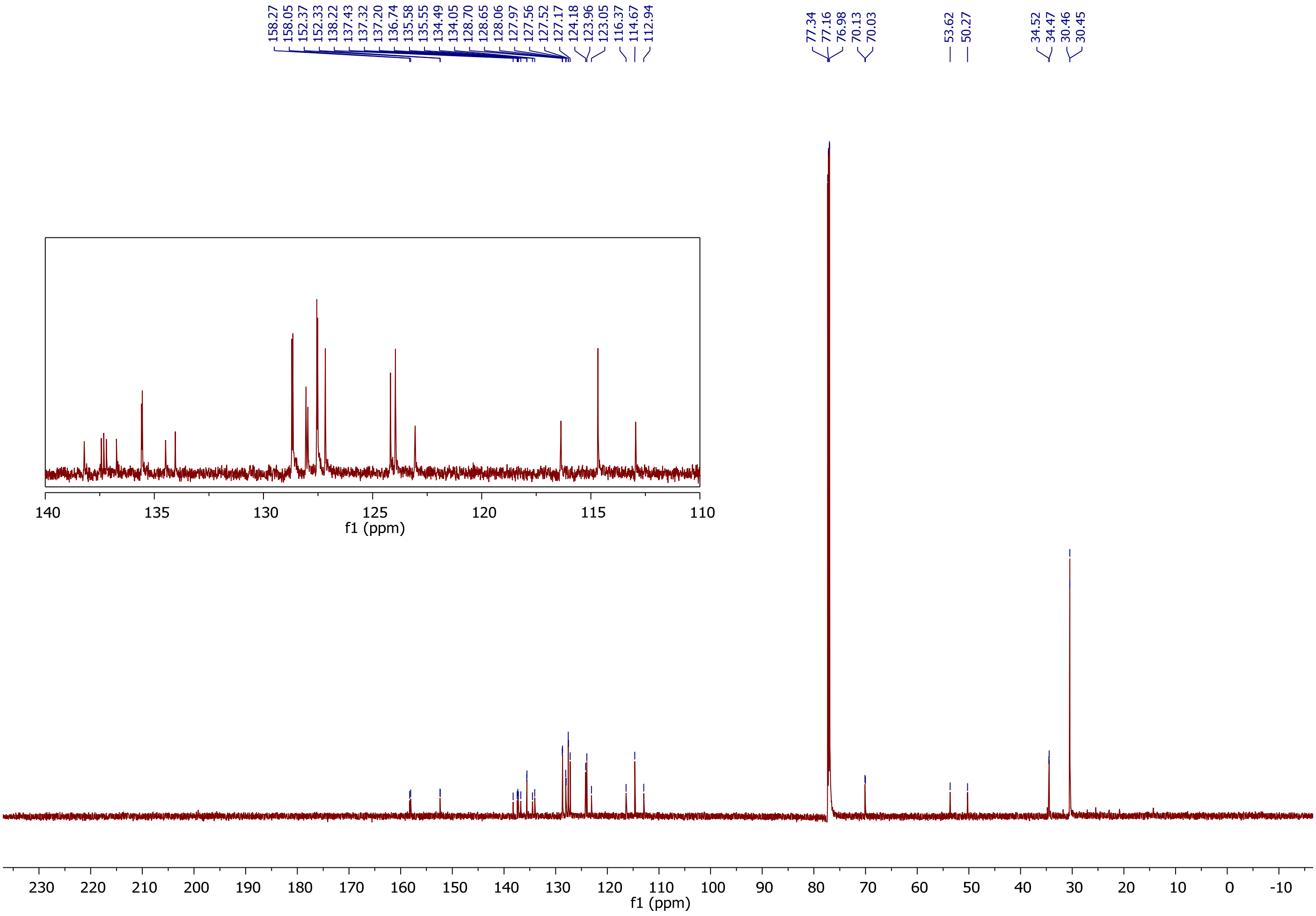

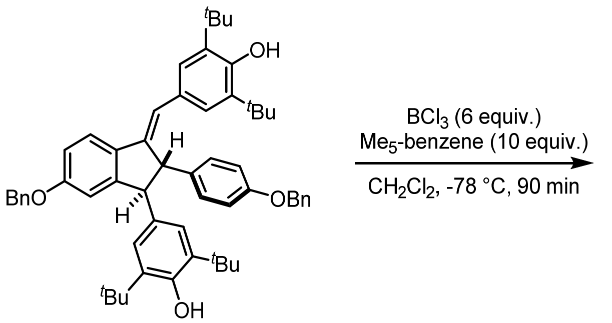

S2 **3b**

(3b) (2*S*,3*S*)-1-((*E*)-3,5-di-*tert*-butyl-4-hydroxybenzylidene)-3-(3,5-di-*tert*-butyl-4-hydroxyphenyl)-2-(4-hydroxyphenyl)-2,3-dihydro-1H-inden-5-ol

^{1}H NMR (700 MHz, Chloroform-*d*) δ 7.31 (d, *J* = 8.3 Hz, 2H), 7.12 (s, 2H), 7.12 (d, *J* = 8.1 Hz, 1H), 7.00 (s, 2H), 6.95 (s, 1H), 6.71 (d, *J* = 8.7 Hz, 2H), 6.63 (dd, *J* = 8.1, 2.8 Hz, 1H), 6.58 (d, *J* = 2.5 Hz, 1H), 5.01 (s, 1H), 4.97 (s, 1H), 4.60 (s, 1H), 4.52 (s, 1H), 4.08 (s, 1H), 4.00 (s, 1H), 1.33 (s, 18H), 1.29 (s, 18H).

^{13}C NMR (176 MHz, Chloroform-*d*) δ 154.79, 154.67, 152.38, 138.41, 137.24, 136.65, 135.60, 135.59, 134.47, 133.95, 127.86, 127.81, 127.36, 124.13, 123.91, 123.00, 116.67, 115.14, 113.55, 53.52, 50.30, 34.51, 34.46, 30.43, 30.41.

IR (Neat): 3628, 2959, 1656, 1595, 1558, 1507, 1459, 1361, 1244, 1197, 1024, 878, 836, 668 cm^{-1};

HRMS (ESI) *m/z* calculated for $C_{44}H_{55}O_4^+$ ([M+H]$^+$) 647.4021, found 647.4029.

^{1}H NMR, 700 MHz, Chloroform-*d*, Compound **3b**

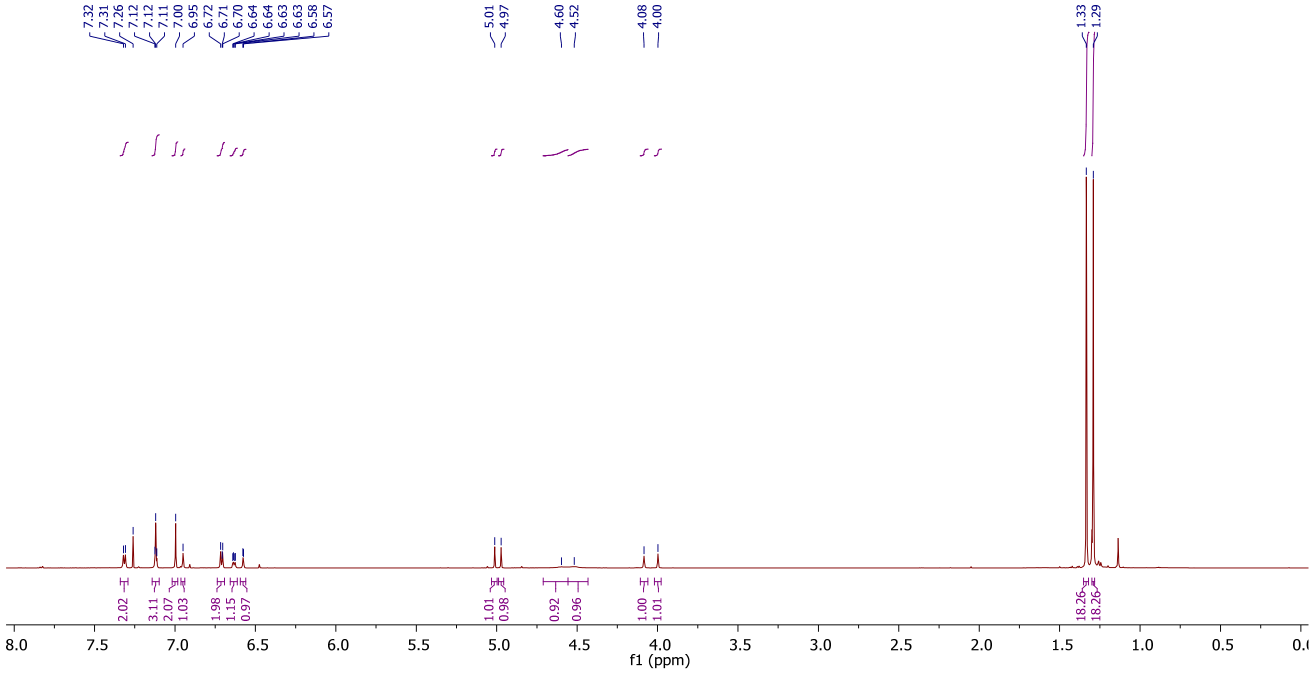

^{13}C NMR, 176 MHz, Chloroform-*d*, Compound **3b**

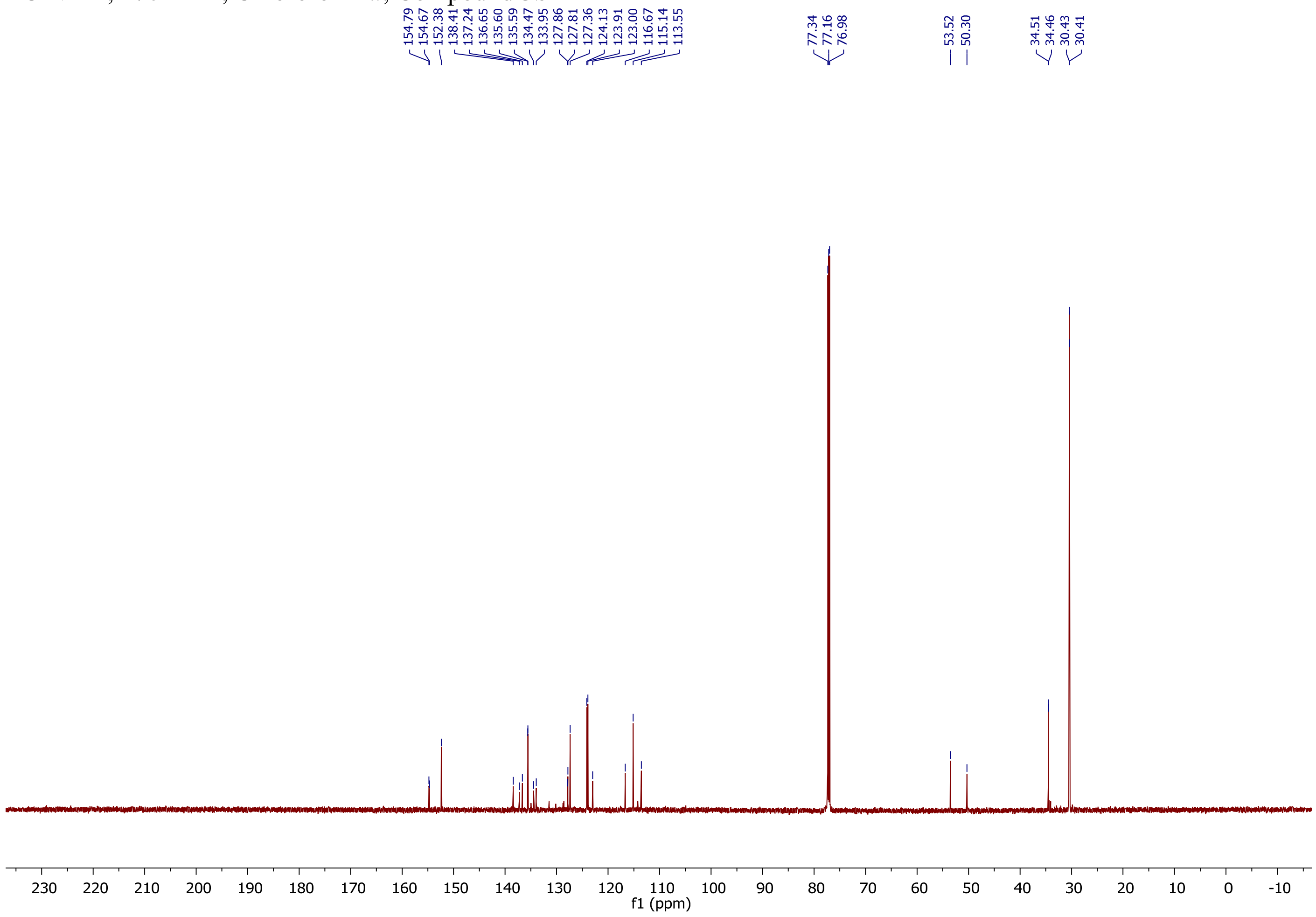

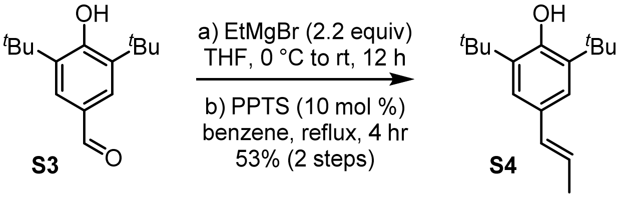

(S4) (E)-2,6-di-tert-butyl-4-(prop-1-en-1-yl)phenol[1]

^{1}H NMR (500 MHz, Chloroform-*d*) δ 7.15 (s, 2H), 6.35 (dq, *J* = 15.6, 1.7 Hz, 1H), 6.06 (dq, *J* = 15.7, 6.6 Hz, 1H), 5.14 (s, 1H), 1.85 (dd, *J* = 6.6, 1.7 Hz, 3H), 1.44 (s, 18H).

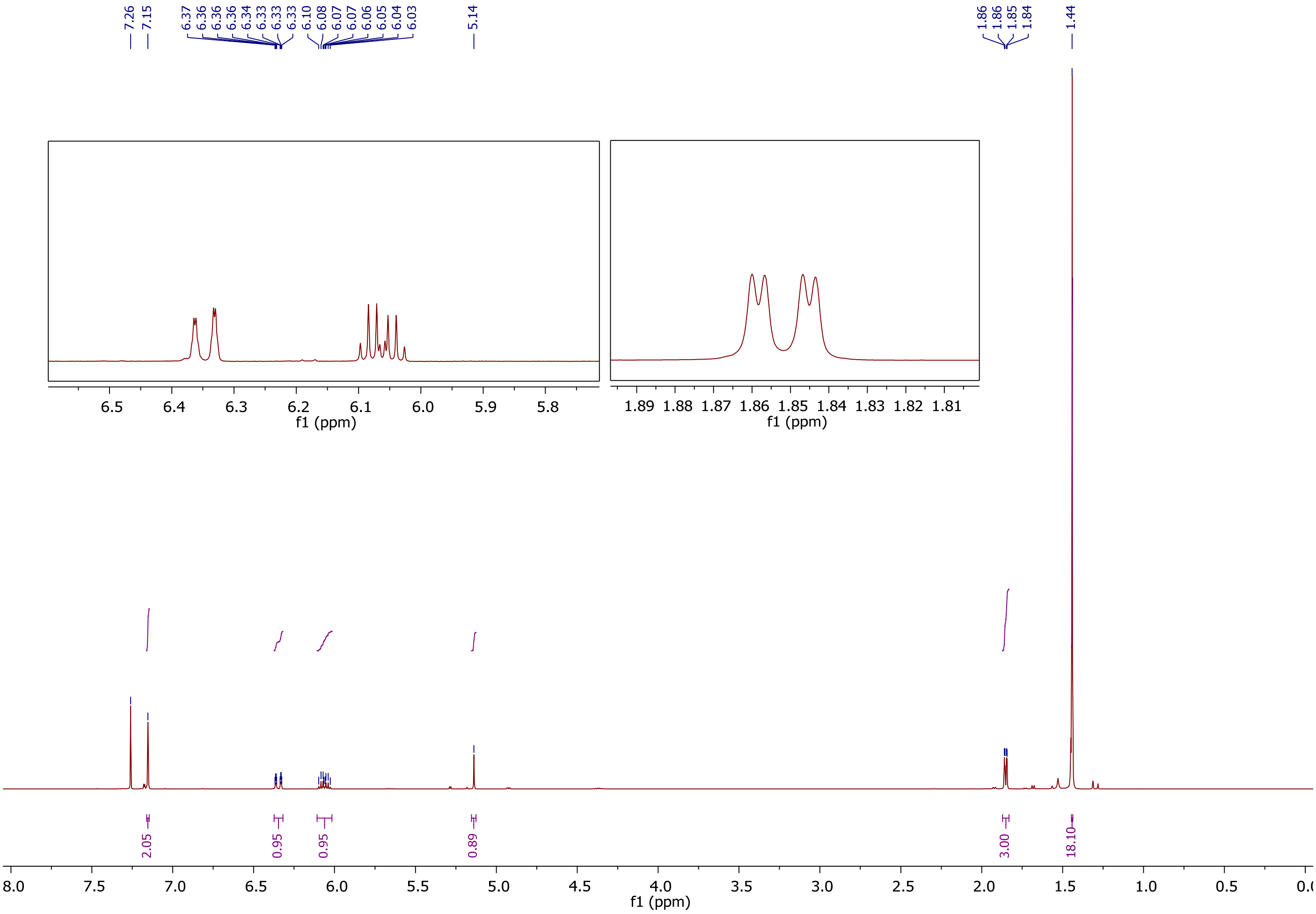

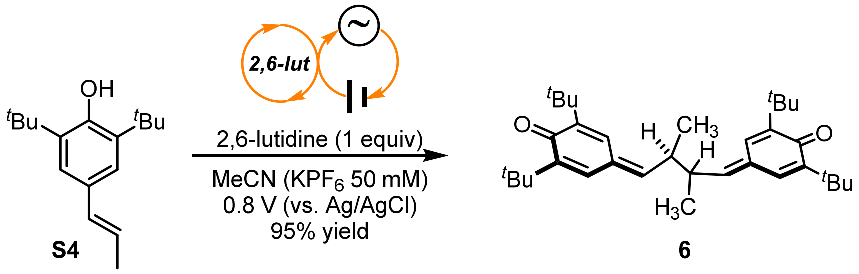

(6) 2,6-di-tert-butyl-4-(4-(3,5-di-tert-butyl-4-hydroxycyclohexa-2,5-dien-1-ylidene)-2,3-dimethylbutylidene)cyclohexa-2,5-dien-1-one

Electrochemical setup: Two pieces of 0.25 × 2-inch RVC panel (0.25 inch thickness) were cut. To each, a hole was made near one end, and copper wire was placed through the hole and wrapped around the top of each electrode. One end of the wire was left free in to connect to the alligator clips. These electrodes were carefully placed into the reaction vial along with the reference electrode (Ag/AgCl in 3 M KCl) and a divider (see image). The alligator clips were connected such that the reference (white) and working (green) electrodes were adjacent to each other, while the counter (red) electrode was opposite the divider. Care was taken to ensure the copper wire was not submerged in solvent, nor the active components of the alligator clips touching each other.

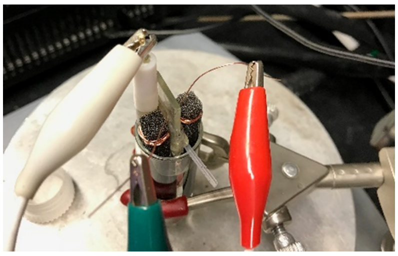

^{1}H NMR (500 MHz, Chloroform-*d*) δ 7.22 (d, *J* = 2.4 Hz, 1H, major diastereomer), 7.19 (d, *J* = 2.4 Hz, 1H, minor diastereomer), 6.84 (d, *J* = 2.4 Hz, 1H, minor diastereomer), 6.80 (d, *J* = 2.4 Hz, 1H, diastereomer), 6.10 (d, *J* = 10.1 Hz, 1H, major + minor diastereomers overlapped), 3.02 (ddd, *J* = 9.7, 8.0, 5.2 Hz, 1H, major diastereomer), 2.98 – 2.91 (m, 1H, minor diastereomer), 1.31 (s, 9H), 1.27 (s, 9H), 1.19 (d, *J* = 6.2 Hz, 3H).[1]

^{13}C NMR (176 MHz, Chloroform-*d*) δ 152.22, 152.21, 145.37, 145.10, 137.79, 137.16, 136.19, 135.45, 134.56, 132.58, 127.44, 126.93, 126.40, 124.26, 124.01, 122.45, 116.78, 115.01, 113.50, 53.72, 50.45, 34.50, 34.45, 30.48, 30.45.

HRMS (ESI) *m/z* calculated for $C_{34}H_{51}O_2$ ([M+H]$^+$) 491.3884, found 491.3879

[1] The methyl and *tert*-butyl signals for the minor diastereomer are obscured by the signals for the major diastereomer.

^{1}H NMR, 500 MHz, Chloroform-*d*, Compound **6**

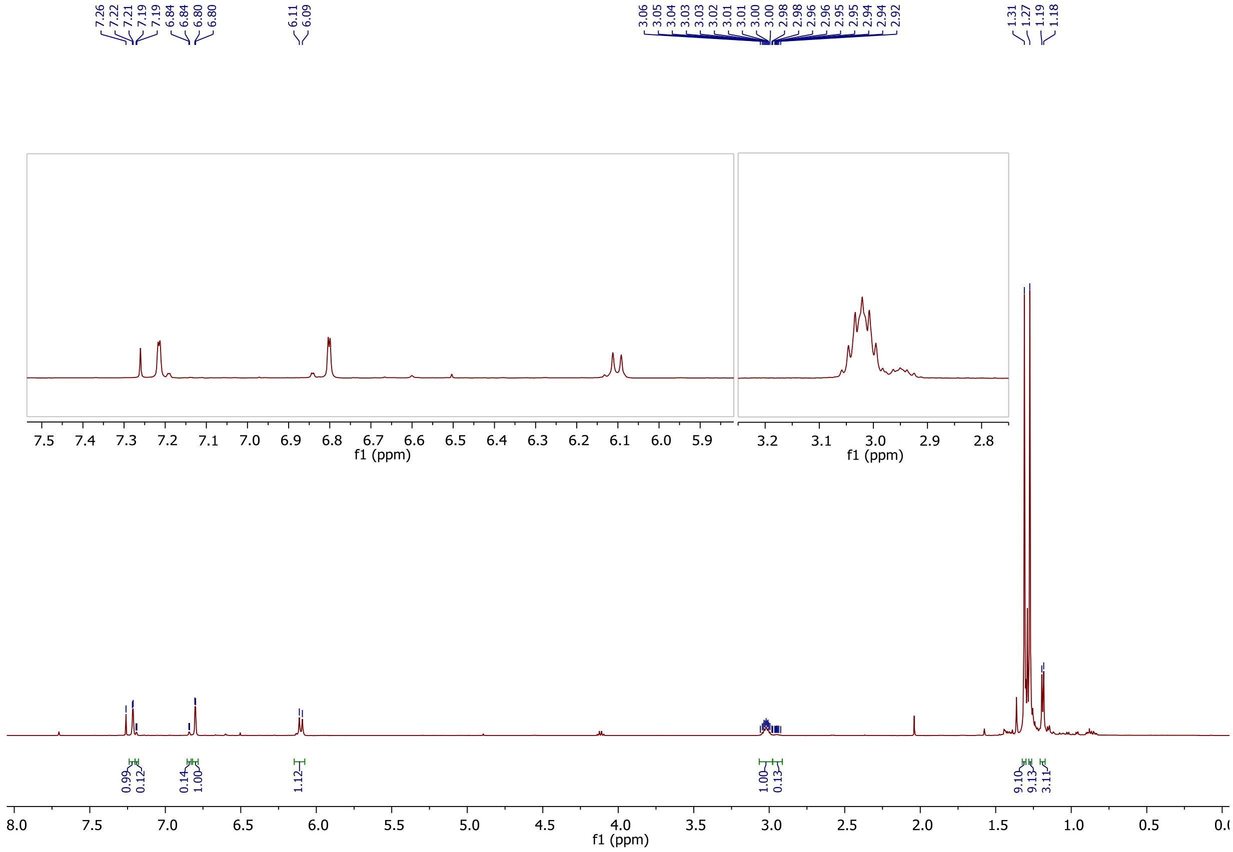

^{13}C NMR, 176 MHz, Chloroform-*d*, Compound **6**

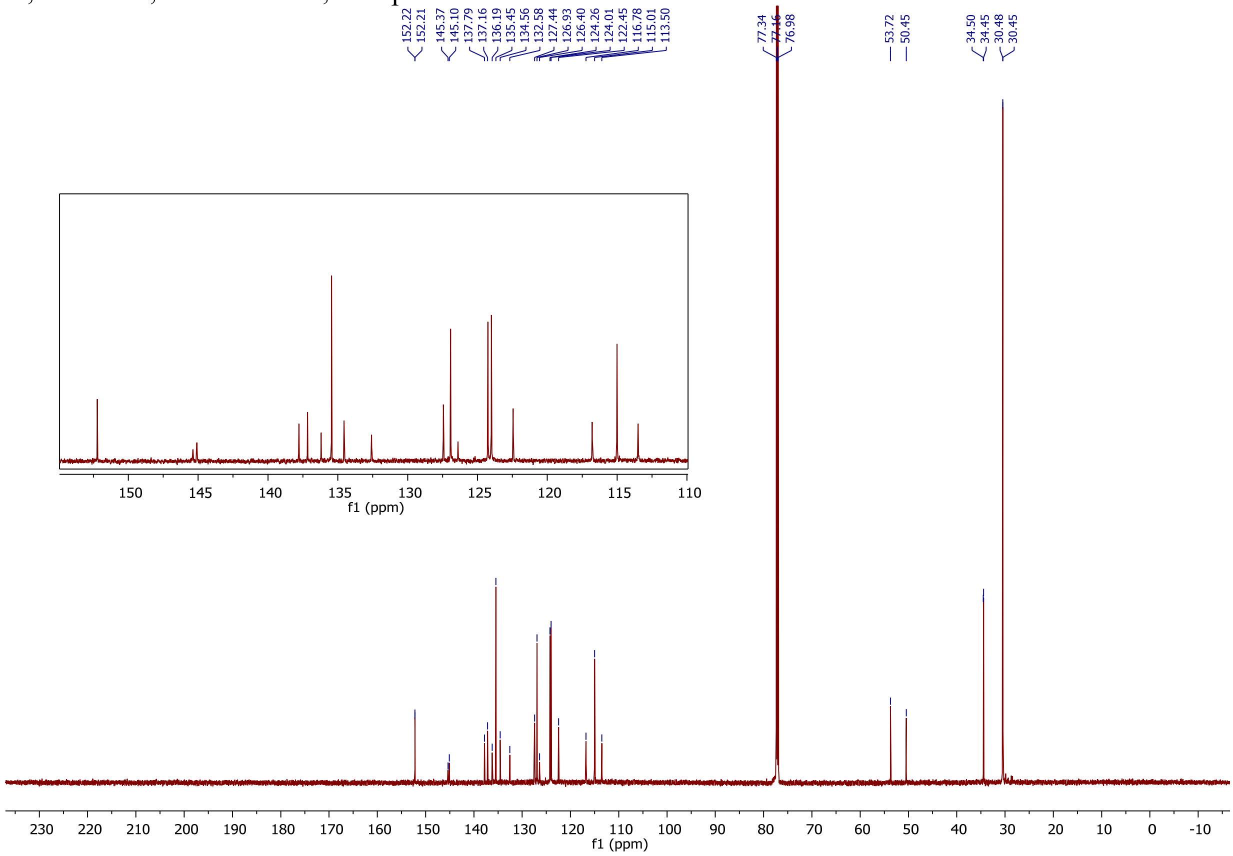

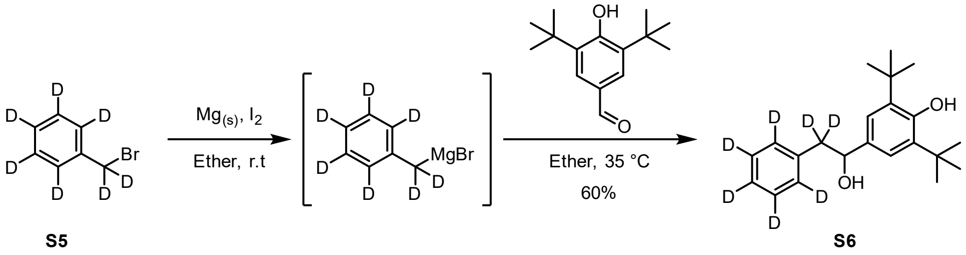

(S6) 2,6-di-*tert*-butyl-4-(1-hydroxy-2-(phenyl-d_5)ethyl-2,2-d_2)phenol[2]

^{1}H-NMR (400 MHz; $CDCl_3$): δ 7.13 (s, 2H), 5.18 (s, 1H), 4.81 (s, 1H), 1.86 (d, *J* = 2.3 Hz, 1H), 1.44 (s, 18H).

^{13}C NMR (101 MHz; $CDCl_3$): δ 153.4, 135.9, 134.5, 122.9, 75.9, 34.5, 30.4.

HRMS (EI, $[M-H_2O]^+$): m/z calcd for $C_{22}H_{21}O_1D_7$ 315.25795, found 315.25837.

^{1}H NMR, 400 MHz, Chloroform-*d*, Compound **S6**

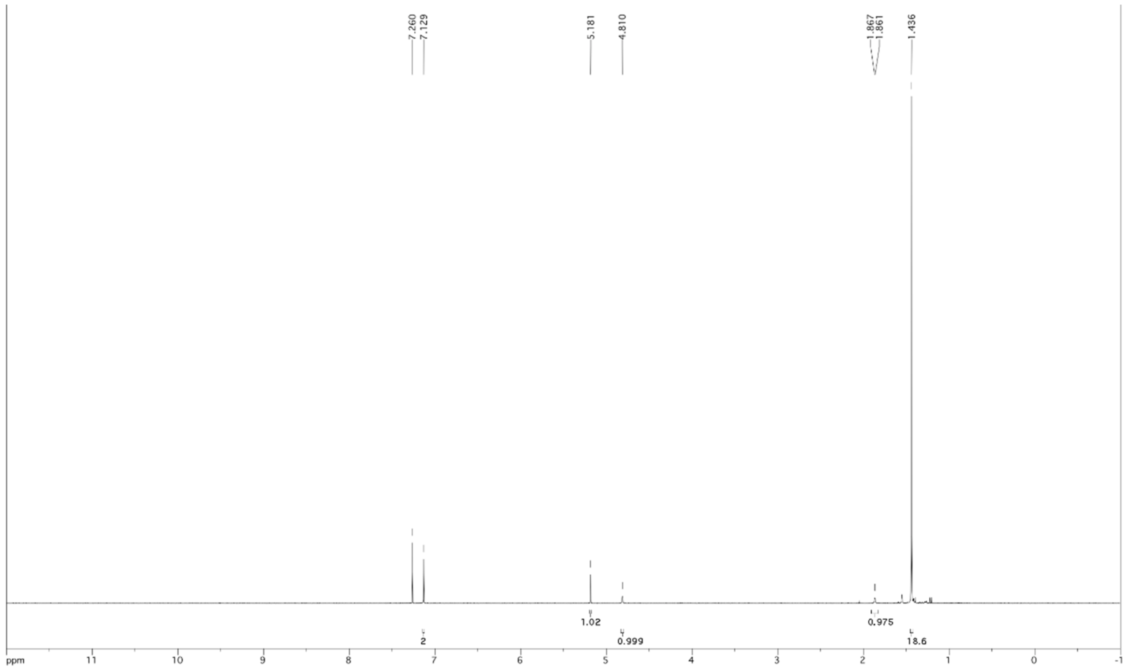

^{13}C NMR, 101 MHz, Chloroform-*d*, Compound **S6**

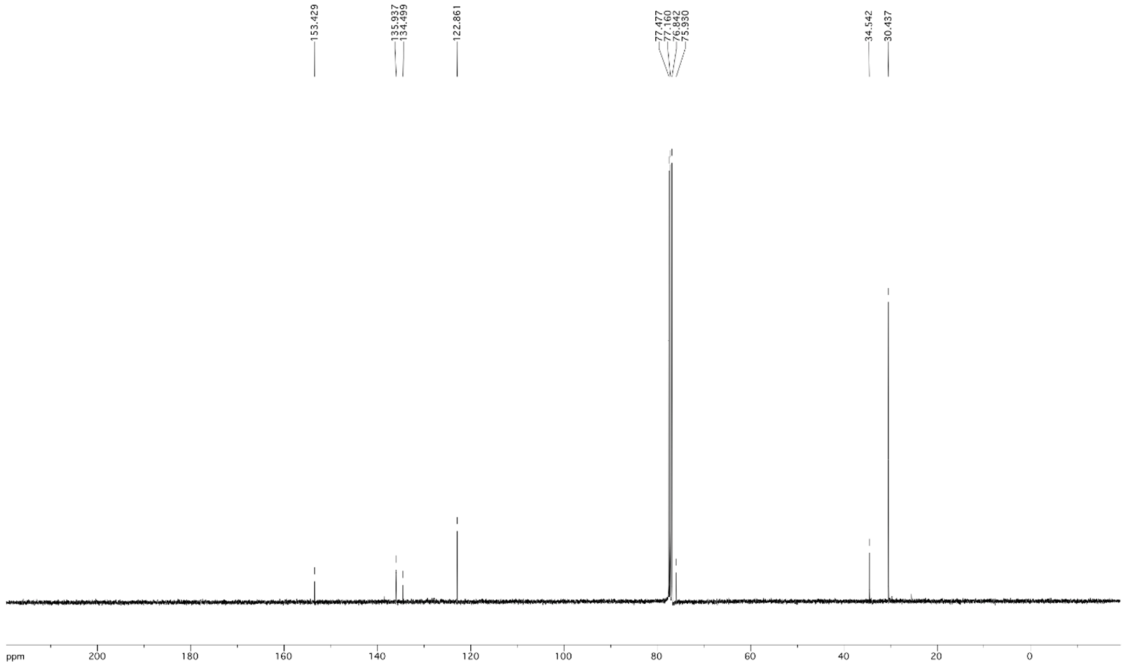

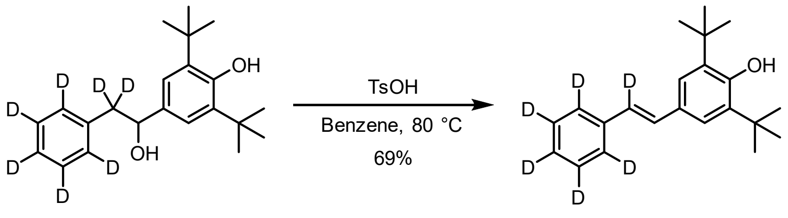

(1d-d_6) (*E*)-2,6-di-*tert*-butyl-4-(2-(phenyl-d_5)vinyl-2-*d*)phenol

^{1}H-NMR (400 MHz; $CDCl_3$): δ 7.35 (s, 2H), 7.07 (s, 1H), 5.28 (s, 1H), 1.48 (s, 18H).

^{13}C NMR (101 MHz; $CDCl_3$): δ 154.0, 136.3, 129.6, 128.8, 123.6, 34.5, 30.4.

HRMS (EI, [M+]): m/z calcd for $C_{22}H_{23}OD_6$ 314.25168, found 314.25172.

^{1}H NMR, 400 MHz, Chloroform-*d*, Compound **1d-d_6**

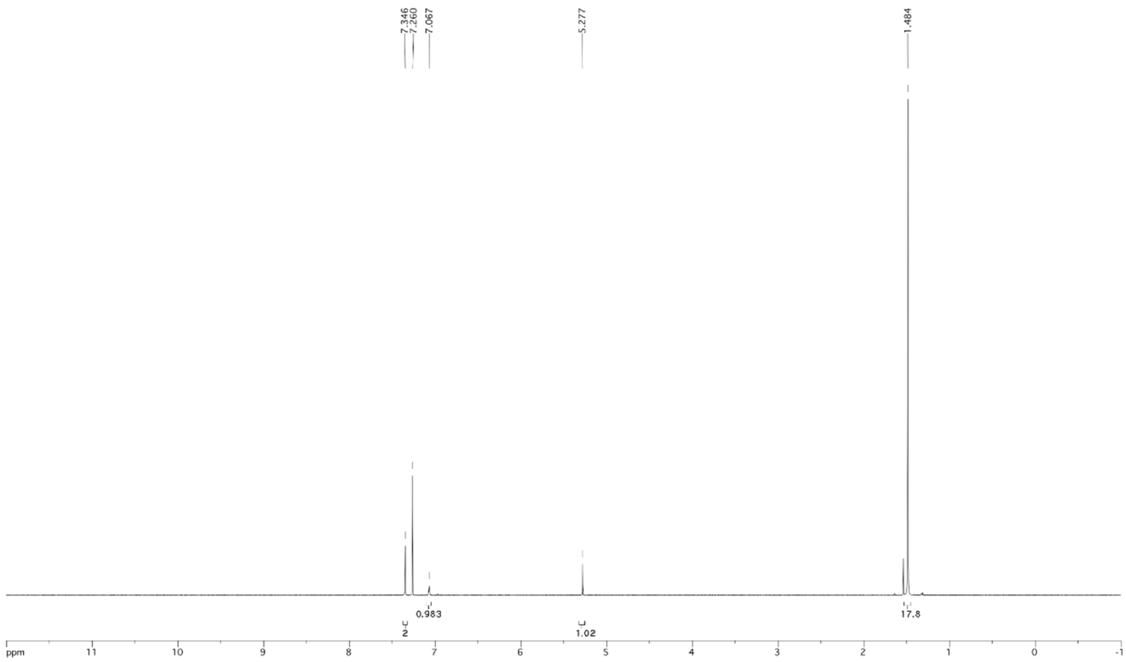

^{13}C NMR, 101 MHz, Chloroform-*d*, Compound **1d-*d*$_6$**

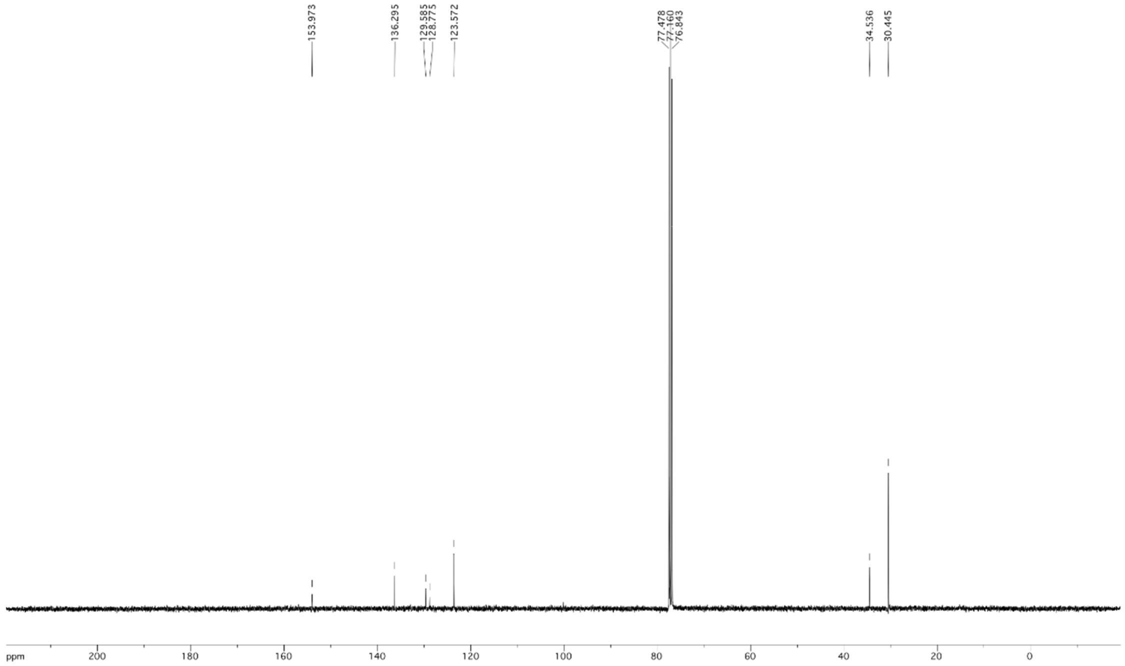

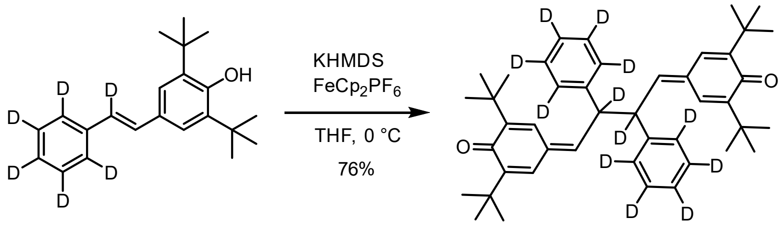

(2d-d_{12}) 4,4'-(2,3-bis(phenyl-d_5)butane-1,4-diylidene-2,3-d_2)bis(2,6-di-*tert*-butylcyclohexa-2,5-dien-1-one)[2]

^{1}H-NMR (600 MHz; CDCl$_3$): δ 7.15 (d, J = 2.4 Hz, 2H), 7.12 (d, J = 2.4 Hz, 2H), β-H's of quinone methides: 6.81 (minor diastereomer, d, J = 2.4 Hz, 2H), 6.69 (major diastereomer, d, J = 2.4 Hz, 2H), δ-H's of quinone methides: 6.49 (minor diastereomer, s, 2H), 6.38 (major diasteromer, s, 2H), ^{t}Bu signals: 1.25 (s, 18 H), 1.24 (s, 18H), 1.23 (s, 18 H), 1.21 (s, 18 H).

^{13}C NMR (151 MHz; CDCl$_3$): δ 186.6, 186.5, 149.1, 148.9, 147.6, 147.3, 145.4, 144.9, 140.9, 140.3, 134.7, 132.7, 132.0, 126.1, 125.9, 35.5, 35.5, 35.0, 34.9, 29.6, 29.6, 29.5.

HRMS (ESI, [M+Na]$^+$): m/z calcd for $C_{44}H_{42}O_2D_{12}Na$ 649.4775, found 649.4774.

^{1}H NMR, 600 MHz, Chloroform-*d*, Compound **2d-d_{12}**

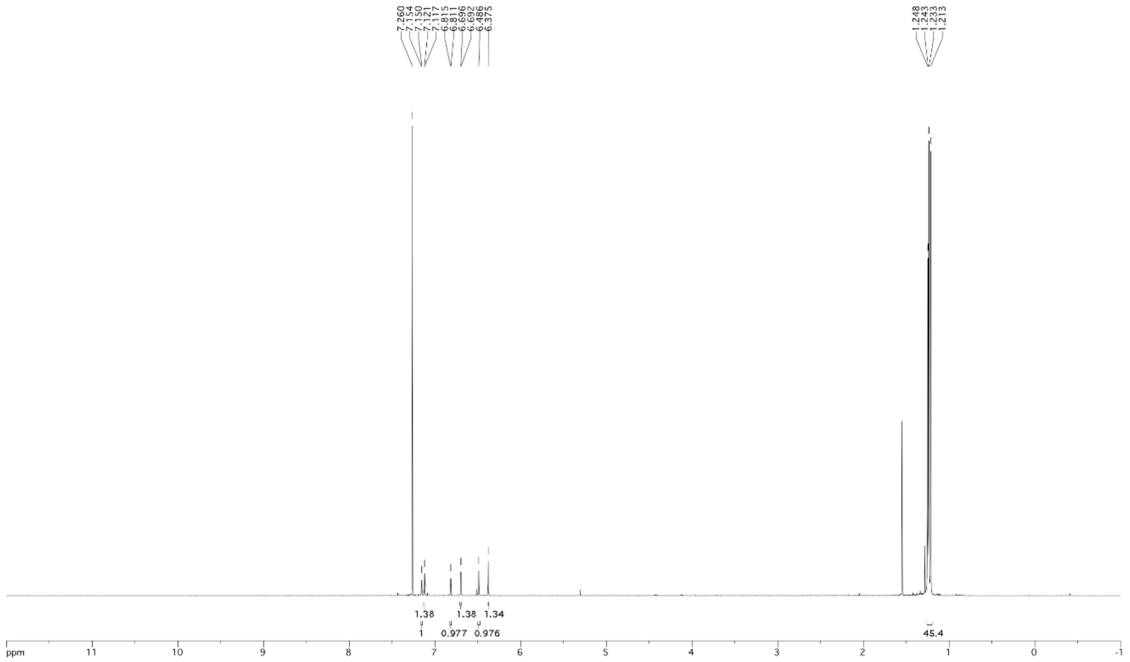

^{13}C NMR, 151 MHz, Chloroform-*d*, Compound **2d-*d*$_{12}$**

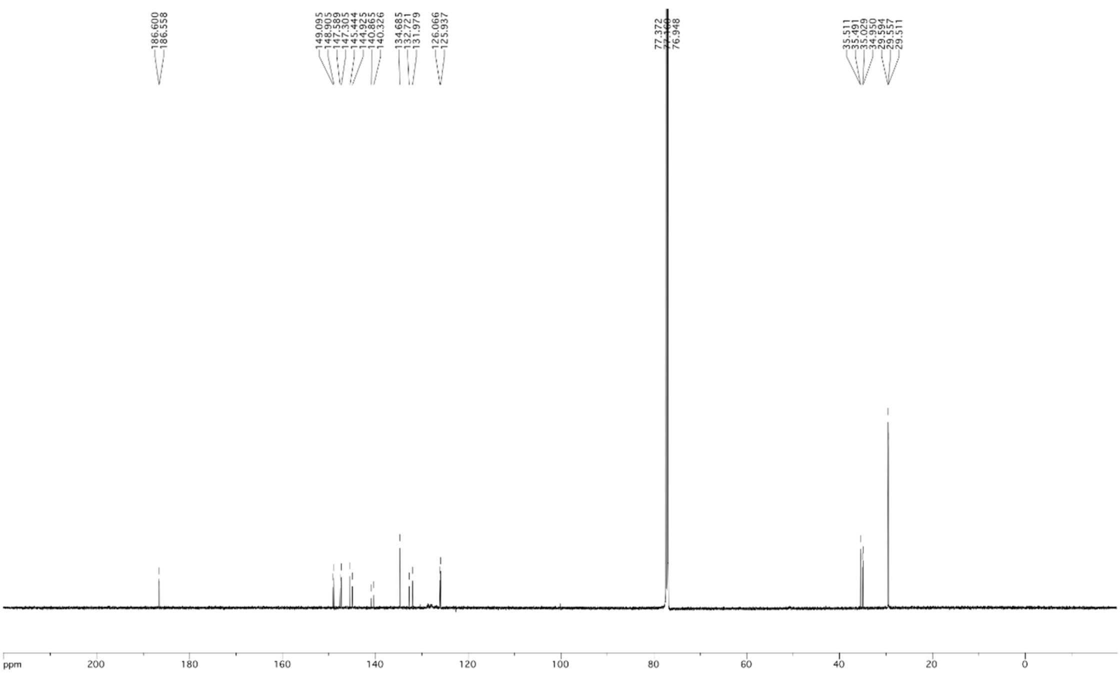

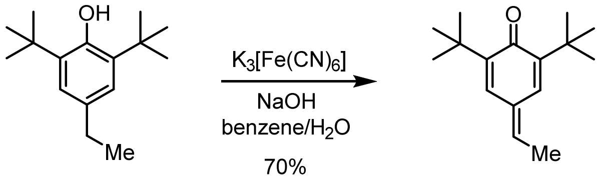

4a

(4a) 2,6-di-tert-butyl-4-ethylidenecyclohexa-2,5-dien-1-one[3]

^{1}H NMR (300 MHz; $CDCl_3$): δ 7.30 (d, J = 2.3 Hz, 1H), 6.85 (d, J = 2.3 Hz, 1H), 6.41 (q, J = 7.6 Hz, 1H), 2.13 (d, J = 7.8 Hz, 3H), 1.33 (s, 9H), 1.29 (s, 9H).

^{3}C NMR (151 MHz; $CDCl_3$): δ 186.8, 148.1, 146.4, 142.4, 134.8, 132.7, 125.7, 34.7, 29.47, 29.43, 14.9.

HRMS (EI, $[M]^+$): m/z calculated for $C_{16}H_{24}O$ 232.1827; found 232.1807.

^{1}H NMR, 300 MHz, Chloroform-*d*, Compound **4a**

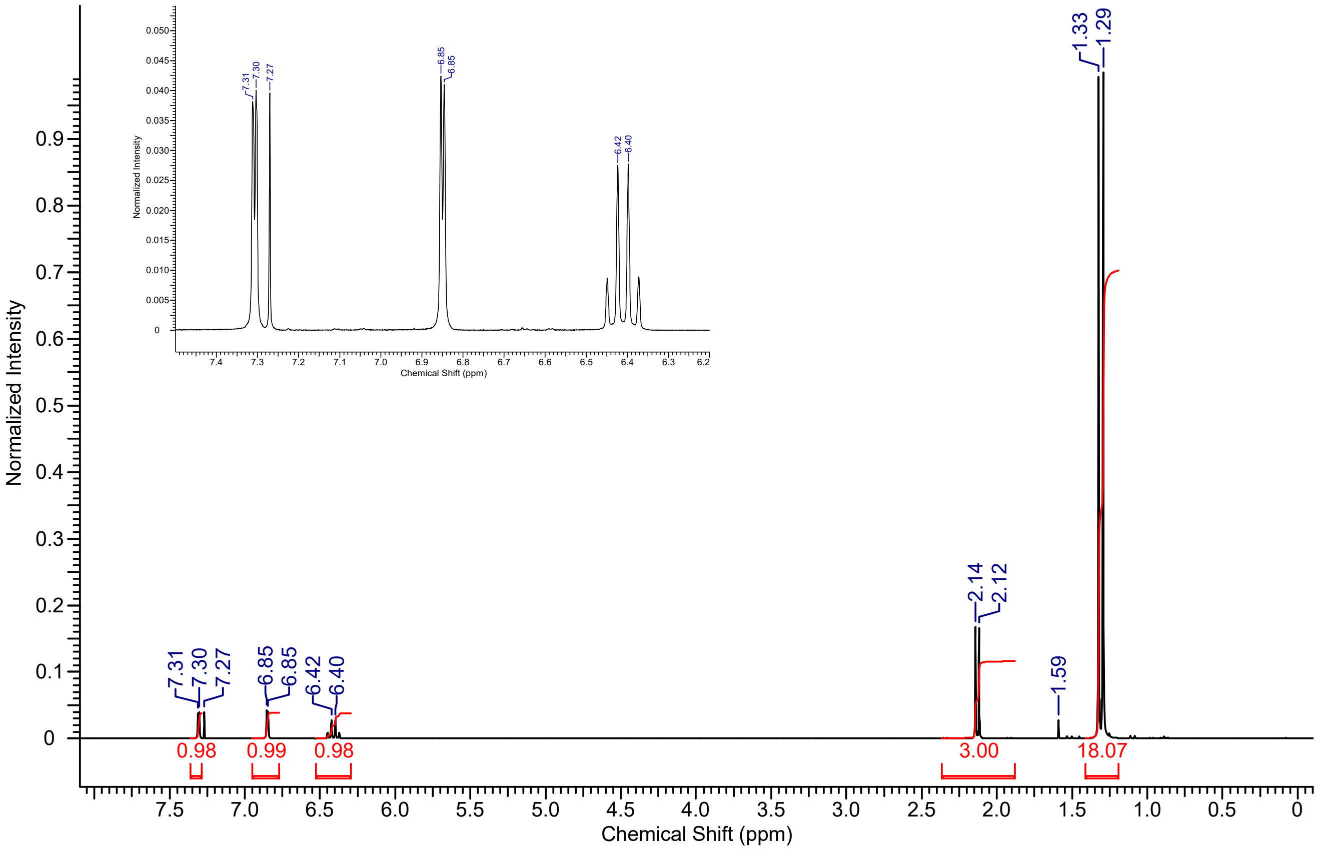

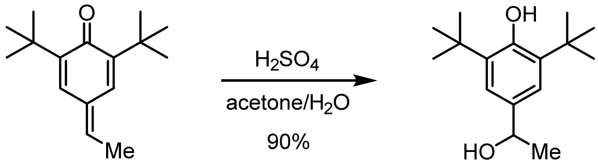

4a **4a-H_2O**

(4a-H_2O) 2,6-di-tert-butyl-4-(1-hydroxyethyl)phenol[4]

^{1}H NMR (300 MHz; $CDCl_3$): δ 7.22 (s, 2H), 5.21 (s, 1H), 4.84 (q, J = 6.5 Hz, 1H), 1.81 (bs, 1H), 1.52 (d, J = 6.5 Hz, 3H), 1.48 (s, 18H).

^{13}C NMR (101 MHz; $CDCl_3$): δ 153.2, 136.3, 135.9, 122.3, 70.9, 34.4, 30.3, 24.9.

^{1}H NMR, 300 MHz, Chloroform-*d*, Compound **4a-H_2O**

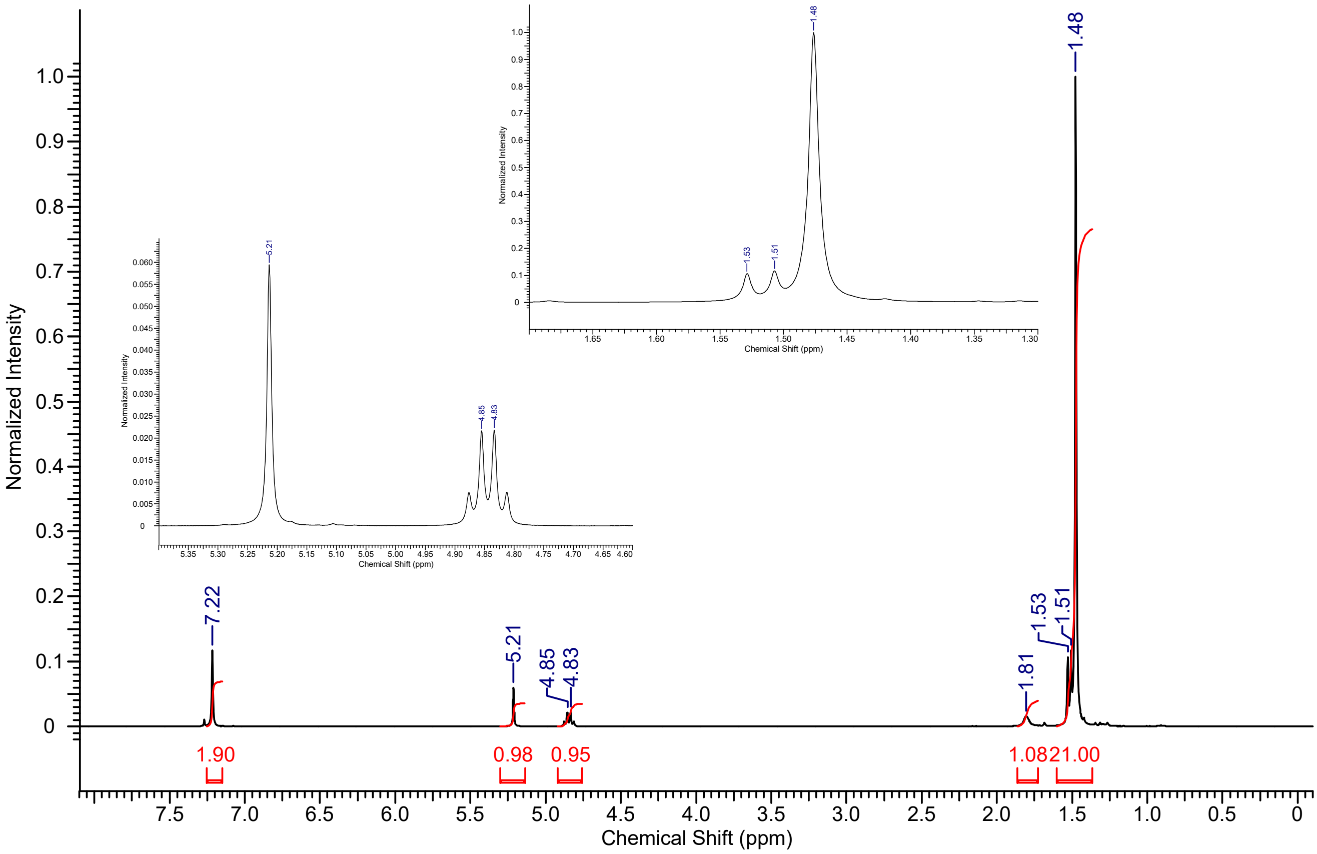

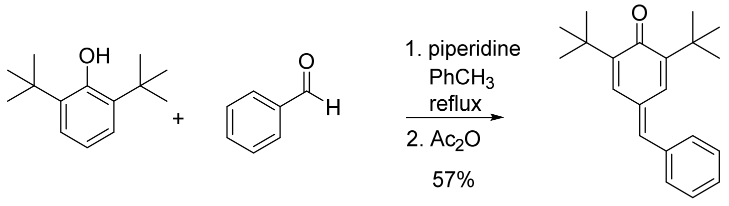

4b

(4b) 4-benzylidene-2,6-di-tert-butylcyclohexa-2,5-dien-1-one[5]

^{1}H NMR (300 MHz; $CDCl_3$): δ 7.54 (d, J = 2.3 Hz, 1H), 7.47 (m, 4H), 7.42 (m, 1H), 7.20 (s, 1H), 7.03 (d, J = 2.3 Hz, 1H), 1.35 (s, 9H), 1.32 (s, 9H).

^{13}C NMR (101 MHz; $CDCl_3$): δ 186.6, 149.4, 147.8, 142.4, 135.9, 135.1, 132.0, 130.3, 129.0, 128.8, 127.8, 35.4, 35.0, 29.53, 29.50.

HRMS (EI): calculated for $C_{21}H_{26}O$ 294.1984; found 294.1967.

^{1}H NMR, 300 MHz, Chloroform-*d*, Compound **4b**

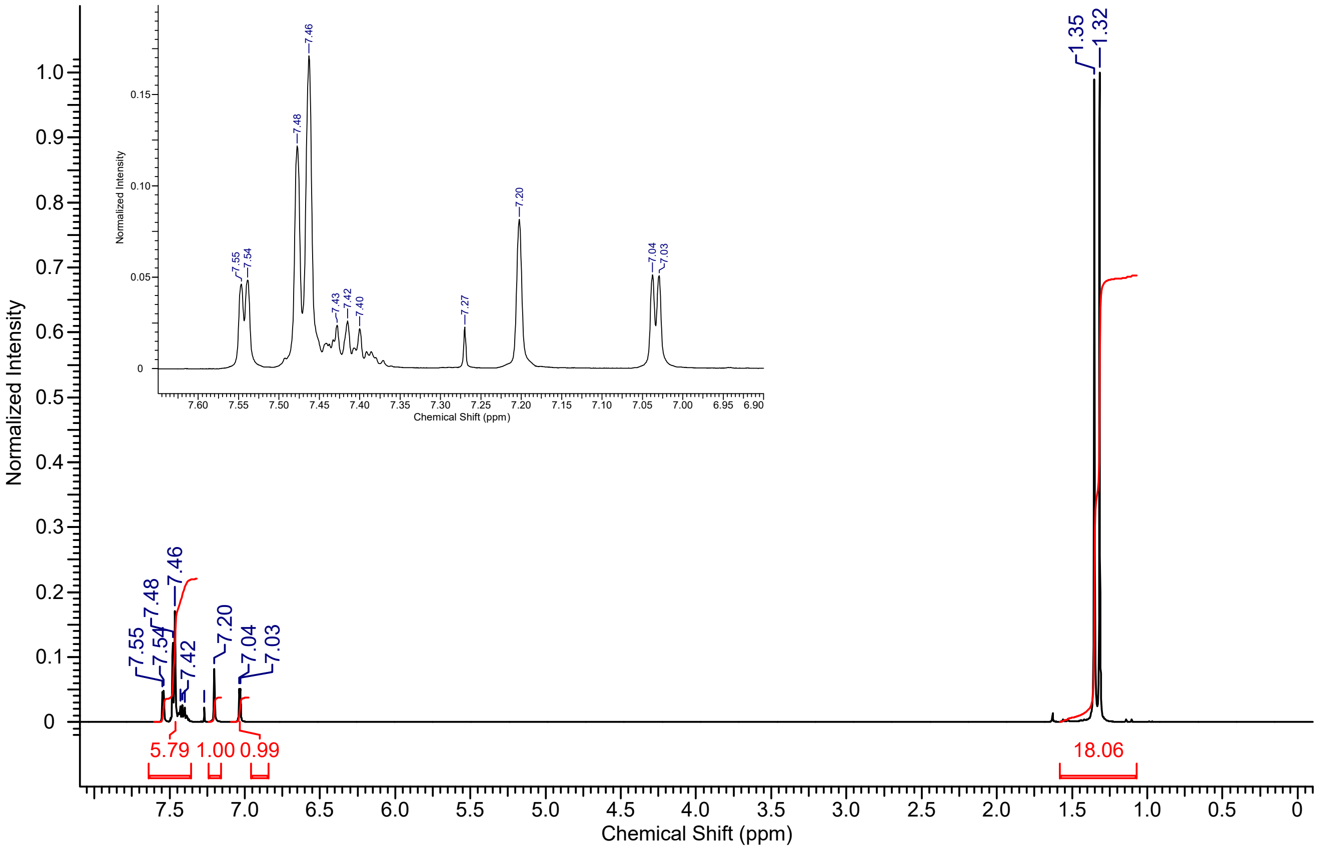

Computational Studies[6-10]

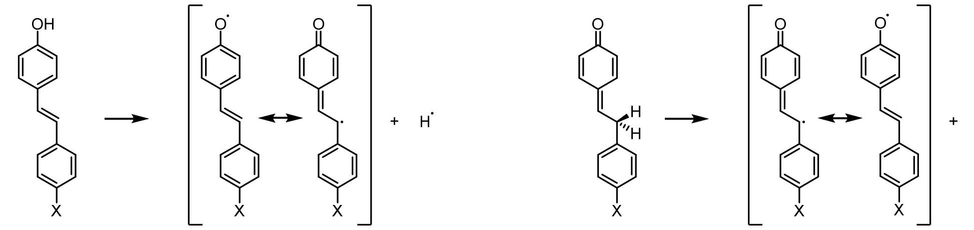

Scheme S1. O–H and C–H BDEs for substituted trans-4-hydroxystilbenes and their corresponding quinone-methide tautomers, respectively

Table S5. Summary of computed (CBS-QB3) O–H and C–H BDEs for substituted trans-4-hydroxystilbenes and their corresponding quinone-methide tautomers, respectively.

		CBS-QB3		
**Substituent**	**$\sigma^{+\,a}$**	**ΔH_{O-H}, $kcal{\cdot}mol^{-1}$**	**ΔH_{C-H}, $kcal{\cdot}mol^{-1}$**	**$\Delta\Delta H$, $kcal{\cdot}mol^{-1}$**
4-NMe_2	-1.7	80.4	65.9	14.5
4-NH_2	-1.3	80.8	66.4	14.4
3,4-dioxyl	-0.84	81.6	66.8	14.8
4-OH	-0.78	81.7	67.2	14.5
4-OMe	-0.78	81.6	67.1	14.5
4-SMe	-0.60	82.1	67.4	14.7
4-CH_3	-0.31	82.1	67.7	14.4
4-SH	-0.03	82.2	67.6	14.6
H	0	82.4	67.9	14.5
4-Cl	0.11	82.9	68.0	14.9
4-CO_2H	0.42	82.8	67.9	14.9
4-CF_3	0.61	83.2	68.3	14.9
4-NO_2	0.79	83.5	68.2	15.3

[a] Substituent constants are taken from Hansch, C.; Leo, A.; Taft, R. W. *Chem. Rev.* **1991**, *91*, 165-195. Those that did not appear there were estimated based on similar substituents (e.g. σ^+ for 3,4-dioxyl (-0.84) is derived from the sum of σ^+ for 4-OCH_2O (-0.68) and σ for 3-OCH_2O (-0.16)).

Table S6. Summary of computed O–H BDEs for substituted trans-4-hydroxystilbenes and the corresponding radical stabilization enthalpy (RSE), molecule stabilization enthalpy (MSE), and total stabilization effect (TSE).

		B3LYP/CBSB7				CBS-QB3			
**Substituent**	**σ^+**	**ΔH_{O-H}, $kcal{\cdot}mol^{-1}$**	**RSE, $kcal{\cdot}mol^{-1}$**	**MSE, $kcal{\cdot}mol^{-1}$**	**TSE, $kcal{\cdot}mol^{-1}$**	**ΔH_{O-H}, $kcal{\cdot}mol^{-1}$**	**RSE, $kcal{\cdot}mol^{-1}$**	**MSE, $kcal{\cdot}mol^{-1}$**	**TSE, $kcal{\cdot}mol^{-1}$**
4-NMe_2	-1.7	74.6	-2.04	0.30	-2.34	80.4	-1.96	0.02	-1.99
4-NH_2	-1.3	76.1	-1.65	0.27	-1.92	80.8	-1.48	0.11	-1.59
3,4-dioxyl	-0.84	76.1	-0.72	0.11	-0.82	81.6	-0.83	-0.01	-0.83
4-OH	-0.78	76.0	-0.72	0.14	-0.86	81.7	-0.73	0.00	-0.72
4-OMe	-0.78	75.9	-0.87	0.15	-1.02	81.6	-0.81	0.02	-0.84
4-SMe	-0.60	76.2	-0.64	0.06	-0.71	82.1	-0.28	0.00	-0.83
4-CH_3	-0.31	76.5	-0.35	0.06	-0.41	82.1	-0.29	0.11	-0.29
4-SH	-0.03	76.6	-0.29	0.01	-0.30	82.2	-0.11	0.11	-0.22
H	0	76.9	0.00	0.00	0.00	82.4	0.00	0.00	0.00
4-Cl	0.11	77.4	0.40	-0.10	0.50	82.9	0.51	0.03	0.47
4-CO_2H	0.42	77.9	0.76	-0.23	0.99	82.8	0.37	-0.07	0.44
4-CF_3	0.61	78.1	0.97	-0.24	1.21	83.2	0.84	0.01	0.83
4-NO_2	0.79	78.8	1.46	-0.43	1.89	83.5	1.04	-0.10	1.15

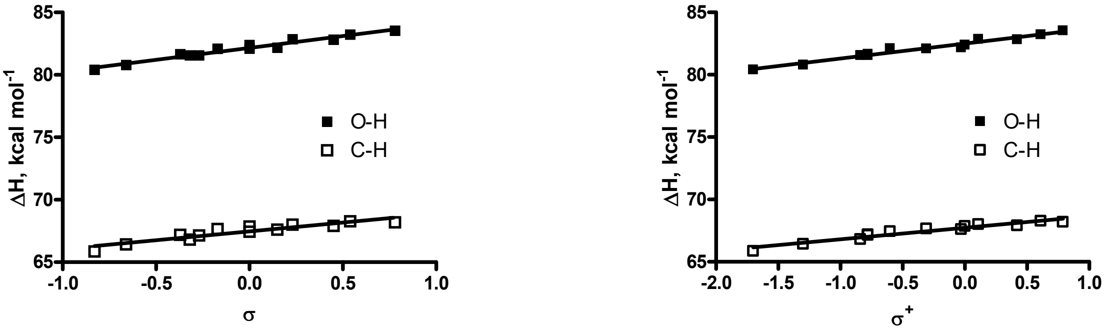

Figure S74. Correlation of computed O–H (■) and C–H (□) BDEs with σ and σ^+ for substituted trans-4-hydroxystilbenes and their corresponding quinone-methide tautomers. The relationships between BDE and σ or σ^+ for O–H are $\rho_{O\text{–}H}$ = 1.9 ± 0.1, r^2 = 0.9584 and $\rho^+_{O\text{–}H}$ = 1.20 ± 0.07, r^2 = 0.9658; those for C–H are $\rho_{C\text{–}H}$ = 1.4 ± 0.2, r^2 = 0.8566 and $\rho^+_{C\text{–}H}$ = 0.93 ± 0.08, r^2 = 0.9243. Note: the C–H BDE data may contain two relationships for electron-donating and electron-withdrawing groups. If the $\sigma^{(+)}$ > 0 points are not included in the fit, the slopes are the same within error for O–H and C–H $\rho_{O\text{–}H}$ = 2.4 ± 0.2 and $\rho_{C\text{–}H}$ = 2.4 ± 0.3; $\rho^+_{O\text{–}H}$ = 1.2 ± 0.2 and $\rho^+_{C\text{–}H}$ = 1.1 ± 0.1.

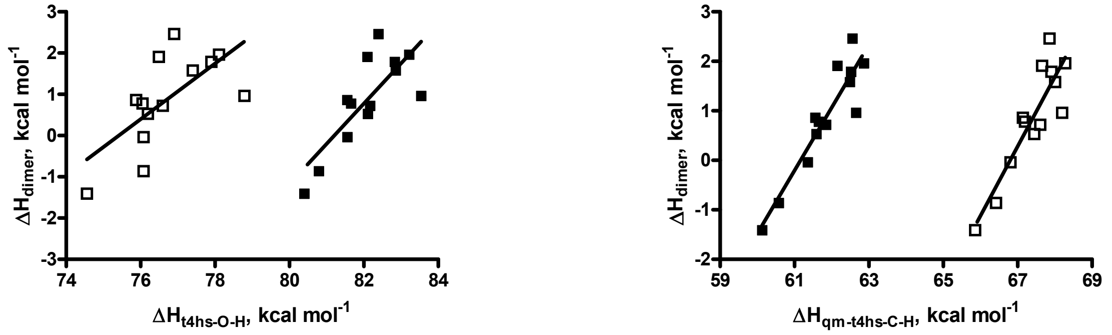

Figure S75. Correlation of computed $\Delta H_{C\text{–}C}$ values with $\Delta H_{O\text{–}H}$ or $\Delta H_{C\text{–}H}$ for the corresponding trans-4-hydroxystilbene or quinone methide. The relationships between $\Delta H_{C\text{–}C}$ and $\Delta H_{O\text{–}H}$ exhibit gradient_{DFT} (■) = 1.0 ± 0.2, r^2 = 0.6174 and gradient_{CBS} (□) = 0.7 ± 0.2, r^2 = 0.4499. The relationships between $\Delta H_{C\text{–}C}$ and $\Delta H_{C\text{–}H}$ exhibit gradient_{DFT} (■) = 1.3 ± 0.2, r^2 = 0.8610; gradient_{CBS} (□) = 1.4 ± 0.2, r^2 = 0.7936. Note: only the ΔH_{t4hs} and $\Delta H_{qm\text{-}t4hs}$ values are CBS-QB3; in both cases, ΔH_{dimer} values are DFT.

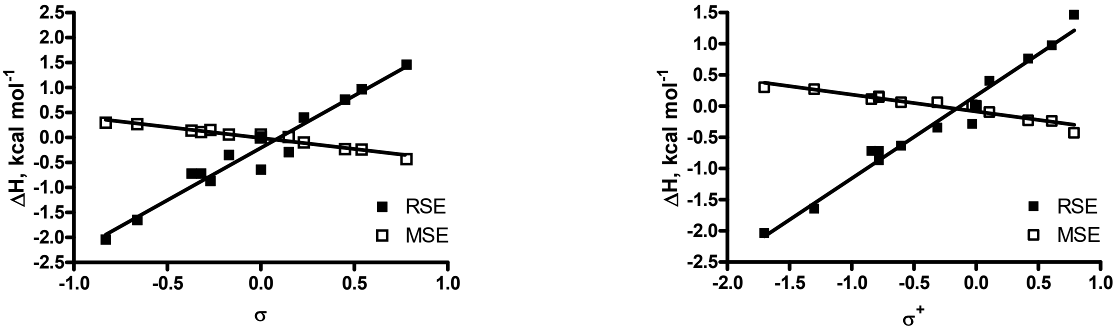

Figure S76. Correlation of DFT-calculated (B3LYP/CBSB7) radical (■) and molecule (□) stabilization enthalpies in substituted trans-4-hydroxystilbenes with σ and σ^+. These relationships exhibit ρ_{RSE} = 2.1 ± 0.1, r^2 = 0.9529 and ρ_{MSE} = -0.44 ± 0.03, r^2 = 0.9539 as well as ρ^+_{RSE} = 1.33 ± 0.07, r^2 = 0.9696 and ρ^+_{MSE} = -0.27 ± 0.02, r^2 = 0.9154.

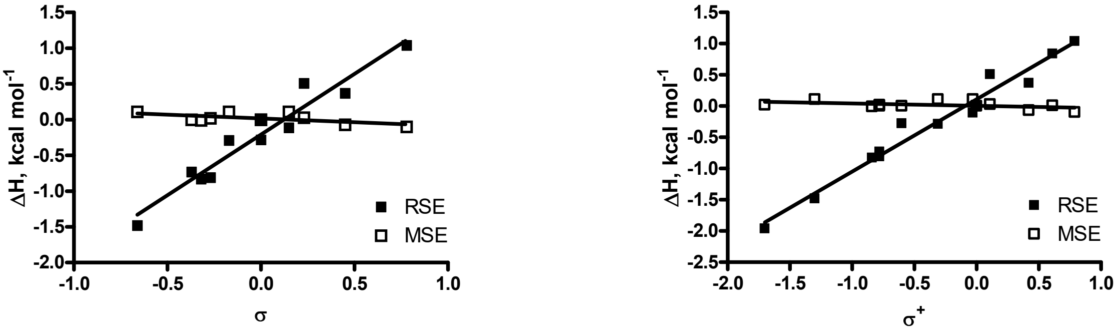

Figure S77. Correlation of CBS QB3-calculated radical (■) and molecule (□) stabilization enthalpies in substituted trans-4-hydroxystilbenes with σ and σ$^+$. These relationships exhibit ρ_{RSE} = 1.7 ± 0.1, r^2 = 0.9364 and ρ_{MSE} = -0.11 ± 0.04, r^2 = 0.3825 as well as ρ^+_{RSE} = 1.16 ± 0.06, r^2 = 0.9685 and ρ^+_{MSE} = -0.04 ± 0.02, r^2 = 0.1805.

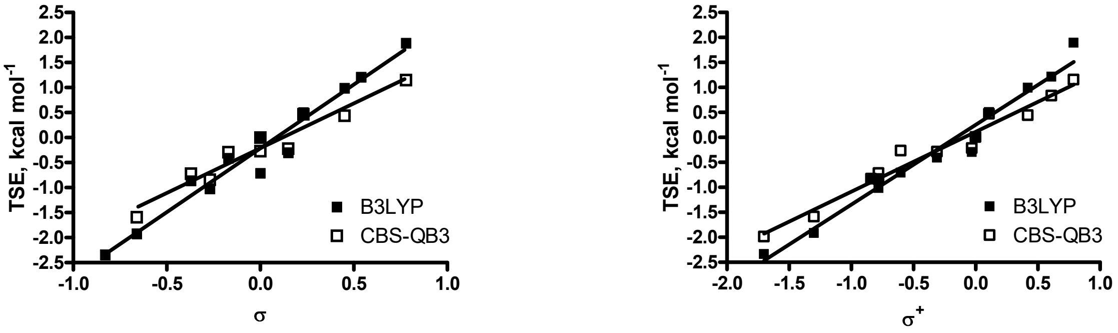

Figure S78. Correlation of B3LYP/CBSB7 (■)- and CBS QB3 (□)-calculated total stabilization effect in substituted trans-4-hydroxystilbenes with σ and σ$^+$. These relationships exhibit ρ_{B3LYP} = 2.6 ± 0.2, r^2 = 0.9591 and $\rho_{CBS\text{-}QB3}$ = 1.8 ± 0.2, r^2 = 0.9332 as well as ρ^+_{B3LYP} = 1.60 ± 0.09, r^2 = 0.9651 and $\rho^+_{CBS\text{-}QB3}$ = 1.20 ± 0.07, r^2 = 0.9651.

Table S7. Summary of ρ^+ values for Y–H (Y = O, C) bond dissociation

Y	ρ^+_{RSE}	ρ^+_{MSE}	ρ^+_{TSE}
O (phenols)[a]	4.5 ± 0.2	-1.6 ± 0.2	6.1 ± 0.2
C (toluenes)[a]	[b]	-0.8 ± 0.1	[b]
O,C (trans-4-hydroxystilbenes)	1.16 ± 0.06	-0.04 ± 0.02	1.20 ± 0.07

[a] Data from Pratt, D. A.; Dilabio, G. A.; Valgimigli, L.; Franco Pedulli, G.; Ingold, K. U. *J. Am. Chem. Soc.* **2002**, *124*, 11085–11092.

[b] Not a single linear correlation; the data have a very small sensitivity to σ$^+$.

Table S8. Summary of enthalpies (ΔH‡) and free energies (ΔG‡) of activation and corresponding computed second order rate constants (k_{add}) at 37 °C for the addition of alkyl peroxyl radical (ROO•) to or hydrogen atom transfer (HAT) from **4a** (R = H) in the gas phase.

	B3LYP/CBSB7			CBS-QB3		
ROO•	ΔH‡, kcal·mol^{-1}	ΔG‡, kcal·mol^{-1}	k_{add}, M^{-1}·s^{-1} (37 °C)	ΔH‡, kcal·mol^{-1}	ΔG‡, kcal·mol^{-1}	k_{add}, M^{-1}·s^{-1} (37 °C)
R = Me	2.4	13.0	1×10^5	-0.8	9.7	2×10^7
R = Et	2.5	13.2	7×10^4	-1.4	9.3	4×10^7
R = ^{i}Pr	3.0	13.7	4×10^4	-1.6	9.0	7×10^7
R = ^{t}Bu	4.8	15.9	1×10^3	-0.3	10.7	4×10^6

Table S9. Summary of enthalpies (ΔH‡) and free energies (ΔG‡) of activation and corresponding computed second order rate constants (k_{add}) at 37 °C for the addition of alkyl peroxyl radical (ROO•) to **4a** (R = H) in the presence of a self-consistent reaction field (SCRF) for chlorobenzene.

	DFT (SCRF = PhCl)			CBS-QB3 (SCRF = PhCl)		
ROO•	ΔH‡, kcal·mol^{-1}	ΔG‡, kcal·mol^{-1}	k_{add}, M^{-1}·s^{-1} (37 °C)	ΔH‡, kcal·mol^{-1}	ΔG‡, kcal·mol^{-1}	k_{add}, M^{-1}·s^{-1} (37 °C)
R = Me	4.0	14.5	1×10^4	0.7	11.1	2×10^6
R = Et						
R = ^{i}Pr						
R = ^{t}Bu	6.3	17.2	1×10^2	1.2	12.1	5×10^5

Table S10. Summary of enthalpies (ΔH, ΔH‡) and free energies (ΔG, ΔG‡) and corresponding computed second order rate constants (k_{add}) at 37 °C for the addition of methyl peroxyl radical to **4** (R = H or ^{t}Bu) in the gas phase.

	B3LYP/CBSB7					CBS-QB3				
QM	ΔH‡, kcal·mol^{-1}	ΔG‡, kcal·mol^{-1}	k_{add}, M^{-1}·s^{-1} (37 °C)	ΔH, kcal·mol^{-1}	ΔG, kcal·mol^{-1}	ΔH‡, kcal·mol^{-1}	ΔG‡, kcal·mol^{-1}	k_{add}, M^{-1}·s^{-1} (37 °C)	ΔH, kcal·mol^{-1}	ΔG, kcal·mol^{-1}
**4d**	1.7	11.8	8×10^5	-19.0	-8.5	-2.5	7.6	7×10^8	-29.9	-19.5
**4a**	2.4	13.0	1×10^5	-19.3	-8.5	-0.8	9.8	2×10^7	-28.2	-17.4
**4a-*d***	2.3	12.9	1×10^5	ND	ND	-0.9	9.7	3×10^7	ND	ND
^{t}Bu$_2$-**4a**	2.5	14.1	2×10^4	-19.6	-7.9	1.6	10.0	2×10^7	-28.9	-17.3
**4** (Y=Z=H)	3.1	14.2	2×10^4	-19.3	-8.6	1.2	12.3	3×10^5	-26.0	-15.3
**4b**	5.6	17.3	1×10^2	-14.7	-2.5	2.8	14.6	9×10^3	-26.1	-14.0
**4e**	7.0	19.7	2×10^0	-9.1	3.5	ND	ND	ND	ND	ND
**4** (Y=Z=CN)	10.5	22.4	3×10^{-2}	-4.9	7.0	3.4	15.2	3×10^3	-16.9	-5.0

Table S11. Summary of enthalpies (ΔH‡) and free energies (ΔG‡) of activation and corresponding computed second order rate constants (k_{add}) at 37 °C for the addition of methyl peroxyl radical to **4a** in the gas phase or presence of a self-consistent reaction field (SCRF) for chlorobenzene.

	B3LYP/CBSB7			CBS-QB3			DKIE	
QM	ΔH‡, kcal·mol^{-1}	ΔG‡, kcal·mol^{-1}	k_{add}, M^{-1}·s^{-1} (37 °C)	ΔH‡, kcal·mol^{-1}	ΔG‡, kcal·mol^{-1}	k_{add}, M^{-1}·s^{-1} (37 °C)	k_H/k_D DFT (37 °C)	k_H/k_D CBS (37 °C)
**4a**	2.4	13.0	1×10^5	-0.8	9.8	2×10^7	1	1
**4a-*d***	2.3	12.9	1×10^5	-0.9	9.7	3×10^7		

Table S12. Summary of enthalpies (ΔH, ΔH‡) and free energies (ΔG, ΔG‡) and corresponding computed second order rate constants (k_{HAT}) at 37 °C for HAT from **4a** or **4e** (R = H) in the gas phase.

	B3LYP/CBSB7					CBS-QB3					DKIE	
QM	ΔH‡, kcal· mol^{-1}	ΔG‡, kcal· mol^{-1}	k_{HAT}, M^{-1}·s^{-1} (37 °C)	ΔH, kcal· mol^{-1}	ΔG, kcal· mol^{-1}	ΔH‡, kcal· mol^{-1}	ΔG‡, kcal· mol^{-1}	k_{HAT}, M^{-1}·s^{-1} (37 °C)	ΔH, kcal· mol^{-1}	ΔG, kcal· mol^{-1}	k_H/k_D DFT (37 °C)	k_H/k_D CBS (37 °C)
**4a**	7.7	18.2	20	-8.0	-8.9	6.8	17.3	100	-13.3	-14.2	4	5
**4a-*d***	8.6	19.1	5			7.7	18.2	20				
**^{t}Bu$_2$-4a**	7.7	19.0	6	-8.1	-8.2	6.0	17.4	90	-13.6	-13.7		
**4c**	9.2	20.9	0.3	-13.7	-13.3	7.5	19.1	6	-15.7	-15.4	6	6
**4c-*d***	10.3	21.9	0.05			8.5	20.2	1				

Table S13. Summary of enthalpies (ΔH‡) and free energies (ΔG‡) of activation and corresponding computed second order rate constants (k_{add}) at 37 °C for the addition of methyl peroxyl radical to **6** (R = H) or **4a** (R = H) in the gas phase determined using DFT, dispersion-corrected DFT (GD3), or CBS-QB3.

	B3LYP/CBSB7			B3LYP/CBSB7 + GD3			CBS-QB3		
QMD	ΔH‡, kcal· mol^{-1}	ΔG‡, kcal· mol^{-1}	k_{add}, M^{-1}·s^{-1} (37 °C)	ΔH‡, kcal· mol^{-1}	ΔG‡, kcal· mol^{-1}	k_{add}, M^{-1}·s^{-1} (37 °C)	ΔH‡, kcal· mol^{-1}	ΔG‡, kcal· mol^{-1}	k_{add}, M^{-1}·s^{-1} (37 °C)
**6**-meso-extended	6.1	18.0	3×10^1	-0.7	12.1	5×10^5	1.2	13.0	1×10^5
**6**-meso-compressed	4.9	17.0	2×10^2	-2.2	9.9	2×10^7	0.5	12.5	2×10^5
**6**-(S,S)-extended	5.5	17.6	6×10^1	-1.3	11.4	2×10^6			
**6**-(S,S)-compressed	5.8	17.5	7×10^1	-2.8	11.0	3×10^6			
**4a**	2.4	13.0	1×10^5	-2.6	8.4	2×10^8	-0.8	9.8	2×10^7

Table S14. Summary of enthalpies (ΔH, ΔH‡) and free energies (ΔG, ΔG‡) and corresponding computed second order rate constant ford addition (k_{add}) at 37 °C for the addition of methyl peroxyl radical to **2d** (R = H) along with those of the subsequent 5-exo-trig cyclization in the gas phase determined using DFT.

	Addition					Cyclization			
QMD	ΔH‡, kcal· mol^{-1}	ΔG‡, kcal· mol^{-1}	k_{add}, M^{-1}·s^{-1} (37 °C)	ΔH, kcal· mol^{-1}	ΔG, kcal· mol^{-1}	ΔH‡, kcal· mol^{-1}	ΔG‡, kcal· mol^{-1}	ΔH, kcal· mol^{-1}	ΔG, kcal· mol^{-1}
**2d**-meso-extended	3.0	16.0	9×10^2	-17.7	-4.6	-2.9	13.4	-16.0	0.2

Computed structures of 1d, 5a, 3d

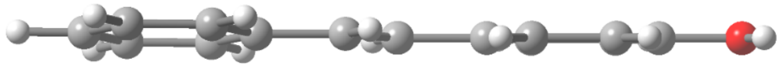

Figure S79. Computed (B3LYP/CBSB7) structure of **1d** (R = H). The stilbenoid C=C is twisted away from planarity with the phenol by 3°; the resorcinol Ph is twisted away from planarity with the C=C by 4°.

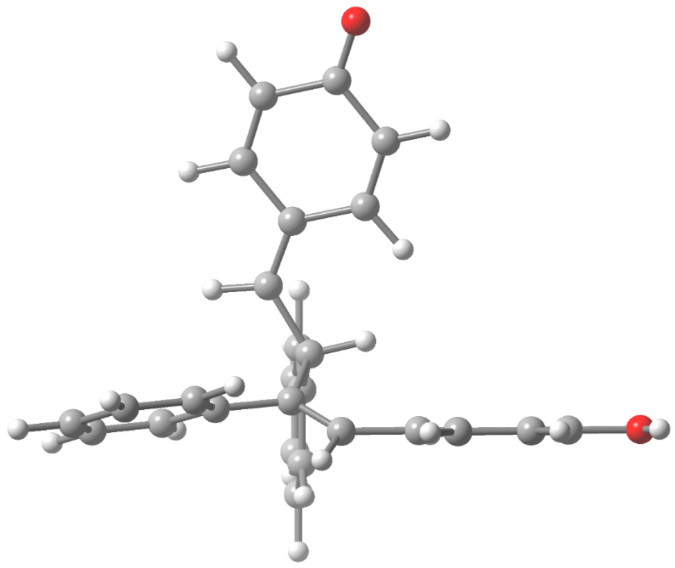

Figure S80. Computed (B3LYP/CBSB7) structure of **5a** (R = H). The stilbenoid C=C is twisted away from planarity with the phenol by 36°; the resorcinol Ph on the left is twisted away from planarity with the C=C by 43°.

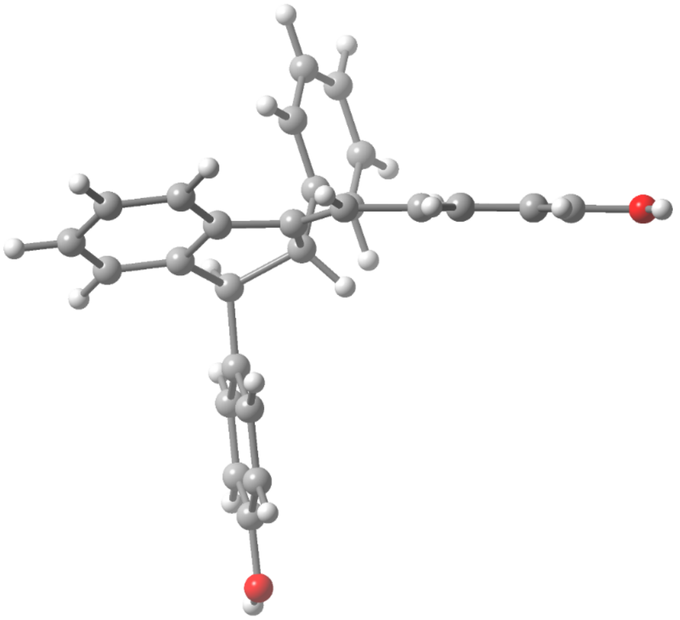

Figure S81. Computed (B3LYP/CBSB7) structure of **3c** (R = H). The stilbenoid C=C is twisted away from planarity with the phenol by 17°; the resorcinol Ph is twisted away from planarity with the phenol by 32°.

Cartesian Coordinates

Computational Data[6]

Optimized Gaussian Structures: DFT (B3LYP/CBSB7),[7] Dispersion-Corrected DFT,[8] and CBS-QB3[9] (Hartree)

<u>Peroxyls</u>

Methyl peroxyl radical

$^{\bullet}OOCH_3$

DFT Enthalpy = -190.224254 DFT Free Energy = -190.254744

CBS-QB3 Enthalpy = -189.954731 CBS-QB3 Free Energy = -189.985243

0 2

C	1.09605900	-0.18318300	0.00000000
H	1.87467700	0.57860700	-0.00001500
H	1.14885000	-0.80070400	0.89699800
H	1.14883700	-0.80072700	-0.89698300
O	-0.15733600	0.54388600	0.00000000
O	-1.18625300	-0.27864500	0.00000000

SCRF = (CPCM, solvent=chlorobenzene)

DFT Enthalpy = -190.228253 DFT Free Energy = -190.258769

CBS-QB3 Enthalpy = -189.958560 CBS-QB3 Free Energy = -189.989099

0 2

C	1.10140100	-0.18287500	-0.00000100
H	1.87355100	0.58411900	-0.00001400
H	1.15391300	-0.79719200	0.89828500
H	1.15390500	-0.79721400	-0.89827200
O	-0.15898600	0.54103200	-0.00000400
O	-1.18973600	-0.27759000	0.00000400

EmpiricalDispersion = GD3

DFT Enthalpy = -190.226031 DFT Free Energy = -190.256454

0 2

C	1.09634800	-0.18305800	-0.00000100
H	1.87629900	0.57778900	-0.00001300
H	1.14811500	-0.80135800	0.89699600
H	1.14810700	-0.80138000	-0.89698300
O	-0.15820200	0.54476300	-0.00000400
O	-1.18562400	-0.27935100	0.00000500

Ethyl peroxyl radical

$^{\bullet}OOCH_2CH_3$

DFT Enthalpy = -229.525047 DFT Free Energy = -229.559316

CBS-QB3 Enthalpy = -229.184415 CBS-QB3 Free Energy = -229.21873

0 2

C	0.41524900	0.53851500	-0.00001400
H	0.26541900	1.15279000	-0.89016500
H	0.26538500	1.15280500	0.89011900
O	-0.63451900	-0.47984700	-0.00002700
O	-1.83540100	0.05936100	0.00002300
C	1.74626600	-0.18275900	0.00001500
H	2.55953100	0.54693100	0.00001500
H	1.84995200	-0.81156800	0.88656800
H	1.84997800	-0.81159700	-0.88651300

iso-Propyl peroxyl radical

$^{\bullet}OOCH(CH_3)_2$

DFT Enthalpy = -268.826494 DFT Free Energy = -268.863611

CBS-QB3 Enthalpy = -268.416419 CBS-QB3 Free Energy = -268.453609

0 2

C	0.37278500	0.02197100	0.34197000
H	0.15576800	-0.03594900	1.41139300
O	-0.77878600	-0.62632700	-0.32204500
O	-1.92887100	-0.16111500	0.11640800
C	1.57370700	-0.83104600	-0.02521500
H	2.46921700	-0.44101800	0.46409000
H	1.42922100	-1.86530900	0.29285200
H	1.74007300	-0.81943700	-1.10524600
C	0.45774800	1.47017600	-0.11018300
H	-0.47743400	1.98595600	0.11139900
H	1.26899100	1.97992100	0.41590100
H	0.64998000	1.52876100	-1.18473400

tert-Butyl peroxyl radical

$^{\bullet}OOC(CH_3)_3$

DFT Enthalpy = -308.126181 DFT Free Energy = -308.165984

CBS-QB3 Enthalpy = -307.648937 CBS-QB3 Free Energy = -307.688842

0 2

C	-0.36949300	0.00000100	0.04891200

O	0.83360400	-0.00002300	-0.85136200
O	1.97204300	-0.00000500	-0.19656400
C	-1.52418900	-0.00004200	-0.94505200
H	-2.47632300	-0.00005300	-0.40935000
H	-1.48664100	0.88630900	-1.58199600
H	-1.48660200	-0.88642400	-1.58195300
C	-0.31660600	-1.26908600	0.89261800
H	0.59155400	-1.28174100	1.49633200
H	-1.18150300	-1.31095200	1.55926700
H	-0.32709600	-2.15672400	0.25591900
C	-0.31662200	1.26915200	0.89252700
H	-0.32708500	2.15673900	0.25575700
H	-1.18154000	1.31107700	1.55914500
H	0.59152000	1.28184700	1.49626700

SCRF = (CPCM, solvent=chlorobenzene)

DFT Enthalpy = -308.129982 DFT Free Energy = -308.169855

CBS-QB3 Enthalpy = -307.652638 CBS-QB3 Free Energy = -307.692613

0 2

C	-0.37344100	0.00000000	0.05037600
O	0.84233100	-0.00000100	-0.84559300
O	1.97955200	0.00000000	-0.19015800
C	-1.51752800	0.00001700	-0.95472400
H	-2.47181500	0.00001800	-0.42382400
H	-1.47697400	0.88790600	-1.58937300
H	-1.47698500	-0.88785900	-1.58939100
C	-0.32475800	-1.27003600	0.89138700
H	0.57153300	-1.28050500	1.51280800
H	-1.20054100	-1.31346900	1.54273800
H	-0.32426600	-2.15669000	0.25335800
C	-0.32474100	1.27002000	0.89141100
H	-0.32423700	2.15668600	0.25339800
H	-1.20052300	1.31345200	1.54276300
H	0.57155100	1.28046500	1.51283200

<u>Hydroperoxides</u>

Methyl hydroperoxide

H_3COOH

DFT Enthalpy = -190.850939 DFT Free Energy = -190.881812

CBS-QB3 Enthalpy = -190.589542 CBS-QB3 Free Energy = -190.620447

0 1

C	1.12961700	-0.22363900	0.02672600
H	1.97292100	0.47126800	0.02466400

H	1.14400500	-0.82582200	0.94203100
H	1.18954700	-0.87767200	-0.84877800
O	-0.01619300	0.60684200	-0.03138300
O	-1.16412600	-0.28550800	-0.09072600
H	-1.64161900	0.00339000	0.69859800

Quinone methides

α,α-Dimethyl quinone methide (**4e**, R = H)

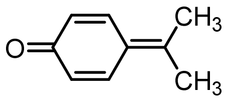

DFT Enthalpy = -424.108921 DFT Free Energy = -424.151520

CBS-QB3 Enthalpy = -423.391518 CBS-QB3 Free Energy = -423.434243

0 1

C	0.00000100	0.17926000	-1.23662900
C	0.00000000	1.52702400	-1.24480700
C	-0.00000300	2.31428500	0.00000000
C	0.00000000	1.52702400	1.24480700
C	0.00000100	0.17926000	1.23662900
H	0.00000200	-0.35460400	-2.17948100
H	0.00000000	2.09303400	-2.16946000
H	0.00000000	2.09303400	2.16946000
H	0.00000200	-0.35460400	2.17948100
C	0.00000100	-0.59228000	0.00000000
C	0.00000100	-1.96065900	0.00000000
O	-0.00000100	3.54079700	0.00000000
C	0.00000000	-2.75520000	1.28672600
H	-0.87967600	-2.53271700	1.89870200
H	0.00000900	-3.82674000	1.08859500
H	0.87966400	-2.53270300	1.89871400
C	0.00000000	-2.75520000	-1.28672600
H	-0.87967600	-2.53271700	-1.89870200
H	0.87966400	-2.53270300	-1.89871400
H	0.00000900	-3.82674000	-1.08859500

α-Methyl quinone methide (**4a**, R = H)

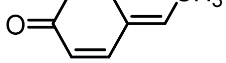

DFT Enthalpy = -384.812984 DFT Free Energy = -384.853432

CBS-QB3 Enthalpy = -384.164138 CBS-QB3 Free Energy = -384.204694

0 1

C	0.06246500	1.40583900	0.00000200
C	1.39290500	1.20302600	-0.00000800
C	1.96685100	-0.15496000	-0.00002000
C	0.99162900	-1.26487900	-0.00000800
C	-0.33688800	-1.04709100	0.00000100
H	-0.34182600	2.41390400	0.00000700
H	2.10129200	2.02338400	-0.00000900
H	1.41007300	-2.26499600	-0.00001000
H	-1.02375900	-1.88641800	0.00000600
C	-0.88995100	0.30150000	0.00000500
C	-2.22323300	0.55261000	0.00001400
H	-2.52735100	1.59612500	0.00001800
O	3.17541300	-0.35564300	-0.00001600
C	-3.32510000	-0.46618100	0.00002100
H	-3.27537300	-1.11591500	0.88015200
H	-4.30305800	0.01580800	0.00001400
H	-3.27536800	-1.11593300	-0.88009600

α-Methyl quinone methide (**4a-*d***, R = H)

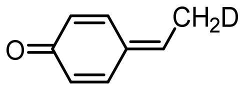

DFT Enthalpy = -384.815982 DFT Free Energy = -384.856616

CBS-QB3 Enthalpy = -384.167103 CBS-QB3 Free Energy = -384.207848

0 1

C	0.06246500	1.40583900	0.00000200
C	1.39290500	1.20302600	-0.00000800
C	1.96685100	-0.15496000	-0.00002000
C	0.99162900	-1.26487900	-0.00000800
C	-0.33688800	-1.04709100	0.00000100
H	-0.34182600	2.41390400	0.00000700
H	2.10129200	2.02338400	-0.00000900
H	1.41007300	-2.26499600	-0.00001000
H	-1.02375900	-1.88641800	0.00000600
C	-0.88995100	0.30150000	0.00000500
C	-2.22323300	0.55261000	0.00001400
H	-2.52735100	1.59612500	0.00001800
O	3.17541300	-0.35564300	-0.00001600
C	-3.32510000	-0.46618100	0.00002100
H(Iso=2)	-3.27537300	-1.11591500	0.88015200
H	-4.30305800	0.01580800	0.00001400
H	-3.27536800	-1.11593300	-0.88009600

SCRF = (CPCM, solvent=chlorobenzene)

DFT Enthalpy = -384.820767 DFT Free Energy = -384.861163

CBS-QB3 Enthalpy = -384.171431 CBS-QB3 Free Energy = -384.211935

0 1

C	0.05918200	1.40374000	0.00000200
C	1.39221100	1.20081600	-0.00000600
C	1.96303900	-0.15314500	-0.00001300
C	0.99458900	-1.26252400	-0.00001000
C	-0.33652200	-1.04703800	-0.00000200
H	-0.34390200	2.41172800	0.00000800
H	2.09569700	2.02568500	-0.00000800
H	1.40930200	-2.26436600	-0.00001600
H	-1.02072900	-1.88795500	-0.00000100
C	-0.88923200	0.29921000	0.00000500
C	-2.22498400	0.55072000	0.00001500
H	-2.52721300	1.59463400	0.00002100
O	3.17961700	-0.35321100	-0.00002100
C	-3.32571200	-0.46530000	0.00002200
H	-3.27169200	-1.11552200	0.87927700
H	-4.30216300	0.01817500	-0.00000100
H	-3.27166900	-1.11556300	-0.87920000

EmpiricalDispersion = GD3

DFT Enthalpy = -384.823760 DFT Free Energy = -384.864167

0 1

C	0.06425500	1.40925300	0.00000300
C	1.39412900	1.20277200	-0.00000800
C	1.96463900	-0.15695200	-0.00002500
C	0.98691300	-1.26498200	-0.00000900
C	-0.34097700	-1.04397700	0.00000100
H	-0.33625500	2.41889300	0.00001000
H	2.10608800	2.01986300	-0.00000700
H	1.40476800	-2.26521200	-0.00000900
H	-1.02954400	-1.88183700	0.00000800
C	-0.89043000	0.30651900	0.00000500
C	-2.22350400	0.55661000	0.00001500
H	-2.53289400	1.59858100	0.00001900
O	3.17265600	-0.36036200	-0.00001400
C	-3.31764100	-0.47064900	0.00002100
H	-3.25851000	-1.11986200	0.88000500
H	-4.30069600	0.00079700	0.00001000
H	-3.25850000	-1.11988600	-0.87994400

Quinone methide

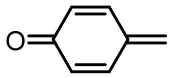

DFT Enthalpy = -345.513500 DFT Free Energy = -345.551382

CBS-QB3 Enthalpy = -344.935618 CBS-QB3 Free Energy = -344.973592

0 1

C	-0.64007800	-1.24671400	0.00001200
C	0.70423200	-1.25335500	0.00000600
C	1.48719700	0.00000000	-0.00000300
C	0.70423300	1.25335500	0.00000600
C	-0.64007800	1.24671400	0.00001200
H	-1.20029400	-2.17667900	0.00002100
H	1.27722800	-2.17344100	0.00000800
H	1.27722700	2.17344100	0.00000800
H	-1.20029400	2.17667900	0.00002100
C	-1.40088400	0.00000000	0.00000500
C	-2.74981200	0.00000000	-0.00001800
H	-3.31508000	0.92474500	-0.00002600
H	-3.31508000	-0.92474500	-0.00002600
O	2.71092900	0.00000000	-0.00001500

α-Phenyl quinone methide (**4b**, R = H)

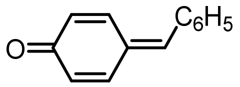

DFT Enthalpy = -576.539362 DFT Free Energy = -576.589007

CBS-QB3 Enthalpy = -575.554316 CBS-QB3 Free Energy = -575.604141

0 1

C	1.17033800	-0.79111800	-0.32834700
C	2.43725600	-1.24459400	-0.40444400
C	3.59858200	-0.38895500	-0.09501600
C	3.28480100	1.00919600	0.24711700
C	2.01446500	1.45268900	0.30497400
H	0.34836500	-1.43920700	-0.60437800
H	2.66296700	-2.25681200	-0.72039100
H	4.13012800	1.65722600	0.44788100
H	1.80554200	2.48671100	0.56320600
C	0.87285200	0.57905300	0.06519500
C	-0.38517700	1.09710900	0.19030700
O	4.74863200	-0.81064700	-0.14222700
C	-1.68687600	0.43948500	0.11025300
C	-2.77885900	1.17629700	-0.38676200
C	-1.92396100	-0.87474600	0.55480400
C	-4.04150500	0.60722000	-0.48872400
H	-2.62177300	2.19984000	-0.71011500
C	-3.19184000	-1.43757000	0.46370500
H	-1.12283500	-1.43870600	1.01453100
C	-4.25227700	-0.70532800	-0.06778000
H	-4.86404600	1.18882300	-0.88896800
H	-3.35597700	-2.44750700	0.82185800
H	-5.23908900	-1.14821700	-0.13748100
H	-0.43913000	2.17060100	0.35999000

α,α-Dicyano quinone methide

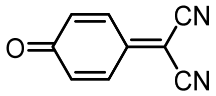

DFT Enthalpy = -530.029824 DFT Free Energy = -530.076761

CBS-QB3 Enthalpy = -529.168150 CBS-QB3 Free Energy = -529.215233

0 1

C	0.00000000	0.52836500	-1.25060000
C	0.00000000	1.87319200	-1.25587000
C	0.00000200	2.65453700	0.00000000
C	0.00000000	1.87319200	1.25587000
C	0.00000000	0.52836500	1.25060000
H	-0.00000100	-0.03070600	-2.17910700
H	-0.00000100	2.44376900	-2.17693000
H	-0.00000100	2.44376900	2.17693000
H	-0.00000100	-0.03070600	2.17910700
C	0.00000000	-0.21582000	0.00000000
C	0.00000000	-1.59367200	0.00000000
O	-0.00000200	3.87520100	0.00000000
C	0.00000000	-2.35080300	1.20913300
C	0.00000000	-2.35080300	-1.20913300
N	0.00000000	-2.96479000	-2.18813400
N	0.00000000	-2.96479000	2.18813400

α,α-Diphenyl quinone methide (**4f**, R = H)

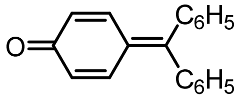

DFT Enthalpy = -807.559158 DFT Free Energy = -807.619831

CBS-QB3 Enthalpy = -806.175841 CBS-QB3 Free Energy = -806.236785

0 1

C	-1.95826300	-1.18546000	0.35132900
C	-3.30725700	-1.18699400	0.36645100
C	-4.09557000	0.00000200	-0.00000400
C	-3.30725200	1.18699600	-0.36645400
C	-1.95825800	1.18545900	-0.35131800
H	-1.41850400	-2.07070500	0.66270700
H	-3.87058700	-2.06152100	0.67201200
H	-3.87057500	2.06152400	-0.67202400
H	-1.41849200	2.07070200	-0.66269200
C	-1.18691500	0.00000000	0.00001200
C	0.19450700	0.00000100	0.00002000

O	-5.32297700	0.00000300	-0.00000900
C	0.98400300	-1.25609300	0.03640900
C	2.07370500	-1.38646100	0.91415700
C	0.69344500	-2.32501800	-0.82774700
C	2.82154200	-2.55744100	0.95134700
H	2.32073100	-0.56746000	1.57925400
C	1.45491300	-3.48962600	-0.80146500
H	-0.11965800	-2.22750000	-1.53704400
C	2.51606600	-3.61294200	0.09263200
H	3.64676100	-2.64599100	1.64881500
H	1.22165700	-4.29865900	-1.48434100
H	3.10643200	-4.52182700	0.11538000
C	0.98400200	1.25609500	-0.03639100
C	0.69345600	2.32503000	0.82775600
C	2.07369500	1.38645000	-0.91415100
C	1.45492700	3.48963500	0.80145400
H	-0.11964000	2.22752100	1.53706300
C	2.82153600	2.55742700	-0.95136200
H	2.32071300	0.56744000	-1.57924100
C	2.51607100	3.61293800	-0.09265500
H	1.22168100	4.29867700	1.48432400
H	3.64674800	2.64596800	-1.64883900
H	3.10644000	4.52182200	-0.11541700

2,6-di-tert-butyl-α-methyl quinone methide (**4a**, R = ^{t}Bu)

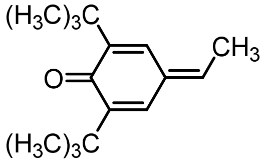

DFT Enthalpy = -699.173329 DFT Free Energy = -699.238444

CBS-QB3 Enthalpy = -697.982229 CBS-QB3 Free Energy = -698.047672

0 1

C	-1.04626800	1.49194700	0.00000000
C	-1.35061900	0.17559200	0.00000100
C	-0.22943800	-0.81236000	0.00000300
C	1.17950100	-0.30392300	0.00000000
C	1.38999100	1.03182800	-0.00000100
H	-1.83224900	2.23736000	-0.00000100
H	2.40065600	1.41394000	-0.00000200
C	0.31303200	2.00248200	0.00000000
C	0.51242900	3.34684800	0.00000100
H	-0.37926400	3.97087500	0.00000100
O	-0.45732600	-2.02149300	0.00000800
C	-2.79980900	-0.33972800	-0.00000100
C	-3.05833800	-1.19189500	1.26672600
H	-4.09876500	-1.53143600	1.27635800

H	-2.40912300	-2.06467500	1.29487200
H	-2.89051500	-0.60037600	2.17196500
C	-3.81563300	0.81869400	-0.00000600
H	-3.71907700	1.45083900	0.88761400
H	-3.71907000	1.45083700	-0.88762800
H	-4.82800800	0.40696300	-0.00000900
C	-3.05833300	-1.19190100	-1.26672500
H	-2.40911800	-2.06468200	-1.29486300
H	-4.09876000	-1.53144100	-1.27636000
H	-2.89050500	-0.60038700	-2.17196600
C	2.33476500	-1.31957100	-0.00000100
C	2.25943300	-2.20687200	1.26706400
H	1.33431900	-2.77882600	1.29445700
H	3.10163900	-2.90570300	1.27715600
H	2.32091400	-1.59507700	2.17230500
C	3.70867200	-0.62196500	-0.00000600
H	4.49489000	-1.38100900	-0.00000700
H	3.85487300	0.00021700	-0.88796200
H	3.85487900	0.00022000	0.88794600
C	2.25942600	-2.20687600	-1.26706300
H	1.33431200	-2.77883200	-1.29444800
H	2.32090100	-1.59508500	-2.17230600
H	3.10163200	-2.90570700	-1.27715600
C	1.80299300	4.09657200	0.00000100
H	1.85999700	4.75280700	0.87646400
H	2.68322600	3.45508100	0.00000200
H	1.85999800	4.75280700	-0.87646100

Truncated dimer (**4c**, R = H)

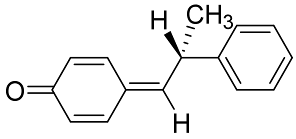

DFT Enthalpy = -655.123649 DFT Free Energy = -655.181436

CBS-QB3 Enthalpy = -654.002976 CBS-QB3 Free Energy = -654.060994

0 1

C	-2.28497800	0.40886900	-1.38146900
C	-3.55270400	-0.03922500	-1.32815500
C	-4.13529300	-0.56092000	-0.07890600
C	-3.23865900	-0.55847700	1.09408900
C	-1.97179800	-0.10764000	1.02679200
H	-1.87418700	0.78842400	-2.31241000
H	-4.20081500	-0.03800300	-2.19697400
H	-3.66131800	-0.94226000	2.01572400
H	-1.34982200	-0.12439600	1.91411400
C	-1.40780200	0.40947300	-0.21456200
C	-0.14133100	0.88393400	-0.33638800

H	0.15649000	1.24391200	-1.31988600
O	-5.28793700	-0.97120500	-0.01361300
C	0.93072100	0.98559600	0.71399400
C	2.15417900	0.17948500	0.27509100
C	2.43091400	-1.05007100	0.87987500
C	3.00520900	0.62664700	-0.74224100
C	3.53098300	-1.81010100	0.48913600
H	1.77888600	-1.41666800	1.66618900
C	4.10408800	-0.13185400	-1.13742500
H	2.81702700	1.57620900	-1.23132600
C	4.37188000	-1.35298100	-0.52222200
H	3.72971200	-2.75869500	0.97523300
H	4.75316900	0.23292500	-1.92573000
H	5.22845900	-1.94234200	-0.82872500
C	1.25966000	2.46665200	1.00604200
H	1.57784500	2.99577200	0.10458500
H	0.38007500	2.98079300	1.40032500
H	2.06455800	2.53733300	1.74154700
H	0.57301500	0.54030300	1.64432900

Truncated dimer (**4c**, R = H)

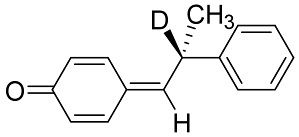

DFT Enthalpy = -655.127050 DFT Free Energy = -655.185001

CBS-QB3 Enthalpy = -654.006339 CBS-QB3 Free Energy = -654.064525

0 1

C	2.28497800	0.40886900	1.38146900
C	3.55270400	-0.03922500	1.32815500
C	4.13529300	-0.56092000	0.07890600
C	3.23865900	-0.55847700	-1.09408900
C	1.97179800	-0.10764000	-1.02679200
H	1.87418700	0.78842400	2.31241000
H	4.20081500	-0.03800300	2.19697400
H	3.66131800	-0.94226000	-2.01572400
H	1.34982200	-0.12439600	-1.91411400
C	1.40780200	0.40947300	0.21456200
C	0.14133100	0.88393400	0.33638800
H	-0.15649000	1.24391200	1.31988600
O	5.28793700	-0.97120500	0.01361300
C	-0.93072100	0.98559600	-0.71399400
C	-2.15417900	0.17948500	-0.27509100
C	-2.43091400	-1.05007100	-0.87987500
C	-3.00520900	0.62664700	0.74224100
C	-3.53098300	-1.81010100	-0.48913600
H	-1.77888600	-1.41666800	-1.66618900
C	-4.10408800	-0.13185400	1.13742500

H	-2.81702700	1.57620900	1.23132600
C	-4.37188000	-1.35298100	0.52222200
H	-3.72971200	-2.75869500	-0.97523300
H	-4.75316900	0.23292500	1.92573000
H	-5.22845900	-1.94234200	0.82872500
C	-1.25966000	2.46665200	-1.00604200
H	-1.57784500	2.99577200	-0.10458500
H	-0.38007500	2.98079300	-1.40032500
H	-2.06455800	2.53733300	-1.74154700
H(Iso=2)	-0.57301500	0.54030300	-1.64432900

Quinone methide dimers

QMD, X = H, meso

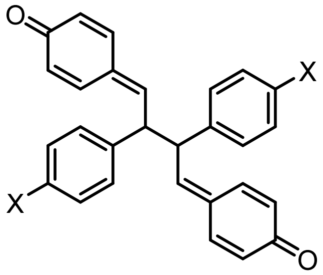

DFT Enthalpy = -1230.464931 DFT Free Energy = -1230.552410

0 1

C	-0.16007500	-1.95808200	-0.05828200
C	-0.18053100	-2.80837900	-1.16703500
C	-0.77030700	-4.06843200	-1.09246200
C	-1.34683400	-4.50005300	0.09874100
C	-1.32806700	-3.66391600	1.21337500
C	-0.73842200	-2.40570700	1.13529500
H	0.26933400	-2.48304200	-2.09949800
H	-0.77847300	-4.71110300	-1.96553700
H	-1.80716600	-5.47935800	0.15946400
H	-1.77463300	-3.99137000	2.14536800
H	-0.73320700	-1.77221400	2.01584800
C	0.50093300	-0.58286000	-0.16181700
H	0.82277500	-0.45661700	-1.19703200
C	-0.50093400	0.58289300	0.16182300
H	-0.82278300	0.45664300	1.19703500
C	0.16007500	1.95811500	0.05830000
C	0.18048600	2.80842200	1.16704700
C	0.73846300	2.40573200	-1.13525900
C	0.77026000	4.06847600	1.09248500
H	-0.26941200	2.48309100	2.09949600
C	1.32810700	3.66394300	-1.21332900
H	0.73328500	1.77223100	-2.01580700
C	1.34682900	4.50008900	-0.09870100
H	0.77839100	4.71115500	1.96555500

H	1.77470600	3.99139100	-2.14530700
H	1.80715900	5.47939600	-0.15941500
C	-1.69521100	0.52606900	-0.75062900
H	-1.45744500	0.52549300	-1.81261600
C	1.69521700	-0.52602800	0.75062700
H	1.45745800	-0.52541400	1.81261500
C	-3.01166800	0.50954500	-0.42147900
C	-3.51468100	0.51923900	0.94692300
C	-4.01071600	0.48445600	-1.48582300
C	-4.83349200	0.50625500	1.21557600
H	-2.80296100	0.54122400	1.76351300
C	-5.33173700	0.46846600	-1.23178300
H	-3.64720100	0.47807100	-2.50908400
C	-5.85250200	0.47752500	0.14709400
H	-5.21122200	0.51491300	2.23169200
H	-6.07048600	0.44877500	-2.02464400
C	3.01167200	-0.50953700	0.42146700
C	3.51467400	-0.51928700	-0.94693800
C	4.01072800	-0.48443200	1.48580300
C	4.83348400	-0.50633800	-1.21560200
H	2.80294800	-0.54128900	-1.76352200
C	5.33174700	-0.46847800	1.23175300
H	3.64722000	-0.47800500	2.50906600
C	5.85250300	-0.47759200	-0.14712700
H	5.21120600	-0.51503900	-2.23172000
H	6.07050200	-0.44877400	2.02460800
O	-7.05171400	0.46192500	0.39572800
O	7.05171300	-0.46202400	-0.39577100

EmpiricalDispersion = GD3

DFT Enthalpy = -1230.521430 DFT Free Energy = -1230.606669

0 1

C	-0.72538000	-1.58864400	0.20580100
C	-1.45804700	-2.10546800	-0.86509300
C	-2.46206600	-3.04758400	-0.65647200
C	-2.74348500	-3.49442700	0.63187500
C	-2.01094300	-2.99599500	1.70742300
C	-1.01231000	-2.04963800	1.49576300
H	-1.25472300	-1.75225200	-1.87031400
H	-3.02864800	-3.42576400	-1.49948800
H	-3.52750200	-4.22404300	0.79707300
H	-2.22011200	-3.34109100	2.71366000
H	-0.46225400	-1.66540800	2.34747200
C	0.29686600	-0.48766100	-0.03854400
H	0.50730700	-0.46181400	-1.10822200
C	-0.31500800	0.92710800	0.34791400
H	-0.71324300	0.82270200	1.35942900
C	0.75325700	2.01018400	0.37465600

C	0.97274300	2.75046500	1.53759900
C	1.54666800	2.27461400	-0.74738600
C	1.96546800	3.72798200	1.58498300
H	0.36243900	2.56086600	2.41474500
C	2.53976300	3.24712800	-0.70380200
H	1.40321100	1.70548500	-1.65960000
C	2.75313800	3.97752600	0.46474200
H	2.12270200	4.29190000	2.49745100
H	3.15289100	3.43003700	-1.57877000
H	3.52914000	4.73328800	0.50037000
C	-1.41485000	1.28494700	-0.60784600
H	-1.07721700	1.73745000	-1.53726200
C	1.56890100	-0.71000900	0.72251200
H	1.48192200	-0.63105300	1.80320200
C	-2.74730900	1.06972600	-0.47157100
C	-3.35514600	0.44878200	0.69892800
C	-3.64447700	1.43600800	-1.56386300
C	-4.67366100	0.18711200	0.75356900
H	-2.71896500	0.16959600	1.52831400
C	-4.96467100	1.18169800	-1.52071500
H	-3.20465800	1.91783300	-2.43221700
C	-5.58656600	0.52271100	-0.35645500
H	-5.12768900	-0.28904700	1.61472300
H	-5.63059400	1.44350500	-2.33473200
C	2.80511800	-0.95773100	0.22431400
C	3.10057600	-1.07666400	-1.19838700
C	3.93035300	-1.09974400	1.14291700
C	4.35188000	-1.29186500	-1.64430400
H	2.28617300	-0.99569300	-1.90836600
C	5.18668300	-1.31205700	0.71115100
H	3.72043200	-1.01789800	2.20522700
C	5.50111900	-1.42009800	-0.72505200
H	4.57808500	-1.38246200	-2.70051600
H	6.02313000	-1.40988100	1.39322200
O	-6.78456400	0.27110100	-0.31164400
O	6.64037300	-1.60443800	-1.13502400

QMD X = H (S,S)

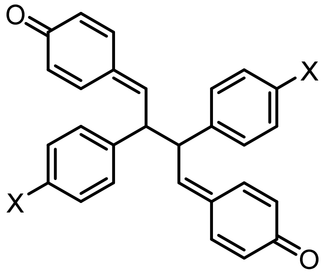

DFT Enthalpy = -1230.462734 DFT Free Energy = -1230.549154

0 1

C	-0.16007500	-1.95808200	-0.05828200

C	-0.18053100	-2.80837900	-1.16703500
C	-0.77030700	-4.06843200	-1.09246200
C	-1.34683400	-4.50005300	0.09874100
C	-1.32806700	-3.66391600	1.21337500
C	-0.73842200	-2.40570700	1.13529500
H	0.26933400	-2.48304200	-2.09949800
H	-0.77847300	-4.71110300	-1.96553700
H	-1.80716600	-5.47935800	0.15946400
H	-1.77463300	-3.99137000	2.14536800
H	-0.73320700	-1.77221400	2.01584800
C	0.50093300	-0.58286000	-0.16181700
H	0.82277500	-0.45661700	-1.19703200
C	-0.50093400	0.58289300	0.16182300
H	-0.82278300	0.45664300	1.19703500
C	0.16007500	1.95811500	0.05830000
C	0.18048600	2.80842200	1.16704700
C	0.73846300	2.40573200	-1.13525900
C	0.77026000	4.06847600	1.09248500
H	-0.26941200	2.48309100	2.09949600
C	1.32810700	3.66394300	-1.21332900
H	0.73328500	1.77223100	-2.01580700
C	1.34682900	4.50008900	-0.09870100
H	0.77839100	4.71115500	1.96555500
H	1.77470600	3.99139100	-2.14530700
H	1.80715900	5.47939600	-0.15941500
C	-1.69521100	0.52606900	-0.75062900
H	-1.45744500	0.52549300	-1.81261600
C	1.69521700	-0.52602800	0.75062700
H	1.45745800	-0.52541400	1.81261500
C	-3.01166800	0.50954500	-0.42147900
C	-3.51468100	0.51923900	0.94692300
C	-4.01071600	0.48445600	-1.48582300
C	-4.83349200	0.50625500	1.21557600
H	-2.80296100	0.54122400	1.76351300
C	-5.33173700	0.46846600	-1.23178300
H	-3.64720100	0.47807100	-2.50908400
C	-5.85250200	0.47752500	0.14709400
H	-5.21122200	0.51491300	2.23169200
H	-6.07048600	0.44877500	-2.02464400
C	3.01167200	-0.50953700	0.42146700
C	3.51467400	-0.51928700	-0.94693800
C	4.01072800	-0.48443200	1.48580300
C	4.83348400	-0.50633800	-1.21560200
H	2.80294800	-0.54128900	-1.76352200
C	5.33174700	-0.46847800	1.23175300
H	3.64722000	-0.47800500	2.50906600
C	5.85250300	-0.47759200	-0.14712700
H	5.21120600	-0.51503900	-2.23172000
H	6.07050200	-0.44877400	2.02460800
O	-7.05171400	0.46192500	0.39572800
O	7.05171300	-0.46202400	-0.39577100

EmpiricalDispersion = GD3

DFT Enthalpy = -1230.521697 DFT Free Energy = -1230.605089

0 1

C	1.01281400	-1.65005800	0.80957900
C	1.39091500	-1.98158300	2.11554800
C	2.55795800	-2.69818600	2.36589100
C	3.37016300	-3.09921600	1.30703000
C	3.00133200	-2.78287400	0.00232600
C	1.83371200	-2.06515800	-0.24373100
H	0.76422500	-1.67181500	2.94641400
H	2.83084800	-2.94473700	3.38575500
H	4.28191500	-3.65332900	1.49713600
H	3.62957500	-3.08380600	-0.82794600
H	1.58540400	-1.80908500	-1.26574600
C	-0.21509800	-0.77638800	0.60980500
H	-0.83066200	-0.87465000	1.50518700
C	0.21509600	0.77639400	0.60979900
C	1.03028500	1.13378700	-0.59126200
H	0.47448400	1.36078900	-1.49588800
C	-1.03028500	-1.13379000	-0.59125500
H	-0.47448100	-1.36079800	-1.49587900
C	2.38403000	1.16224500	-0.68613300
C	3.28618900	0.84669200	0.41623300
C	3.00170200	1.49266200	-1.96763200
C	4.62155100	0.83588300	0.25361000
H	2.86285800	0.58597600	1.37792400
C	4.33522100	1.48474600	-2.14322000
H	2.33991200	1.74370400	-2.79161300
C	5.25693500	1.14631000	-1.04168900
H	5.29465800	0.58736200	1.06571500
H	4.79446600	1.72364900	-3.09539600
C	-2.38403000	-1.16225200	-0.68613000
C	-3.28619300	-0.84669000	0.41623100
C	-3.00169600	-1.49267600	-1.96762900
C	-4.62155500	-0.83588700	0.25360400
H	-2.86286500	-0.58596300	1.37792000
C	-4.33521500	-1.48476600	-2.14322100
H	-2.33990400	-1.74371800	-2.79160700
C	-5.25693400	-1.14633800	-1.04169100
H	-5.29466400	-0.58735600	1.06570400
H	-4.79445700	-1.72367000	-3.09539800
O	6.47217800	1.12260500	-1.19241500
O	-6.47217600	-1.12261100	-1.19242800
C	-1.01281500	1.65006700	0.80956800
C	-1.39091300	1.98160300	2.11553600
C	-1.83371700	2.06515900	-0.24374300
C	-2.55795500	2.69820800	2.36587600

H	-0.76422200	1.67184000	2.94640300
C	-3.00133600	2.78287700	0.00231200
H	-1.58541300	1.80907700	-1.26575600
C	-3.37016300	3.09923000	1.30701400
H	-2.83084200	2.94476700	3.38573900
H	-3.62958200	3.08380300	-0.82796000
H	-4.28191400	3.65334600	1.49711900
H	0.83066000	0.87466100	1.50518100

QMD X = NO_2 meso

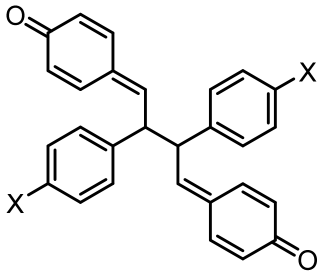

DFT Enthalpy = -1639.562414 DFT Free Energy = -1639.663424

0 1

C	-1.45069000	-1.31661200	-0.09681000
C	-2.02889100	-1.90445100	-1.22674300
C	-3.31697200	-2.42574300	-1.18669400
C	-4.02591800	-2.35651000	0.00499800
C	-3.48224500	-1.78348400	1.14880400
C	-2.19464700	-1.26750100	1.08963400
H	-1.46677600	-1.95863500	-2.15225700
H	-3.77549900	-2.88038500	-2.05369600
H	-4.06828300	-1.75082400	2.05654800
H	-1.77188800	-0.82466400	1.98388100
C	-0.02675500	-0.76709100	-0.16800800
H	0.31347800	-0.88665500	-1.19786300
C	0.02676300	0.76710100	0.16800500
H	-0.31346900	0.88666300	1.19786100
C	1.45069800	1.31662100	0.09680800
C	2.02889000	1.90447800	1.22673700
C	2.19466400	1.26749400	-1.08963000
C	3.31697100	2.42576900	1.18669000
H	1.46676800	1.95867600	2.15224600
C	3.48226300	1.78347700	-1.14879700
H	1.77191200	0.82464300	-1.98387400
C	4.02592700	2.35651900	-0.00499600
H	3.77549200	2.88042400	2.05368900
H	4.06830700	1.75080300	-2.05653700
C	-0.86693300	1.55104600	-0.75634800
H	-0.68255600	1.39003100	-1.81660600
C	0.86694200	-1.55103600	0.75634600
H	0.68257700	-1.39000700	1.81660400
C	-1.83745800	2.44170600	-0.43284800

C	-2.20777300	2.79180500	0.93456500
C	-2.57202800	3.11149400	-1.50414000
C	-3.17595400	3.68825400	1.19625500
H	-1.68290400	2.31858400	1.75601500
C	-3.54344000	4.00752000	-1.25700400
H	-2.30381600	2.85825000	-2.52536300
C	-3.92539300	4.37122900	0.12052000
H	-3.45333900	3.95338100	2.21003300
H	-4.08676300	4.50260000	-2.05333900
C	1.83745700	-2.44170700	0.43284500
C	2.20775400	-2.79182500	-0.93456800
C	2.57203400	-3.11148700	1.50413700
C	3.17592500	-3.68828400	-1.19625800
H	1.68287800	-2.31861000	-1.75601700
C	3.54343700	-4.00752300	1.25700100
H	2.30383500	-2.85823000	2.52536100
C	3.92537200	-4.37125100	-0.12052200
H	3.45329700	-3.95342500	-2.21003600
H	4.08676500	-4.50259700	2.05333700
O	-4.80495900	5.18568300	0.36326800
O	4.80493000	-5.18571300	-0.36327100
N	5.39779100	2.91009200	-0.06006600
N	-5.39778100	-2.91008500	0.06007200
O	-5.85000200	-3.39879400	-0.96549400
O	-5.98988500	-2.84342100	1.12785300
O	5.85000300	3.39881500	0.96549700
O	5.98990300	2.84341300	-1.12784200

EmpiricalDispersion = GD3

DFT Enthalpy = -1639.626443 DFT Free Energy = -1639.725639

0 1

C	1.38267900	1.15025100	0.03192100
C	2.24686900	1.24163300	-1.06321400
C	3.57488200	1.61617800	-0.90083300
C	4.03262000	1.90541800	0.37785700
C	3.19847700	1.83920000	1.48750500
C	1.87506800	1.46059500	1.30588700
H	1.88368700	0.99786500	-2.05467500
H	4.25557700	1.67946100	-1.73804300
H	3.59409100	2.07611900	2.46511700
H	1.22844400	1.40140600	2.17280800
C	-0.03460700	0.63688000	-0.16880500
H	-0.26096300	0.69954100	-1.23338900
C	-0.10919900	-0.90036800	0.23118000
H	0.29953200	-0.97612400	1.24075900
C	-1.54773500	-1.38992900	0.27303000
C	-2.06244800	-1.94974900	1.44519600
C	-2.38263000	-1.28041100	-0.84629700

C	-3.38086400	-2.38661800	1.51169200
H	-1.42697200	-2.04680800	2.31826800
C	-3.70112900	-1.70934500	-0.79924000
H	-2.00930000	-0.84119500	-1.76385400
C	-4.18216700	-2.25761100	0.38505700
H	-3.79322300	-2.81969600	2.41219400
H	-4.35965500	-1.62230100	-1.65190000
C	0.70667600	-1.72474800	-0.72301700
H	0.20227900	-1.97748600	-1.65251100
C	-1.05017900	1.41454300	0.61454200
H	-0.97425500	1.32256500	1.69502200
C	1.99131300	-2.13511900	-0.58109800
C	2.81048100	-1.85526800	0.59346600
C	2.62996200	-2.86798200	-1.67221000
C	4.10499700	-2.21478500	0.65236100
H	2.36414400	-1.32703300	1.42592100
C	3.92207300	-3.23633300	-1.62500500
H	2.02254200	-3.09993300	-2.54201800
C	4.77055600	-2.93086400	-0.45618900
H	4.72236000	-1.99881700	1.51656200
H	4.40070900	-3.77126700	-2.43681200
C	-2.06633000	2.17054200	0.13263600
C	-2.32063100	2.38427700	-1.28837200
C	-2.98899100	2.80670000	1.07002500
C	-3.36310600	3.11873000	-1.71568700
H	-1.64461000	1.94535300	-2.01254600
C	-4.03669400	3.54107000	0.65699200
H	-2.80489900	2.65866000	2.12988200
C	-4.31169400	3.75513500	-0.77662100
H	-3.55751100	3.28372600	-2.76910400
H	-4.72553400	4.00698800	1.35163500
O	5.94882400	-3.25355900	-0.40324500
O	-5.26128600	4.41793300	-1.16959800
O	-5.98934200	-3.17183500	1.50848800
O	-6.26176700	-2.61116300	-0.57014300
N	-5.58980800	-2.71645600	0.44603900
O	5.83323400	2.49083400	1.71067400
O	6.14446700	2.39596300	-0.43508400
N	5.44995500	2.29523600	0.56558700

QMD X = CF_3 meso

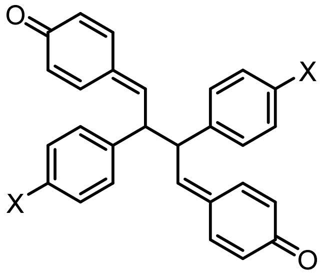

DFT Enthalpy = -1904.723641 DFT Free Energy = -1904.832210

0 1

C	-1.70687800	0.96347900	0.11093300
C	-2.39601200	1.40703200	1.24244200
C	-3.76887800	1.62871300	1.20612600
C	-4.47486000	1.40906900	0.02593900
C	-3.80201300	0.96907000	-1.11362000
C	-2.43086400	0.75028100	-1.06795100
H	-1.85605000	1.58138400	2.16673600
H	-4.29066800	1.96328800	2.09369300
H	-4.35194900	0.78998900	-2.02881800
H	-1.92820800	0.40807300	-1.96544400
C	-0.19604300	0.74037600	0.17432100
H	0.11342600	0.92472900	1.20443400
C	0.19604400	-0.74037600	-0.17432100
H	-0.11342600	-0.92472900	-1.20443300
C	1.70687800	-0.96347900	-0.11093200
C	2.39601200	-1.40703300	-1.24244200
C	2.43086500	-0.75028000	1.06795000
C	3.76887700	-1.62871400	-1.20612600
H	1.85604900	-1.58138600	-2.16673500
C	3.80201400	-0.96906900	1.11362000
H	1.92820900	-0.40807100	1.96544400
C	4.47486100	-1.40906900	-0.02594000
H	4.29066700	-1.96329000	-2.09369300
H	4.35195100	-0.78998700	2.02881700
C	-0.49834400	-1.70888700	0.74528900
H	-0.34753800	-1.51962900	1.80626800
C	0.49834400	1.70888700	-0.74528800
H	0.34754000	1.51962800	-1.80626700
C	-1.25016800	-2.78980500	0.41873500
C	-1.54340400	-3.20205900	-0.94958700
C	-1.81057900	-3.61452200	1.48653800
C	-2.29108600	-4.28860600	-1.21512300
H	-1.14212900	-2.61708100	-1.76864800
C	-2.56154100	-4.70145500	1.23561900
H	-1.59788600	-3.31679700	2.50887400
C	-2.86359900	-5.12913900	-0.14295100
H	-2.50990400	-4.59992200	-2.23014600
H	-2.97623400	-5.31100500	2.02998800
C	1.25016900	2.78980500	-0.41873300
C	1.54340200	3.20206000	0.94958900
C	1.81058100	3.61452200	-1.48653600
C	2.29108300	4.28860700	1.21512600
H	1.14212800	2.61708100	1.76865000
C	2.56154200	4.70145500	-1.23561700
H	1.59789000	3.31679600	-2.50887200
C	2.86359500	5.12914100	0.14295300
H	2.50990200	4.59992200	2.23014800
H	2.97623600	5.31100500	-2.02998600
O	-3.54387300	-6.11605600	-0.38910400
O	3.54387300	6.11605600	0.38910700

C	-5.95009500	1.69740000	-0.03719900
C	5.95009600	-1.69740000	0.03719700
F	-6.54871300	1.53866100	1.16218300
F	-6.19194300	2.97007300	-0.42927400
F	-6.58479100	0.89266200	-0.91582100
F	6.54871400	-1.53864800	-1.16218300
F	6.19194300	-2.97007900	0.42925600
F	6.58479000	-0.89267200	0.91582800

EmpiricalDispersion = GD3

DFT Enthalpy = -1904.790730 DFT Free Energy = -1904.895801

0 1

C	-1.47726400	1.06448300	0.01361300
C	-2.36319900	1.02898800	1.09235700
C	-3.72891500	1.21252900	0.90432100
C	-3.34715900	1.53468900	-1.45676200
C	-1.98617900	1.34126900	-1.26102600
H	-1.98763300	0.81936100	2.08726000
H	-4.41047400	1.15728500	1.74306300
H	-3.73449700	1.74237200	-2.44637800
H	-1.32358800	1.38983100	-2.11676700
C	-0.01925400	0.68730000	0.22268400
H	0.19359700	0.75697500	1.28969200
C	0.19682100	-0.83596300	-0.19558000
H	-0.21643800	-0.93724400	-1.20134300
C	1.67436700	-1.18762600	-0.25928000
C	2.22874200	-1.67769900	-1.44271700
C	2.51009000	-1.01050000	0.84938600
C	3.58435000	-1.98190000	-1.52505200
H	1.59640300	-1.82187400	-2.31198600
C	3.86383700	-1.30790900	0.77631100
H	2.10858300	-0.61721600	1.77626300
H	4.00782900	-2.35380400	-2.44924900
H	4.50719100	-1.14902900	1.63229400
C	-0.52867400	-1.74170800	0.75743100
H	0.02200100	-1.98114300	1.66393000
C	0.92768900	1.56008000	-0.54426100
H	0.86782000	1.47318000	-1.62614800
C	-1.78902000	-2.23217500	0.65000700
C	-2.67110000	-1.97531600	-0.48315500
C	-2.34399400	-3.01306900	1.75322300
C	-3.95769400	-2.36719900	-0.48034900
H	-2.28274200	-1.42407200	-1.32934300
C	-3.62501500	-3.42154600	1.76435600
H	-1.68560100	-3.24090700	2.58635700
C	-4.54434400	-3.11493300	0.65051800
H	-4.62726400	-2.14680200	-1.30296900
H	-4.04401400	-3.98768400	2.58801600

C	1.86996000	2.39852000	-0.04824200
C	2.09686200	2.61776100	1.37609000
C	2.73790900	3.12389200	-0.97210100
C	3.06837800	3.43647700	1.81809100
H	1.45847600	2.11202000	2.09074400
C	3.71480500	3.94308300	-0.54437000
H	2.57403300	2.97147200	-2.03467800
C	3.96262500	4.16387000	0.89271100
H	3.24168800	3.60564500	2.87457000
H	4.36409400	4.47508200	-1.22964100
O	-5.71701500	-3.46360100	0.66008000
O	4.84956900	4.90231000	1.29902500
C	4.40255800	-1.79393000	-0.41492400
C	-4.22197600	1.45118600	-0.37461700
C	5.85819300	-2.16583900	-0.47859300
C	-5.70263200	1.53769000	-0.61753400
F	6.06147900	-3.44162000	-0.07281100
F	6.61520500	-1.37899600	0.31581100
F	6.35110300	-2.07127800	-1.73248500
F	-6.19698200	0.34828500	-1.03850700
F	-6.38607500	1.87867100	0.49333200
F	-6.00411900	2.44270000	-1.57553400

QMD X = CO_2H meso

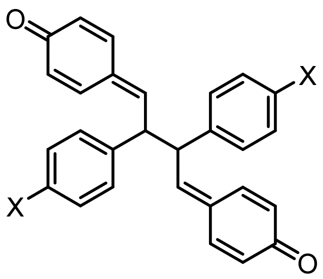

DFT Enthalpy = -1607.689151 DFT Free Energy = -1607.790880

0 1

C	-1.49002900	-1.27401500	-0.08494300
C	-2.09648400	-1.83968200	-1.21021600
C	-3.40105400	-2.31856100	-1.15751200
C	-4.12417500	-2.24251400	0.03519000
C	-3.52512200	-1.68279300	1.16786200
C	-2.22388800	-1.20600800	1.10683700
H	-1.54254700	-1.90821500	-2.14034300
H	-3.86074400	-2.75165800	-2.03599200
H	-4.09721800	-1.63091400	2.08577600
H	-1.78005000	-0.77881100	1.99911300
C	-0.05107200	-0.76620600	-0.16676500
H	0.28043600	-0.89752600	-1.19810900
C	0.05107000	0.76620500	0.16676500
H	-0.28043700	0.89752500	1.19811000
C	1.49002700	1.27401300	0.08494200

C	2.09648400	1.83967600	1.21021600
C	2.22388300	1.20600900	-1.10684000
C	3.40105400	2.31855400	1.15751000
H	1.54255000	1.90820600	2.14034300
C	3.52511700	1.68279300	-1.16786600
H	1.78004200	0.77881600	-1.99911700
C	4.12417300	2.24251100	-0.03519400
H	3.86074800	2.75164800	2.03599000
H	4.09721100	1.63091700	-2.08578200
C	-0.82340500	1.57321000	-0.75466800
H	-0.64387000	1.40845200	-1.81516300
C	0.82340100	-1.57321100	0.75466900
H	0.64386200	-1.40845500	1.81516400
C	-1.76979600	2.48998300	-0.43143300
C	-2.13289300	2.84807400	0.93513700
C	-2.48308500	3.18179600	-1.50214800
C	-3.07582800	3.77168100	1.19656200
H	-1.62423800	2.35697700	1.75614700
C	-3.42904400	4.10527300	-1.25522000
H	-2.22039600	2.92254000	-2.52336000
C	-3.80304600	4.47770700	0.12159200
H	-3.34803800	4.04165000	2.21050600
H	-3.95642400	4.61697300	-2.05187300
C	1.76979500	-2.48998100	0.43143600
C	2.13289900	-2.84806800	-0.93513400
C	2.48308200	-3.18179500	1.50215200
C	3.07583600	-3.77167200	-1.19655700
H	1.62424500	-2.35697100	-1.75614400
C	3.42904400	-4.10526900	1.25522500
H	2.22038900	-2.92254200	2.52336300
C	3.80305200	-4.47769900	-0.12158600
H	3.34805100	-4.04163800	-2.21050100
H	3.95642200	-4.61697000	2.05187900
O	-4.66007200	5.31741300	0.36360500
O	4.66008100	-5.31740300	-0.36359700
C	-5.52124500	-2.73978300	0.15484900
C	5.52124300	2.73977800	-0.15485500
O	-5.99898100	-3.24994600	-1.00586100
H	-6.90382100	-3.54181400	-0.82257400
O	5.99898300	3.24993200	1.00585700
H	6.90382400	3.54179800	0.82256900
O	6.18047100	2.70495700	-1.16554400
O	-6.18047700	-2.70495700	1.16553600

EmpiricalDispersion = GD3

DFT Enthalpy = -1607.754984 DFT Free Energy = -1607.854570

0 1

C	1.39371700	1.13773300	0.03441800

C	2.26708600	1.20325400	-1.05438800
C	3.60507200	1.53611000	-0.88082800
C	4.09147800	1.82097600	0.39722900
C	3.22020500	1.78572000	1.48943400
C	1.88731500	1.44634700	1.30862700
H	1.90242100	0.96297900	-2.04675000
H	4.27721500	1.56220200	-1.72795600
H	3.61113200	2.01480800	2.47296500
H	1.23458200	1.40793000	2.17274200
C	-0.02688500	0.63636900	-0.17070500
H	-0.25005100	0.69232200	-1.23639400
C	-0.11926700	-0.90123600	0.24034100
H	0.27839300	-0.97046100	1.25491900
C	-1.56433800	-1.37095200	0.26808500
C	-2.12130100	-1.85858100	1.45209200
C	-2.37051000	-1.30131900	-0.87526900
C	-3.45172300	-2.26173800	1.50325200
H	-1.50870200	-1.92271800	2.34500900
C	-3.69723100	-1.70019800	-0.83330900
H	-1.96470100	-0.91676900	-1.80422800
C	-4.24810300	-2.18275700	0.35853400
H	-3.87558000	-2.63613900	2.42557200
H	-4.32869000	-1.63992000	-1.71080600
C	0.70328400	-1.73462800	-0.69831700
H	0.20395600	-2.00430100	-1.62564200
C	-1.03935800	1.42371700	0.60472900
H	-0.94546700	1.36298600	1.68587200
C	1.99419200	-2.12547900	-0.55355700
C	2.81050300	-1.82208000	0.61607700
C	2.64696600	-2.84568100	-1.64387400
C	4.11906500	-2.12962700	0.66248400
H	2.35148700	-1.31300800	1.45311300
C	3.95381900	-3.16129600	-1.60943200
H	2.03998500	-3.10575300	-2.50609900
C	4.80374500	-2.80920700	-0.45539300
H	4.73421300	-1.88860000	1.52157000
H	4.44452400	-3.68068600	-2.42425100
C	-2.07192500	2.15614300	0.12054800
C	-2.35650000	2.32636400	-1.29987500
C	-2.98140800	2.81142300	1.05662500
C	-3.41217300	3.04218100	-1.72745200
H	-1.69437300	1.86659700	-2.02365700
C	-4.04176300	3.52786700	0.64338900
H	-2.77622500	2.69410300	2.11650300
C	-4.34550400	3.70031600	-0.78949400
H	-3.62945400	3.17294800	-2.78122300
H	-4.72026500	4.00893200	1.33800700
O	5.99994800	-3.06732500	-0.42356000
O	-5.30694500	4.34788800	-1.18192600
O	-6.39733700	-2.56186600	-0.61830900
O	5.98740900	2.37478500	1.74378000

C	-5.67761700	-2.59514400	0.35036600
C	5.52076400	2.14713400	0.65346100
O	6.26608400	2.15856000	-0.47653900
H	7.17471500	2.35665800	-0.20770600
O	-6.10788800	-3.02322700	1.56255100
H	-7.03891400	-3.26500300	1.45368300

QMD X = Cl meso

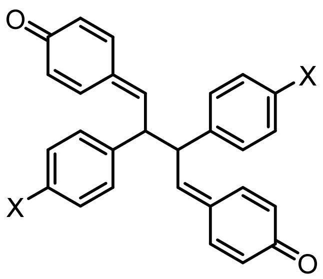

DFT Enthalpy = -2149.726820 DFT Free Energy = -2149.820898

0 1

C	-1.08128400	1.63750100	0.09668100
C	-1.48966700	2.36657000	1.21625900
C	-2.60830700	3.19441500	1.17427400
C	-3.32885600	3.29810400	-0.00958200
C	-2.94451600	2.58831600	-1.14304400
C	-1.82454300	1.76625900	-1.08216800
H	-0.92802700	2.29227600	2.14137300
H	-2.91706800	3.75251100	2.04861700
H	-3.51543200	2.67881100	-2.05795700
H	-1.53633100	1.22237700	-1.97488800
C	0.16064300	0.74946800	0.17067400
H	0.51559000	0.77886200	1.20238600
C	-0.16063800	-0.74947500	-0.17067400
H	-0.51558700	-0.77886800	-1.20238500
C	1.08128900	-1.63750800	-0.09668400
C	1.48966300	-2.36658500	-1.21626000
C	1.82455600	-1.76625900	1.08216000
C	2.60830200	-3.19443100	-1.17427800
H	0.92801600	-2.29229600	-2.14137100
C	2.94452800	-2.58831700	1.14303400
H	1.53635100	-1.22237100	1.97487800
C	3.32885900	-3.29811300	0.00957400
H	2.91705600	-3.75253300	-2.04862000
H	3.51545100	-2.67880700	2.05794400
C	-1.22425600	-1.28983400	0.74672300
H	-1.00598300	-1.18704000	1.80798100
C	1.22426300	1.28982600	-0.74672100
H	1.00599700	1.18702200	-1.80797900
C	-2.38628700	-1.90962200	0.42053900
C	-2.83354500	-2.14613700	-0.94738900
C	-3.26259600	-2.38524800	1.48782900

C	-3.99446800	-2.77253100	-1.21307000
H	-2.20842700	-1.81028500	-1.76622600
C	-4.42670100	-3.01042200	1.23685700
H	-2.93900600	-2.21478100	2.51025800
C	-4.88894200	-3.25646300	-0.14137700
H	-4.32975400	-2.95196600	-2.22821900
H	-5.07493200	-3.36145500	2.03132600
C	2.38628900	1.90962200	-0.42053500
C	2.83353600	2.14615200	0.94739500
C	3.26260300	2.38524500	-1.48782200
C	3.99445400	2.77255400	1.21307900
H	2.20841200	1.81030500	1.76623000
C	4.42670300	3.01042600	-1.23684700
H	2.93902000	2.21476800	-2.51025200
C	4.88893400	3.25647800	0.14138700
H	4.32973100	2.95200100	2.22822900
H	5.07493700	3.36145700	-2.03131400
O	-5.94477600	-3.82477300	-0.38758300
O	5.94476100	3.82480100	0.38759600
Cl	-4.73943900	4.33678300	-0.07735400
Cl	4.73944200	-4.33679300	0.07734200

EmpiricalDispersion = GD3

DFT Enthalpy = -2149.787653 DFT Free Energy = -2149.880061

0 1

C	-1.24792300	1.26026000	-0.08474000
C	-2.12062300	1.43934700	0.99071700
C	-3.40405400	1.94339800	0.80545000
C	-2.96552700	2.13175000	-1.56669300
C	-1.68838300	1.62059800	-1.36277400
H	-1.80517300	1.15998900	1.98969600
H	-4.07909600	2.06385000	1.64242400
H	-3.30120000	2.40679800	-2.55806900
H	-1.03888300	1.49745800	-2.22157300
C	0.10366700	0.59865300	0.13741600
H	0.31969700	0.63849200	1.20558500
C	0.02967500	-0.93776800	-0.26372200
H	-0.39063900	-0.97109600	-1.27117700
C	1.41072700	-1.57216100	-0.31361600
C	1.85242100	-2.20613700	-1.47575000
C	2.27258400	-1.52751200	0.78767000
C	3.12054100	-2.77733100	-1.55001900
H	1.19969800	-2.25825200	-2.34065700
C	3.54225100	-2.08903100	0.73227000
H	1.96391800	-1.03315500	1.70192700
H	3.45707800	-3.26646800	-2.45478800
H	4.20861700	-2.04024000	1.58341700
C	-0.86067300	-1.67478400	0.69413500

H	-0.37304700	-1.99046500	1.61353700
C	1.20627200	1.26710300	-0.62790600
H	1.15107200	1.16188500	-1.70857500
C	-2.18533300	-1.93956900	0.57124200
C	-2.98789900	-1.55708200	-0.58489600
C	-2.88376700	-2.60657300	1.66709100
C	-4.31649900	-1.76392800	-0.62121100
H	-2.49932000	-1.07112300	-1.41916600
C	-4.21075800	-2.82341600	1.64169100
H	-2.29137200	-2.91623400	2.52308800
C	-5.03986700	-2.40839500	0.49343900
H	-4.92056600	-1.46441500	-1.46960700
H	-4.73284300	-3.30863500	2.45807900
C	2.27110200	1.94285900	-0.13132900
C	2.50529800	2.15996700	1.29181500
C	3.26939900	2.48000500	-1.05150800
C	3.59614400	2.80974900	1.73664500
H	1.77414000	1.79409200	2.00291700
C	4.36600200	3.12879900	-0.62084100
H	3.10184600	2.32844700	-2.11365300
C	4.62146700	3.34321900	0.81556600
H	3.77565700	2.97643400	2.79251600
H	5.11104900	3.52021100	-1.30344900
O	-6.24986700	-2.59025600	0.46339900
O	5.61686300	3.92614800	1.22475200
C	3.95728200	-2.71152000	-0.44216000
C	-3.81890100	2.28169000	-0.47771200
Cl	5.55973000	-3.41936800	-0.52379600
Cl	-5.43822100	2.90243600	-0.72910300

QMD = SH meso

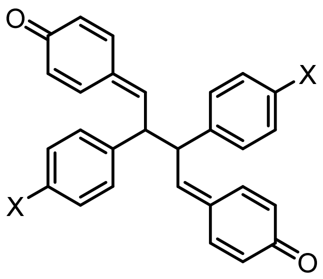

DFT Enthalpy = -2026.894544 DFT Free Energy = -2026.992770

0 1

C	-1.10218900	-1.62455300	-0.09853900
C	-1.51297900	-2.36244500	-1.21082200
C	-2.63958800	-3.17764600	-1.16721400
C	-3.38744800	-3.27860300	0.00798800
C	-2.98374100	-2.54897500	1.13171000
C	-1.85789300	-1.73808500	1.07417600
H	-0.94533300	-2.30394500	-2.13384100
H	-2.93066600	-3.73240800	-2.05158000

H	-3.55130200	-2.61450600	2.05306300
H	-1.57273300	-1.18909200	1.96507800
C	0.14955900	-0.75109400	-0.17177700
H	0.50486300	-0.78432300	-1.20339900
C	-0.14956000	0.75109300	0.17177700
H	-0.50486300	0.78432100	1.20340000
C	1.10218800	1.62455200	0.09853800
C	1.51298100	2.36244100	1.21082100
C	1.85788900	1.73808600	-1.07417900
C	2.63959000	3.17764200	1.16721200
H	0.94533700	2.30394000	2.13384100
C	2.98373700	2.54897500	-1.13171500
H	1.57272700	1.18909500	-1.96508200
C	3.38744700	3.27860100	-0.00799200
H	2.93067100	3.73240200	2.05157800
H	3.55129500	2.61450800	-2.05306900
C	-1.20425800	1.30773500	-0.74560300
H	-0.98563200	1.20243900	-1.80661100
C	1.20425600	-1.30773700	0.74560400
H	0.98562700	-1.20244400	1.80661200
C	-2.35612000	1.94809900	-0.42170600
C	-2.80302200	2.18975800	0.94493300
C	-3.22024300	2.44216800	-1.48983500
C	-3.95292800	2.83746800	1.20881700
H	-2.18696100	1.83927100	1.76447200
C	-4.37330100	3.08886300	-1.24078200
H	-2.89697700	2.26797100	-2.51178700
C	-4.83477700	3.34091000	0.13625400
H	-4.28788400	3.02014700	2.22353500
H	-5.01240600	3.45379200	-2.03644300
C	2.35611900	-1.94809900	0.42170800
C	2.80302500	-2.18975500	-0.94493000
C	3.22024000	-2.44216900	1.48983800
C	3.95293300	-2.83746300	-1.20881200
H	2.18696500	-1.83926700	-1.76447000
C	4.37330000	-3.08886300	1.24078700
H	2.89697200	-2.26797500	2.51179000
C	4.83477900	-3.34090600	-0.13624900
H	4.28789100	-3.02013800	-2.22353000
H	5.01240300	-3.45379300	2.03644800
O	-5.88061900	3.92924800	0.38063700
O	5.88062300	-3.92924300	-0.38063100
S	-4.84194700	-4.29476000	0.15533000
H	-4.82173800	-4.78596000	-1.09349400
S	4.84194600	4.29475900	-0.15533500
H	4.82174100	4.78595500	1.09348900

EmpiricalDispersion = GD3

DFT Enthalpy = -2026.956673 DFT Free Energy = -2027.053239

0 1

C	-1.25046300	1.24598400	-0.08861000
C	-2.13657700	1.40971100	0.97838100
C	-3.42395200	1.89421600	0.78311400
C	-2.97644700	2.09180900	-1.57591100
C	-1.69068700	1.60798000	-1.36576900
H	-1.82478100	1.13443800	1.97993200
H	-4.09077700	2.00237800	1.63074600
H	-3.29122100	2.35059400	-2.58017200
H	-1.03437300	1.50012100	-2.22190000
C	0.10278500	0.59152600	0.13829900
H	0.31496700	0.62986800	1.20743200
C	0.04129100	-0.94549800	-0.26757200
H	-0.37940600	-0.97761200	-1.27515000
C	1.42675000	-1.56830700	-0.31969000
C	1.87960500	-2.19310100	-1.48171400
C	2.29095500	-1.52285900	0.77987300
C	3.15325000	-2.74957200	-1.55632300
H	1.22941200	-2.24836600	-2.34883100
C	3.56349600	-2.07252200	0.72020900
H	1.97847500	-1.03665900	1.69759200
H	3.47479600	-3.22471400	-2.47578300
H	4.21454600	-2.01328200	1.58500800
C	-0.84431300	-1.69125100	0.68736800
H	-0.35078700	-2.01861500	1.59953700
C	1.20500100	1.26591200	-0.62163500
H	1.15703600	1.15653500	-1.70227700
C	-2.17141700	-1.94959800	0.57304600
C	-2.98292100	-1.54814700	-0.56989700
C	-2.86409800	-2.62191600	1.66884000
C	-4.31485900	-1.73564900	-0.59085100
H	-2.49865000	-1.05845300	-1.40428200
C	-4.19424000	-2.82148700	1.65777100
H	-2.26519400	-2.94841700	2.51404000
C	-5.03262600	-2.38111400	0.52602800
H	-4.92568800	-1.41575400	-1.42683000
H	-4.71223800	-3.30925900	2.47530600
C	2.26066900	1.95417500	-0.12204600
C	2.48551600	2.17802600	1.30110700
C	3.25782500	2.49861300	-1.03838700
C	3.56677900	2.84181300	1.74940600
H	1.75514600	1.80515900	2.00933400
C	4.34498100	3.16137700	-0.60430100
H	3.09784600	2.34098900	-2.10086700
C	4.59039900	3.38410200	0.83229900
H	3.73904900	3.01330900	2.80574000
H	5.08939300	3.55838700	-1.28443100
O	-6.24610700	-2.54438600	0.51142600
O	5.57720600	3.98046900	1.24437500
C	4.00909800	-2.69256600	-0.45383600
C	-3.86123700	2.23088800	-0.50164300

S	5.65847200	-3.36631700	-0.46234600
S	-5.54113300	2.78940300	-0.69297900
H	5.62052800	-3.87395800	-1.70433200
H	-5.43168300	3.20001900	-1.96633600

QMD X = CH_3 meso

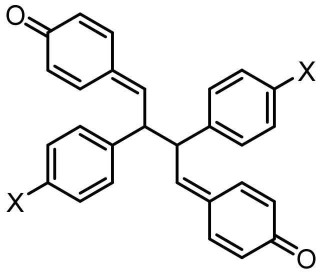

DFT Enthalpy = -1309.062703 DFT Free Energy = -1309.159928

0 1

C	0.53530500	-1.88906800	0.08760700
C	0.71115100	-2.71265600	1.20081500
C	1.53285900	-3.83577100	1.14176000
C	2.20540900	-4.17752200	-0.03331800
C	2.02006600	-3.35636300	-1.15126400
C	1.20116000	-2.23469800	-1.09402200
H	0.19839500	-2.47790400	2.12822100
H	1.65029000	-4.45582400	2.02459300
H	2.52476200	-3.59908700	-2.08096100
H	1.08328000	-1.62797300	-1.98573300
C	-0.37871100	-0.66662700	0.17030200
H	-0.72273300	-0.58997900	1.20334900
C	0.37871100	0.66663500	-0.17030500
H	0.72273800	0.58998300	-1.20334900
C	-0.53530700	1.88907500	-0.08761800
C	-0.71113700	2.71266600	-1.20082500
C	-1.20117800	2.23470100	1.09400300
C	-1.53284500	3.83578100	-1.14177800
H	-0.19836900	2.47791700	-2.12822600
C	-2.02008400	3.35636600	1.15123800
H	-1.08331000	1.62797300	1.98571300
C	-2.20541100	4.17752900	0.03329200
H	-1.65026400	4.45583700	-2.02461000
H	-2.52479300	3.59908600	2.08092900
C	1.55748300	0.85540300	0.74410100
H	1.31782100	0.83257500	1.80547400
C	-1.55748800	-0.85539000	-0.74409700
H	-1.31783500	-0.83254700	-1.80547200
C	2.85555100	1.08132700	0.41819500
C	3.35649200	1.15477800	-0.94880800
C	3.83453400	1.26934300	1.48453300

C	4.65607200	1.38400800	-1.21445500
H	2.65858500	1.02343000	-1.76702100
C	5.13678500	1.49639100	1.23360400
H	3.47218300	1.21820100	2.50696500
C	5.65553700	1.57127600	-0.14384600
H	5.03182600	1.44035300	-2.22980400
H	5.86087600	1.63428800	2.02827100
C	-2.85555300	-1.08132400	-0.41818400
C	-3.35648200	-1.15479600	0.94882100
C	-3.83454300	-1.26933200	-1.48451700
C	-4.65606000	-1.38403400	1.21447700
H	-2.65857000	-1.02345500	1.76703100
C	-5.13679200	-1.49638900	-1.23358100
H	-3.47220100	-1.21817400	-2.50695100
C	-5.65553200	-1.57129400	0.14387300
H	-5.03180500	-1.44039400	2.22982700
H	-5.86088800	-1.63427800	-2.02824300
O	6.83834300	1.77473400	-0.38959700
O	-6.83833600	-1.77475900	0.38963000
C	3.12013500	-5.37610700	-0.09414300
H	2.89878700	-6.08723500	0.70426300
H	3.02856100	-5.89935200	-1.04920200
H	4.16776300	-5.07496100	0.01299700
C	-3.12013600	5.37611500	0.09411000
H	-3.02853700	5.89938400	1.04915400
H	-2.89880800	6.08722400	-0.70431800
H	-4.16776600	5.07496700	-0.01299500

EmpiricalDispersion = GD3

DFT Enthalpy = -1309.125970 DFT Free Energy = -1309.217690

0 1

C	1.21035700	-1.24908900	-0.03979000
C	2.06147500	-1.35424600	1.06567700
C	3.34992500	-1.85438800	0.93641100
C	2.96346500	-2.24488400	-1.39209900
C	1.67607500	-1.72786700	-1.26755800
H	1.72441400	-0.99753100	2.03324700
H	4.00100600	-1.88420600	1.80372900
H	3.30491300	-2.59230200	-2.36171900
H	1.04722100	-1.67918500	-2.14951900
C	-0.11138900	-0.51306900	0.10676900
H	-0.36347800	-0.48707600	1.16734500
C	0.03634900	1.02020700	-0.35793500
H	0.38594300	0.98178000	-1.39199500
C	-1.31400500	1.71336900	-0.33516700
C	-1.89770900	2.16474700	-1.51835400
C	-2.03375700	1.87017400	0.85403000
C	-3.16504600	2.74374900	-1.51775600

H	-1.35896800	2.06012500	-2.45480300
C	-3.29814000	2.44538000	0.85364400
H	-1.61322300	1.52621500	1.79328000
H	-3.59570800	3.08436800	-2.45371000
H	-3.83929700	2.54501800	1.78895100
C	1.04801700	1.70497900	0.50711400
H	0.66717100	2.09828700	1.44630700
C	-1.22770800	-1.14134500	-0.66651200
H	-1.06707000	-1.20690000	-1.73960700
C	2.39067900	1.74591600	0.31226500
C	3.04418200	1.23654000	-0.88541900
C	3.26210900	2.17606700	1.40129300
C	4.36416100	0.98338800	-0.91144900
H	2.43898600	1.02108400	-1.75589900
C	4.58281400	1.91513500	1.39333700
H	2.79829800	2.65459600	2.25890300
C	5.21861500	1.20526900	0.26717700
H	4.85023400	0.56578400	-1.78496400
H	5.22833100	2.17227200	2.22503100
C	-2.42334800	-1.58212800	-0.20263000
C	-2.81803400	-1.54641100	1.20011100
C	-3.40423600	-2.10812400	-1.14669300
C	-4.03359500	-1.95990900	1.60306700
H	-2.10925600	-1.17643100	1.93050900
C	-4.62460100	-2.52058700	-0.75864400
H	-3.11788700	-2.14757600	-2.19352700
C	-5.04256600	-2.47138200	0.65421800
H	-4.33296100	-1.93426300	2.64453200
H	-5.35586700	-2.90254800	-1.46140700
O	6.38139500	0.81499200	0.31078700
O	-6.15484700	-2.83035600	1.02147500
C	-3.89124200	2.88790900	-0.33428200
C	3.83631700	-2.28630300	-0.30273200
C	-5.28297800	3.47016800	-0.33125900
H	-5.44018900	4.12199700	0.53188800
H	-5.47606800	4.05158500	-1.23518000
H	-6.03472500	2.67538200	-0.28105500
C	5.27317200	-2.71747300	-0.44908600
H	5.55729000	-3.42933000	0.33071900
H	5.45723300	-3.18378600	-1.41906500
H	5.93513900	-1.84920700	-0.35657600

QMD X = SMe meso

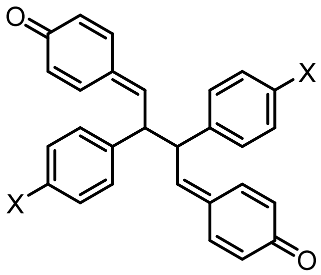

DFT Enthalpy = -2105.479594 DFT Free Energy = -2105.584340

0 1

C	-1.62232500	-1.10970000	0.01792800
C	-2.33024000	-1.67834000	-1.04024500
C	-3.67552500	-2.02272300	-0.91970900
C	-4.35121500	-1.80679500	0.28356200
C	-3.64616200	-1.23957100	1.35624400
C	-2.30875900	-0.90132800	1.22235200
H	-1.82785900	-1.86121800	-1.98483100
H	-4.18196200	-2.45795300	-1.77068800
H	-4.15279800	-1.06316700	2.29869900
H	-1.79842000	-0.46769600	2.07571500
C	-0.14374300	-0.75884300	-0.13976500
H	0.12484300	-0.95466800	-1.17956800
C	0.14374000	0.75884200	0.13976600
H	-0.12484400	0.95466600	1.17956900
C	1.62232200	1.10969900	-0.01793000
C	2.33024200	1.67833200	1.04024400
C	2.30875200	0.90133300	-1.22235700
C	3.67552600	2.02271500	0.91970400
H	1.82786300	1.86120600	1.98483300
C	3.64615500	1.23957500	-1.35625200
H	1.79841000	0.46770600	-2.07572100
C	4.35121200	1.80679300	-0.28357000
H	4.18196700	2.45794000	1.77068400
H	4.15278700	1.06317600	-2.29871000
C	-0.67759900	1.63201500	-0.76903200
H	-0.56196100	1.41922800	-1.83016900
C	0.67759300	-1.63201700	0.76903500
H	0.56194900	-1.41923200	1.83017200
C	-1.50049900	2.66024800	-0.44064100
C	-1.76590700	3.09312100	0.92587600
C	-2.17231100	3.40150100	-1.50374700
C	-2.58693000	4.12554900	1.19393300
H	-1.28406000	2.56759500	1.74173600
C	-2.99728600	4.43366600	-1.25034700
H	-1.98164300	3.08786500	-2.52578800
C	-3.27177400	4.88209200	0.12654400
H	-2.78594200	4.45071300	2.20877900
H	-3.49452300	4.98141600	-2.04249700

C	1.50049800	-2.66024600	0.44064600
C	1.76591500	-3.09311600	-0.92587000
C	2.17230500	-3.40150000	1.50375400
C	2.58694200	-4.12554100	-1.19392400
H	1.28407100	-2.56758900	-1.74173100
C	2.99728400	-4.43366300	1.25035700
H	1.98162900	-3.08786800	2.52579500
C	3.27178500	-4.88208200	-0.12653300
H	2.78596100	-4.45070300	-2.20877000
H	3.49451600	-4.98141400	2.04250900
O	-4.01850600	5.82084900	0.37450700
O	4.01851500	-5.82084100	-0.37449400
S	-6.05783600	-2.19889700	0.57367500
S	6.05783200	2.19889400	-0.57368600
C	-6.61442600	-2.89446900	-1.00881000
H	-6.06686700	-3.80346400	-1.26055300
H	-7.66506700	-3.14513000	-0.85949400
H	-6.53711400	-2.16395900	-1.81495100
C	6.61442800	2.89445600	1.00880100
H	7.66506900	3.14511700	0.85948300
H	6.06687200	3.80345100	1.26055200
H	6.53711900	2.16394200	1.81493800

EmpiricalDispersion = GD3

DFT Enthalpy = -2105.548399 DFT Free Energy = -2105.647832

0 1

C	-1.53844500	1.14288800	0.25044000
C	-2.39576100	1.03564200	1.35088200
C	-3.73506700	1.38166700	1.26069600
C	-3.41300000	1.99681000	-1.04503600
C	-2.07181000	1.63734000	-0.94158900
H	-2.01484400	0.64625000	2.28882900
H	-4.37842300	1.26435900	2.12558600
H	-3.78020400	2.37340200	-1.98992700
H	-1.44665300	1.73356600	-1.82264000
C	-0.11800200	0.62231500	0.34394500
H	0.12765100	0.48101100	1.39726800
C	-0.01422500	-0.83893500	-0.33655800
H	-0.47249200	-0.73897600	-1.32239200
C	1.44238100	-1.22205700	-0.52936200
C	2.00343300	-1.23011400	-1.81015100
C	2.29921400	-1.41346100	0.55562100
C	3.37281300	-1.35834600	-1.99728700
H	1.36332600	-1.09301200	-2.67597500
C	3.67305000	-1.54294000	0.38345300
H	1.90647100	-1.40793900	1.56681700
H	3.78351100	-1.32324400	-3.00030500
H	4.30251100	-1.65027600	1.25615900

C	-0.77264700	-1.82964100	0.48974800
H	-0.21536600	-2.26087500	1.31744800
C	0.89948400	1.51214900	-0.29828300
H	0.71562900	1.75338300	-1.34191900
C	-2.07296300	-2.19941500	0.36556900
C	-2.96680300	-1.69648600	-0.67040700
C	-2.65058700	-3.11704600	1.34341500
C	-4.27417800	-2.01265300	-0.68980200
H	-2.56718900	-1.02874900	-1.42163800
C	-3.95502600	-3.44578700	1.33214200
H	-1.98793600	-3.52050100	2.10360500
C	-4.87939900	-2.90657200	0.31625000
H	-4.94695600	-1.62276400	-1.44454300
H	-4.38839100	-4.11585100	2.06551800
C	2.09180500	1.90490900	0.21645100
C	2.50636500	1.65235500	1.59061700
C	3.08805000	2.49524700	-0.67036200
C	3.78468200	1.80844900	1.97924800
H	1.76212500	1.32297700	2.30580500
C	4.37708400	2.62391800	-0.30471100
H	2.77139200	2.76229300	-1.67392300
C	4.83893900	2.20247700	1.02729500
H	4.10772400	1.60658300	2.99401900
H	5.13675200	2.99292500	-0.98329100
O	-6.07139100	-3.18776400	0.30380700
O	6.02955500	2.15388100	1.32549000
C	4.23294500	-1.48813000	-0.89710100
C	-4.26700700	1.86092000	0.05465500
C	6.73878400	-1.22523000	0.38402500
H	6.54853300	-2.02885300	1.09661900
H	7.81066600	-1.18116400	0.18704500
H	6.41515400	-0.26216800	0.77992100
C	-6.32207200	2.69585400	-1.68485400
H	-6.08935200	1.86718000	-2.35552200
H	-7.39133000	2.90338700	-1.73655700
H	-5.77329300	3.59163200	-1.97950000
S	5.97362500	-1.56745600	-1.23302000
S	-5.99715900	2.25056400	0.04648500

QMD X = OMe meso

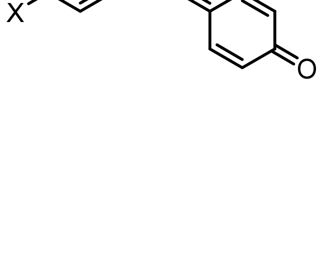

DFT Enthalpy = -1459.502742 DFT Free Energy = -1459.604345

0 1

C	1.14748100	-1.59625800	-0.01679100
C	1.62996000	-2.35864300	1.04468700
C	2.78049900	-3.13993400	0.92754500
C	3.47265500	-3.17120200	-0.28464100
C	2.99717100	-2.41469100	-1.36419600
C	1.85483600	-1.64478700	-1.22799300
H	1.10228900	-2.35078800	1.99307400
H	3.12014100	-3.71234600	1.77999900
H	3.54555400	-2.44896300	-2.29757600
H	1.51303600	-1.07273900	-2.08398200
C	-0.12270600	-0.76198100	0.14222300
H	-0.43925300	-0.85131100	1.18325600
C	0.12260500	0.76211600	-0.14221000
H	0.43924000	0.85142800	-1.18321600
C	-1.14759600	1.59639500	0.01668600
C	-1.62985200	2.35894400	-1.04477700
C	-1.85517300	1.64478400	1.22776400
C	-2.78038500	3.14025900	-0.92774000
H	-1.10200300	2.35120400	-1.99306600
C	-2.99750500	2.41470900	1.36386100
H	-1.51355800	1.07259800	2.08373500
C	-3.47276300	3.17138600	0.28432200
H	-3.11984800	3.71280200	-1.78017800
H	-3.54606200	2.44886800	2.29714300
C	1.19542300	1.30410500	0.76174000
H	1.01658100	1.14656300	1.82379300
C	-1.19561700	-1.30393600	-0.76164000
H	-1.01699300	-1.14617800	-1.82369700
C	2.31987800	1.98840400	0.43001500
C	2.71581700	2.29992700	-0.93765100
C	3.20568200	2.45886500	1.49028000
C	3.84062900	2.98793600	-1.20930300
H	2.08215800	1.96810800	-1.75145400
C	4.33411100	3.14540700	1.23336100
H	2.92012600	2.23324200	2.51348000
C	4.74460500	3.46728500	-0.14491200
H	4.13771800	3.22168900	-2.22537000
H	4.98947000	3.49275100	2.02374900
C	-2.31992800	-1.98843100	-0.42983000
C	-2.71556500	-2.30025800	0.93785400
C	-3.20587900	-2.45880000	-1.49001200
C	-3.84024300	-2.98845000	1.20959500
H	-2.08178000	-1.96852900	1.75159600
C	-4.33417800	-3.14552200	-1.23300400
H	-2.92054700	-2.23295100	-2.51322500
C	-4.74436900	-3.46770400	0.14528800
H	-4.13710500	-3.22243500	2.22567500
H	-4.98964900	-3.49279500	-2.02333000

O	5.76853100	4.09153600	-0.39603200
O	-5.76817400	-4.09212000	0.39649000
O	4.60108600	-3.89317200	-0.51913200
O	-4.60121100	3.89336500	0.51870400
C	5.13845500	-4.67757000	0.53801400
H	6.02615700	-5.15683400	0.12935100
H	5.42461500	-4.05536100	1.39306400
H	4.43068600	-5.44665400	0.86635600
C	-5.13835600	4.67792800	-0.53843300
H	-5.42437800	4.05584900	-1.39362300
H	-6.02611800	5.15716600	-0.12986800
H	-4.43050000	5.44703500	-0.86653500

EmpiricalDispersion = GD3

DFT Enthalpy = -1459.568078 DFT Free Energy = -1459.665671

0 1

C	-1.27854200	1.25195100	0.02068400
C	-2.13099800	1.44452000	1.11509400
C	-3.41406700	1.93888700	0.95294000
C	-3.04665700	2.09360800	-1.42807800
C	-1.75901200	1.59086300	-1.24420800
H	-1.78911000	1.18254300	2.11063500
H	-4.07842400	2.07457100	1.79713200
H	-3.37941900	2.34220700	-2.42643000
H	-1.13360100	1.45584800	-2.11979000
C	0.08165200	0.60268700	0.22178600
H	0.31445000	0.64331700	1.28664900
C	0.03614700	-0.93446600	-0.18243500
H	-0.36922600	-0.96990500	-1.19616100
C	1.42847600	-1.54308200	-0.21266000
C	1.93121000	-2.10960500	-1.37993100
C	2.25855100	-1.52770200	0.91826800
C	3.22002900	-2.64351400	-1.44174600
H	1.31063400	-2.13909800	-2.26988400
C	3.53869500	-2.05001900	0.87748200
H	1.90711500	-1.08524400	1.84421500
H	3.57076400	-3.07299400	-2.37008000
H	4.18609400	-2.02874400	1.74526200
C	-0.86093200	-1.68148000	0.75884300
H	-0.38649400	-1.99307000	1.68651900
C	1.16236400	1.28737900	-0.55835900
H	1.06956900	1.21143700	-1.63903700
C	-2.18107900	-1.96052800	0.61478600
C	-2.96629900	-1.58728800	-0.55484400
C	-2.89227500	-2.63523000	1.69639900
C	-4.29140800	-1.81218300	-0.61644400
H	-2.46806800	-1.09356200	-1.37830500
C	-4.21650100	-2.86796100	1.64634300

H	-2.31295400	-2.93671100	2.56427000
C	-5.02860900	-2.46352100	0.48332200
H	-4.88217200	-1.52282100	-1.47779800
H	-4.74792400	-3.35795600	2.45388800
C	2.24516400	1.95129800	-0.08369400
C	2.53441000	2.12917500	1.33375200
C	3.20545100	2.51737900	-1.02527000
C	3.63889300	2.77310700	1.75434700
H	1.83516100	1.73478800	2.06122600
C	4.31512200	3.16085800	-0.61924900
H	2.99766500	2.39419500	-2.08411500
C	4.62479000	3.33793400	0.81102400
H	3.85914200	2.90871200	2.80698500
H	5.03141700	3.57535200	-1.31900500
O	-6.23692000	-2.65922000	0.42971900
O	5.63359600	3.91614500	1.19668400
C	4.03310300	-2.61362400	-0.30706700
C	-3.88724800	2.26332500	-0.32411300
O	5.30299800	-3.09669700	-0.24385500
O	-5.16141900	2.73260900	-0.38060300
C	5.87250600	-3.66571400	-1.41614000
H	5.92869100	-2.93423700	-2.23056800
H	6.87945100	-3.97201200	-1.13826300
H	5.30731900	-4.54267900	-1.75250200
C	-5.72231100	3.02848200	-1.65352600
H	-5.74515900	2.14117000	-2.29659800
H	-6.74157000	3.35729700	-1.45921300
H	-5.17313900	3.83168800	-2.15842900

QMD X = OH meso

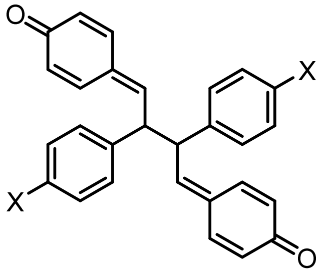

DFT Enthalpy = -1380.940993 DFT Free Energy = -1381.033355

0 1

C	-0.44988200	-1.91269300	-0.08317700
C	-0.57848700	-2.75417000	-1.18915400
C	-1.34157800	-3.91756500	-1.13197000
C	-1.99287100	-4.26493300	0.05174600
C	-1.87138000	-3.43811800	1.17156600
C	-1.10819400	-2.28208600	1.09784300
H	-0.07541700	-2.50285700	-2.11715100
H	-1.42837000	-4.55331900	-2.00819900
H	-2.38222200	-3.71777200	2.08467000

H	-1.02980800	-1.66044800	1.98331900
C	0.40646300	-0.64979500	-0.16876800
H	0.74677900	-0.56064500	-1.20225400
C	-0.40646500	0.64978600	0.16876700
H	-0.74677700	0.56063800	1.20225300
C	0.44988000	1.91268400	0.08317000
C	0.57849900	2.75415700	1.18914800
C	1.10818000	2.28207900	-1.09785600
C	1.34159100	3.91755200	1.13195900
H	0.07543900	2.50284200	2.11715000
C	1.87136600	3.43811100	-1.17158400
H	1.02978200	1.66044500	-1.98333400
C	1.99287100	4.26492200	-0.05176300
H	1.42839400	4.55330200	2.00819000
H	2.38219800	3.71776800	-2.08469300
C	-1.59332000	0.78634700	-0.74497000
H	-1.35338200	0.76716400	-1.80650700
C	1.59331500	-0.78635900	0.74497200
H	1.35337100	-0.76719100	1.80650900
C	-2.89860700	0.96836000	-0.41994000
C	-3.40162100	1.03017100	0.94677500
C	-3.88338100	1.12018200	-1.48657100
C	-4.70766600	1.21982600	1.21209400
H	-2.70001000	0.92223900	1.76534900
C	-5.19202900	1.30789700	-1.23616200
H	-3.51983200	1.07648100	-2.50895900
C	-5.71241400	1.37392400	0.14109900
H	-5.08502500	1.26678300	2.22731500
H	-5.92020000	1.42007400	-2.03114700
C	2.89860500	-0.96836000	0.41994700
C	3.40162700	-1.03015200	-0.94676600
C	3.88337400	-1.12019000	1.48658200
C	4.70767400	-1.21979700	-1.21208000
H	2.70002000	-0.92221400	-1.76534200
C	5.19202400	-1.30789500	1.23617800
H	3.51981900	-1.07650300	2.50896800
C	5.71241800	-1.37390300	-0.14108100
H	5.08503900	-1.26674000	-2.22729900
H	5.92019200	-1.42007700	2.03116600
O	-6.90036800	1.54518200	0.38643000
O	6.90037400	-1.54515200	-0.38640700
O	2.75457300	5.38781700	-0.17999300
H	2.76499100	5.86660000	0.65531200
O	-2.75457300	-5.38782800	0.17997100
H	-2.76498200	-5.86661300	-0.65533300

EmpiricalDispersion = GD3

DFT Enthalpy = -1381.000264 DFT Free Energy = -1381.091363

0 1

C	0.98621400	-1.45514500	-0.12178100
C	1.79376300	-1.82460000	0.95893700
C	2.94317800	-2.58161400	0.78271900
C	2.51278000	-2.64689800	-1.59011500
C	1.36575600	-1.88336800	-1.39666700
H	1.52841100	-1.49762700	1.95848700
H	3.57247400	-2.85549000	1.62012800
H	2.78812200	-2.97049200	-2.58945100
H	0.77051400	-1.61805800	-2.26314200
C	-0.20931600	-0.54079400	0.09761700
H	-0.43444400	-0.54143100	1.16493700
C	0.14792600	0.95489100	-0.29947300
H	0.55687700	0.91145900	-1.31152900
C	-1.08775500	1.84010400	-0.33203300
C	-1.42736700	2.54160300	-1.48783600
C	-1.92713600	1.96631500	0.78290000
C	-2.56745400	3.34077000	-1.54154300
H	-0.79395900	2.46741600	-2.36579600
C	-3.06597600	2.75555000	0.74612300
H	-1.70018200	1.42672700	1.69600500
H	-2.81282600	3.87804500	-2.45277300
H	-3.72025200	2.84308400	1.60445400
C	1.17327500	1.50257500	0.64873400
H	0.76540800	1.89991900	1.57540300
C	-1.41540700	-0.98593000	-0.67316800
H	-1.33210600	-0.89503300	-1.75351600
C	2.52284900	1.51564900	0.50932300
C	3.22304400	0.99486000	-0.65814200
C	3.34907100	2.04054800	1.59244700
C	4.56672100	0.96258600	-0.71873400
H	2.63997400	0.60166400	-1.48027300
C	4.69330000	2.01676600	1.54305300
H	2.83727200	2.44772800	2.45959000
C	5.41398200	1.46551100	0.38000900
H	5.09234100	0.56260200	-1.57802000
H	5.30802700	2.39704500	2.35058600
C	-2.59239600	-1.45236200	-0.18800300
C	-2.87870700	-1.61810800	1.23165900
C	-3.66120300	-1.80129900	-1.11822700
C	-4.07405400	-2.05969500	1.66458500
H	-2.10235100	-1.38421500	1.95029400
C	-4.86179800	-2.24128700	-0.69974600
H	-3.45723400	-1.68519100	-2.17860400
C	-5.16772500	-2.40323600	0.73321800
H	-4.29273000	-2.18579400	2.71872700
H	-5.65753900	-2.49408500	-1.39067400
O	6.63727200	1.43041400	0.32562700
O	-6.25716000	-2.79759200	1.13055000
C	-3.39317500	3.44973900	-0.42240400
C	3.31164900	-2.99545300	-0.49870400

O	-4.52487800	4.20957000	-0.40113000
H	-4.64932100	4.62894600	-1.25867200
O	4.44974800	-3.73501400	-0.61540400
H	4.60942500	-3.94230200	-1.54179200

QMD X = 3,4-dioxyl meso

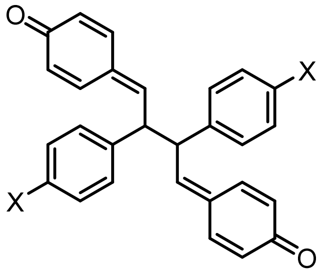

DFT Enthalpy = -1607.581059 DFT Free Energy = -1607.680065

0 1

C	-1.14158000	-1.54577800	-0.37646800
C	-1.97125200	-1.76662700	0.74453400
C	-3.11972400	-2.49896200	0.55375600
C	-3.46810500	-3.01977600	-0.68971700
C	-2.67337800	-2.82832500	-1.79642500
C	-1.49863900	-2.07865500	-1.61465000
H	-1.72977800	-1.38247800	1.72743100
H	-2.94230200	-3.23942300	-2.76061400
H	-0.85369700	-1.91111400	-2.46974600
C	0.14741500	-0.73590600	-0.24018400
H	0.59180300	-0.67133700	-1.23479800
C	-0.12490600	0.73313400	0.24713600
H	-0.57257900	0.66742000	1.24018200
C	1.16271900	1.54426300	0.38817200
C	2.00182400	1.75788100	-0.72725500
C	1.50547200	2.09160000	1.62407800
C	3.14091900	2.50440700	-0.53505700
H	1.77655200	1.35437000	-1.70623000
C	2.67405000	2.85011100	1.80865300
H	0.85166900	1.93244100	2.47402300
C	3.47453200	3.04020700	0.70580400
H	2.93675200	3.26470200	2.77305300
C	-1.07060100	1.43356200	-0.68918900
H	-0.74884900	1.45633100	-1.72863600
C	1.09681600	-1.43469800	0.69387300
H	0.78983400	-1.43856900	1.73798500
C	-2.24140100	2.05727500	-0.40198400
C	-2.82316000	2.13478600	0.93244100
C	-2.98721900	2.70118600	-1.47909600
C	-3.99071300	2.76522100	1.15941800
H	-2.29545400	1.67353500	1.75858000
C	-4.15642900	3.33238600	-1.26707400
H	-2.56167200	2.65234600	-2.47701300

C	-4.75697700	3.41339400	0.07643000
H	-4.42563800	2.82524000	2.15063100
H	-4.70818800	3.80755600	-2.06994500
C	2.25473800	-2.07830100	0.39884900
C	2.81526500	-2.18326600	-0.94282000
C	3.00714700	-2.71776500	1.47408400
C	3.96920400	-2.83515900	-1.17813900
H	2.28212500	-1.72585300	-1.76765000
C	4.16297300	-3.37025600	1.25370800
H	2.59772700	-2.64778100	2.47746300
C	4.74110800	-3.48078200	-0.09753500
H	4.38833900	-2.91575300	-2.17466000
H	4.71958400	-3.84236800	2.05501800
O	-5.82296000	3.97973900	0.28567500
O	5.79365700	-4.06865200	-0.31483300
O	4.66660600	3.70906000	0.61472300
O	4.11034600	2.81973000	-1.44988100
C	5.00204900	3.72235200	-0.77931800
H	4.86308500	4.73370400	-1.17818600
H	6.02904700	3.37886700	-0.90845700
O	-4.63891100	-3.72389300	-0.58262600
O	-4.05950500	-2.85915300	1.48355400
C	-5.13030000	-3.45499900	0.73710200
H	-5.96835600	-2.75062500	0.67810900
H	-5.42494800	-4.39091400	1.21180600

EmpiricalDispersion = GD3

DFT Enthalpy = -1607.648314 DFT Free Energy = -1607.741131

0 1

C	1.49036900	1.19673200	-0.44979200
C	2.24657300	1.05591700	-1.61245200
C	3.58511700	1.47324100	-1.69105800
C	3.38429400	2.18551100	0.60800800
C	2.07160600	1.78590300	0.69444100
H	1.79738300	0.58759700	-2.48034300
H	4.16821100	1.35382800	-2.59430900
H	1.52828100	1.90872900	1.62244200
C	0.08983600	0.61730700	-0.39351200
H	-0.21061700	0.35646300	-1.40881200
C	0.08132900	-0.76417900	0.44160600
H	0.57006800	-0.54202000	1.39163900
C	-1.34860000	-1.18516900	0.73462800
C	-1.85032700	-1.07399100	2.02968900
C	-2.21920900	-1.51092900	-0.32620300
C	-3.21760600	-1.23410500	2.31718400
H	-1.17453100	-0.82285600	2.83948100
C	-3.55752500	-1.63715200	-0.03918200
H	-1.87674400	-1.59085700	-1.34942000

H	-3.60474500	-1.13213700	3.32231700
C	0.85472200	-1.79973600	-0.31115200
H	0.29555000	-2.32308200	-1.08268500
C	-0.91974000	1.54534800	0.20892700
H	-0.66213100	1.94662100	1.18542400
C	2.17147600	-2.10698100	-0.19202100
C	3.06476700	-1.48839600	0.77928400
C	2.76532600	-3.07976300	-1.10409200
C	4.38332400	-1.75558500	0.79646700
H	2.65240300	-0.78431800	1.48926800
C	4.08242000	-3.35486200	-1.09851200
H	2.10405000	-3.56981700	-1.81277500
C	5.00515900	-2.69754000	-0.15395300
H	5.05280400	-1.29161400	1.51172900
H	4.52720400	-4.06455300	-1.78632800
C	-2.16703800	1.81584500	-0.25269200
C	-2.68426000	1.33607500	-1.52812500
C	-3.11425100	2.50166000	0.61910500
C	-3.99962900	1.36307500	-1.81088800
H	-1.98734200	0.93306800	-2.25270800
C	-4.43531000	2.51580300	0.35908800
H	-2.72759600	2.94445600	1.53195500
C	-4.98614400	1.84861700	-0.83175200
H	-4.39640900	0.97612600	-2.74170300
H	-5.15336100	2.96226900	1.03667100
O	6.20951700	-2.92210900	-0.15217600
O	-6.19338000	1.67569500	-0.98690800
C	-4.05380600	-1.49167100	1.25479100
C	4.13013300	2.03178300	-0.55807100
O	4.15534600	2.78095400	1.57278700
O	5.39064000	2.53392700	-0.36768400
C	5.48991500	2.78288500	1.04018800
H	6.07018700	1.98007400	1.51237000
H	5.94395900	3.75970000	1.20514800
O	-5.41278600	-1.64302600	1.24723000
O	-4.58668600	-1.86672100	-0.90718700
C	-5.79383600	-1.67722800	-0.13993900
H	-6.25262700	-0.72396400	-0.42031100
H	-6.45973600	-2.52397200	-0.30761200

QMD X = NH_2 meso

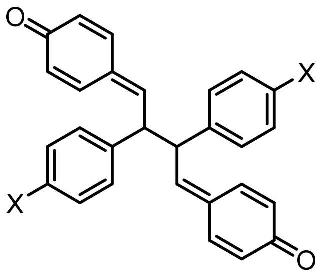

DFT Enthalpy = -1341.177010 DFT Free Energy = -1341.270344

0 1

C	0.40173200	-1.92467800	0.08518500
C	0.47397400	-2.79254800	1.17836100
C	1.19959600	-3.97558200	1.12476300
C	1.88359100	-4.34221900	-0.04389500
C	1.81042500	-3.47818400	-1.14840500
C	1.08216800	-2.29906900	-1.07964900
H	-0.04855800	-2.54138600	2.09610000
H	1.24295700	-4.62154200	1.99579800
H	2.33580700	-3.73435300	-2.06279800
H	1.05032500	-1.66349600	-1.95846500
C	-0.42050000	-0.63943400	0.16873800
H	-0.76053900	-0.54213500	1.20174800
C	0.42050100	0.63942700	-0.16873700
H	0.76053700	0.54213000	-1.20174800
C	-0.40173100	1.92467100	-0.08517900
C	-0.47398600	2.79253800	-1.17835600
C	-1.08215400	2.29906400	1.07966200
C	-1.19960800	3.97557100	-1.12475300
H	0.04853600	2.54137400	-2.09610000
C	-1.81041200	3.47817900	1.14842400
H	-1.05030000	1.66349300	1.95848000
C	-1.88359100	4.34221000	0.04391200
H	-1.24298000	4.62152800	-1.99578900
H	-2.33578400	3.73435000	2.06282200
C	1.60865200	0.74976800	0.74644900
H	1.36793700	0.71566000	1.80755400
C	-1.60864800	-0.74977900	-0.74645200
H	-1.36792800	-0.71568300	-1.80755600
C	2.91584100	0.92856100	0.42496800
C	3.41882200	1.00876800	-0.94015300
C	3.90169400	1.05678800	1.49268900
C	4.72626400	1.19457000	-1.20315800
H	2.71574400	0.91903600	-1.75969600
C	5.21190700	1.23982900	1.24469300
H	3.53861800	0.99764800	2.51450100
C	5.73235100	1.32501100	-0.13094300
H	5.10323900	1.25583500	-2.21782900
H	5.94104800	1.33309800	2.04128300
C	-2.91584000	-0.92856200	-0.42497500
C	-3.41882700	-1.00875200	0.94014400
C	-3.90168800	-1.05679600	-1.49269900
C	-4.72627100	-1.19454600	1.20314400
H	-2.71575300	-0.91901500	1.75969000
C	-5.21190300	-1.23982800	-1.24470800
H	-3.53860600	-0.99766800	-2.51451100
C	-5.73235400	-1.32499300	0.13092600
H	-5.10325200	-1.25579800	2.21781500
H	-5.94104100	-1.33310300	-2.04130100
O	6.92187300	1.49285400	-0.37432200
O	-6.92187800	-1.49282900	0.37430100

N	-2.66058700	5.49411400	0.08794200
H	-2.85074800	5.86389300	1.00702000
H	-2.42201100	6.21336600	-0.57793200
N	2.66058600	-5.49412300	-0.08792000
H	2.42200200	-6.21337700	0.57795000
H	2.85075800	-5.86389900	-1.00699700

EmpiricalDispersion = GD3

DFT Enthalpy = -1341.237922 DFT Free Energy = -1341.330041

0 1

C	0.96684700	-1.41095800	-0.18738800
C	1.81528400	-1.79106500	0.85526400
C	2.96870400	-2.52777200	0.62432900
C	2.45744000	-2.56015700	-1.72738800
C	1.31009100	-1.81936300	-1.48130800
H	1.58318400	-1.48439800	1.86973400
H	3.61698900	-2.78860200	1.45431500
H	2.70151400	-2.85518800	-2.74290100
H	0.68075900	-1.54935100	-2.32238200
C	-0.21827800	-0.49773600	0.07925900
H	-0.41514600	-0.51302100	1.15239600
C	0.13325200	1.00253300	-0.30773700
H	0.55722500	0.96424700	-1.31398500
C	-1.10335600	1.88484800	-0.35540400
C	-1.40294300	2.63204600	-1.49590200
C	-1.98652900	1.97299100	0.72686500
C	-2.53815800	3.43084900	-1.56555000
H	-0.73508200	2.59149600	-2.35078700
C	-3.12418200	2.76366700	0.67225200
H	-1.79700100	1.40024900	1.62868200
H	-2.74723200	3.99436300	-2.46922000
H	-3.79937100	2.79680000	1.52112600
C	1.14275900	1.54892800	0.65767600
H	0.71537000	1.96157200	1.56895900
C	-1.44682200	-0.92938200	-0.66386100
H	-1.41986400	-0.76331200	-1.73804900
C	2.49632500	1.54038000	0.55592600
C	3.22210000	0.99171900	-0.58248900
C	3.29851600	2.05930300	1.65939600
C	4.56582800	0.91835600	-0.59435200
H	2.65761700	0.60348900	-1.41975100
C	4.64260700	1.99651000	1.65776700
H	2.76854500	2.49252500	2.50282200
C	5.38715200	1.40507700	0.52994100
H	5.10763500	0.48675100	-1.42772100
H	5.23964500	2.37034800	2.48157100
C	-2.58095100	-1.47310100	-0.15612400
C	-2.78539700	-1.74676200	1.26031600

C	-3.68461700	-1.79675900	-1.05282900
C	-3.94058500	-2.26228100	1.72103800
H	-1.97891100	-1.53586400	1.95263600
C	-4.84619200	-2.30927200	-0.60597400
H	-3.54225000	-1.59826500	-2.11098900
C	-5.07026700	-2.58051000	0.82489900
H	-4.09791700	-2.47003200	2.77329000
H	-5.66884700	-2.54187200	-1.27207100
O	6.61033200	1.32529400	0.52666200
O	-6.12431700	-3.03997300	1.24919900
C	-3.42298300	3.51042200	-0.47998000
C	3.31730600	-2.92103200	-0.67685200
N	-4.59519100	4.25466700	-0.56351400
H	-5.00743200	4.52873400	0.31547700
H	-4.59567400	5.00262200	-1.24046300
N	4.50662900	-3.59250100	-0.92899500
H	4.92742400	-4.06549800	-0.14402800
H	4.54292700	-4.13437400	-1.77873300

Trans-4-hydroxy stilbenes

trans-4-hydroxystilbene X = H

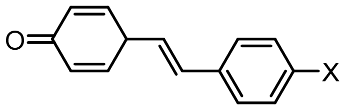

DFT Enthalpy = -615.853301 DFT Free Energy = -615.908611

CBS-QB3 Enthalpy = -614.799749 CBS-QB3 Free Energy = -614.855267

0 1

C	-1.45801200	0.24162400	0.00318000
C	-2.04035300	-1.03545500	0.07324600
C	-3.41735400	-1.20375100	0.06903200
C	-4.26343200	-0.09241400	-0.00425000
C	-3.71103500	1.18691600	-0.07133400
C	-2.33264700	1.34105000	-0.06629800
H	-1.41240300	-1.91609000	0.13658600
H	-3.84086800	-2.20272800	0.12547200
H	-4.37363100	2.04199700	-0.12618300
H	-1.91591000	2.34157200	-0.11925900
C	-0.01446500	0.47987300	0.00049700
H	0.26054200	1.53148400	-0.01401000
C	0.96628800	-0.44117900	0.00562700
H	0.69489900	-1.49388000	0.00184100
C	2.41058700	-0.19511800	0.00539700
C	3.28384700	-1.29398100	-0.06732800
C	2.98303800	1.08837900	0.07617800
C	4.66452800	-1.12421100	-0.07537600
H	2.86663400	-2.29453300	-0.12017800
C	4.36085000	1.25846400	0.06786000

H	2.34619700	1.96248100	0.14376500
C	5.21164000	0.15433200	-0.00869200
H	5.31296800	-1.99147400	-0.13343800
H	4.77681000	2.25850000	0.12415100
H	6.28684800	0.29190700	-0.01375500
O	-5.62401300	-0.19857500	-0.01127900
H	-5.87086300	-1.12781000	0.03879700

trans-4-hydroxystilbene X = NO_2

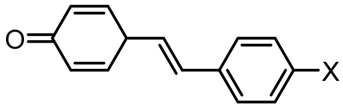

DFT Enthalpy = -820.406261 DFT Free Energy = -820.467850

CBS-QB3 Enthalpy = -819.091596 CBS-QB3 Free Energy = -819.153435

0 1

C	-2.68222900	0.22835700	0.00000300
C	-3.29710000	-1.03605100	0.00011700
C	-4.67693800	-1.16670400	0.00011000
C	-5.49261300	-0.02911500	-0.00001100
C	-4.90670000	1.23805600	-0.00012200
C	-3.52583700	1.35476700	-0.00011200
H	-2.69295100	-1.93528000	0.00022000
H	-5.12776000	-2.15495100	0.00020100
H	-5.54690900	2.11150700	-0.00021200
H	-3.08199300	2.34483300	-0.00019900
C	-1.23636700	0.42791800	0.00000000
H	-0.93453900	1.47206100	-0.00003600
C	-0.27720300	-0.51866000	0.00002200
H	-0.57096700	-1.56453400	0.00003000
C	1.16655500	-0.29888000	0.00002000
C	2.02021100	-1.41935900	-0.00006700
C	1.76270800	0.97907000	0.00010500
C	3.39982200	-1.28382200	-0.00008100
H	1.58618400	-2.41302100	-0.00012900
C	3.13808900	1.13014400	0.00009300
H	1.14445300	1.86777200	0.00018900
C	3.94671700	-0.00531900	-0.00000200
H	4.05652600	-2.14247500	-0.00015200
H	3.60066900	2.10721100	0.00016000
O	-6.85224000	-0.09681700	-0.00002400
H	-7.12898400	-1.01921400	0.00006000
N	5.40951800	0.15288000	-0.00001400
O	5.85898600	1.29315100	0.00007600
O	6.09262400	-0.86464500	-0.00011200

trans-4-hydroxystilbene X = CF_3

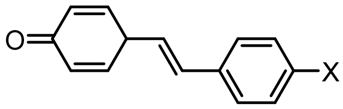

DFT Enthalpy = -952.984989 DFT Free Energy = -953.050919

CBS-QB3 Enthalpy = -951.552780 CBS-QB3 Free Energy = -951.618979

0 1

C	3.12824600	-0.22885200	-0.00289300
C	3.74944600	1.03195000	0.00259200
C	5.13043500	1.15633100	0.00945100
C	5.94097800	0.01571800	0.01107300
C	5.34895200	-1.24807300	0.00572900
C	3.96697800	-1.35831100	-0.00112200
H	3.14954800	1.93408600	0.00152100
H	5.58547400	2.14276300	0.01359800
H	5.98472400	-2.12485500	0.00705900
H	3.51880300	-2.34648500	-0.00520400
C	1.67953800	-0.42169800	-0.01020600
H	1.37289100	-1.46454300	-0.01382300
C	0.72636500	0.52881200	-0.01259300
H	1.02625200	1.57324400	-0.00880400
C	-0.72195700	0.31993600	-0.01913500
C	-1.56778900	1.44243900	-0.02092400
C	-1.32846800	-0.95078400	-0.02791800
C	-2.95046500	1.31204500	-0.02764900
H	-1.12850100	2.43415000	-0.01915200
C	-2.70675000	-1.08735100	-0.03488600
H	-0.71743300	-1.84486200	-0.03211000
C	-3.52760200	0.04418600	-0.03533600
H	-3.58073400	2.19222700	-0.03487500
H	-3.15305000	-2.07410400	-0.04968900
O	7.30234800	0.07771600	0.01771100
H	7.58120000	0.99935300	0.02092100
C	-5.01885900	-0.11587000	0.01094100
F	-5.43724500	-1.18608300	-0.70286000
F	-5.46910800	-0.30106900	1.27651400
F	-5.66611900	0.96653500	-0.47408400

trans-4-hydroxystilbene X = CO_2H

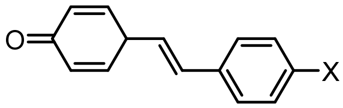

DFT Enthalpy = -804.467536 DFT Free Energy = -804.529692

CBS-QB3 Enthalpy = -803.163590 CBS-QB3 Free Energy = -803.226004

0 1

C	-2.71215000	0.23082000	0.00000500
C	-3.32177300	-1.03581600	0.00010000
C	-4.70152400	-1.17297600	0.00009100
C	-5.52260900	-0.03990800	-0.00001000
C	-4.94239200	1.22938200	-0.00009900
C	-3.56154300	1.35250500	-0.00009000
H	-2.71337900	-1.93222400	0.00018600
H	-5.14748000	-2.16357900	0.00016700
H	-5.58632500	2.10019700	-0.00017500
H	-3.12242200	2.34472000	-0.00016100
C	-1.26576100	0.43714000	0.00000500
H	-0.96806400	1.48245900	-0.00002300
C	-0.30339500	-0.50509300	0.00002200
H	-0.59417700	-1.55216500	0.00002800
C	1.14137800	-0.28244900	0.00002200
C	1.99740200	-1.39918800	-0.00004800
C	1.73575500	0.99563400	0.00008900
C	3.37735700	-1.25698100	-0.00005800
H	1.56429700	-2.39387400	-0.00009700
C	3.11084900	1.14250600	0.00007800
H	1.11508700	1.88326300	0.00015500
C	3.94919200	0.01913300	0.00000500
H	4.01700500	-2.12976700	-0.00011400
H	3.56489500	2.12578600	0.00013000
O	-6.88345400	-0.11471600	-0.00002200
H	-7.15340300	-1.03900000	0.00004600
O	5.95884500	1.31635000	0.00003300
C	5.41510400	0.23631600	-0.00000200
O	6.12365100	-0.92265500	-0.00009900
H	7.05628200	-0.66378400	-0.00011200

trans-4-hydroxystilbene X = Cl

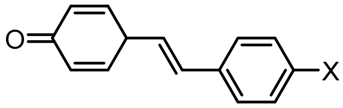

DFT Enthalpy = -1075.485748 DFT Free Energy = -1075.544928

CBS-QB3 Enthalpy = -1073.962242 CBS-QB3 Free Energy = -1074.021649

0 1

C	2.38336100	-0.23521700	0.00208500
C	2.99141900	1.03132900	0.03876500
C	4.37132700	1.17089700	0.03579600
C	5.19434200	0.04032900	-0.00339100
C	4.61581800	-1.22894500	-0.03846600
C	3.23481800	-1.35436700	-0.03501300
H	2.38196300	1.92644800	0.07264000
H	4.81540500	2.16196800	0.06540200

H	5.26082300	-2.09853400	-0.06776300
H	2.79762400	-2.34711600	-0.06270800
C	0.93600300	-0.44388700	0.00130400
H	0.64049600	-1.49005000	-0.00585400
C	-0.02663300	0.49657400	0.00391800
H	0.26440300	1.54372100	0.00119500
C	-1.47381900	0.27725600	0.00451600
C	-2.32956700	1.39126800	-0.03388900
C	-2.07199800	-0.99581500	0.04268900
C	-3.71304200	1.25497400	-0.03767400
H	-1.90017900	2.38712900	-0.06255100
C	-3.45076500	-1.14864900	0.03924700
H	-1.45577000	-1.88592800	0.07853800
C	-4.26587700	-0.01928200	-0.00148400
H	-4.35548800	2.12554000	-0.06824100
H	-3.89629000	-2.13474900	0.06942100
O	6.55590500	0.11763100	-0.00788600
H	6.82340300	1.04218600	0.01839600
Cl	-6.01152700	-0.21120400	-0.00504800

trans-4-hydroxystilbene X = SH

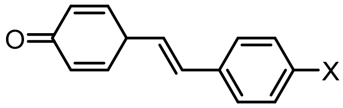

DFT Enthalpy = -1014.069012 DFT Free Energy = -1014.131124

CBS-QB3 Enthalpy = -1012.552306 CBS-QB3 Free Energy = -1012.614658

0 1

C	-2.39392600	-0.23741000	0.00008600
C	-3.00148600	1.02998000	0.00129300
C	-4.38153000	1.17067300	0.00118500
C	-5.20579500	0.04065100	-0.00011200
C	-4.62825900	-1.22933400	-0.00126100
C	-3.24713200	-1.35591300	-0.00113900
H	-2.39133200	1.92528200	0.00242100
H	-4.82448300	2.16277700	0.00216000
H	-5.27392200	-2.09899900	-0.00223000
H	-2.81103000	-2.34957100	-0.00204900
C	-0.94648000	-0.44741900	0.00006400
H	-0.65160400	-1.49382900	-0.00011700
C	0.01684100	0.49278500	0.00009400
H	-0.27530100	1.53987400	-0.00004800
C	1.46341900	0.27662300	0.00012600
C	2.32100600	1.38981000	-0.00109300
C	2.06745500	-0.99368400	0.00134300
C	3.70242000	1.25272500	-0.00121500
H	1.89190600	2.38650300	-0.00201700
C	3.44565600	-1.14202600	0.00125200

H	1.45405000	-1.88688100	0.00248000
C	4.28180500	-0.01876700	-0.00005400
H	4.33046500	2.13665400	-0.00222500
H	3.87284000	-2.13848100	0.00225700
O	-6.56816400	0.11947000	-0.00026900
H	-6.83345500	1.04497200	0.00059500
S	6.05973000	-0.13037700	-0.00019100
H	6.13753900	-1.47019700	0.00056200

trans-4-hydroxystilbene X = CH_3

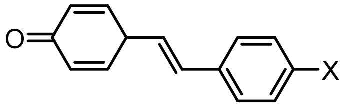

DFT Enthalpy = -655.151961 DFT Free Energy = -655.213711

CBS-QB3 Enthalpy = -654.028707 CBS-QB3 Free Energy = -654.090694

0 1

C	1.95252900	-0.23854000	0.00304600
C	2.55100600	1.03238900	0.04372300
C	3.93014000	1.18335400	0.04023000
C	4.76255700	0.06035400	-0.00327500
C	4.19425400	-1.21304900	-0.04198000
C	2.81391800	-1.34984100	-0.03800600
H	1.93413400	1.92227900	0.08163200
H	4.36585900	2.17817100	0.07312100
H	4.84613400	-2.07749400	-0.07446900
H	2.38497000	-2.34614800	-0.06863300
C	0.50602500	-0.45901500	0.00226400
H	0.21807700	-1.50727800	-0.00279400
C	-0.46342800	0.47420600	0.00215200
H	-0.17844300	1.52342700	-0.00351100
C	-1.90993100	0.24795500	0.00306200
C	-2.77291100	1.35347000	-0.03873400
C	-2.50410300	-1.02757600	0.04451200
C	-4.15585500	1.19844600	-0.04235900
H	-2.34918400	2.35232900	-0.07024300
C	-3.88174400	-1.17721400	0.04067300
H	-1.88214800	-1.91428700	0.08293600
C	-4.73972600	-0.06837000	-0.00317100
H	-4.79029400	2.07846800	-0.07617700
H	-4.30608700	-2.17626000	0.07376400
O	6.12480900	0.14958500	-0.00836900
H	6.38246000	1.07675000	0.02064900
C	-6.23753100	-0.25000400	-0.00468000
H	-6.75451400	0.71025000	-0.05551300
H	-6.56421800	-0.85168200	-0.85882800
H	-6.57640800	-0.76459500	0.90027300

trans-4-hydroxystilbene X = SMe

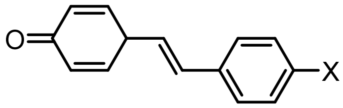

DFT Enthalpy = -1053.361047 DFT Free Energy = -1053.425429

CBS-QB3 Enthalpy = -1051.775411 CBS-QB3 Free Energy = -1051.840056

0 1

C	-2.79426500	0.23834200	0.00216600
C	-3.45498500	-1.00088900	0.05880800
C	-4.83984300	-1.08347500	0.05636800
C	-5.61578100	0.07865800	-0.00186400
C	-4.98515200	1.32185900	-0.05608400
C	-3.59982400	1.39034600	-0.05302800
H	-2.88306000	-1.91971600	0.10905000
H	-5.32415800	-2.05503300	0.10189400
H	-5.59351600	2.21698900	-0.09970500
H	-3.12223700	2.36382300	-0.09592800
C	-1.33909900	0.38688700	-0.00125700
H	-0.99973900	1.41967500	-0.01455400
C	-0.41674200	-0.59367800	0.00406200
H	-0.75422900	-1.62716600	0.00426900
C	1.03775300	-0.44095000	0.00277400
C	1.84689400	-1.59137700	-0.04123600
C	1.69803900	0.79778300	0.04426700
C	3.23019700	-1.51387800	-0.04776200
H	1.37418200	-2.56780500	-0.07240400
C	3.08390200	0.88749000	0.03805300
H	1.12495900	1.71668500	0.08581200
C	3.87237100	-0.26882400	-0.00879900
H	3.81979300	-2.42360700	-0.08314200
H	3.54217400	1.86702000	0.07201900
O	-6.98067200	0.05693800	-0.00661200
H	-7.28408100	-0.85585600	0.03372200
C	6.09636700	1.47503100	0.03657900
H	5.72669800	2.01007600	-0.83900600
H	5.73994200	1.94863400	0.95213500
H	7.18662500	1.49607600	0.02926500
S	5.64831400	-0.28407800	-0.02055100

trans-4-hydroxystilbene X = OMe

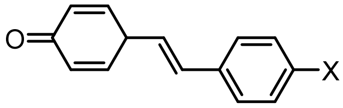

DFT Enthalpy = -730.371844 DFT Free Energy = -730.433073

CBS-QB3 Enthalpy = -729.157262 CBS-QB3 Free Energy = -729.218745

0 1

C	2.38208000	-0.23927600	0.00454700
C	3.02839300	1.00482900	0.10274000
C	4.41240900	1.10436800	0.09821900
C	5.20212400	-0.04520400	-0.00265100
C	4.58615600	-1.29321900	-0.09631500
C	3.20155100	-1.37864700	-0.09095300
H	2.44555400	1.91399000	0.19031900
H	4.88502400	2.07961200	0.17716300
H	5.20494900	-2.17908400	-0.17181400
H	2.73568800	-2.35594500	-0.16522200
C	0.92808000	-0.40465500	-0.00140300
H	0.60016600	-1.44114900	-0.02088800
C	-0.00446200	0.56572800	0.00361300
H	0.32350300	1.60246400	-0.00083700
C	-1.45849700	0.40071200	0.00231600
C	-2.27761400	1.54299000	-0.08593500
C	-2.10712400	-0.84023900	0.08632000
C	-3.65886000	1.45447400	-0.09756200
H	-1.81198800	2.52126400	-0.14923800
C	-3.49379200	-0.94705700	0.07559500
H	-1.52490900	-1.75056500	0.16847700
C	-4.28304300	0.20482500	-0.01814400
H	-4.28039600	2.33897600	-0.16725800
H	-3.94698900	-1.92698500	0.14418200
O	6.56733100	-0.00654900	-0.01095300
H	6.85890400	0.90829000	0.05862200
C	-6.33455500	-1.02506100	0.04147200
H	-6.08859800	-1.67563300	-0.80533100
H	-6.11481000	-1.54969900	0.97811600
H	-7.39456200	-0.77864600	0.00857200
O	-5.64590600	0.21476200	-0.03605100

trans-4-hydroxystilbene X = OH

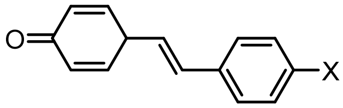

DFT Enthalpy = -691.091308 DFT Free Energy = -691.149348

CBS-QB3 Enthalpy = -689.947992 CBS-QB3 Free Energy = -690.006263

0 1

C	-1.93177700	0.24163900	0.00429400
C	-2.52746300	-1.02975900	0.06643800
C	-3.90634600	-1.18448000	0.06139000
C	-4.74172900	-0.06498700	-0.00457300
C	-4.17645600	1.20888400	-0.06359200
C	-2.79639600	1.34948600	-0.05799700
H	-1.90865900	-1.91728700	0.12407000
H	-4.33933000	-2.17980500	0.11153900
H	-4.83038900	2.07101900	-0.11286500
H	-2.37026500	2.34641900	-0.10483900
C	-0.48566700	0.46573700	0.00258500
H	-0.20063200	1.51497700	-0.00559000
C	0.48566700	-0.46573700	0.00258500
H	0.20063200	-1.51497800	-0.00558800
C	1.93177700	-0.24164000	0.00429500
C	2.79639600	-1.34948600	-0.05799700
C	2.52746300	1.02975900	0.06643900
C	4.17645600	-1.20888400	-0.06359300
H	2.37026500	-2.34641900	-0.10483900
C	3.90634600	1.18448000	0.06139100
H	1.90865900	1.91728700	0.12407100
C	4.74172900	0.06498700	-0.00457300
H	4.83038900	-2.07101900	-0.11286600
H	4.33933000	2.17980500	0.11154000
O	-6.10415300	-0.15800300	-0.01196300
H	-6.35899100	-1.08532200	0.03211200
O	6.10415300	0.15800300	-0.01196200
H	6.35899100	1.08532300	0.03210300

trans-4-hydroxystilbene X = 3,4-dioxyl

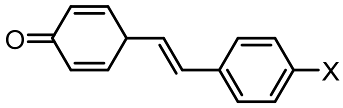

DFT Enthalpy = -804.411631 DFT Free Energy = -804.472149

CBS-QB3 Enthalpy = -803.113796 CBS-QB3 Free Energy = -803.174563

0 1

C	-2.50987400	-0.19429000	-0.00346700
C	-3.21054900	1.02372100	-0.02985500
C	-4.59748100	1.06134600	-0.02344000
C	-5.33530800	-0.12620600	0.00877900
C	-4.66459800	-1.34911700	0.03350100
C	-3.27770900	-1.37261300	0.02687700
H	-2.66891300	1.96167800	-0.05807600
H	-5.11301900	2.01757800	-0.04492500

H	-5.24344900	-2.26435000	0.05740900
H	-2.76881600	-2.33085900	0.04649900
C	-1.05034300	-0.29592000	-0.00643700
H	-0.67845000	-1.31743700	-0.00612100
C	-0.16073400	0.71416200	-0.00499300
H	-0.53087700	1.73601300	0.00395300
C	1.29847900	0.60970200	-0.01011100
C	2.05550700	1.79112800	0.02781300
C	1.97576900	-0.63614000	-0.05011200
C	3.45786400	1.79226400	0.03159900
H	1.53487600	2.74151100	0.05915600
C	3.34736300	-0.61904800	-0.04239500
H	1.44412400	-1.57708000	-0.09067200
C	4.08102800	0.56545300	-0.00201100
H	4.02673300	2.71260100	0.05657500
O	-6.70047400	-0.14874300	0.01619900
H	-7.03295400	0.75453300	-0.00320600
O	5.42271500	0.27972000	-0.03758500
O	4.20945000	-1.68767700	-0.10566400
C	5.51312100	-1.14160700	0.12171200
H	5.83307800	-1.37297400	1.14554700
H	6.20893600	-1.54462400	-0.61450000

trans-4-hydroxystilbene X = NH_2

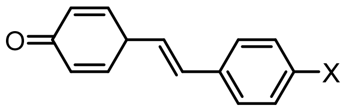

DFT Enthalpy = -671.208933 DFT Free Energy = -671.267969

CBS-QB3 Enthalpy = -670.076772 CBS-QB3 Free Energy = -670.136046

0 1

C	-1.94206000	0.24275500	-0.00183600
C	-2.53659000	-1.03086000	-0.01164400
C	-3.91552100	-1.18732400	-0.00881200
C	-4.75293900	-0.06778000	0.00358000
C	-4.18945200	1.20799800	0.01267400
C	-2.80938100	1.35043500	0.00973900
H	-1.91656100	-1.91936100	-0.02283200
H	-4.34675800	-2.18474500	-0.01698700
H	-4.84458400	2.07066200	0.02171900
H	-2.38500200	2.34921400	0.01681700
C	-0.49635400	0.46948100	-0.00348200
H	-0.21283400	1.51910300	-0.00580400
C	0.47690800	-0.46096700	-0.00139700
H	0.19152100	-1.51044800	0.00321800
C	1.92140700	-0.23862700	-0.00398200
C	2.78795100	-1.34469800	0.00677900
C	2.52251000	1.03385000	-0.01578400

C	4.16784700	-1.20179700	0.00740400
H	2.36426400	-2.34409600	0.01666400
C	3.89788600	1.18899600	-0.01592000
H	1.90399100	1.92383300	-0.02468300
C	4.75227200	0.07241900	-0.00217300
H	4.80254500	-2.08246200	0.01170500
H	4.32660600	2.18638300	-0.03092700
O	-6.11620100	-0.16301700	0.00677100
H	-6.36806300	-1.09209200	-0.00114200
N	6.13496000	0.23110900	-0.06105300
H	6.67702100	-0.54879500	0.28015500
H	6.48584700	1.11589800	0.27441300

Substituted stilbenes

Stilbene X = H

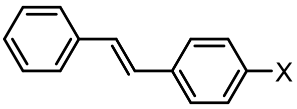

DFT Enthalpy = -540.615077 DFT Free Energy = -540.667614

CBS-QB3 Enthalpy = -539.651513 CBS-QB3 Free Energy = -539.704237

0 1

C	1.93761300	-0.18703500	0.00572300
C	2.49096500	1.10272900	0.10371800
C	3.86614800	1.29257700	0.09426900
C	4.73156700	0.20216900	-0.01024100
C	4.20304200	-1.08248900	-0.10331100
C	2.82492900	-1.27206100	-0.09392800
H	1.84151100	1.96528200	0.19487800
H	4.26855000	2.29662300	0.17214800
H	4.86371900	-1.93864300	-0.18277500
H	2.42130100	-2.27680400	-0.16730700
C	0.49663800	-0.45331400	0.00485900
H	0.23947500	-1.50929300	-0.00756600
C	-0.49663800	0.45331400	0.00485600
H	-0.23947500	1.50929200	-0.00757600
C	-1.93761300	0.18703500	0.00572100
C	-2.82492900	1.27206100	-0.09392800
C	-2.49096600	-1.10272900	0.10371600
C	-4.20304200	1.08248900	-0.10331000
H	-2.42130100	2.27680400	-0.16730800
C	-3.86614800	-1.29257700	0.09426800
H	-1.84151100	-1.96528200	0.19487400
C	-4.73156700	-0.20216900	-0.01024000
H	-4.86371900	1.93864400	-0.18277300
H	-4.26855100	-2.29662300	0.17214700
H	5.80469700	0.35508200	-0.01588400

H -5.80469700 -0.35508100 -0.01588200

Stilbene X = NO_2

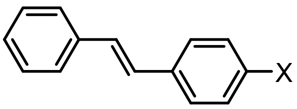

DFT Enthalpy = -745.167348 DFT Free Energy = -745.226989

CBS-QB3 Enthalpy = -743.943193 CBS-QB3 Free Energy = -744.003061

0 1

C	3.15619800	-0.19329300	0.00000300
C	3.75289200	1.08084900	0.00010900
C	5.13364800	1.22005500	0.00009900
C	5.95855700	0.09352100	-0.00001400
C	5.38633400	-1.17569100	-0.00011400
C	4.00254400	-1.31506800	-0.00010400
H	3.13460800	1.97054300	0.00020500
H	5.57245400	2.21150400	0.00018200
H	6.01685600	-2.05753500	-0.00020000
H	3.56310700	-2.30723400	-0.00018400
C	1.70871700	-0.40876000	0.00000300
H	1.41639200	-1.45544100	-0.00002600
C	0.74496800	0.53111000	0.00002000
H	1.03287900	1.57840400	0.00002400
C	-0.69902100	0.30322400	0.00001800
C	-1.55726200	1.41926900	-0.00006200
C	-1.28664500	-0.97792200	0.00009600
C	-2.93654300	1.27617500	-0.00007400
H	-1.12839800	2.41510600	-0.00012000
C	-2.66144600	-1.13670800	0.00008500
H	-0.66299400	-1.86279900	0.00017300
C	-3.47525900	-0.00533900	-0.00000100
H	-3.59813800	2.13103700	-0.00013800
H	-3.11894400	-2.11613800	0.00014500
N	-4.93869700	-0.17183700	-0.00001300
O	-5.38089000	-1.31446900	0.00005100
O	-5.62654500	0.84189900	-0.00009100
H	7.03644200	0.20744700	-0.00002000

Stilbene X = CF_3

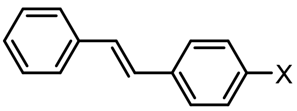

DFT Enthalpy = -877.746388 DFT Free Energy = -877.809701

CBS-QB3 Enthalpy = -876.404558 CBS-QB3 Free Energy = -876.468118

0 1

C	3.59203900	-0.19818300	-0.00346800
C	4.19789000	1.07032500	-0.05652500
C	5.57961100	1.20144900	-0.04133600
C	6.39743900	0.07192000	0.02562000
C	5.81618200	-1.19212900	0.07587500
C	4.43145300	-1.32310400	0.06049700
H	3.58556100	1.96232800	-0.11433700
H	6.02453500	2.18937200	-0.08381900
H	6.44046900	-2.07707600	0.12669200
H	3.98566000	-2.31170500	0.10013800
C	2.14198100	-0.40418300	-0.01277300
H	1.84235700	-1.44891100	-0.00918500
C	1.18586200	0.54189900	-0.01659100
H	1.48210600	1.58710100	-0.00440900
C	-0.26250000	0.32738900	-0.02546100
C	-1.11163600	1.44508400	0.03448600
C	-0.86234800	-0.94403100	-0.09684500
C	-2.49401400	1.30879600	0.03216900
H	-0.67617200	2.43722000	0.08324400
C	-2.24010200	-1.08651400	-0.09964900
H	-0.24692300	-1.83297000	-0.15784800
C	-3.06501800	0.04015300	-0.03485200
H	-3.12825100	2.18505000	0.07431200
H	-2.68270700	-2.07291100	-0.16387800
C	-4.55577000	-0.12988400	0.01364500
F	-4.97429000	-1.15715900	-0.76019700
F	-4.99289500	-0.39366100	1.26935900
F	-5.21159600	0.97629600	-0.39985400
H	7.47597900	0.17928700	0.03654600

Stilbene X = CO_2H

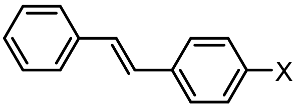

DFT Enthalpy = -729.228940 DFT Free Energy = -729.289140

CBS-QB3 Enthalpy = -728.015238 CBS-QB3 Free Energy = -728.075672

0 1

C	-3.18770800	-0.19272600	-0.00000600
C	-3.77878200	1.08403000	-0.00010100
C	-5.15897900	1.23057500	-0.00009400
C	-5.99032200	0.10889400	0.00000700
C	-5.42395200	-1.16292600	0.00009900
C	-4.04074700	-1.30941200	0.00009100
H	-3.15580000	1.97048700	-0.00018800

H	-5.59220700	2.22460900	-0.00016900
H	-6.05874300	-2.04185400	0.00017600
H	-3.60653700	-2.30392500	0.00016300
C	-1.74055900	-0.41558500	-0.00000400
H	-1.45278800	-1.46351300	0.00001900
C	-0.77278800	0.51955400	-0.00001600
H	-1.05716300	1.56816200	-0.00001700
C	0.67158200	0.28798600	-0.00001400
C	1.53298100	1.39987100	0.00005600
C	1.25692200	-0.99370100	-0.00008200
C	2.91235000	1.24933800	0.00006600
H	1.10557000	2.39694500	0.00010600
C	2.63126500	-1.14895700	-0.00007200
H	0.63042800	-1.87717400	-0.00014800
C	3.47557000	-0.03029100	0.00000300
H	3.55745600	2.11803000	0.00012200
H	3.07970900	-2.13475500	-0.00012500
O	5.47691300	-1.34028000	-0.00004500
C	4.94110300	-0.25662200	0.00000800
O	5.65592500	0.89775700	0.00008400
H	6.58741200	0.63460800	0.00008200
H	-7.06765500	0.22839400	0.00001200

Stilbene X = Cl

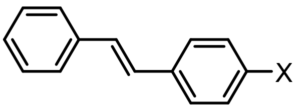

DFT Enthalpy = -1000.247368 DFT Free Energy = -1000.303341

CBS-QB3 Enthalpy = -998.814058 CBS-QB3 Free Energy = -998.870236

0 1

C	2.86012400	-0.19734500	0.00522900
C	3.44874500	1.07762900	0.08958400
C	4.82862200	1.22859100	0.07968600
C	5.66281400	0.11294900	-0.01209700
C	5.09893500	-1.15745200	-0.09209000
C	3.71611500	-1.30821200	-0.08216200
H	2.82391600	1.95924000	0.16972700
H	5.25921200	2.22157100	0.14681700
H	5.73550100	-2.03240900	-0.16177900
H	3.28455500	-2.30194800	-0.14523600
C	1.41268900	-0.42335700	0.00593400
H	1.12700500	-1.47207300	-0.00326300
C	0.44445600	0.51023600	0.00533200
H	0.72929900	1.55881000	-0.00736700
C	-1.00168700	0.28146800	0.00773700
C	-1.86356400	1.38759800	-0.07859800
C	-1.59022700	-0.99334000	0.09444300

C	-3.24601600	1.24144900	-0.08563000
H	-1.44038900	2.38432900	-0.14389400
C	-2.96780500	-1.15597600	0.08845500
H	-0.96751700	-1.87607000	0.17371100
C	-3.78996100	-0.03453900	-0.00276900
H	-3.89438800	2.10537000	-0.15421900
H	-3.40712800	-2.14289400	0.15692800
Cl	-5.53348700	-0.23869200	-0.00890700
H	6.73977400	0.23563900	-0.01833300

Stilbene X = SH

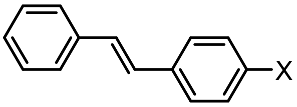

DFT Enthalpy = -938.830809 DFT Free Energy = -938.889288

CBS-QB3 Enthalpy = -937.404244 CBS-QB3 Free Energy = -937.462940

0 1

C	-2.87148300	-0.19880300	0.00380400
C	-3.45967100	1.07842300	0.05432700
C	-4.83950600	1.23025700	0.04790000
C	-5.67552900	0.11357900	-0.00739400
C	-5.11247500	-1.15884800	-0.05507000
C	-3.72972500	-1.31043900	-0.04870700
H	-2.83406000	1.96179300	0.10295400
H	-5.26867900	2.22538700	0.08812600
H	-5.74986100	-2.03508100	-0.09699400
H	-3.29941900	-2.30605700	-0.08640500
C	-1.42444300	-0.42633700	0.00447100
H	-1.13984900	-1.47541900	0.00133200
C	-0.45481400	0.50655500	0.00163200
H	-0.74023400	1.55522900	-0.00835900
C	0.99035000	0.28060400	0.00361300
C	1.85449400	1.38731900	-0.04599400
C	1.58474100	-0.99302900	0.05379200
C	3.23480300	1.24060500	-0.04963800
H	1.43179400	2.38595400	-0.08404700
C	2.96170100	-1.15101000	0.05097900
H	0.96475400	-1.88046000	0.09926400
C	3.80519400	-0.03421800	-0.00159200
H	3.86901100	2.11912600	-0.08992400
H	3.38238200	-2.14933300	0.09170600
S	5.58152800	-0.15803300	-0.00731800
H	5.65030000	-1.49759800	0.03815000
H	-6.75243100	0.23703400	-0.01145100

Stilbene X = CH_3

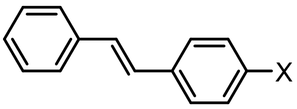

DFT Enthalpy = -579.913835 DFT Free Energy = -579.972399

CBS-QB3 Enthalpy = -578.880469 CBS-QB3 Free Energy = -578.939248

0 1

C	2.43302000	-0.19398200	0.00564900
C	3.00895300	1.08786900	0.07514100
C	4.38727400	1.25356700	0.06665800
C	5.23420700	0.14638800	-0.00902300
C	4.68339900	-1.13059900	-0.07459600
C	3.30216700	-1.29595400	-0.06618700
H	2.37453100	1.96376200	0.14177500
H	4.80666100	2.25218500	0.12202400
H	5.32920800	-1.99981500	-0.13197800
H	2.88148900	-2.29505500	-0.11799900
C	0.98779000	-0.43551300	0.00639200
H	0.71264900	-1.48705300	0.00353500
C	0.00982100	0.48803100	0.00079700
H	0.28555800	1.53940000	-0.01418200
C	-1.43460200	0.24846000	0.00316800
C	-2.30660900	1.34516700	-0.06668800
C	-2.01607700	-1.03142800	0.07304900
C	-3.68797500	1.17724600	-0.07153500
H	-1.89159700	2.34667000	-0.12019700
C	-3.39215300	-1.19392700	0.06800500
H	-1.38557100	-1.91061400	0.13598300
C	-4.25974400	-0.09398900	-0.00473800
H	-4.33056200	2.05004900	-0.12811400
H	-3.80774000	-2.19557300	0.12354700
C	-5.75567900	-0.28945400	-0.00441900
H	-6.28134300	0.66217800	-0.10378200
H	-6.07216000	-0.93521900	-0.82951900
H	-6.09373700	-0.76273200	0.92316400
H	6.30985500	0.28052700	-0.01430300

Stilbene X = SMe

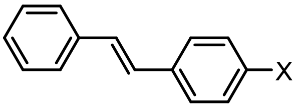

DFT Enthalpy = -978.122923 DFT Free Energy = -978.184265

CBS-QB3 Enthalpy = -976.627170 CBS-QB3 Free Energy = -976.688752

0 1

C	-3.26644200	0.23204800	0.00447100

C	-3.91673300	-1.01298000	0.08860300
C	-5.30237000	-1.09745600	0.08290300
C	-6.08299200	0.05663900	-0.00436100
C	-5.45817900	1.29829300	-0.08430500
C	-4.06968400	1.38237100	-0.07875700
H	-3.33497800	-1.92396700	0.16458000
H	-5.77956400	-2.06905700	0.14959200
H	-6.05191000	2.20325900	-0.15075200
H	-3.59123800	2.35447500	-0.14194700
C	-1.81002900	0.38821300	0.00099800
H	-1.47379200	1.42183300	-0.01371000
C	-0.88759800	-0.59165200	0.00398600
H	-1.22550100	-1.62477400	-0.00011200
C	0.56686600	-0.43898100	0.00230000
C	1.37510600	-1.58893900	-0.06087400
C	1.22618300	0.79934600	0.06199200
C	2.75824400	-1.51112900	-0.06964400
H	0.90207600	-2.56460700	-0.10582300
C	2.61180600	0.88940300	0.05383500
H	0.65252700	1.71690400	0.12071200
C	3.40006600	-0.26635500	-0.01312900
H	3.34807300	-2.41991200	-0.12028500
H	3.07034400	1.86811800	0.10262200
C	5.62399600	1.47665200	0.05508600
H	5.25273900	2.02578600	-0.81101200
H	5.27003600	1.93543400	0.97906800
H	6.71423400	1.49708100	0.04559700
S	5.17513500	-0.28120300	-0.02935900
H	-7.16465000	-0.01414900	-0.00740800

Stilbene X = OMe

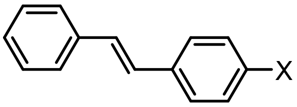

DFT Enthalpy = -655.133861 DFT Free Energy = -655.192407

CBS-QB3 Enthalpy = -654.009061 CBS-QB3 Free Energy = -654.067837

0 1

C	2.85880800	-0.22358700	0.00635200
C	3.49218900	1.02902800	0.10601200
C	4.87657400	1.13322600	0.09863800
C	5.67352400	-0.00822900	-0.00498600
C	5.06572700	-1.25731700	-0.09932100
C	3.67851800	-1.36105100	-0.09234200
H	2.89780000	1.93049500	0.19683200
H	5.33994600	2.11064400	0.17763700
H	5.67187800	-2.15308400	-0.17813400
H	3.21382200	-2.33902800	-0.16707400

C	1.40437700	-0.39981200	0.00250600
H	1.08206000	-1.43797100	-0.01111600
C	0.46932100	0.56773400	0.00156100
H	0.79533800	1.60480700	-0.00858700
C	-0.98406100	0.39978700	0.00054500
C	-1.80471900	1.54093800	-0.08676900
C	-1.62937000	-0.84288700	0.08387600
C	-3.18558600	1.44971300	-0.09754100
H	-1.34071800	2.51993500	-0.14971400
C	-3.01551200	-0.95245500	0.07429400
H	-1.04484500	-1.75185200	0.16407400
C	-3.80694600	0.19841400	-0.01814900
H	-3.80915200	2.33277900	-0.16638600
H	-3.46703600	-1.93313700	0.14228900
C	-5.85626300	-1.03542400	0.04271100
H	-5.61024800	-1.68504700	-0.80466900
H	-5.63492300	-1.55971900	0.97904800
H	-6.91642800	-0.78987200	0.01086100
O	-5.16888500	0.20559500	-0.03505900
H	6.75409100	0.07782500	-0.00891300

Stilbene X = OH

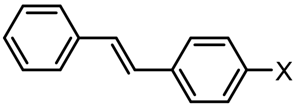

DFT Enthalpy = -615.853301 DFT Free Energy = -615.908611

CBS-QB3 Enthalpy = -614.799749 CBS-QB3 Free Energy = -614.855267

0 1

C	-1.45801200	0.24162400	0.00318000
C	-2.04035300	-1.03545500	0.07324600
C	-3.41735400	-1.20375100	0.06903200
C	-4.26343200	-0.09241400	-0.00425000
C	-3.71103500	1.18691600	-0.07133400
C	-2.33264700	1.34105000	-0.06629800
H	-1.41240300	-1.91609000	0.13658600
H	-3.84086800	-2.20272800	0.12547200
H	-4.37363100	2.04199700	-0.12618300
H	-1.91591000	2.34157200	-0.11925900
C	-0.01446500	0.47987300	0.00049700
H	0.26054200	1.53148400	-0.01401000
C	0.96628800	-0.44117900	0.00562700
H	0.69489900	-1.49388000	0.00184100
C	2.41058700	-0.19511800	0.00539700
C	3.28384700	-1.29398100	-0.06732800
C	2.98303800	1.08837900	0.07617800
C	4.66452800	-1.12421100	-0.07537600
H	2.86663400	-2.29453300	-0.12017800

C	4.36085000	1.25846400	0.06786000
H	2.34619700	1.96248100	0.14376500
C	5.21164000	0.15433200	-0.00869200
H	5.31296800	-1.99147400	-0.13343800
H	4.77681000	2.25850000	0.12415100
H	6.28684800	0.29190700	-0.01375500
O	-5.62401300	-0.19857500	-0.01127900
H	-5.87086300	-1.12781000	0.03879700

Stilbene X = 3,4-dioxyl

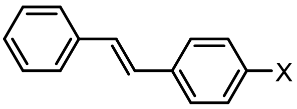

DFT Enthalpy = -729.173580 DFT Free Energy = -729.231647

CBS-QB3 Enthalpy = -727.965550 CBS-QB3 Free Energy = -728.023842

0 1

C	-2.97095200	-0.20495800	-0.00163400
C	-3.66607200	1.01824600	-0.02723800
C	-5.05387500	1.05228200	-0.01946000
C	-5.79239600	-0.13195100	0.01288900
C	-5.12290400	-1.35246700	0.03667500
C	-3.73225400	-1.38598900	0.02905000
H	-3.11792300	1.95252400	-0.05579400
H	-5.56580500	2.00817700	-0.03994700
H	-5.68337700	-2.28048400	0.06101900
H	-3.21901600	-2.34208700	0.04790900
C	-1.50972300	-0.30892900	-0.00588500
H	-1.13747800	-1.33014100	-0.00715600
C	-0.62318000	0.70332900	-0.00347500
H	-0.99685000	1.72363800	0.00665200
C	0.83618000	0.60367800	-0.00909900
C	1.58799400	1.78824100	0.02697900
C	1.51714600	-0.64011700	-0.04786900
C	2.99018700	1.79468200	0.02986500
H	1.06352400	2.73648100	0.05735800
C	2.88850500	-0.61799200	-0.04127800
H	0.98841100	-1.58273700	-0.08648900
C	3.61749100	0.56983200	-0.00281600
H	3.55590300	2.71692500	0.05361500
O	4.95935900	0.28999900	-0.03742200
O	3.75447200	-1.68295600	-0.10297000
C	5.05750600	-1.13191600	0.11627200
H	5.38653200	-1.36494400	1.13660600
H	5.74944200	-1.52902300	-0.62682500
H	-6.87592900	-0.10050800	0.01833700

Stilbene X = NH_2

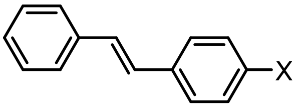

DFT Enthalpy = -595.971136 DFT Free Energy = -596.026980

CBS-QB3 Enthalpy = -594.928718 CBS-QB3 Free Energy = -594.984778

0 1

C	2.42149100	-0.19533700	0.00341900
C	2.99275600	1.09046700	0.04137700
C	4.37036800	1.26249200	0.03847200
C	5.22384200	0.15845100	-0.00087500
C	4.67840000	-1.12213200	-0.03657500
C	3.29798900	-1.29388300	-0.03389000
H	2.35448600	1.96547900	0.07630200
H	4.78418300	2.26465800	0.06851700
H	5.32826200	-1.98987200	-0.06653700
H	2.88277800	-2.29629200	-0.06238600
C	0.97810000	-0.44409100	0.00197600
H	0.70827900	-1.49715300	0.00034000
C	-0.00498200	0.47581000	-0.00279300
H	0.27014000	1.52776000	-0.01131100
C	-1.44654100	0.23895100	-0.00295000
C	-2.32348000	1.33636900	-0.03742700
C	-2.03418100	-1.03943500	0.03150800
C	-3.70151700	1.17973900	-0.03923300
H	-1.90939900	2.33944400	-0.06273200
C	-3.40753400	-1.20849900	0.02951700
H	-1.40632400	-1.92226200	0.06356600
C	-4.27293400	-0.10033300	-0.00445100
H	-4.34513500	2.05323400	-0.07113300
H	-3.82653900	-2.20979400	0.05195000
N	-5.65213200	-0.27495100	-0.06391400
H	-6.20666200	0.51193500	0.23853900
H	-5.99868800	-1.15150100	0.29625900
H	6.29888300	0.29760300	-0.00241600

QM dimer meso extended conformation

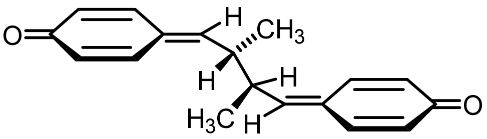

DFT Enthalpy = -847.024559 DFT Free Energy = -847.093800
CBS-QB3 Enthalpy = -845.606924 CBS-QB3 Free Energy = -845.676482

0 1

C	0.53425400	0.06560600	-0.56892800
H	0.82489800	1.11931500	-0.62145400
C	-0.53425600	-0.06560800	0.56894000
H	-0.82490000	-1.11931700	0.62146700
C	-1.73207900	0.78458500	0.26833000
H	-1.50962400	1.84465600	0.14408800
C	1.73207900	-0.78458600	-0.26831900
H	1.50962300	-1.84465600	-0.14407200
C	-3.03668700	0.42135700	0.17032400
C	-3.51568300	-0.94881900	0.31740200
C	-4.04591400	1.44318600	-0.09540200
C	-4.82189200	-1.25591600	0.21133500
H	-2.79516900	-1.73291500	0.51839200
C	-5.35423200	1.14982400	-0.20455000
H	-3.70062400	2.46715300	-0.20417100
C	-5.85040200	-0.23115300	-0.05996700
H	-5.18176000	-2.27269300	0.32030400
H	-6.10060500	1.91048200	-0.40230300
C	3.03668700	-0.42135800	-0.17031900
C	3.51568100	0.94881800	-0.31740600
C	4.04591500	-1.44318500	0.09540800
C	4.82189100	1.25591500	-0.21134600
H	2.79516600	1.73291300	-0.51839700
C	5.35423400	-1.14982200	0.20455000
H	3.70062600	-2.46715200	0.20418200
C	5.85040200	0.23115400	0.05996100
H	5.18175900	2.27269200	-0.32032300
H	6.10060700	-1.91048000	0.40230200
O	-7.03759700	-0.51446100	-0.15778400
O	7.03759800	0.51446200	0.15776200
C	-0.04317700	-0.34620300	-1.93957300
H	-0.91426400	0.26117200	-2.19498800
H	0.70448400	-0.22431300	-2.72588900
H	-0.35720200	-1.39450100	-1.93322000
C	0.04317600	0.34620300	1.93958300
H	0.91426000	-0.26117600	2.19500000
H	0.35720700	1.39449900	1.93322600
H	-0.70448500	0.22431800	2.72590000

EmpiricalDispersion = GD3

DFT Enthalpy = -847.060231 DFT Free Energy = -847.129809

0 1

C	0.47526200	-0.11208800	-0.61389000
H	0.76871900	0.87636800	-0.97935800
C	-0.47526200	0.11209800	0.61388500
H	-0.76871600	-0.87635800	0.97935500
C	-1.68258600	0.89104500	0.19297400
H	-1.46715200	1.91517100	-0.11185000
C	1.68258300	-0.89104000	-0.19297900

H	1.46714700	-1.91516600	0.11184200
C	-2.97943700	0.49493300	0.14720500
C	-3.43202100	-0.84392100	0.50937500
C	-4.00385600	1.43773900	-0.29431600
C	-4.72869700	-1.19594800	0.43235200
H	-2.69838600	-1.56719500	0.84492600
C	-5.30327800	1.09909000	-0.37489400
H	-3.67896900	2.43885900	-0.56235700
C	-5.77269100	-0.25279200	-0.01808300
H	-5.07102700	-2.18949900	0.69804300
H	-6.06303400	1.79818000	-0.70433800
C	2.97943600	-0.49493200	-0.14720800
C	3.43202500	0.84392100	-0.50937400
C	4.00385100	-1.43774200	0.29431500
C	4.72870300	1.19594400	-0.43234800
H	2.69839300	1.56719800	-0.84492500
C	5.30327400	-1.09909700	0.37489600
H	3.67896100	-2.43886200	0.56235300
C	5.77269200	0.25278300	0.01808800
H	5.07103600	2.18949500	-0.69803600
H	6.06302700	-1.79819000	0.70434000
O	-6.95133300	-0.57652700	-0.08891500
O	6.95133500	0.57651500	0.08892500
C	-0.24129800	-0.86090500	-1.75377700
H	-1.11345100	-0.30343400	-2.10068000
H	0.43232300	-1.01142600	-2.59985600
H	-0.58636400	-1.84322000	-1.41625700
C	0.24129700	0.86092000	1.75377100
H	1.11345300	0.30345300	2.10067400
H	0.58635900	1.84323500	1.41624800
H	-0.43232300	1.01143900	2.59985000

QMD dimer (S,S)

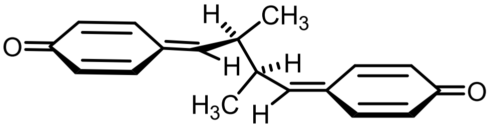

DFT Enthalpy = -847.023158 DFT Free Energy = -847.092282
CBS-QB3 Enthalpy = -845.606244 CBS-QB3 Free Energy = -845.675684

0 1

C	0.51976300	-1.11369700	0.59119100
H	1.12844600	-2.00880800	0.43907600
C	-0.51976300	-1.11369700	-0.59118800
C	-1.40106900	0.09981600	-0.59283400
H	-0.89546900	1.03130800	-0.84291300
C	1.40107000	0.09981500	0.59283800
H	0.89547100	1.03130600	0.84292100
C	-2.73717700	0.18286300	-0.36338600
C	-3.58368700	-0.95833200	-0.03089700

C	-3.39670100	1.48367500	-0.44891900
C	-4.90335900	-0.81788200	0.19343700
H	-3.13068600	-1.94040000	0.03506700
C	-4.71437500	1.63808800	-0.22700800
H	-2.77755100	2.33940900	-0.70186600
C	-5.57843300	0.49361500	0.11648800
H	-5.53529400	-1.66342600	0.44028200
H	-5.19995700	2.60502700	-0.29041100
C	2.73717800	0.18286200	0.36338700
C	3.58368600	-0.95833200	0.03089500
C	3.39670100	1.48367400	0.44892300
C	4.90335800	-0.81788200	-0.19344200
H	3.13068600	-1.94040000	-0.03507000
C	4.71437500	1.63808800	0.22701000
H	2.77755300	2.33940700	0.70187400
C	5.57843200	0.49361500	-0.11649500
H	5.53529300	-1.66342500	-0.44029000
H	5.19995700	2.60502600	0.29041500
O	-6.77870300	0.61788800	0.32367500
O	6.77870300	0.61788800	-0.32367900
H	-1.12844600	-2.00880700	-0.43907400
C	0.18517900	-1.24902300	-1.95504900
H	-0.54660800	-1.33159000	-2.76156600
H	0.82270800	-0.38582900	-2.16267100
H	0.81380600	-2.14350500	-1.97585100
C	-0.18517900	-1.24902600	1.95505100
H	-0.82270800	-0.38583200	2.16267400
H	-0.81380600	-2.14350800	1.97585200
H	0.54660800	-1.33159300	2.76156900

EmpiricalDispersion = GD3

DFT Enthalpy = -847.059878 DFT Free Energy = -847.128128

0 1

C	0.44268600	-1.23722500	0.65132900
H	1.08588700	-2.11754100	0.56977600
C	-0.44268500	-1.23722400	-0.65132900
C	-1.28186500	0.00122800	-0.75163600
H	-0.74253300	0.88752900	-1.08199000
C	1.28186500	0.00122700	0.75163600
H	0.74253300	0.88752800	1.08199200
C	-2.60220800	0.15321400	-0.47862300
C	-3.46568100	-0.92897700	-0.01714400
C	-3.22202500	1.46673100	-0.63550900
C	-4.76531500	-0.72382400	0.26528400
H	-3.03932700	-1.91753000	0.10304800
C	-4.52001100	1.68500400	-0.35804900
H	-2.59076000	2.27768500	-0.98698800
C	-5.39993100	0.60253500	0.12119200
H	-5.41135500	-1.52282000	0.61047800

H	-4.97912400	2.66009600	-0.47164400
C	2.60220800	0.15321300	0.47862300
C	3.46568100	-0.92897700	0.01714300
C	3.22202600	1.46673000	0.63551000
C	4.76531500	-0.72382400	-0.26528500
H	3.03932700	-1.91753000	-0.10304900
C	4.52001100	1.68500300	0.35805000
H	2.59076000	2.27768400	0.98699100
C	5.39993100	0.60253500	-0.12119400
H	5.41135500	-1.52281900	-0.61048000
H	4.97912400	2.66009500	0.47164600
O	-6.58179100	0.78685000	0.38199300
O	6.58179000	0.78685000	-0.38199400
H	-1.08588700	-2.11754100	-0.56977700
C	0.42227100	-1.39411300	-1.91342600
H	-0.20318700	-1.43768600	-2.80776800
H	1.11693000	-0.55739300	-2.02312700
H	1.01012000	-2.31456100	-1.86608200
C	-0.42227100	-1.39411400	1.91342600
H	-1.11693000	-0.55739400	2.02312800
H	-1.01012000	-2.31456300	1.86608100
H	0.20318700	-1.43768800	2.80776800

QM dimer meso compressed conformation

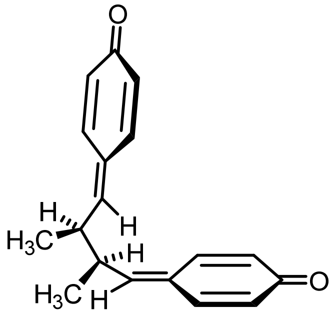

DFT Enthalpy = -847.022697 DFT Free Energy = -847.092051
CBS-QB3 Enthalpy = -845.605644 CBS-QB3 Free Energy = -845.675314

0 1

C	-0.64232500	2.01491100	-0.69332800
H	-0.41344200	1.28494400	-1.47326300
C	0.63359700	2.07183600	0.22880000
H	1.41792200	2.51352600	-0.39091700
C	1.03685300	0.68866500	0.64359700
H	0.29889300	0.16066500	1.24538000
C	-1.87583000	1.59017900	0.04742200
H	-2.28227100	2.33767500	0.72663400
C	2.19057300	0.02574700	0.37167000
C	3.28925500	0.58121600	-0.41177000
C	2.37212200	-1.33030500	0.88405600

C	4.41230700	-0.12102700	-0.65097300
H	3.19265000	1.58622200	-0.80577500
C	3.48915900	-2.04307400	0.65172200
H	1.56008200	-1.75377700	1.46755000
C	4.60543900	-1.49302700	-0.13870300
H	5.23115500	0.28860300	-1.23147700
H	3.61941500	-3.04950900	1.03235800
C	-2.55654300	0.41783800	-0.03742500
C	-2.16184600	-0.69657900	-0.89333800
C	-3.76089100	0.23098600	0.76838800
C	-2.86926400	-1.84115600	-0.93313300
H	-1.27305700	-0.59406800	-1.50455900
C	-4.47767100	-0.90670500	0.73627500
H	-4.06556200	1.05565900	1.40611900
C	-4.08662800	-2.04138000	-0.12088900
H	-2.57841600	-2.67166900	-1.56625200
H	-5.37092400	-1.04225400	1.33505300
O	5.62887400	-2.12815900	-0.35920800
O	-4.72775000	-3.08367100	-0.15945000
C	0.46242300	2.96368600	1.47593800
H	1.41297800	3.05128800	2.00614000
H	0.13763300	3.97117300	1.20574400
H	-0.26666000	2.54458100	2.17410100
C	-0.88914500	3.36869200	-1.38909900
H	-1.70666100	3.28406900	-2.10852700
H	-1.15657100	4.15141300	-0.67478700
H	0.00432400	3.69308800	-1.92951800

EmpiricalDispersion = GD3

DFT Enthalpy = -847.059972 DFT Free Energy = -847.127865

0 1

C	-0.64358300	2.21515700	-0.75508300
H	-0.08833100	1.53864800	-1.40927000
C	0.32631100	2.52835000	0.44923900
H	1.19333300	3.02511100	0.00152900
C	0.76525700	1.24186000	1.08886700
H	0.16917400	0.91260600	1.93704700
C	-1.90007000	1.53916800	-0.29059500
H	-2.66922800	2.20786000	0.09156700
C	1.75862800	0.41762500	0.67285000
C	2.60542800	0.70344700	-0.48012800
C	1.97821000	-0.85420700	1.35400100
C	3.52662200	-0.17685700	-0.91396400
H	2.47869800	1.64804500	-0.99694100
C	2.89652200	-1.74261900	0.93219000
H	1.35348800	-1.07424300	2.21420700
C	3.74627500	-1.47576400	-0.24292800
H	4.15720800	0.02530200	-1.77216400
H	3.05098600	-2.69314300	1.42908600

C	-2.17333100	0.21003300	-0.26447900
C	-1.24564200	-0.82013400	-0.72330900
C	-3.45275900	-0.24866400	0.27138000
C	-1.54253800	-2.12950900	-0.62910000
H	-0.29138600	-0.51616900	-1.13419000
C	-3.76240100	-1.55393400	0.36768300
H	-4.15637900	0.50967800	0.60256500
C	-2.82360600	-2.60524400	-0.06954800
H	-0.85329500	-2.89736500	-0.96071900
H	-4.70877700	-1.89535300	0.77070500
O	4.58392600	-2.27420600	-0.64237900
O	-3.09004800	-3.79671000	0.02304600
C	-0.27589800	3.47655600	1.49688700
H	0.45261600	3.67963100	2.28527300
H	-0.56258000	4.43312900	1.05657700
H	-1.16052400	3.03746800	1.96653000
C	-0.97375900	3.47980900	-1.56436300
H	-1.53608600	3.21892200	-2.46396100
H	-1.57912500	4.18371600	-0.98789100
H	-0.05882200	3.99302900	-1.87288500

QM dimer (S,S) compressed conformation

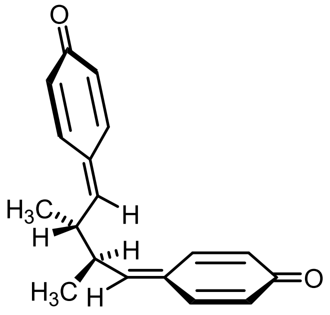

DFT Enthalpy = -847.023464 DFT Free Energy = -847.092929
CBS-QB3 Enthalpy = -845.606445 CBS-QB3 Free Energy = -845.676227

0 1

C	0.77352200	2.22589000	-0.10587000
H	1.23736300	2.28772100	0.88356100
C	-0.77352600	2.22589800	0.10585800
C	-1.19946200	0.95836900	0.79139900
H	-0.64010400	0.72200900	1.69599200
C	1.19944800	0.95835000	-0.79139500
H	0.64007700	0.72197300	-1.69597700
C	-2.19853300	0.10459400	0.45084000
C	-3.05526400	0.27800700	-0.71730400
C	-2.45724800	-1.06543100	1.28535900
C	-4.03762200	-0.59480100	-1.00886100
H	-2.89416500	1.13701300	-1.35840300
C	-3.43411600	-1.94669200	1.00411900
H	-1.82288000	-1.20596000	2.15543500

C	-4.30855000	-1.78098500	-0.17112200
H	-4.67835600	-0.46824100	-1.87415900
H	-3.62248700	-2.81493200	1.62491900
C	2.19852500	0.10458300	-0.45083500
C	3.05527200	0.27801800	0.71729500
C	2.45723100	-1.06545700	-1.28533800
C	4.03763800	-0.59478100	1.00885100
H	2.89417900	1.13703400	1.35838100
C	3.43410600	-1.94671000	-1.00409700
H	1.82285000	-1.20600200	-2.15540200
C	4.30855900	-1.78097800	0.17112800
H	4.67838300	-0.46820600	1.87413800
H	3.62247000	-2.81495900	-1.62488500
O	-5.20291500	-2.57284200	-0.44053600
O	5.20292900	-2.57282900	0.44054300
H	-1.23736800	2.28772000	-0.88357400
C	-1.24836300	3.43790700	0.93582700
H	-0.78426400	3.43988600	1.92684400
H	-1.00397400	4.38116600	0.44371700
H	-2.33088800	3.40308500	1.07435900
C	1.24836800	3.43788700	-0.93585300
H	0.78427000	3.43985700	-1.92687000
H	1.00398600	4.38115300	-0.44375400
H	2.33089300	3.40305500	-1.07438400

EmpiricalDispersion = GD3

DFT Enthalpy = -847.059277 DFT Free Energy = -847.129707

0 1

C	0.76431000	2.43093400	-0.16441800
H	1.30039500	2.48645800	0.78752500
C	-0.76431000	2.43093400	0.16441800
C	-1.11206700	1.16075800	0.88316900
H	-0.57712600	1.01836100	1.82168900
C	1.11206700	1.16075800	-0.88316900
H	0.57712600	1.01836100	-1.82168900
C	-1.97797000	0.18510800	0.51322700
C	-2.76344100	0.22104500	-0.71567400
C	-2.14277600	-0.98996200	1.36433000
C	-3.59614600	-0.78159100	-1.05094700
H	-2.67027500	1.08151600	-1.36785800
C	-2.97052600	-1.99969900	1.04033900
H	-1.56027100	-1.02858800	2.27998900
C	-3.76821000	-1.97685600	-0.19954100
H	-4.18316400	-0.76104900	-1.96200800
H	-3.08810300	-2.87422200	1.66943400
C	1.97797000	0.18510800	-0.51322700
C	2.76344100	0.22104500	0.71567400
C	2.14277600	-0.98996200	-1.36433000
C	3.59614600	-0.78159100	1.05094700

H	2.67027500	1.08151600	1.36785800
C	2.97052600	-1.99969900	-1.04033900
H	1.56027100	-1.02858800	-2.27998900
C	3.76821000	-1.97685600	0.19954100
H	4.18316300	-0.76104900	1.96200800
H	3.08810200	-2.87422200	-1.66943400
O	-4.52624600	-2.88733100	-0.50881700
O	4.52624600	-2.88733100	0.50881700
H	-1.30039500	2.48645800	-0.78752500
C	-1.17604500	3.64104800	1.02438100
H	-0.62147600	3.65437900	1.96780500
H	-0.98905700	4.58305900	0.50583400
H	-2.24116900	3.59402500	1.26095200
C	1.17604500	3.64104900	-1.02438100
H	0.62147600	3.65437900	-1.96780500
H	0.98905700	4.58305900	-0.50583400
H	2.24116900	3.59402500	-1.26095200

<u>Tautomerized QMD</u>

Partially-tautomerized QMD

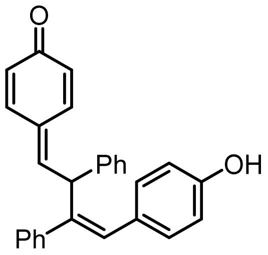

DFT Enthalpy = -1230.483599 DFT Free Energy = -1230.568525

0 1

C	1.45905300	-1.39936500	-1.58563400
C	2.43271200	-2.19042800	-0.95375900
C	3.57828900	-2.59632200	-1.63328300
C	3.78656600	-2.21609200	-2.95684000
C	2.83860300	-1.41870300	-3.59381000
C	1.69382000	-1.01272200	-2.91515700
H	2.28685600	-2.50969700	0.07014900
H	4.30813100	-3.21693100	-1.12511100
H	4.68054800	-2.53073900	-3.48308200
H	2.99597200	-1.09909900	-4.61803500
H	0.98132400	-0.36378600	-3.41069400
C	0.33391300	-0.36821300	0.52482000
H	-0.59256600	0.18194200	0.69816400
C	0.43382800	-1.39237000	1.66882300
C	0.00155000	-2.71402400	1.51523600
C	0.89948900	-0.98842300	2.92625300
C	0.03978700	-3.60712400	2.58486500

H	-0.36137100	-3.04955800	0.55063300
C	0.93965200	-1.88004000	3.99522700
H	1.22964800	0.03418100	3.07243400
C	0.51071500	-3.19485200	3.82860400
H	-0.29802500	-4.62756600	2.44187900
H	1.30511600	-1.54483700	4.95954000
H	0.54250000	-3.89014800	4.65967200
C	1.47530300	0.61933900	0.56044900
H	2.46987100	0.18326900	0.57797700
C	1.39570600	1.97475900	0.54917800
C	0.14449100	2.72425700	0.51333400
C	2.62854100	2.75765000	0.56707000
C	0.12907500	4.07044800	0.49832100
H	-0.79129000	2.17816600	0.49152700
C	2.62581100	4.10307400	0.55470900
H	3.56517000	2.20857700	0.58876000
C	1.36864000	4.87189900	0.51940700
H	-0.79862300	4.63073000	0.47035400
H	3.54270100	4.68112300	0.56742100
C	-2.32030700	-0.61938400	-1.14594600
C	-2.84900400	-0.74307700	0.15253100
C	-3.17643700	-0.11343500	-2.13886700
C	-4.14511900	-0.34485500	0.45060500
H	-2.25877800	-1.19583200	0.93849400
C	-4.47177500	0.29545200	-1.85107000
H	-2.81227300	-0.02682900	-3.15712400
C	-4.96265300	0.18761200	-0.54770600
H	-4.54602000	-0.45401800	1.45093600
H	-5.10374700	0.69360200	-2.63971700
O	1.34992000	6.09710800	0.50829500
O	-6.22465400	0.56594700	-0.19684300
H	-6.68253900	0.90980500	-0.97094000
C	0.21281400	-0.96497800	-0.88394500
C	-0.96209000	-1.02288400	-1.54664600
H	-0.91058500	-1.40001000	-2.56545900

Quad A

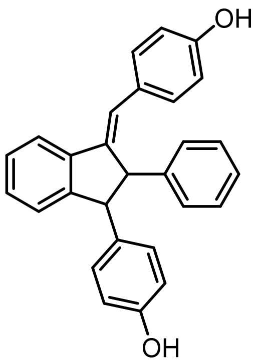

DFT Enthalpy = -1230.528876 DFT Free Energy = -1230.611236

0 1

C	1.90704200	1.41367000	-0.89972400
C	3.07977900	2.01891900	-1.33953800
C	1.92591400	2.72594900	-3.34807400
C	0.75394100	2.11650900	-2.91335100
H	3.98055500	1.97530100	-0.73650500
H	1.93948000	3.24890000	-4.29798100
H	-0.14310100	2.17258200	-3.52023200
C	0.74422100	1.45179500	-1.68072600
C	3.08559600	2.67864400	-2.56734900
H	3.99091200	3.16134700	-2.91801400
C	1.68533900	0.64077400	0.38567900
H	1.98438200	1.24956900	1.24254400
C	-0.34510900	0.72573000	-1.00913200
C	0.11058700	0.44681000	0.42178700
H	-0.11388900	-0.58769500	0.69194200
C	-0.50689000	1.35389800	1.48126500
C	-0.58524800	0.90345000	2.80579900
C	-0.95349900	2.64718200	1.19322300
C	-1.09220900	1.72007700	3.81322700
H	-0.24886100	-0.10044900	3.04675900
C	-1.45896900	3.46708300	2.20060000
H	-0.91518900	3.01307100	0.17410800
C	-1.53039500	3.00852200	3.51357100
H	-1.14965000	1.34825600	4.83049500
H	-1.80269800	4.46615600	1.95568900
H	-1.92899300	3.64593300	4.29476600
C	2.45988100	-0.66542800	0.46212500
C	3.19666900	-0.98558900	1.60352100
C	2.44200100	-1.59690300	-0.58620100
C	3.89101200	-2.19032400	1.70770200
H	3.23294900	-0.28348400	2.43031800
C	3.12484500	-2.80141300	-0.49555700
H	1.88825100	-1.37429800	-1.49154200
C	3.85548000	-3.10458800	0.65634200
H	4.46036200	-2.41413200	2.60562700

H	3.10945000	-3.51868800	-1.30732700
C	-1.47001600	0.31920300	-1.63601200
H	-1.53008000	0.57177700	-2.69292800
C	-2.61388800	-0.46538100	-1.17434300
C	-3.45947800	-1.02784100	-2.14826700
C	-2.94931200	-0.70308600	0.17365800
C	-4.55808000	-1.80572400	-1.80925200
H	-3.24204600	-0.85618700	-3.19734400
C	-4.04636800	-1.47534200	0.52352400
H	-2.36898200	-0.25212300	0.96500700
C	-4.85567500	-2.03891000	-0.46516900
H	-5.18607000	-2.22957900	-2.58789500
H	-4.29963300	-1.64399800	1.56322800
O	-5.92014700	-2.79162300	-0.06072300
H	-6.39329900	-3.11351500	-0.83496300
O	4.51258100	-4.30316700	0.69035400
H	4.97365200	-4.38651400	1.53122800

C–H HAT radical product

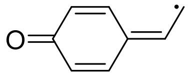

DFT Enthalpy = -384.199024 DFT Free Energy = -384.240577

CBS-QB3 Enthalpy = -383.550505 CBS-QB3 Free Energy = -383.592169

0 2

C	0.05400900	1.35511900	0.00000000
C	-1.30438300	1.20396400	0.00000000
C	-1.91583900	-0.11808800	-0.00000500
C	-0.99552900	-1.25061100	-0.00000100
C	0.35602700	-1.07221400	0.00000000
H	0.48958600	2.34944800	0.00000200
H	1.00793700	-1.93797200	0.00000100
C	0.93178600	0.23415300	0.00000000
C	2.36009900	0.46617500	0.00000000
H	2.65571000	1.51258600	0.00000100
O	-3.14876800	-0.27398600	0.00000200
C	3.33704300	-0.46115300	0.00000000
H	3.13647900	-1.52590900	0.00000000
H	4.37891200	-0.16612000	0.00000100
H	-1.44149000	-2.23839700	0.00000100
H	-1.97626200	2.05418400	0.00000100

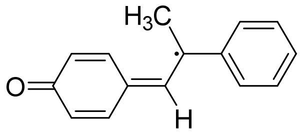

DFT Enthalpy = -654.518714 DFT Free Energy = -654.575525

CBS-QB3 Enthalpy = -653.393246 CBS-QB3 Free Energy = -653.450284

0 2

C	1.52663700	-0.17878300	-0.06031300
C	2.26612700	1.04646900	-0.05163000
C	3.63125800	1.05609200	-0.05797800
C	4.41372500	-0.17389500	-0.07235200
C	3.64436100	-1.41088100	-0.11084300
C	2.28208000	-1.39605400	-0.11637600
H	1.74048500	1.98910300	-0.07297000
H	4.18983800	1.98508300	-0.06360700
H	4.20631700	-2.33724000	-0.13798000
H	1.73406700	-2.33262400	-0.14873300
C	0.09559100	-0.30235800	-0.05597100
H	-0.23773000	-1.30730800	-0.29735500
C	-0.90681300	0.59889900	0.18954500
C	-2.31412300	0.15072700	0.07608700
C	-3.30656300	1.03105100	-0.39201800
C	-2.71066100	-1.15268400	0.42751200
C	-4.62699500	0.61798900	-0.53178300
H	-3.03832200	2.04156900	-0.67632200
C	-4.03301100	-1.56037100	0.29989500
H	-1.98216700	-1.84097800	0.83868200
C	-4.99806500	-0.67954400	-0.18603900
H	-5.36792600	1.31206600	-0.91227100
H	-4.31363000	-2.56608300	0.59198900
H	-6.02906600	-0.99866600	-0.28584100
O	5.65560200	-0.16513300	-0.06495700
C	-0.67848700	2.04011600	0.58017800
H	0.15428900	2.12915000	1.27928800
H	-0.44885500	2.67090900	-0.28670000
H	-1.56247000	2.45544600	1.06398500

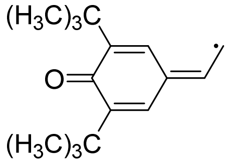

DFT Enthalpy = -698.559574 DFT Free Energy = -698.624394

CBS-QB3 Enthalpy = -697.369115 CBS-QB3 Free Energy = -697.434257

0 2

C	1.07613400	1.48438700	0.00000100
C	1.32845900	0.13521400	0.00000200
C	0.17361600	-0.79071000	0.00000300
C	-1.19792600	-0.22608700	0.00000000
C	-1.34270100	1.13509900	0.00000000
H	1.89747300	2.18964400	0.00000000
H	-2.33391700	1.56526200	-0.00000100
C	-0.23419900	2.02394800	0.00000000
C	-0.38759600	3.46459800	-0.00000100
H	0.54473000	4.02453300	-0.00000200
O	0.35025700	-2.02238400	0.00000500
C	2.75526800	-0.43149500	0.00000000
C	2.97968000	-1.29459200	-1.26720400
H	4.00736900	-1.67050400	-1.27710900
H	2.29856800	-2.14274800	-1.29159000
H	2.83197500	-0.69907900	-2.17319000
C	3.81665500	0.68580600	-0.00000100
H	3.74640200	1.32113600	-0.88759800
H	3.74641100	1.32113000	0.88760100
H	4.81117000	0.23292900	-0.00000700
C	2.97968500	-1.29459500	1.26720100
H	2.29857300	-2.14275100	1.29158700
H	4.00737500	-1.67050700	1.27710000
H	2.83198400	-0.69908400	2.17318800
C	-2.40461400	-1.17680700	-0.00000100
C	-2.37435500	-2.06769100	-1.26730400
H	-1.48041300	-2.68741200	-1.29201300
H	-3.25273300	-2.72030700	-1.27755600
H	-2.40198000	-1.45444300	-2.17310100
C	-3.74090100	-0.40908300	-0.00000200
H	-4.56459700	-1.12718400	-0.00000400
H	-3.85535200	0.21954000	0.88787700
H	-3.85534800	0.21954400	-0.88787800
C	-2.37435800	-2.06769400	1.26730100
H	-1.48041500	-2.68741300	1.29201300
H	-2.40199100	-1.45444700	2.17309900
H	-3.25273500	-2.72031300	1.27754600
C	-1.53741300	4.16593200	0.00000300
H	-2.51337700	3.69538100	0.00000500
H	-1.52382800	5.24878300	0.00000200

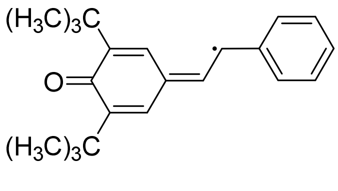

DFT Enthalpy = -929.590865 DFT Free Energy = -929.668109

0 2

C	0.24438200	-0.30497200	-0.00000700

C	-0.28203700	1.01944900	-0.00001300
C	-1.62299200	1.28289100	-0.00000900
C	-2.57169300	0.14101700	0.00000500
C	-2.02784700	-1.23677700	0.00000700
C	-0.66793400	-1.39626800	0.00000100
H	0.41879000	1.84178000	-0.00002500
H	-0.23907800	-2.39044400	0.00000300
C	1.64828700	-0.59251400	-0.00001000
H	1.89423400	-1.65079400	-0.00001800
C	2.67304400	0.30713600	0.00000500
H	2.43396400	1.36636200	0.00003000
C	4.09673800	0.01130700	0.00000000
C	5.00906800	1.08479200	0.00008800
C	4.62324700	-1.29679600	-0.00009100
C	6.38099000	0.86658100	0.00009300
H	4.62606000	2.09997600	0.00015700
C	5.99321000	-1.51271600	-0.00008700
H	3.95630900	-2.15034400	-0.00017000
C	6.88081700	-0.43417700	0.00000500
H	7.06107000	1.71080700	0.00016300
H	6.37577800	-2.52719100	-0.00016000
H	7.95050300	-0.60882400	0.00000700
O	-3.79875200	0.33818900	0.00001400
C	-2.99370300	-2.43098900	0.00001800
C	-3.88442200	-2.38876000	-1.26691800
H	-4.55064300	-3.25698800	-1.27635100
H	-4.48989900	-1.48507000	-1.29188000
H	-3.27177400	-2.42638100	-2.17278600
C	-2.24301000	-3.77682000	0.00001400
H	-1.61550000	-3.89870900	-0.88765200
H	-1.61548200	-3.89870400	0.88766800
H	-2.97122700	-4.59169600	0.00002400
C	-3.88440000	-2.38875400	1.26696800
H	-4.48987700	-1.48506400	1.29193700
H	-4.55062200	-3.25698100	1.27641600
H	-3.27173600	-2.42637100	2.17282600
C	-2.17402300	2.71742400	-0.00001800
C	-3.03385300	2.95229100	1.26717600
H	-3.39938000	3.98382100	1.27688000
H	-3.88889900	2.27997500	1.29264300
H	-2.43915000	2.79886200	2.17282000
C	-3.03387400	2.95226600	-1.26720300
H	-3.88892000	2.27994900	-1.29264400
H	-3.39940200	3.98379600	-1.27692100
H	-2.43918500	2.79882100	-2.17285400
C	-1.04577400	3.76714400	-0.00003800
H	-0.41150600	3.69050100	0.88799300
H	-0.41151900	3.69048100	-0.88807600
H	-1.48821700	4.76638600	-0.00004600

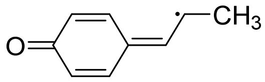

DFT Enthalpy = -423.500577 DFT Free Energy = -423.546003

CBS-QB3 Enthalpy = -422.780327 CBS-QB3 Free Energy = -422.825892

0 2

C	-0.36876800	0.24738400	0.00001200
C	0.19820100	-1.06377500	0.00001500
C	1.54807600	-1.25387100	0.00000800
C	2.48005200	-0.13111400	-0.00000500
C	1.87902600	1.19548500	-0.00000600
C	0.52251500	1.35908400	0.00000400
H	-0.45909400	-1.92557500	0.00002600
H	1.98422800	-2.24613100	0.00001200
H	2.55786100	2.04027500	-0.00001400
H	0.09706000	2.35790900	0.00000400
C	-1.79102900	0.49721200	0.00001500
H	-2.07554000	1.54788500	0.00004000
C	-2.78568400	-0.41681200	-0.00001300
H	-2.54217400	-1.47610100	-0.00004300
O	3.71155500	-0.29873100	-0.00001400
C	-4.24216600	-0.08166400	-0.00001000
H	-4.41166900	0.99702600	0.00002600
H	-4.74222000	-0.50847600	-0.87732300
H	-4.74223400	-0.50853900	0.87726300

EmpiricalDispersion = GD3

DFT Enthalpy = -423.512567 DFT Free Energy = -423.558010

0 2

C	0.36887000	0.24874000	-0.00000400
C	-0.19727500	-1.06320100	-0.00000200
C	-1.54688400	-1.25422300	0.00000000
C	-2.47959500	-0.13180000	-0.00000200
C	-1.87948000	1.19544700	-0.00000300
C	-0.52313300	1.36025500	-0.00000500
H	0.46019900	-1.92473900	-0.00000200
H	-1.98403900	-2.24589800	0.00000200
H	-2.56035700	2.03842100	-0.00000300
H	-0.09966100	2.35995800	-0.00000700
C	1.79094300	0.49830400	-0.00000400
H	2.07751400	1.54852700	-0.00001500
C	2.78463900	-0.41659100	0.00000800
H	2.54141400	-1.47588500	0.00002000
O	-3.71085300	-0.30014300	0.00000300
C	4.24154400	-0.08259500	0.00000700
H	4.41366300	0.99594000	-0.00000700
H	4.74015500	-0.51059100	0.87761300

H	4.74016000	-0.51061600	-0.87758400

trans-4-hydroxystilbene radical X = H

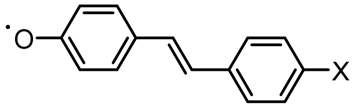

DFT Enthalpy = -615.230500 DFT Free Energy = -615.284489

CBS-QB3 Enthalpy = -614.170513 CBS-QB3 Free Energy = -614.224704

0 2

C	1.50410500	0.24049200	0.00000100
C	2.07863100	-1.07154100	0.00000000
C	3.42687700	-1.25630100	0.00000000
C	4.35832700	-0.12991200	0.00000000
C	3.75148300	1.19668700	0.00000000
C	2.39737600	1.35625200	0.00000000
H	1.42564200	-1.93650100	0.00000000
H	3.86640500	-2.24709000	-0.00000100
H	4.42810000	2.04325800	0.00000000
H	1.96898300	2.35395900	0.00000000
C	0.09475200	0.48822300	0.00000100
H	-0.18251500	1.53859300	0.00000400
C	-0.90178000	-0.44338100	-0.00000100
H	-0.62849600	-1.49435100	-0.00000200
C	-2.33349400	-0.19291900	0.00000000
C	-3.21103200	-1.29511700	0.00000000
C	-2.90067700	1.09815400	-0.00000100
C	-4.58904600	-1.12037900	0.00000000
H	-2.79606100	-2.29761700	0.00000000
C	-4.27666100	1.27051800	0.00000000
H	-2.26114900	1.97241300	-0.00000200
C	-5.12939200	0.16411200	0.00000000
H	-5.24223900	-1.98549400	0.00000100
H	-4.69138400	2.27220800	-0.00000100
H	-6.20404400	0.30484000	0.00000100
O	5.58749300	-0.29419400	0.00000000

EmpiricalDispersion = GD3

DFT Enthalpy = -615.249220 DFT Free Energy = -615.303304

0 2

C	1.50318000	0.24138800	0.00000000
C	2.07720600	-1.07130400	-0.00000100
C	3.42519800	-1.25662000	-0.00000100
C	4.35703100	-0.13022800	0.00000000
C	3.75072600	1.19689200	0.00000100

C	2.39676400	1.35729500	0.00000100
H	1.42409400	-1.93607600	-0.00000100
H	3.86606900	-2.24666700	-0.00000100
H	4.42920100	2.04180200	0.00000100
H	1.96998600	2.35574500	0.00000100
C	0.09418800	0.48827100	0.00000000
H	-0.18532100	1.53802200	0.00000100
C	-0.90108400	-0.44458000	0.00000000
H	-0.62601100	-1.49507700	-0.00000100
C	-2.33232300	-0.19428800	0.00000000
C	-3.21082700	-1.29593700	0.00000000
C	-2.89840000	1.09751900	-0.00000100
C	-4.58877400	-1.11967200	0.00000100
H	-2.79794800	-2.29935100	0.00000100
C	-4.27419300	1.27128000	-0.00000100
H	-2.25818600	1.97117000	-0.00000200
C	-5.12809200	0.16547400	0.00000000
H	-5.24280900	-1.98412300	0.00000100
H	-4.68798000	2.27333200	-0.00000100
H	-6.20260000	0.30712400	0.00000000
O	5.58599000	-0.29485600	0.00000000

trans-4-hydroxystilbene radical X = NO_2

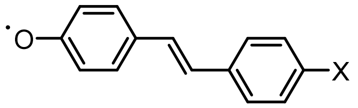

DFT Enthalpy = -819.780437 DFT Free Energy = -819.841307

CBS-QB3 Enthalpy = -818.460523 CBS-QB3 Free Energy = -818.521636

0 2

C	-2.72613300	0.23212400	-0.00000300
C	-3.33312800	-1.06555400	0.00003200
C	-4.68528900	-1.21445400	0.00003100
C	-5.58521400	-0.06163100	-0.00000600
C	-4.94468500	1.25001200	-0.00004000
C	-3.58715500	1.37321500	-0.00003800
H	-2.70307200	-1.94716100	0.00006200
H	-5.15199100	-2.19255400	0.00005800
H	-5.59917400	2.11360300	-0.00006700
H	-3.13165400	2.35862400	-0.00006400
C	-1.31083200	0.44248200	-0.00000600
H	-1.00637000	1.48510000	-0.00002800
C	-0.33998300	-0.51614300	0.00001300
H	-0.63819800	-1.55973600	0.00002500
C	1.09544800	-0.29696100	0.00001100
C	1.94832700	-1.42060600	-0.00001200
C	1.68980600	0.98374200	0.00003300
C	3.32705600	-1.28392500	-0.00001800
H	1.51363000	-2.41376000	-0.00002800

C	3.06492000	1.13359900	0.00002800
H	1.07166000	1.87222100	0.00005800
C	3.87074600	-0.00377100	0.00000200
H	3.98535400	-2.14126400	-0.00003700
H	3.52934900	2.10971300	0.00004600
O	-6.81709300	-0.19282400	-0.00000700
N	5.33732100	0.15510300	-0.00000400
O	5.78318000	1.29543000	0.00001500
O	6.01715000	-0.86301700	-0.00002700

EmpiricalDispersion = GD3

DFT Enthalpy = -819.802820 DFT Free Energy = -819.863789

0 2

C	2.72520600	0.23308300	-0.00000100
C	3.33187200	-1.06520800	0.00000600
C	4.68380500	-1.21451600	0.00000700
C	5.58400100	-0.06157500	0.00000100
C	4.94383800	1.25054700	-0.00000800
C	3.58643000	1.37443700	-0.00000900
H	2.70181000	-1.94671800	0.00001200
H	5.15190800	-2.19180300	0.00001300
H	5.60010400	2.11262100	-0.00001400
H	3.13241700	2.36058500	-0.00001500
C	1.31028400	0.44247900	-0.00000200
H	1.00355900	1.48441600	-0.00000800
C	0.34084100	-0.51748800	0.00000200
H	0.64100800	-1.56051600	0.00000700
C	-1.09413900	-0.29881900	0.00000100
C	-1.94769000	-1.42209400	0.00000500
C	-1.68786300	0.98236800	-0.00000300
C	-3.32624600	-1.28433800	0.00000600
H	-1.51501100	-2.41614700	0.00000800
C	-3.06266400	1.13320700	-0.00000500
H	-1.06954500	1.87059200	-0.00000600
C	-3.86955400	-0.00375500	0.00000100
H	-3.98449300	-2.14168500	0.00001200
H	-3.52569900	2.10995300	-0.00001100
O	6.81567800	-0.19298000	0.00000000
O	-6.01813200	-0.86158100	0.00001400
O	-5.78299000	1.29660800	-0.00001500
N	-5.33732000	0.15605400	0.00000000

trans-4-hydroxystilbene radical X = CF_3

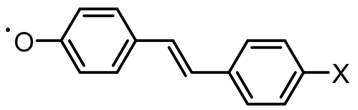

DFT Enthalpy = -952.360256 DFT Free Energy = -952.424828

CBS-QB3 Enthalpy = -950.922217 CBS-QB3 Free Energy = -950.987052

0 2

C	3.16828200	-0.23208700	-0.00271500
C	3.78307100	1.06147100	0.00299300
C	5.13636800	1.20297400	0.00969700
C	6.03026500	0.04613500	0.01142300
C	5.38210900	-1.26114400	0.00557500
C	4.02360800	-1.37697200	-0.00111100
H	3.15799900	1.94670600	0.00195300
H	5.60798900	2.17877000	0.01403600
H	6.03153500	-2.12862200	0.00681300
H	3.56306000	-2.36011000	-0.00537400
C	1.75101400	-0.43447500	-0.00992300
H	1.44081800	-1.47551300	-0.01368800
C	0.78586800	0.52862600	-0.01222800
H	1.09023200	1.57066000	-0.00825300
C	-0.65284700	0.31944100	-0.01884800
C	-1.49873900	1.44505500	-0.02070600
C	-1.25737700	-0.95461200	-0.02753300
C	-2.87995500	1.31294800	-0.02748400
H	-1.05899000	2.43629700	-0.01882200
C	-2.63485400	-1.09037200	-0.03456400
H	-0.64611400	-1.84819000	-0.03148500
C	-3.45413000	0.04288100	-0.03470100
H	-3.51214400	2.19147800	-0.03489200
H	-3.08251300	-2.07625700	-0.04937500
O	7.26351000	0.17066800	0.01748000
C	-4.94790900	-0.11833600	0.01096600
F	-5.36144100	-1.18827500	-0.70314700
F	-5.39164800	-0.30320000	1.27682400
F	-5.59231100	0.96372400	-0.47432100

EmpiricalDispersion = GD3

DFT Enthalpy = -952.383664 DFT Free Energy = -952.448189

0 2

C	3.16582000	-0.23306100	-0.00273100
C	3.78017600	1.06115800	0.00298900
C	5.13323600	1.20318700	0.01028700
C	6.02750400	0.04631700	0.01264800
C	5.37982600	-1.26148700	0.00680000
C	4.02145100	-1.37812300	-0.00049300
H	3.15501800	1.94623900	0.00146000
H	5.60616800	2.17820400	0.01464100
H	6.03111200	-2.12740300	0.00854400
H	3.56246900	-2.36204600	-0.00474800
C	1.74895600	-0.43461400	-0.01055300
H	1.43658500	-1.47497800	-0.01434100
C	0.78514400	0.52973400	-0.01333300
H	1.09142800	1.57119700	-0.00917800

C	-0.65315200	0.32101100	-0.02059200
C	-1.49938700	1.44689800	-0.02223400
C	-1.25650400	-0.95396600	-0.03020400
C	-2.88039000	1.31354500	-0.02942900
H	-1.06094100	2.43873900	-0.01972900
C	-2.63381500	-1.09055000	-0.03764000
H	-0.64445500	-1.84687300	-0.03451800
C	-3.45178500	0.04278500	-0.03774700
H	-3.51595600	2.18963000	-0.03682200
H	-3.08422900	-2.07507500	-0.05346600
O	7.26054300	0.17117600	0.01928000
C	-4.94475300	-0.11908100	0.01144900
F	-5.59052000	0.95546900	-0.49022600
F	-5.35730300	-1.20161700	-0.68494300
F	-5.38501100	-0.28380300	1.28168200

trans-4-hydroxystilbene radical X = CO_2H

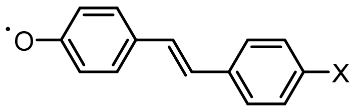

DFT Enthalpy = -803.843145 DFT Free Energy = -803.904323

CBS-QB3 Enthalpy = -802.533646 CBS-QB3 Free Energy = -802.595074

0 2

C	2.75029700	0.23425400	0.00006900
C	3.36472900	-1.05990400	0.00011900
C	4.71776900	-1.20213800	0.00016100
C	5.61271300	-0.04586500	0.00015900
C	4.96512300	1.26195200	0.00010600
C	3.60695800	1.37869100	0.00006400
H	2.73902400	-1.94467300	0.00012200
H	5.18877400	-2.17825700	0.00019800
H	5.61517100	2.12898700	0.00010300
H	3.14690300	2.36208400	0.00002600
C	1.33432000	0.43758000	0.00002400
H	1.02414600	1.47857000	-0.00001300
C	0.36751000	-0.52557700	0.00002400
H	0.67181200	-1.56768300	0.00006300
C	-1.06924400	-0.31580200	-0.00002300
C	-1.91579700	-1.44399900	0.00000100
C	-1.67308400	0.95941200	-0.00009100
C	-3.29363900	-1.30963500	-0.00003900
H	-1.47383100	-2.43447900	0.00005400
C	-3.05017900	1.09627300	-0.00013200
H	-1.05976800	1.85181800	-0.00011500
C	-3.87519700	-0.03754000	-0.00010600
H	-3.94099900	-2.17760200	-0.00002100
H	-3.49961600	2.08045100	-0.00018400
O	6.84572600	-0.17113100	0.00019600

O	-6.10949000	-0.88905500	-0.00013300
C	-5.35765600	0.05637600	-0.00014900
O	-5.80471400	1.33653100	-0.00020400
H	-6.77153000	1.28554200	-0.00022600

EmpiricalDispersion = GD3

DFT Enthalpy = -803.866255 DFT Free Energy = -803.927466

0 2

C	2.75311300	0.23423800	-0.00007600
C	3.35587700	-1.06584900	-0.00007500
C	4.70738200	-1.22034100	-0.00010800
C	5.61266700	-0.07181600	-0.00014700
C	4.97691300	1.24206400	-0.00014700
C	3.61992700	1.37140100	-0.00011400
H	2.72242900	-1.94500900	-0.00004600
H	5.17129500	-2.19971200	-0.00010700
H	5.63625800	2.10187900	-0.00017600
H	3.16997600	2.35949300	-0.00011500
C	1.33931700	0.44889700	-0.00004200
H	1.03572300	1.49176500	-0.00004800
C	0.36567900	-0.50729100	-0.00000300
H	0.66290700	-1.55143700	0.00000500
C	-1.06857200	-0.28483400	0.00003200
C	-1.92561600	-1.40432700	0.00007400
C	-1.66017200	0.99731600	0.00002600
C	-3.30404800	-1.25913400	0.00010900
H	-1.49399600	-2.39941100	0.00007800
C	-3.03425000	1.14449200	0.00006000
H	-1.03885000	1.88399700	-0.00000600
C	-3.87118500	0.01970500	0.00010200
H	-3.94666800	-2.12943400	0.00014100
H	-3.48964200	2.12698600	0.00005500
O	6.84433800	-0.20821100	-0.00017700
O	-5.87333300	1.32549800	0.00014200
O	-6.04719100	-0.91591400	0.00016700
H	-6.98217000	-0.66544900	0.00018800
C	-5.33999500	0.24136800	0.00013800

trans-4-hydroxystilbene radical X = Cl

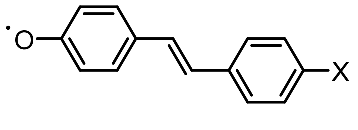

DFT Enthalpy = -1074.862150 DFT Free Energy = -1074.919541

CBS-QB3 Enthalpy = -1073.332248 CBS-QB3 Free Energy = -1073.389860

0 2

C	-2.42557500	-0.23670900	0.00000200

C	-3.02761300	1.06300500	0.00001500
C	-4.37936500	1.21880900	0.00001400
C	-5.28618100	0.07219000	-0.00000300
C	-4.65128200	-1.24145500	-0.00001400
C	-3.29416300	-1.37185100	-0.00001200
H	-2.39345000	1.94185000	0.00002800
H	-4.84026600	2.19978700	0.00002500
H	-5.30967100	-2.10221700	-0.00002500
H	-2.84440800	-2.36007700	-0.00002100
C	-1.01141300	-0.45404600	0.00000100
H	-0.71218500	-1.49843700	-0.00000500
C	-0.03507800	0.49888500	0.00000500
H	-0.33002700	1.54376300	0.00000700
C	1.40012500	0.27767600	0.00000400
C	2.25835900	1.39476200	-0.00001100
C	1.99564400	-1.00055400	0.00001800
C	3.63908100	1.25526400	-0.00001500
H	1.82949400	2.39096900	-0.00002200
C	3.37250000	-1.15376600	0.00001600
H	1.37816200	-1.89012700	0.00003300
C	4.18943600	-0.02288500	-0.00000100
H	4.28441600	2.12399400	-0.00002700
H	3.81810800	-2.14008700	0.00002700
O	-6.51823100	0.21001700	-0.00000700
Cl	5.92934300	-0.21679500	-0.00000500

EmpiricalDispersion = GD3

DFT Enthalpy = -1074.882856 DFT Free Energy = -1074.940323

0 2

C	-2.42429300	-0.23761500	0.00000000
C	-3.02602200	1.06269200	0.00000000
C	-4.37755200	1.21887200	0.00000000
C	-5.28460600	0.07212200	0.00000000
C	-4.65005300	-1.24197500	0.00000100
C	-3.29305000	-1.37303200	0.00000100
H	-2.39184900	1.94143600	-0.00000100
H	-4.83989400	2.19902700	-0.00000100
H	-5.31020500	-2.10121800	0.00000100
H	-2.84475800	-2.36197400	0.00000100
C	-1.01049600	-0.45391100	0.00000000
H	-0.70889200	-1.49759400	0.00000000
C	-0.03559100	0.50039500	0.00000000
H	-0.33244000	1.54472700	0.00000000
C	1.39913800	0.27958700	0.00000000
C	2.25801500	1.39641000	0.00000000
C	1.99362600	-0.99938300	-0.00000100
C	3.63851100	1.25569200	0.00000000
H	1.83093300	2.39340400	0.00000100
C	3.37010500	-1.15379200	0.00000000

H	1.37552400	-1.88838800	-0.00000100
C	4.18800800	-0.02317100	0.00000000
H	4.28565800	2.12299500	0.00000100
H	3.81617400	-2.13984000	-0.00000100
O	-6.51646700	0.21013700	0.00000000
Cl	5.92806100	-0.21829500	0.00000000

trans-4-hydroxystilbene radical X = SH

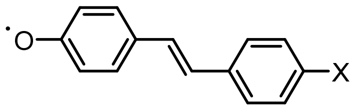

DFT Enthalpy = -1013.446695 DFT Free Energy = -1013.505580

CBS-QB3 Enthalpy = -1011.923422 CBS-QB3 Free Energy = -1011.982538

0 2

C	2.43432200	-0.23771900	0.00001200
C	3.03779300	1.06172600	0.00003000
C	4.38947800	1.21720500	0.00002300
C	5.29702200	0.07110300	-0.00000400
C	4.66111800	-1.24209700	-0.00001600
C	3.30423400	-1.37245100	-0.00000600
H	2.40393300	1.94088600	0.00005600
H	4.85017600	2.19835100	0.00003900
H	5.31922300	-2.10315900	-0.00003400
H	2.85461600	-2.36085900	-0.00001600
C	1.02184200	-0.45460000	0.00001300
H	0.72210000	-1.49894500	0.00002800
C	0.04365300	0.49888400	-0.00001000
H	0.33975000	1.54361100	-0.00003200
C	-1.38847200	0.27950200	-0.00001100
C	-2.25094700	1.39448900	-0.00002200
C	-1.98929400	-0.99670000	0.00000100
C	-3.62850100	1.25258800	-0.00001900
H	-1.82340100	2.39150600	-0.00003500
C	-3.36459100	-1.14739000	0.00000600
H	-1.37353900	-1.88782100	0.00000600
C	-4.20428500	-0.02382900	-0.00000200
H	-4.26062600	2.13328400	-0.00002700
H	-3.79054600	-2.14404400	0.00001400
O	6.52915000	0.20891400	-0.00001500
S	-5.97522000	-0.13813300	0.00000700
H	-6.05159400	-1.47825600	0.00003000

EmpiricalDispersion = GD3

DFT Enthalpy = -1013.468020 DFT Free Energy = -1013.526930

0 2

C	-2.43298400	-0.23800300	-0.00000500

C	-3.03616400	1.06202000	-0.00000700
C	-4.38762300	1.21788100	-0.00000300
C	-5.29538400	0.07162800	0.00000100
C	-4.65981200	-1.24200000	0.00000100
C	-3.30302900	-1.37302800	-0.00000200
H	-2.40229000	1.94108400	-0.00001200
H	-4.84981900	2.19818200	-0.00000600
H	-5.31964700	-2.10156400	0.00000400
H	-2.85485600	-2.36212900	-0.00000200
C	-1.02083200	-0.45377600	-0.00000500
H	-0.71845500	-1.49730000	-0.00001300
C	-0.04424200	0.50124800	0.00000400
H	-0.34232600	1.54542600	0.00001200
C	1.38731300	0.28209400	0.00000400
C	2.24987100	1.39638900	0.00000500
C	1.98770900	-0.99514500	0.00000300
C	3.62833100	1.25501900	0.00000200
H	1.82376000	2.39415100	0.00000600
C	3.36188800	-1.14546900	0.00000300
H	1.37154700	-1.88574600	0.00000500
C	4.20321200	-0.02114900	0.00000100
H	4.25636800	2.13818800	0.00000100
H	3.79092800	-2.14125800	0.00000400
O	-6.52732500	0.20970800	0.00000500
S	5.95657000	-0.30016400	-0.00000300
H	6.32874500	0.98967100	-0.00001600

trans-4-hydroxystilbene radical X = SMe

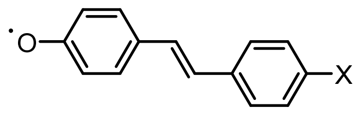

DFT Enthalpy = -1052.739377 DFT Free Energy = -1052.801696

CBS-QB3 Enthalpy = -1051.146610 CBS-QB3 Free Energy = -1051.209184

0 2

C	2.83169000	0.24099700	-0.00000400
C	3.49258400	-1.03035200	-0.00000200
C	4.84980200	-1.12585300	-0.00000100
C	5.70620800	0.05895500	-0.00000300
C	5.01249400	1.34255100	-0.00000300
C	3.65125900	1.41285000	-0.00000400
H	2.89826500	-1.93674400	-0.00000200
H	5.35327100	-2.08579100	0.00000000
H	5.63179500	2.23197300	-0.00000200
H	3.15846400	2.38052100	-0.00000400
C	1.41174500	0.39520200	-0.00000400
H	1.06606800	1.42530300	-0.00000500
C	0.47561500	-0.60059400	-0.00000400
H	0.81795100	-1.63118100	-0.00000300

C	-0.96377900	-0.44517900	-0.00000400
C	-1.77768500	-1.59883700	-0.00000300
C	-1.62211000	0.80037200	-0.00000500
C	-3.15713800	-1.51713200	-0.00000200
H	-1.30611900	-2.57593900	-0.00000200
C	-3.00485200	0.89178600	-0.00000500
H	-1.04691200	1.71840800	-0.00000600
C	-3.79660100	-0.26629200	-0.00000300
H	-3.75074500	-2.42461100	0.00000100
H	-3.46258400	1.87185300	-0.00000700
O	6.94329300	-0.02429800	0.00000200
C	-6.01734700	1.47962700	0.00003900
H	-5.65631900	1.98409100	0.89682700
H	-5.65634700	1.98413200	-0.89673800
H	-7.10757500	1.49570300	0.00005600
S	-5.56480400	-0.27949600	-0.00000600

EmpiricalDispersion = GD3

DFT Enthalpy = -1052.763329 DFT Free Energy = -1052.825529

0 2

C	-2.86920100	-0.23165800	0.00000500
C	-3.43027600	1.08716700	0.00004300
C	-4.77596500	1.28689400	0.00003800
C	-5.72090900	0.17114200	-0.00000900
C	-5.12805900	-1.16226100	-0.00004400
C	-3.77631300	-1.33745500	-0.00003500
H	-2.76814400	1.94519700	0.00008200
H	-5.20585500	2.28182500	0.00006900
H	-5.81541900	-2.00002100	-0.00007800
H	-3.36068400	-2.34071500	-0.00006200
C	-1.46538100	-0.49313500	0.00000500
H	-1.19684400	-1.54587900	-0.00000400
C	-0.45747600	0.42972500	0.00000800
H	-0.72160700	1.48309400	0.00000400
C	0.96486700	0.16430500	0.00000900
C	1.86419700	1.24689700	-0.00001900
C	1.52462900	-1.13367200	0.00003700
C	3.23971000	1.06234000	-0.00002100
H	1.47129600	2.25841300	-0.00004100
C	2.89049100	-1.32690200	0.00003600
H	0.87966100	-2.00374400	0.00006300
C	3.77585000	-0.23105100	0.00000600
H	3.88372800	1.93097800	-0.00004500
H	3.28777900	-2.33613300	0.00005900
O	-6.94780600	0.34937100	-0.00001600
S	5.50244000	-0.61144600	0.00000800
C	6.30140700	1.02091500	-0.00004100
H	7.37334400	0.82114700	-0.00004400
H	6.04536900	1.58722400	-0.89652100

H	6.04538300	1.58727100	0.89641400

trans-4-hydroxystilbene radical X = CH_3

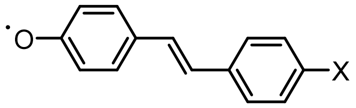

DFT Enthalpy = -654.529823 DFT Free Energy = -654.590455

CBS-QB3 Enthalpy = -653.399934 CBS-QB3 Free Energy = -653.460796

0 2

C	1.99595000	-0.23808800	-0.00003200
C	2.58913500	1.06578100	0.00011200
C	3.93978000	1.23189200	0.00020300
C	4.85604700	0.09313300	0.00015500
C	4.23057200	-1.22476200	0.00000800
C	2.87448900	-1.36574800	-0.00007700
H	1.94825100	1.93980800	0.00015800
H	4.39273600	2.21665300	0.00031900
H	4.89535500	-2.08069800	-0.00002800
H	2.43256500	-2.35759500	-0.00018500
C	0.58408900	-0.46616700	-0.00012700
H	0.29198600	-1.51258300	-0.00018500
C	-0.40044500	0.47932100	-0.00015100
H	-0.11238000	1.52643800	-0.00011500
C	-1.83336700	0.24911500	-0.00018400
C	-2.70058800	1.35709300	-0.00031400
C	-2.42349100	-1.03267600	-0.00026100
C	-4.08037900	1.19744000	-0.00043100
H	-2.27842100	2.35672600	-0.00047900
C	-3.79886600	-1.18430500	-0.00037800
H	-1.79926700	-1.91813800	-0.00039500
C	-4.65913000	-0.07438300	-0.00033700
H	-4.71884000	2.07463800	-0.00068200
H	-4.22237500	-2.18379200	-0.00059500
O	6.08742500	0.24056700	0.00023500
C	-6.15488500	-0.26081200	0.00101300
H	-6.67664900	0.69762500	-0.01338500
H	-6.48226000	-0.83547400	-0.87107800
H	-6.48356700	-0.80915100	0.88956900

EmpiricalDispersion = GD3

DFT Enthalpy = -654.551276 DFT Free Energy = -654.610482

0 2

C	1.99465100	-0.23906700	-0.00030700
C	2.58734900	1.06547300	0.00102400
C	3.93774300	1.23214400	0.00234100
C	4.85441300	0.09338400	0.00245600

C	4.22946300	-1.22504300	0.00107700
C	2.87352000	-1.36687600	-0.00020900
H	1.94632800	1.93930600	0.00100700
H	4.39201600	2.21615400	0.00337200
H	4.89611800	-2.07935200	0.00113000
H	2.43319400	-2.35948500	-0.00122300
C	0.58318700	-0.46628900	-0.00172000
H	0.28881200	-1.51204800	-0.00238700
C	-0.40007000	0.48043000	-0.00222400
H	-0.11026000	1.52705700	-0.00153900
C	-1.83250500	0.25031500	-0.00294900
C	-2.70070700	1.35833100	-0.00419600
C	-2.42134900	-1.03187200	-0.00538900
C	-4.07992200	1.19760700	-0.00652000
H	-2.28001600	2.35863400	-0.00588000
C	-3.79693800	-1.18449400	-0.00776300
H	-1.79652100	-1.91678200	-0.00832500
C	-4.65702600	-0.07536900	-0.00655700
H	-4.71947400	2.07403100	-0.00969600
H	-4.22107100	-2.18357400	-0.01232200
O	6.08557800	0.24117800	0.00366000
C	-6.15212600	-0.26259600	0.01722700
H	-6.46454900	-1.04933600	-0.67477100
H	-6.49108000	-0.55670800	1.01661000
H	-6.67621700	0.65621100	-0.25300100

trans-4-hydroxystilbene radical X = OMe

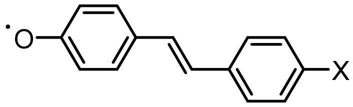

DFT Enthalpy = -729.750681 DFT Free Energy = -729.810621

CBS-QB3 Enthalpy = -728.529363 CBS-QB3 Free Energy = -728.589549

0 2

C	-2.42202200	-0.23971900	-0.00000700
C	-3.06802600	1.03906100	-0.00000100
C	-4.42420300	1.15075900	0.00000800
C	-5.29481500	-0.02330000	0.00000900
C	-4.61630000	-1.31467400	-0.00000400
C	-3.25580800	-1.40126800	-0.00001100
H	-2.46311100	1.93845900	-0.00000500
H	-4.91596300	2.11678300	0.00001200
H	-5.24601200	-2.19679300	-0.00000800
H	-2.77479900	-2.37488900	-0.00002100
C	-1.00353000	-0.41090600	-0.00000800
H	-0.67022900	-1.44516200	-0.00001900
C	-0.05602700	0.57349400	0.00000200
H	-0.38757600	1.60772300	0.00001400
C	1.38259100	0.40461300	-0.00000300

C	2.20733000	1.55203100	-0.00000100
C	2.02812500	-0.84604200	-0.00001000
C	3.58466500	1.45895500	-0.00000500
H	1.74353800	2.53281300	0.00000600
C	3.41143900	-0.95497300	-0.00001600
H	1.44307000	-1.75773000	-0.00001400
C	4.20454500	0.20109700	-0.00001200
H	4.21119500	2.34233600	-0.00000300
H	3.86358900	-1.93748500	-0.00002500
O	-6.53116900	0.07475700	0.00001800
C	6.25529400	-1.03413200	0.00004500
H	6.02320400	-1.62123600	0.89482000
H	6.02329000	-1.62128700	-0.89471900
H	7.31350800	-0.78021700	0.00008700
O	5.56076100	0.20858000	-0.00002200

EmpiricalDispersion = GD3

DFT Enthalpy = -729.774000 DFT Free Energy = -729.833830

0 2

C	-2.45363800	-0.23798000	-0.00000300
C	-3.01277400	1.08142700	-0.00001100
C	-4.35833800	1.28351200	-0.00000900
C	-5.30528000	0.16985700	0.00000300
C	-4.71453900	-1.16414500	0.00001000
C	-3.36288800	-1.34173800	0.00000700
H	-2.34931600	1.93847900	-0.00002100
H	-4.78628500	2.27931400	-0.00001600
H	-5.40318600	-2.00088800	0.00001900
H	-2.94918500	-2.34582900	0.00001200
C	-1.04970300	-0.50201600	-0.00000500
H	-0.78301600	-1.55527600	-0.00000600
C	-0.04066100	0.41892300	-0.00000200
H	-0.30429000	1.47253600	0.00000300
C	1.38237600	0.15364900	-0.00000200
C	2.28049100	1.23566100	0.00000600
C	1.94146300	-1.14604300	-0.00000900
C	3.65859700	1.05522000	0.00000600
H	1.88645100	2.24659700	0.00001100
C	3.30585100	-1.33985800	-0.00000800
H	1.29503000	-2.01498700	-0.00001500
C	4.18319400	-0.24156800	-0.00000100
H	4.30691000	1.92055500	0.00001100
H	3.73444700	-2.33448700	-0.00001400
O	-6.53224600	0.35027800	0.00000600
C	6.45256000	0.51602000	0.00000700
H	7.43082300	0.03906300	0.00000600
H	6.35276700	1.14020000	-0.89486700
H	6.35276400	1.14018800	0.89489100
O	5.50422500	-0.54665300	-0.00000100

trans-4-hydroxystilbene radical X = OH

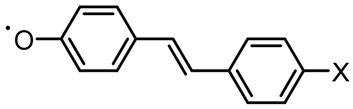

DFT Enthalpy = -690.469875 DFT Free Energy = -690.526330

CBS-QB3 Enthalpy = -689.319913 CBS-QB3 Free Energy = -689.376591

0 2

C	-1.97373100	-0.23949300	0.00000000
C	-2.56521300	1.06531400	0.00000000
C	-3.91552500	1.23387500	0.00000000
C	-4.83448600	0.09727300	0.00000000
C	-4.21094600	-1.22152200	0.00000000
C	-2.85524800	-1.36511100	0.00000000
H	-1.92308000	1.93852100	0.00000100
H	-4.36634800	2.21964800	-0.00000100
H	-4.87716300	-2.07638500	0.00000000
H	-2.41553900	-2.35804700	0.00000000
C	-0.56333200	-0.47012000	0.00000000
H	-0.27401100	-1.51752900	-0.00000100
C	0.42425200	0.47357800	0.00000100
H	0.13670200	1.52082400	0.00000100
C	1.85483800	0.24411500	0.00000100
C	2.72623100	1.35438600	0.00000000
C	2.44538000	-1.03598800	0.00000100
C	4.10192300	1.20723300	0.00000000
H	2.30307700	2.35328500	0.00000000
C	3.82077400	-1.19495800	0.00000000
H	1.82281800	-1.92223300	0.00000100
C	4.66070500	-0.07402600	0.00000000
H	4.76180500	2.06588900	0.00000000
H	4.25120000	-2.19218900	0.00000000
O	-6.06560400	0.24711000	-0.00000100
O	6.01693900	-0.16981500	-0.00000100
H	6.27612900	-1.09747900	0.00000000

EmpiricalDispersion = GD3

DFT Enthalpy = -690.490169 DFT Free Energy = -690.546660

0 2

C	-1.97318900	-0.23959400	-0.00000200
C	-2.56179800	1.06691400	0.00000100
C	-3.91155700	1.23856600	0.00000100
C	-4.83296100	0.10364400	0.00000100
C	-4.21237000	-1.21676500	-0.00000200
C	-2.85703800	-1.36374100	-0.00000400
H	-1.91792000	1.93876200	0.00000200
H	-4.36196700	2.22439300	0.00000300

H	-4.88196300	-2.06881100	-0.00000400
H	-2.42073800	-2.35819700	-0.00000700
C	-0.56346200	-0.47193200	-0.00000300
H	-0.27325600	-1.51895800	-0.00000700
C	0.42418300	0.47153900	0.00000200
H	0.13646300	1.51879300	0.00000500
C	1.85360600	0.23911200	0.00000200
C	2.72590400	1.34487600	-0.00000200
C	2.44182100	-1.04584800	0.00000500
C	4.10401900	1.18845800	-0.00000300
H	2.30936400	2.34651000	-0.00000300
C	3.81268900	-1.21302800	0.00000400
H	1.81442400	-1.92857400	0.00000900
C	4.65741200	-0.09484200	0.00000000
H	4.75206800	2.05966900	-0.00000500
H	4.26014800	-2.19922600	0.00000600
O	-6.06361400	0.25628000	0.00000300
O	5.99696400	-0.32733000	-0.00000100
H	6.47301600	0.50989900	-0.00000500

trans-4-hydroxystilbene radical X = 3,4-dioxyl

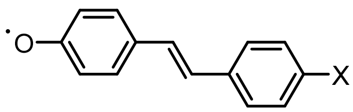

DFT Enthalpy = -803.790149 DFT Free Energy = -803.849803

CBS-QB3 Enthalpy = -802.485881 CBS-QB3 Free Energy = -802.545776

0 2

C	-2.54782700	-0.20004400	-0.00218600
C	-3.24885300	1.04939600	0.00655600
C	-4.60853400	1.10166200	0.01114800
C	-5.42677200	-0.10964700	0.00733600
C	-4.69252600	-1.37028500	-0.00178700
C	-3.32964000	-1.39724200	-0.00616900
H	-2.68377900	1.97433100	0.00964300
H	-5.14226100	2.04510400	0.01783700
H	-5.28306800	-2.27905900	-0.00485200
H	-2.80633600	-2.34873100	-0.01294100
C	-1.12332800	-0.30882700	-0.00683800
H	-0.74523300	-1.32740100	-0.01277300
C	-0.22011000	0.71647600	-0.00396600
H	-0.59437100	1.73548200	0.00293500
C	1.22388000	0.60750400	-0.00904400
C	1.98326900	1.79426500	-0.00007900
C	1.90171600	-0.64307000	-0.02146300
C	3.38194900	1.79508700	-0.00137100
H	1.46113000	2.74393600	0.01042800
C	3.27094700	-0.62430200	-0.02053200
H	1.37050200	-1.58464600	-0.03437300

C	4.00412900	0.56478200	-0.01010400
H	3.95280500	2.71419600	0.00270100
O	-6.66605000	-0.06570000	0.01162500
O	5.33957400	0.28498100	-0.03267200
O	4.13262100	-1.68996100	-0.05382800
C	5.44690300	-1.14165100	0.09571200
H	5.83601500	-1.38575600	1.09027300
H	6.09422600	-1.52665000	-0.69315000

EmpiricalDispersion = GD3

DFT Enthalpy = -803.812608 DFT Free Energy = -803.872208

0 2

C	-2.54591200	-0.20064600	-0.00241000
C	-3.24675900	1.04935000	0.00683600
C	-4.60625200	1.10190200	0.01193500
C	-5.42462200	-0.10961900	0.00816900
C	-4.69059800	-1.37067400	-0.00150800
C	-3.32777300	-1.39819100	-0.00638900
H	-2.68173100	1.97422400	0.00988600
H	-5.14143400	2.04436600	0.01900700
H	-5.28295500	-2.27810600	-0.00455400
H	-2.80579100	-2.35045700	-0.01356800
C	-1.12183400	-0.30832800	-0.00753500
H	-0.74145000	-1.32601300	-0.01382300
C	-0.22031000	0.71837700	-0.00457000
H	-0.59675600	1.73656800	0.00295900
C	1.22318400	0.60986000	-0.01020000
C	1.98388800	1.79609900	0.00019400
C	1.89983500	-0.64153100	-0.02433600
C	3.38268100	1.79496600	-0.00107100
H	1.46418200	2.74712000	0.01215900
C	3.26870400	-0.62441000	-0.02328600
H	1.36967800	-1.58341000	-0.03894900
C	4.00350500	0.56411000	-0.01122200
H	3.95624300	2.71219300	0.00395100
O	-6.66373000	-0.06556500	0.01293600
O	5.33859100	0.28331200	-0.03548900
O	4.12845700	-1.69131700	-0.05977600
C	5.44195000	-1.14339000	0.10582600
H	5.81478900	-1.37976700	1.10915900
H	6.10055700	-1.53540800	-0.67019800

trans-4-hydroxystilbene radical X = NH_2

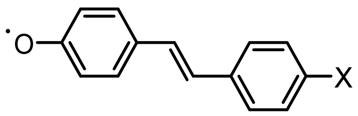

DFT Enthalpy = -670.589196 DFT Free Energy = -670.646038

CBS-QB3 Enthalpy = -669.450087 CBS-QB3 Free Energy = -669.507160

0 2

C	-1.98234400	0.24007900	-0.00134100
C	-2.57439700	-1.06486100	0.00129500
C	-3.92454200	-1.23427000	0.00334500
C	-4.84539600	-0.09917800	0.00301900
C	-4.22178200	1.21954700	-0.00003900
C	-2.86640300	1.36434700	-0.00207000
H	-1.93187800	-1.93793500	0.00162200
H	-4.37425400	-2.22065500	0.00531900
H	-4.88833200	2.07425600	-0.00060400
H	-2.42779300	2.35790200	-0.00435800
C	-0.57406900	0.47139200	-0.00299500
H	-0.28483900	1.51887800	-0.00503800
C	0.41655900	-0.47225100	-0.00241000
H	0.12832700	-1.51954600	-0.00145500
C	1.84286900	-0.24333500	-0.00351100
C	2.71854400	-1.34983000	-0.00432600
C	2.43892100	1.03749100	-0.00271700
C	4.09273800	-1.19974800	-0.00379700
H	2.29837900	-2.35030000	-0.00407500
C	3.80955400	1.19870000	-0.00252100
H	1.81584800	1.92374200	-0.00104700
C	4.67037600	0.08233600	-0.00123200
H	4.73379400	-2.07525100	-0.00824400
H	4.23507000	2.19709500	-0.00637300
O	-6.07654500	-0.25026500	0.00524000
N	6.04078300	0.24684600	-0.05176800
H	6.60985600	-0.53714900	0.22664300
H	6.40893300	1.14063800	0.23385800

EmpiricalDispersion = GD3

DFT Enthalpy = -670.609992 DFT Free Energy = -670.666864

0 2

C	-1.98114500	0.24088000	-0.00140300
C	-2.57291700	-1.06462000	0.00146500
C	-3.92284800	-1.23436200	0.00357200
C	-4.84389800	-0.09911600	0.00307900
C	-4.22059300	1.22002700	-0.00021000
C	-2.86532400	1.36544800	-0.00229700
H	-1.93038700	-1.93759200	0.00193600
H	-4.37406000	-2.21991700	0.00572600
H	-4.88887800	2.07321000	-0.00089600
H	-2.42814700	2.35968600	-0.00475800
C	-0.57319000	0.47102800	-0.00311800
H	-0.28141100	1.51778000	-0.00531400
C	0.41594700	-0.47406000	-0.00244500
H	0.12593500	-1.52085100	-0.00142600
C	1.84174900	-0.24533600	-0.00355400

C	2.71839700	-1.35122200	-0.00456500
C	2.43654500	1.03627200	-0.00257300
C	4.09240100	-1.19958200	-0.00403700
H	2.30022400	-2.35256500	-0.00446600
C	3.80684400	1.19902900	-0.00238900
H	1.81259000	1.92177800	-0.00072100
C	4.66891600	0.08327400	-0.00122700
H	4.73442600	-2.07436200	-0.00860200
H	4.23131600	2.19786000	-0.00607100
O	-6.07487300	-0.25034800	0.00535200
N	6.03932800	0.24903500	-0.05152900
H	6.40725400	1.14287700	0.23425600
H	6.60951800	-0.53431900	0.22642200

<u>Addition radical product</u>

α,α-Dimethyl quinone methide

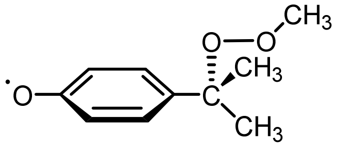

DFT Enthalpy = -614.363462 DFT Free Energy = -614.419823

CBS-QB3 Enthalpy = -613.393953 CBS-QB3 Free Energy = -613.450525

0 2

C	-1.34616900	1.22990600	-0.36008700
C	-2.68638400	0.94335000	-0.44678100
C	-3.19771200	-0.37439000	-0.12116600
C	-2.21737000	-1.35594300	0.30193800
C	-0.88380900	-1.04388800	0.38653600
H	-1.00159900	2.22422000	-0.61536000
H	-3.40860100	1.68658300	-0.76381100
H	-2.58723600	-2.34410400	0.54980100
H	-0.16752200	-1.79060400	0.70186000
C	-0.41391800	0.25053000	0.05379800
O	-4.41601600	-0.64603100	-0.19993000
O	1.72664600	-0.65993800	-0.08386700
O	3.15161200	-0.50978100	0.20651200
C	3.81681300	-1.25509100	-0.79762400
H	4.87490100	-1.17978000	-0.53654000
H	3.50865300	-2.30496800	-0.77946000
H	3.64437200	-0.83323400	-1.79284400
C	1.06525300	0.59458500	0.21286500
C	1.55393600	1.66561400	-0.76974200
H	1.27856500	1.40681900	-1.79401100
H	1.14466300	2.64907600	-0.53130900
H	2.63990500	1.73131900	-0.70108700

C	1.33731600	1.00566300	1.67240500
H	2.39867400	1.21380100	1.81161500
H	0.76452800	1.90106100	1.92404500
H	1.04501900	0.20379100	2.35252900

α-Methyl quinone methide

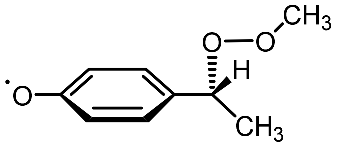

DFT Enthalpy = -575.068013 DFT Free Energy = -575.121728

CBS-QB3 Enthalpy = -574.163739 CBS-QB3 Free Energy = -574.217637

0 2

C	-1.15142900	0.99220800	-0.92428200
C	-2.49118600	0.69348800	-0.94361000
C	-3.04252900	-0.32981000	-0.07354600
C	-2.10586200	-1.00705300	0.80492000
C	-0.77192500	-0.68414300	0.81038100
H	-0.75528900	1.75349700	-1.58902000
H	-3.18224500	1.19878100	-1.60797100
H	-2.51049800	-1.77501100	1.45372800
H	-0.08334700	-1.20149000	1.46699400
C	-0.26924000	0.31847600	-0.05107900
O	-4.26036600	-0.61319000	-0.08320000
O	1.91669100	-0.51244400	0.17261400
O	3.33350000	-0.18541100	0.05229500
C	3.87341200	-1.14680800	-0.83944500
H	4.93798400	-0.90635400	-0.88708400
H	3.74269500	-2.16158200	-0.45195400
H	3.42741900	-1.06695900	-1.83604700
C	1.18937900	0.71013700	-0.01481100
H	1.47455000	1.13421300	-0.98495000
C	1.49145100	1.72109300	1.09717100
H	2.55714500	1.95195800	1.10660500
H	0.93158100	2.64510700	0.93270300
H	1.20897400	1.31068400	2.06912700

Quinone methide

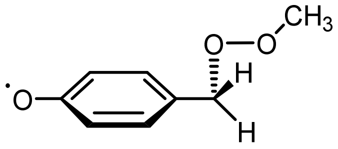

DFT Enthalpy = -535.768538 DFT Free Energy = -535.819811

CBS-QB3 Enthalpy = -534.931834 CBS-QB3 Free Energy = -534.983263

0 2

C	1.10585400	1.41003300	0.06878600
C	2.43336500	1.10988900	0.25334300
C	2.91610900	-0.25428400	0.13463400
C	1.92634900	-1.26746200	-0.18728100
C	0.60777200	-0.93996000	-0.37380100
H	0.76057800	2.43370000	0.17225200
H	3.16438900	1.87027500	0.50142300
H	2.28060000	-2.28781400	-0.27519600
H	-0.12389000	-1.70378900	-0.60771700
C	0.17309100	0.40096600	-0.25266200
O	4.12132600	-0.54050400	0.30270000
O	-2.07702400	-0.22208800	0.13107400
O	-3.42973300	0.00992900	-0.37705200
C	-4.29712900	-0.42090400	0.65534400
H	-5.29962900	-0.26715800	0.25020600
H	-4.15023900	-1.48236900	0.87917800
H	-4.16442000	0.17501300	1.56380200
C	-1.26648300	0.75827400	-0.50401100
H	-1.47732800	0.74608800	-1.58255200
H	-1.50017900	1.75805000	-0.12128200

α-Phenyl quinone methide

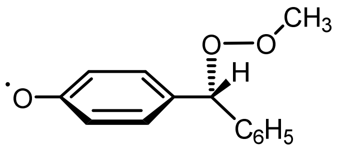

DFT Enthalpy = -766.789766 DFT Free Energy = -766.852318

CBS-QB3 Enthalpy = -765.552346 CBS-QB3 Free Energy = -765.615145

0 2

C	1.07835800	-0.43150500	-0.84051400
C	2.29970000	-0.88904900	-1.26667300
C	3.42004000	-1.01422900	-0.35069300
C	3.17811700	-0.62634000	1.02781200
C	1.94778500	-0.16402300	1.42458300
H	0.25132300	-0.34683900	-1.53408000
H	2.47570000	-1.17539700	-2.29701000
H	4.00774000	-0.72007200	1.71864400
H	1.78498400	0.12837400	2.45724200
C	0.87635500	-0.05899700	0.50913300
O	4.53543200	-1.43408100	-0.72776700
O	-0.46857800	1.94493500	0.83911500
O	-0.33215900	2.27973100	-0.57950400
C	0.68994200	3.26176900	-0.64897400
H	0.72114300	3.54475600	-1.70365100

H	0.43619900	4.13524100	-0.04128900
H	1.65844200	2.85659900	-0.34162700
C	-0.44751300	0.51612000	0.98031000
H	-0.48088400	0.43678500	2.07392100
C	-1.69003600	-0.16765700	0.42349700
C	-2.76269600	0.56919800	-0.08130800
C	-1.79319500	-1.56184200	0.48556400
C	-3.91298800	-0.07974800	-0.52680600
H	-2.68930900	1.64672300	-0.13352700
C	-2.94482800	-2.20792600	0.04562900
H	-0.96947800	-2.14828500	0.87931100
C	-4.00927700	-1.46728800	-0.46482600
H	-4.73630900	0.50416900	-0.92304400
H	-3.00986200	-3.28877800	0.10114400
H	-4.90583000	-1.96884500	-0.81119400

α,α-Dicyano quinone methide

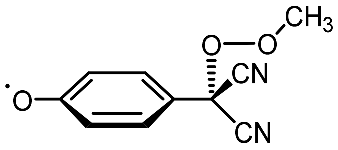

DFT Enthalpy = -720.261848 DFT Free Energy = -720.320365

CBS-QB3 Enthalpy = -719.149734 CBS-QB3 Free Energy = -719.208468

0 2

C	-1.28672900	1.21893700	0.30622500
C	-2.64354800	1.23407000	0.08163700
C	-3.35317000	0.03245600	-0.31658600
C	-2.56424700	-1.17875800	-0.46061500
C	-1.21375200	-1.17431200	-0.22766200
H	-0.77386000	2.12650600	0.60020500
H	-3.22781800	2.13956000	0.19084800
H	-3.08945700	-2.07872800	-0.75624500
H	-0.63366600	-2.08265600	-0.33708300
C	-0.56422000	0.02131200	0.15631900
O	-4.58482300	0.04059200	-0.52525000
O	1.46756500	-0.39120400	-0.92212100
O	2.91738800	-0.54941500	-0.77659500
C	3.50998900	0.21282400	-1.82376000
H	4.57862300	0.02343000	-1.70322900
H	3.17854400	-0.14474100	-2.80180100
H	3.30381300	1.27960700	-1.70842900
C	0.95409700	-0.03586500	0.35978600
C	1.52347900	1.25869100	0.79812500
C	1.29244900	-1.06830900	1.37082900
N	1.96099400	2.27875900	1.10384100
N	1.51567700	-1.87719300	2.15925600

α,α-Diphenyl quinone methide

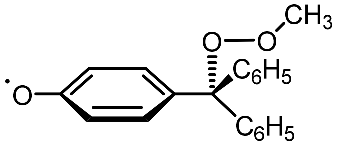

DFT Enthalpy = -997.797934 DFT Free Energy = -997.868935

CBS-QB3 Enthalpy = NA CBS-QB3 Free Energy = NA

0 1

C	-1.95826300	-1.18546000	0.35132900
C	-3.30725700	-1.18699400	0.36645100
C	-4.09557000	0.00000200	-0.00000400
C	-3.30725200	1.18699600	-0.36645400
C	-1.95825800	1.18545900	-0.35131800
H	-1.41850400	-2.07070500	0.66270700
H	-3.87058700	-2.06152100	0.67201200
H	-3.87057500	2.06152400	-0.67202400
H	-1.41849200	2.07070200	-0.66269200
C	-1.18691500	0.00000000	0.00001200
C	0.19450700	0.00000100	0.00002000
O	-5.32297700	0.00000300	-0.00000900
C	0.98400300	-1.25609300	0.03640900
C	2.07370500	-1.38646100	0.91415700
C	0.69344500	-2.32501800	-0.82774700
C	2.82154200	-2.55744100	0.95134700
H	2.32073100	-0.56746000	1.57925400
C	1.45491300	-3.48962600	-0.80146500
H	-0.11965800	-2.22750000	-1.53704400
C	2.51606600	-3.61294200	0.09263200
H	3.64676100	-2.64599100	1.64881500
H	1.22165700	-4.29865900	-1.48434100
H	3.10643200	-4.52182700	0.11538000
C	0.98400200	1.25609500	-0.03639100
C	0.69345600	2.32503000	0.82775600
C	2.07369500	1.38645000	-0.91415100
C	1.45492700	3.48963500	0.80145400
H	-0.11964000	2.22752100	1.53706300
C	2.82153600	2.55742700	-0.95136200
H	2.32071300	0.56744000	-1.57924100
C	2.51607100	3.61293800	-0.09265500
H	1.22168100	4.29867700	1.48432400
H	3.64674800	2.64596800	-1.64883900
H	3.10644000	4.52182200	-0.11541700

2,6-di-tert-butyl-α-methyl quinone methide

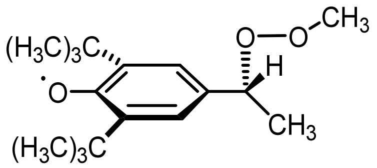

DFT Enthalpy =	-889.428856	DFT Free Energy =	-889.505845
CBS-QB3 Enthalpy =	-887.983036	CBS-QB3 Free Energy =	-888.060419

0 2

C	-0.02919500	-1.46399600	0.15558700
C	1.29263300	-1.11097100	-0.01181600
C	1.62620400	0.32718400	-0.06534800
C	0.54252800	1.32304700	0.05855200
C	-0.74623800	0.86753500	0.23256300
H	-0.31173800	-2.50844100	0.18988100
H	-1.56690500	1.56428300	0.32191600
C	-1.05099900	-0.50609700	0.28296200
O	2.81121100	0.69482700	-0.21560200
O	-3.30272000	-0.08960100	-0.25249000
O	-4.66418700	-0.60831100	-0.16655500
C	-5.13739400	-0.66304400	-1.50181400
H	-6.16632200	-1.01931500	-1.41252700
H	-5.13073400	0.32867000	-1.96408100
H	-4.55197800	-1.36149600	-2.10862300
C	-2.47084900	-0.96678600	0.52275400
H	-2.59162500	-1.98473700	0.13384500
C	2.40664700	-2.15777700	-0.14944800
C	1.85696300	-3.59563800	-0.07230400
H	1.36957700	-3.80023400	0.88529500
H	1.14622400	-3.80928700	-0.87578800
H	2.68616100	-4.30005600	-0.17388800
C	3.11574100	-1.99749200	-1.51801400
H	3.58464400	-1.01963100	-1.60594200
H	3.88695700	-2.76671200	-1.62297200
H	2.40466200	-2.12142600	-2.34034000
C	3.43582900	-1.98497200	0.99629500
H	4.20978600	-2.75399300	0.91208900
H	3.91025200	-1.00672000	0.95448100
H	2.95367300	-2.10028400	1.97174400
C	0.87390600	2.82041200	-0.00682000
C	1.52554200	3.15640300	-1.37223000
H	1.72799000	4.23044800	-1.42644200
H	2.46202900	2.61844200	-1.50502600
H	0.85282700	2.90037000	-2.19626000
C	1.84317500	3.19749400	1.14199000
H	2.78476400	2.65899500	1.05600200
H	2.04951500	4.27164800	1.10872700
H	1.39702400	2.97241600	2.11541900
C	-0.38799800	3.69335400	0.13773300
H	-1.11174700	3.50727500	-0.66055400

H	-0.88739100	3.54035000	1.09855000
H	-0.10082900	4.74632600	0.08206600
C	-2.85121800	-0.93504700	2.00698000
H	-2.73276100	0.07422100	2.40728600
H	-3.88952900	-1.24497200	2.13017000
H	-2.21060600	-1.61311700	2.57639900

Truncated dimer

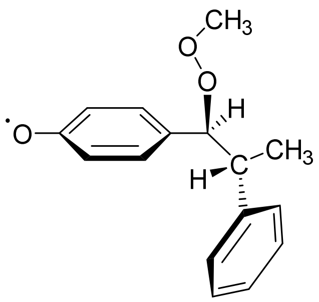

DFT Enthalpy = -845.379443 DFT Free Energy = -845.448249

CBS-QB3 Enthalpy = -844.008115 CBS-QB3 Free Energy = -844.077218

0 2

C	1.68586100	-0.73545200	-0.94678200
C	2.03864400	-2.05690200	-1.04386900
C	1.65692100	-3.01393600	-0.01965400
C	0.89517400	-2.49267000	1.10074700
C	0.55651100	-1.16384100	1.17166500
H	1.98969400	-0.02773600	-1.70968300
H	2.61367700	-2.43783300	-1.87997300
H	0.61296200	-3.19839200	1.87314800
H	-0.01169700	-0.79243000	2.01765600
C	0.93550600	-0.26276200	0.15473600
O	1.97130500	-4.22160000	-0.09771200
O	1.58857600	2.04790200	-0.20443700
O	2.61138400	1.96629700	0.84596800
C	3.78537300	2.50236300	0.26894200
H	4.52260200	2.46789200	1.07383500
H	4.12979300	1.89716600	-0.57569200
H	3.63454500	3.53954000	-0.04808300
C	0.53023100	1.19587100	0.22243000
C	-0.67504300	1.54242800	-0.70021500
H	0.27533000	1.45818500	1.25591200
C	-1.89282900	0.70535500	-0.33377400
C	-2.36613100	-0.27753900	-1.20731200
C	-2.57489100	0.90298300	0.87332900
C	-3.48544600	-1.04383900	-0.88862100
H	-1.85000100	-0.44796500	-2.14632700
C	-3.69261200	0.13833500	1.19677100
H	-2.23685900	1.66374000	1.56895000

C	-4.15235100	-0.83895000	0.31597800
H	-3.83382500	-1.80139600	-1.58165800
H	-4.20687700	0.30800700	2.13631100
H	-5.02313000	-1.43390300	0.56681800
C	-0.98133300	3.05080000	-0.68657800
H	-1.22038800	3.40135600	0.32101200
H	-0.12719900	3.62451100	-1.04555200
H	-1.84128200	3.26025700	-1.32687200
H	-0.37898200	1.26472500	-1.71711200

Cyclized intermediate

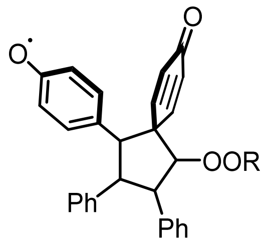

DFT Enthalpy = -1420.714716 DFT Free Energy = -1420.806833

0 2

C	-2.74321300	0.50606000	1.01443000
C	-3.30831100	0.55096600	2.29627500
C	-4.68583600	0.48520500	2.48810700
C	-5.53443100	0.37453600	1.39061600
C	-4.99045300	0.32927200	0.10920600
C	-3.61212200	0.39254000	-0.07947000
H	-2.65787700	0.64004500	3.16121300
H	-5.09251000	0.52239400	3.49252400
H	-6.60814400	0.32327400	1.53167700
H	-5.64238200	0.23986400	-0.75280600
H	-3.21285900	0.35538200	-1.08115800
C	-1.23211000	0.57207000	0.90094200
H	-0.89756700	0.83017600	1.90778400
C	-0.42299100	-0.74079400	0.53949000
H	-0.10945300	-1.17361000	1.49085100
C	-1.15941000	-1.84985600	-0.19442900
C	-1.51014800	-3.01205400	0.49984700
C	-1.49710200	-1.76769300	-1.55144400
C	-2.17590800	-4.05959000	-0.13257600
H	-1.25453700	-3.10100800	1.55077400
C	-2.16032000	-2.81290500	-2.18841500
H	-1.25436700	-0.87536900	-2.11710400
C	-2.50239000	-3.96422100	-1.48201400
H	-2.43280900	-4.95088400	0.42882100
H	-2.41049900	-2.72720600	-3.24016400
H	-3.01585500	-4.77886400	-1.97989300
C	0.83582800	-0.23113800	-0.23407800
H	0.60115900	-0.29933200	-1.29668000

C	2.10869400	-1.01216800	-0.01864400
C	2.55533900	-1.40308900	1.26792400
C	2.89787200	-1.36668900	-1.13859200
C	3.71942400	-2.10808100	1.43721800
H	1.96706700	-1.14342000	2.14015900
C	4.06808500	-2.07082800	-1.00214600
H	2.55838700	-1.07439900	-2.12670800
C	4.54451900	-2.48484800	0.30386700
H	4.06670800	-2.41055800	2.41818000
H	4.67296500	-2.34724500	-1.85768300
C	0.93216400	1.33601600	0.02773700
C	1.65705500	2.00407700	-1.10419300
C	1.54045500	1.67359700	1.36206500
C	2.72458000	2.79659300	-0.95334400
H	1.26149100	1.81349000	-2.09526800
C	2.59431400	2.48209000	1.51889200
H	1.09054400	1.23081300	2.24493600
C	3.28150900	3.11723600	0.37329800
H	3.22030100	3.25252600	-1.80313800
H	3.00154400	2.70461700	2.49915800
O	5.60357000	-3.13314000	0.44660400
O	4.23964100	3.85911000	0.52495900
C	-0.60852000	1.69124200	0.03929600
H	-0.79060700	2.68614100	0.45078800
O	-1.17086900	1.62737400	-1.26665200
O	-0.93576500	2.90614800	-1.93062600
C	-2.21520100	3.46155500	-2.20538000
H	-2.77573900	3.65474000	-1.28603700
H	-1.99247900	4.40386800	-2.71083100
H	-2.79332400	2.81402300	-2.87059700

Addition transition states

QMD meso

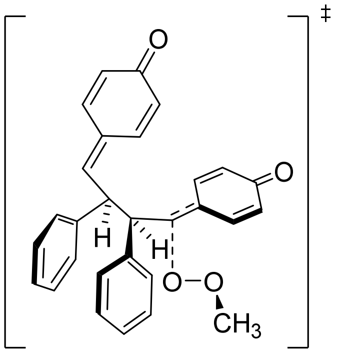

TS frequency: -336.38 cm^{-1}

DFT Enthalpy = -1420.684490 DFT Free Energy = -1420.781737

0 2

C	-0.38120600	-1.79043800	-0.95719400
C	-0.52137000	-3.16660000	-0.74843600
C	-0.65608200	-4.04459400	-1.82186800
C	-0.65292100	-3.55902800	-3.12680100
C	-0.51822300	-2.19002300	-3.34919200
C	-0.38606800	-1.31430200	-2.27447400
H	-0.51969600	-3.55507600	0.26423000
H	-0.76552700	-5.10712400	-1.63614400
H	-0.76185400	-4.23956800	-3.96344700
H	-0.52420200	-1.80064300	-4.36106500
H	-0.29705800	-0.25113200	-2.46698500
C	-0.21950100	-0.85522200	0.23816700
H	-0.56341700	-1.39733500	1.12419300
C	1.28443300	-0.46809600	0.55765400
H	1.20311200	0.23784700	1.38064300
C	2.07382800	-1.66343100	1.08893100
C	2.21401700	-1.80975400	2.47405100
C	2.65538600	-2.62794300	0.25821300
C	2.90925600	-2.88737200	3.01661200
H	1.77341500	-1.06870800	3.13335700
C	3.35448700	-3.70538500	0.79856100
H	2.56329900	-2.55120500	-0.81803800
C	3.48421800	-3.83985900	2.17853700
H	3.00716300	-2.97783400	4.09267400
H	3.79913200	-4.44046500	0.13700900
H	4.03170100	-4.67683300	2.59678200
C	1.98124600	0.19808300	-0.59229800
H	2.24049000	-0.44308900	-1.42947500
C	-1.07192000	0.38993600	0.10919900
H	-0.56209500	1.28544700	-0.22922000
C	2.33766900	1.50551100	-0.70129800
C	2.06212800	2.52072700	0.31158700

C	3.04184700	1.95043700	-1.90153100
C	2.43473200	3.80355100	0.14109600
H	1.53264300	2.23310700	1.21194800
C	3.42210800	3.22842700	-2.08022700
H	3.25579200	1.20161700	-2.65854000
C	3.14571800	4.26420000	-1.06778500
H	2.22768800	4.56082700	0.88868100
H	3.94649200	3.55452900	-2.97109100
C	-2.46428600	0.41100400	0.01626500
C	-3.29617900	-0.72958800	0.31369700
C	-3.11901300	1.64037600	-0.36095500
C	-4.64558100	-0.65652100	0.23303900
H	-2.82265300	-1.66046600	0.59996600
C	-4.46784800	1.73189900	-0.45091800
H	-2.49411500	2.50021900	-0.58082700
C	-5.33330100	0.58272400	-0.15630400
H	-5.27899800	-1.50900200	0.44940100
H	-4.96411100	2.64943800	-0.74513000
O	3.48153000	5.43257200	-1.21862600
O	-6.56075600	0.65315300	-0.22691400
O	-0.84226400	0.95570200	2.11526800
O	-1.12752700	2.26439700	2.34580800
C	-2.31169200	2.37376600	3.15533500
H	-2.15731500	1.85607000	4.10393100
H	-2.44603700	3.44339600	3.31828700
H	-3.16700600	1.95448500	2.62458800

QMD meso

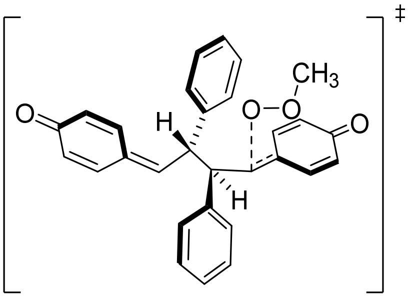

TS frequency: -353.86 cm^{-1}
DFT Enthalpy = -1420.682110 DFT Free Energy = -1420.778952

0 2

C	-0.12248400	-1.58014000	0.65937600
C	0.04835500	-2.35166400	1.81406700
C	0.31831300	-3.71648800	1.73444000
C	0.42651300	-4.33237500	0.49101000
C	0.26239200	-3.57368900	-0.66669600
C	-0.00756700	-2.21040100	-0.58602400
H	-0.03970500	-1.88231100	2.78901400
H	0.44361600	-4.29494800	2.64282100
H	0.63793200	-5.39333000	0.42332300

H	0.34549700	-4.04545300	-1.63937300
H	-0.14872900	-1.63826400	-1.49370000
C	-0.37207300	-0.08258600	0.78594800
H	-0.47836100	0.12570400	1.86076000
C	0.87853800	0.74625900	0.30397400
H	0.92613100	0.63473300	-0.77677400
C	0.74534700	2.24047600	0.60545700
C	0.73173400	3.16560000	-0.44316600
C	0.65073200	2.71950600	1.91841900
C	0.62555100	4.53165500	-0.18916500
H	0.79072500	2.81179100	-1.46643300
C	0.54412700	4.08366600	2.17433600
H	0.66222900	2.02743100	2.75400200
C	0.53258300	4.99542300	1.12040100
H	0.61466700	5.23221400	-1.01658100
H	0.47281700	4.43495000	3.19761500
H	0.45163800	6.05786300	1.31942200
C	2.14227200	0.22921900	0.93502400
H	2.12502000	0.15776700	2.02078900
C	-1.64631600	0.48175500	0.18901500
H	-1.59893400	1.55622000	0.05820900
C	3.30353600	-0.11365300	0.32208800
C	3.50812600	-0.07474200	-1.12090600
C	4.43572000	-0.55062000	1.13294100
C	4.68468300	-0.41991000	-1.67674300
H	2.68814500	0.24197600	-1.75411000
C	5.61618500	-0.90011700	0.59013200
H	4.29318700	-0.58533200	2.20898600
C	5.83699900	-0.86185400	-0.86669600
H	4.84095400	-0.39202000	-2.74918200
H	6.45514700	-1.22419200	1.19511800
C	-2.94605700	-0.03316200	0.23604200
C	-3.29798700	-1.39561800	0.55795700
C	-4.02921000	0.87888000	-0.06268400
C	-4.59170700	-1.79764800	0.59794100
H	-2.51027900	-2.10312500	0.77239500
C	-5.32653100	0.49310900	-0.02836400
H	-3.77000700	1.90381100	-0.30840000
C	-5.70419700	-0.88501800	0.30723100
H	-4.86112000	-2.81755900	0.84740100
H	-6.13697100	1.18160500	-0.23846400
O	6.90575700	-1.17732600	-1.37619600
O	-6.87944000	-1.25250600	0.34015800
O	-1.20383800	0.31668700	-1.86552000
O	-1.80851100	1.30751700	-2.57548400
C	-2.78821900	0.74360300	-3.46491500
H	-3.55850400	0.22509000	-2.89303100
H	-3.21108000	1.59550800	-3.99837000
H	-2.30189200	0.05810800	-4.16175200

QMD meso extended conformation

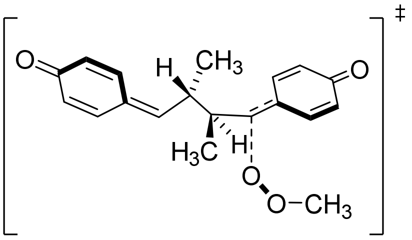

TS frequency: -368.24 cm^{-1}

DFT Enthalpy = -1037.239059 DFT Free Energy = -1037.319825

CBS-QB3 Enthalpy = -1035.559817 CBS-QB3 Free Energy = -1035.640966

0 2

C	-0.07485800	0.55779400	-0.51903000
H	-0.31501600	0.15720100	-1.50910000
C	0.93420500	-0.44773000	0.15962400
H	1.18466700	-0.03152300	1.13994100
C	2.17490900	-0.56466900	-0.67782100
H	1.99348200	-0.87164900	-1.70796000
C	-1.33231900	0.64143600	0.31175200
H	-1.19477300	1.12362300	1.27591900
C	3.47087200	-0.39323200	-0.31207100
C	3.89533600	0.00723100	1.02488600
C	4.52731300	-0.61911600	-1.29486400
C	5.19577300	0.15936400	1.33724000
H	3.13821300	0.18403400	1.77977100
C	5.83045600	-0.46952900	-0.99617000
H	4.22244700	-0.91781900	-2.29356600
C	6.27221200	-0.06653800	0.35157100
H	5.51520100	0.45679600	2.32956800
H	6.61215400	-0.63784300	-1.72788800
C	-2.45442700	-0.18969400	0.20367100
C	-2.75963200	-0.96851000	-0.97142800
C	-3.38895700	-0.24406400	1.29980700
C	-3.86393100	-1.75042500	-1.03513000
H	-2.09157000	-0.91547400	-1.82255500
C	-4.49602400	-1.02641200	1.25869700
H	-3.17447400	0.35610500	2.17813700
C	-4.81270000	-1.84447400	0.08200200
H	-4.09829800	-2.33825400	-1.91512200
H	-5.19044000	-1.08306700	2.08900700
O	7.45428400	0.07361300	0.63903700
O	-5.81573800	-2.55818400	0.03085300
C	0.59808200	1.93065200	-0.69399100
H	1.53222900	1.82784200	-1.24904800
H	-0.04975200	2.62374700	-1.22530200
H	0.83386200	2.37439800	0.27825300
C	0.36683400	-1.86767300	0.37246200
H	-0.50472800	-1.86000500	1.02821800
H	0.06908400	-2.32263100	-0.57652600
H	1.12634900	-2.50812100	0.82571300

O	-2.18118400	2.35646000	-0.45426100
O	-3.13634900	2.86159600	0.37095600
C	-4.41548100	2.82690300	-0.28562700
H	-4.70057400	1.79637200	-0.49982000
H	-5.11047100	3.27889800	0.42267400
H	-4.37165900	3.41160600	-1.20645800

EmpiricalDispersion = GD3

TS frequency: -354.21 cm^{-1}
DFT Enthalpy = -1037.287378 DFT Free Energy = -1037.366983

0 2

C	-0.01975100	0.52921400	-0.42670200
H	-0.27143300	0.19328800	-1.43740400
C	0.92578400	-0.55850000	0.21295900
H	1.19267400	-0.19622000	1.20978100
C	2.15608200	-0.70615200	-0.63093900
H	1.96080800	-1.07022600	-1.63960300
C	-1.27556200	0.64439400	0.39650000
H	-1.13130700	1.12825200	1.35860300
C	3.45042800	-0.47452900	-0.29858400
C	3.87698000	0.02298000	1.00477700
C	4.50019700	-0.72146500	-1.28360700
C	5.17418100	0.24619800	1.28522200
H	3.12330400	0.21793500	1.75833200
C	5.80028700	-0.50306200	-1.01592500
H	4.19413000	-1.09299500	-2.25722800
C	6.24444300	0.00105300	0.29702300
H	5.49734400	0.61782400	2.25081300
H	6.57915500	-0.68536100	-1.74711100
C	-2.41877000	-0.15350800	0.27860100
C	-2.71700500	-0.94398200	-0.89030900
C	-3.39358700	-0.12893800	1.33686000
C	-3.85846700	-1.66672900	-0.98194700
H	-2.01031500	-0.94984700	-1.71081200
C	-4.54304400	-0.84658500	1.26526200
H	-3.18439300	0.48617100	2.20538700
C	-4.85709600	-1.67402700	0.09509000
H	-4.09034000	-2.26578500	-1.85475500
H	-5.27697800	-0.83963400	2.06251600
O	7.42386700	0.20506100	0.55500800
O	-5.89974100	-2.32652500	0.01564700
C	0.72231400	1.87316100	-0.52251000
H	1.64808300	1.75928800	-1.08837700
H	0.10811000	2.62978900	-1.00507100
H	0.98348100	2.23909300	0.47546200
C	0.27825900	-1.94947400	0.36430500
H	-0.58327600	-1.92271800	1.03229300
H	-0.05977300	-2.33400800	-0.60202600
H	1.00387000	-2.65588700	0.77295200

O	-2.07238100	2.37676500	-0.39445000
O	-3.20830600	2.74468400	0.25115200
C	-4.34088200	2.54675300	-0.61513700
H	-4.42819400	1.49228300	-0.87881600
H	-5.20591600	2.87114600	-0.03606400
H	-4.22530100	3.15692100	-1.51336900

QMD meso compressed conformation

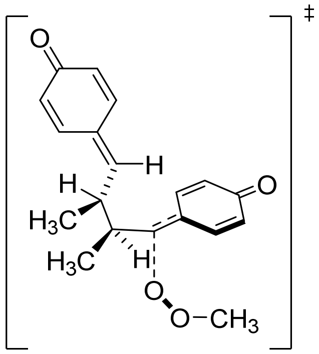

TS frequency: -366.74 cm^{-1}

DFT Enthalpy = -1037.239170 DFT Free Energy = -1037.319755

CBS-QB3 Enthalpy = -1035.560805 CBS-QB3 Free Energy = -1035.641773

0 2

C	0.46551200	-1.94857600	-0.12571600
H	0.08217800	-1.51263800	-1.05231800
C	-0.72971000	-1.89448800	0.91444300
H	-1.43847400	-2.63938200	0.54555300
C	-1.40431300	-0.55582600	0.95028000
H	-0.86379900	0.22380900	1.48226700
C	1.64692500	-1.14013500	0.35076800
H	2.26802900	-1.63866200	1.08862200
C	-2.60546300	-0.20778600	0.41846200
C	-3.46690500	-1.12023900	-0.32598800
C	-3.08740300	1.16149100	0.58108900
C	-4.64457200	-0.71917000	-0.84006900
H	-3.14386800	-2.14526300	-0.46610700
C	-4.26298400	1.57484800	0.07362800
H	-2.45299800	1.84938400	1.13174900
C	-5.14088100	0.66310800	-0.68209400
H	-5.28737700	-1.39413100	-1.39371700
H	-4.61623100	2.59233100	0.19496500
C	1.77730700	0.25191300	0.29507200
C	1.00548100	1.09077300	-0.59010400
C	2.76905400	0.89212900	1.12403400
C	1.17688300	2.43443000	-0.61843600
H	0.28022100	0.62839500	-1.24823500
C	2.94976800	2.23567700	1.11587900
H	3.36690800	0.26272900	1.77573800
C	2.15730900	3.10993800	0.24199000

H	0.60116100	3.06912500	-1.28214500
H	3.68104000	2.72112100	1.75186100
O	-6.21348800	1.02308600	-1.15075100
O	2.31159800	4.33189700	0.22554600
C	-0.32257400	-2.30785600	2.34281500
H	-1.20843800	-2.38550500	2.97692100
H	0.17890900	-3.27777400	2.35229800
H	0.34552300	-1.57264400	2.79938200
C	0.83854600	-3.41379000	-0.40998800
H	1.56378300	-3.47756800	-1.21832900
H	1.28058200	-3.89493500	0.46643300
H	-0.05166600	-3.98109300	-0.69405400
O	2.96912100	-1.61560900	-1.16403400
O	4.26074000	-1.41294000	-0.79575300
C	4.84360200	-0.37821400	-1.60778100
H	4.32559600	0.56669100	-1.44035100
H	5.88148100	-0.31158300	-1.28019000
H	4.79018600	-0.66324900	-2.66011200

EmpiricalDispersion = GD3

TS frequency: -344.70 cm^{-1}
DFT Enthalpy = -1037.289537 DFT Free Energy = -1037.368479

0 2

C	0.51425600	-2.09842500	-0.14854600
H	-0.01429200	-1.63302600	-0.98448100
C	-0.55388900	-2.27089300	1.00630600
H	-1.27111800	-2.99680000	0.61107100
C	-1.27277900	-0.98296300	1.28723200
H	-0.85742800	-0.37539700	2.08785900
C	1.62840600	-1.18891600	0.29736000
H	2.38173500	-1.66477400	0.91700400
C	-2.33683900	-0.47590700	0.61543100
C	-2.96903500	-1.15198900	-0.51152200
C	-2.85649800	0.83967400	0.97300100
C	-3.96934200	-0.57871900	-1.20645200
H	-2.61341000	-2.13654400	-0.79354300
C	-3.85628100	1.42423000	0.28768500
H	-2.39013600	1.35027300	1.80981400
C	-4.49420100	0.75999000	-0.86403700
H	-4.44214500	-1.07175500	-2.04808500
H	-4.23443400	2.40688300	0.54403800
C	1.57091300	0.20421200	0.37165500
C	0.60079500	1.00195400	-0.34151500
C	2.57455900	0.89851400	1.13739300
C	0.59712200	2.35303800	-0.25465000
H	-0.13507000	0.50278100	-0.95775900
C	2.58666300	2.25070800	1.23810700
H	3.32708300	0.30372600	1.64464600
C	1.58490200	3.07902700	0.55436800

H	-0.13177200	2.95692700	-0.78204400
H	3.32980300	2.77844700	1.82435900
O	-5.40181800	1.27949500	-1.50081800
O	1.58304000	4.30762500	0.64520300
C	0.03298400	-2.84839100	2.30428100
H	-0.76312200	-3.02364400	3.03154700
H	0.53914300	-3.79909000	2.12971500
H	0.74942400	-2.15778600	2.75783300
C	1.03101000	-3.46825200	-0.61398900
H	1.63499100	-3.36628200	-1.51369000
H	1.65516200	-3.94244100	0.14727200
H	0.19274700	-4.13587200	-0.83076800
O	2.84462400	-1.35861100	-1.37995300
O	4.01445100	-0.69618800	-1.21120300
C	4.02143000	0.49024500	-2.02735700
H	3.21826700	1.15890000	-1.71614000
H	4.99461400	0.95112300	-1.85622600
H	3.90251200	0.21297000	-3.07676100

QMD (S,S) extended conformation

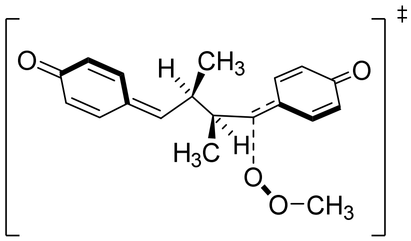

TS frequency: -372.03 cm^{-1}
DFT Enthalpy = -1037.238693 DFT Free Energy = -1037.318992

0 2

C	-0.88484000	-0.53863900	1.38396600
H	-1.56593300	-0.29204200	2.20179400
C	0.12217000	0.68246700	1.33568600
C	-1.66627900	-0.69365000	0.11174000
H	-1.08393100	-1.03901500	-0.74130300
C	2.16205700	-0.18187100	-0.02642600
C	2.91293500	-0.72040000	1.08185000
C	2.66291400	-0.43624800	-1.35501400
C	4.02465800	-1.46867400	0.88665700
H	2.58123400	-0.50496500	2.09016700
C	3.76693400	-1.19250300	-1.57195700
H	2.11588700	-0.01298500	-2.19171400
C	4.52958500	-1.77180400	-0.45913500
H	4.59477500	-1.87220200	1.71549800
H	4.13262600	-1.40216900	-2.57054600
C	-2.99406800	-0.50551600	-0.10179700
C	-3.93409100	-0.06275400	0.92275100
C	-3.54569900	-0.75750300	-1.43098300
C	-5.24176600	0.10980500	0.65488200

H	-3.56162900	0.13077500	1.92159100
C	-4.85020800	-0.58875600	-1.71210900
H	-2.85597300	-1.09206500	-2.20033900
C	-5.80862600	-0.13741700	-0.68637800
H	-5.94294300	0.43843800	1.41354100
H	-5.25575800	-0.77611900	-2.69963900
O	5.53528000	-2.45891600	-0.64203200
O	-6.99862400	0.02188300	-0.92669400
H	0.73078700	0.60060100	2.24139600
C	-0.66832900	1.99950100	1.39948000
H	0.00018500	2.85560700	1.45385300
H	-1.30211900	2.12181200	0.51687100
H	-1.31583000	2.00597500	2.28020500
C	-0.21424700	-1.87901600	1.74050500
H	0.46986800	-2.21808300	0.96019800
H	0.34990700	-1.79387400	2.67296200
H	-0.97417100	-2.65190700	1.87746700
C	1.02076400	0.61451900	0.12468300
H	0.55146200	0.93774100	-0.79991600
O	1.98775300	2.42343200	0.33721000
O	2.46056900	2.88376400	-0.85080100
C	3.89606000	2.96536500	-0.80618400
H	4.18615200	3.38116100	-1.77154700
H	4.20146900	3.62878900	0.00507800
H	4.32457400	1.97178300	-0.67039300

EmpiricalDispersion = GD3

TS frequency: -353.35 cm^{-1}
DFT Enthalpy = -1037.287999 DFT Free Energy = -1037.366495

0 2

C	-0.86868500	-0.59288000	1.49806400
H	-1.56041000	-0.33800700	2.30466900
C	0.04901100	0.68487800	1.33864500
C	-1.63753400	-0.88084600	0.24206300
H	-1.05668300	-1.36513600	-0.54143000
C	2.06643300	-0.16553700	-0.05399100
C	2.87285000	-0.61207100	1.05652800
C	2.55522100	-0.42737900	-1.38318100
C	4.03037300	-1.28666300	0.86268600
H	2.54146600	-0.38913000	2.06282100
C	3.70932200	-1.10652600	-1.59885600
H	1.96725600	-0.06538500	-2.22012200
C	4.53183000	-1.59199800	-0.48416000
H	4.64327400	-1.62440900	1.69015700
H	4.07593700	-1.31744800	-2.59658700
C	-2.93977900	-0.61978400	-0.03394600
C	-3.85514500	0.02567500	0.90151800
C	-3.48468500	-0.98761700	-1.33859100
C	-5.13524800	0.28060400	0.57418700

H	-3.48379000	0.30790200	1.87906200
C	-4.76228900	-0.73919400	-1.67796100
H	-2.81382400	-1.47470500	-2.04028800
C	-5.69478700	-0.07918400	-0.74487600
H	-5.81961300	0.76140000	1.26366300
H	-5.16592900	-1.00961700	-2.64669100
O	5.58432200	-2.20578200	-0.66677500
O	-6.85954800	0.15513200	-1.04035300
H	0.69605500	0.71100400	2.22017200
C	-0.82211100	1.95040500	1.33329400
H	-0.21053800	2.84843600	1.27962700
H	-1.50209100	1.95174700	0.47732400
H	-1.42661700	1.99508900	2.24282200
C	-0.09820700	-1.85013700	1.93443000
H	0.60283800	-2.18445200	1.16713200
H	0.46738600	-1.66033200	2.85016000
H	-0.79543300	-2.66786500	2.13141300
C	0.89587700	0.58216800	0.09671300
H	0.37066200	0.82702100	-0.82153700
O	1.77887400	2.45160000	0.16378300
O	2.48747200	2.70301400	-0.96441300
C	3.89505600	2.70480900	-0.66051700
H	4.39236000	2.92454500	-1.60558600
H	4.10853300	3.47943800	0.07889700
H	4.19517800	1.72583800	-0.28591300

QMD (S,S) compressed conformation

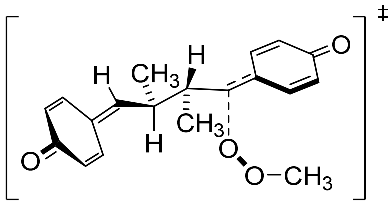

TS frequency: -352.59 cm^{-1}
DFT Enthalpy = -1037.238540 DFT Free Energy = -1037.319715

0 2

C	-0.91229900	-2.17532400	0.59666000
H	-1.30243800	-1.97390900	1.59874200
C	0.65322700	-2.30650600	0.72346800
C	1.31627900	-0.99258500	1.02190700
H	0.92889800	-0.47254800	1.89637800
C	-1.21968900	-1.00528600	-0.30950100
H	-0.88500800	-1.13439300	-1.33485200
C	2.36258500	-0.40983900	0.38135100
C	3.01678500	-0.97424900	-0.79397100
C	2.88725500	0.85953800	0.87676600
C	4.05641500	-0.35878400	-1.38778200
H	2.65499600	-1.91566900	-1.19088700
C	3.92443800	1.48596100	0.29166800

H	2.40231200	1.29277700	1.74628800
C	4.59766600	0.92328800	-0.89259900
H	4.54715800	-0.77634500	-2.25965600
H	4.30945200	2.42929500	0.66123700
C	-1.42435900	0.31564100	0.10212000
C	-1.80684900	0.68200900	1.44488200
C	-1.29294900	1.37740800	-0.86476300
C	-2.00455500	1.97479300	1.79699600
H	-1.95272700	-0.10004400	2.18058400
C	-1.47965500	2.67779900	-0.53059400
H	-1.01888600	1.10651600	-1.87901600
C	-1.84762000	3.07359900	0.83407300
H	-2.29474200	2.25567600	2.80294700
H	-1.36034600	3.47830300	-1.25146800
O	5.54418600	1.47893300	-1.43615200
O	-2.02273300	4.25055000	1.15208800
H	1.01383600	-2.69444100	-0.23332400
O	-3.16855300	-1.43536300	-0.88656700
O	-3.52838300	-0.73565600	-1.99366400
C	-4.60457100	0.16391600	-1.67519900
H	-4.85469700	0.65255600	-2.61731500
H	-5.45774400	-0.40321300	-1.29812200
H	-4.27549700	0.89649400	-0.93750400
C	1.05030100	-3.30993700	1.83005400
H	0.68308000	-2.97925500	2.80630300
H	0.65158200	-4.30643600	1.63557900
H	2.13747900	-3.39089900	1.89156500
C	-1.52166000	-3.49350100	0.09074600
H	-1.24345800	-4.32463400	0.73986700
H	-2.60758300	-3.43578600	0.06172000
H	-1.17626000	-3.71939200	-0.92241300

EmpiricalDispersion = GD3

TS frequency: -314.52 cm^{-1}
DFT Enthalpy = -1037.289774 DFT Free Energy = -1037.368559

0 2

C	-2.03038200	-1.66942100	0.25665900
H	-2.26835000	-1.40703800	1.29209200
C	-0.81569600	-2.68674100	0.29416500
C	0.39758900	-2.07061600	0.92039700
H	0.34855300	-1.97189700	2.00370000
C	-1.60590300	-0.43464700	-0.49535100
H	-1.59402000	-0.54879800	-1.57567100
C	1.49970600	-1.57249500	0.30606300
C	1.69984800	-1.60239100	-1.13819000
C	2.51863600	-0.89119700	1.09754000
C	2.75352800	-0.99926100	-1.71752500
H	0.97357700	-2.12070000	-1.75296000
C	3.57050400	-0.27249900	0.53119400

H	2.38138700	-0.86713900	2.17406400
C	3.76943400	-0.27107600	-0.92985800
H	2.90683400	-1.01348500	-2.79049600
H	4.30974600	0.26150400	1.11621500
C	-0.83682200	0.60722500	0.01858300
C	-0.71652700	0.87465600	1.43277200
C	-0.14289400	1.48467800	-0.88852400
C	0.06033100	1.87876500	1.89821400
H	-1.27162900	0.25654100	2.12777000
C	0.65338600	2.48675000	-0.44255600
H	-0.24906100	1.30082300	-1.95218400
C	0.82128900	2.75160800	0.99198500
H	0.15705700	2.08804200	2.95721500
H	1.20710500	3.12664700	-1.11915400
O	4.71525600	0.29471000	-1.46291100
O	1.54340100	3.65517100	1.41253000
H	-0.60575300	-2.95501900	-0.74524400
O	-3.44454000	0.58501300	-0.56634600
O	-3.36130600	1.66700500	-1.37795600
C	-3.36850300	2.86881100	-0.58569600
H	-3.33153300	3.68764200	-1.30484000
H	-4.28578900	2.91274300	0.00519200
H	-2.49377400	2.88868100	0.06528400
C	-1.17932500	-3.97407200	1.05889900
H	-1.51854800	-3.74065700	2.07311500
H	-1.96709200	-4.53531900	0.55613600
H	-0.30521400	-4.62440800	1.13712500
C	-3.26414300	-2.32372200	-0.38452300
H	-3.62699800	-3.15902200	0.21477700
H	-4.07044900	-1.60036000	-0.48599700
H	-3.02288200	-2.70010100	-1.38417000

α,α-Dimethyl quinone methide

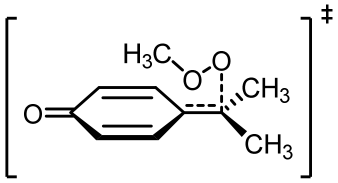

TS frequency: -337.98 cm^{-1}

DFT Enthalpy =	-614.330501	DFT Free Energy =	-614.387457
CBS-QB3 Enthalpy =	-613.350221	CBS-QB3 Free Energy =	-613.407390

0 2

C	-0.69112300	-0.00845700	1.23598700
C	-1.99582300	0.35992800	1.18710400
C	-2.83605900	0.07755200	0.01928000
C	-2.16784200	-0.62415300	-1.07996600

C	-0.86303200	-0.98387800	-1.01173400
H	-0.11478100	0.21877500	2.12351500
H	-2.47314000	0.87565500	2.01265000
H	-2.77210700	-0.83983900	-1.95369100
H	-0.41802100	-1.49646700	-1.85428100
C	-0.05002700	-0.70969700	0.14971400
O	-4.02148800	0.41276400	-0.03386700
O	2.15839200	0.58928700	-0.73135600
O	2.11528300	1.71881500	0.01658900
C	1.23210800	2.67796400	-0.59111700
H	1.29393300	3.56513000	0.03993900
H	1.57428700	2.90017200	-1.60354200
H	0.21365700	2.28783700	-0.60902500
C	1.30597000	-1.08827400	0.22759600
C	1.92789300	-2.02383200	-0.77233200
H	1.51564000	-1.92507200	-1.77413800
H	3.00119600	-1.83974400	-0.82852400
H	1.78144300	-3.05964200	-0.44250500
C	2.06815000	-0.95551800	1.52137000
H	1.99166500	0.04982500	1.93577400
H	1.68303200	-1.66277100	2.26481700
H	3.12441300	-1.17060200	1.36267100

α-Methyl quinone methide

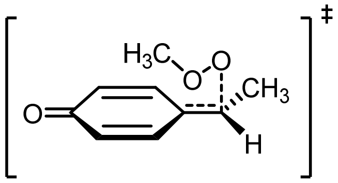

TS frequency: -378.13 cm^{-1}
DFT Enthalpy = -575.033441 DFT Free Energy = -575.087440
CBS-QB3 Enthalpy = -574.120188 CBS-QB3 Free Energy = -574.174372

0 2

C	0.58547500	-0.16243500	1.28738000
C	1.85222200	-0.60080000	1.08695100
C	2.64032200	-0.16158600	-0.07091100
C	1.96797600	0.77075800	-0.98585300
C	0.69961400	1.19443300	-0.76991400
H	0.01815000	-0.49900800	2.14933000
H	2.33817600	-1.28710700	1.77079800
H	2.54310600	1.09999500	-1.84363200
H	0.23622300	1.87969400	-1.46975600
C	-0.05310300	0.76002000	0.38133200
O	3.79211000	-0.55314200	-0.26666500
O	-2.49350100	-0.30796000	-0.30086900
O	-2.29724700	-1.55438200	0.19215400
C	-1.60614300	-2.36187800	-0.77643400

H	-1.53481300	-3.35215500	-0.32565000
H	-2.18362900	-2.39989100	-1.70202000
H	-0.61261700	-1.95151300	-0.96275900
C	-1.36566800	1.16106300	0.63526700
H	-1.77562000	0.86380300	1.59416600
C	-2.08732100	2.27657300	-0.04958800
H	-1.81534900	2.37313300	-1.10056100
H	-3.16417600	2.11452000	0.01333900
H	-1.86058200	3.22550800	0.45041800

SCRF = (CPCM, solvent=chlorobenzene)

TS frequency: -389.90 cm^{-1}
DFT Enthalpy = -575.042660 DFT Free Energy = -575.096858
CBS-QB3 Enthalpy = -574.128947 CBS-QB3 Free Energy = -574.183329

C	-0.58709100	0.17542000	1.28047900
C	-1.85388600	0.61733800	1.08005500
C	-2.64159200	0.17634800	-0.07267400
C	-1.97848900	-0.75895200	-0.98523700
C	-0.71053800	-1.18856400	-0.77034600
H	-0.02046100	0.51168400	2.14254400
H	-2.33183400	1.30800400	1.76531400
H	-2.55228800	-1.09124900	-1.84287500
H	-0.25361800	-1.87862200	-1.46909300
C	0.04410700	-0.75297300	0.37739900
O	-3.79950700	0.57158400	-0.26704000
O	2.47382300	0.27882400	-0.33350500
O	2.34699300	1.52558800	0.18222600
C	1.65660500	2.38165200	-0.74870200
H	1.64355500	3.36417300	-0.27748700
H	2.20301300	2.41320700	-1.69259000
H	0.64131300	2.01523400	-0.90453500
C	1.35322800	-1.16990800	0.63676700
H	1.76536600	-0.86866600	1.59339100
C	2.05393200	-2.30613000	-0.03230600
H	1.78808300	-2.41071300	-1.08398200
H	3.13390700	-2.17578800	0.04453300
H	1.79483200	-3.24061900	0.47871500

EmpiricalDispersion = GD3

TS frequency: -370.22 cm^{-1}
DFT Enthalpy = -575.053879 DFT Free Energy = -575.107157

0 2

C	-0.51930700	0.07795200	1.32599100
C	-1.80397700	0.46096800	1.12441900
C	-2.56148200	0.01209400	-0.04990800
C	-1.84115900	-0.86923600	-0.97926500
C	-0.55486700	-1.23424200	-0.76435400

H	0.02785900	0.42885400	2.19470700
H	-2.32674000	1.11162300	1.81562200
H	-2.39663200	-1.20654400	-1.84660600
H	-0.05365300	-1.87986300	-1.47554400
C	0.16896700	-0.78646200	0.40078800
O	-3.72743800	0.35776700	-0.24877600
O	2.45267100	0.55671600	-0.23772600
O	1.94574000	1.73781300	0.18409400
C	1.11569000	2.30485800	-0.84642800
H	0.76386300	3.25549200	-0.44475300
H	1.70908100	2.46006100	-1.74988500
H	0.27622900	1.63913800	-1.05109500
C	1.51118600	-1.08533600	0.62957500
H	1.90623100	-0.79629200	1.59725500
C	2.33065900	-2.08119100	-0.12453900
H	2.05283900	-2.14300400	-1.17640100
H	3.38579300	-1.80952800	-0.06491800
H	2.21309600	-3.07473700	0.32321200

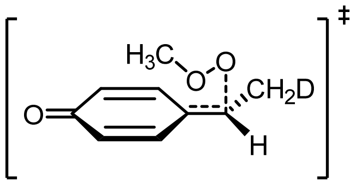

TS frequency: -378.04 cm^{-1}

DFT Enthalpy =	-575.036512	DFT Free Energy =	-575.090817
CBS-QB3 Enthalpy =	-574.123226	CBS-QB3 Free Energy =	-574.177718

0 2

C	-0.58547500	0.16243500	1.28738000
C	-1.85222200	0.60080000	1.08695100
C	-2.64032200	0.16158600	-0.07091100
C	-1.96797600	-0.77075800	-0.98585300
C	-0.69961400	-1.19443300	-0.76991400
H	-0.01815000	0.49900800	2.14933000
H	-2.33817600	1.28710700	1.77079800
H	-2.54310600	-1.09999500	-1.84363200
H	-0.23622300	-1.87969400	-1.46975600
C	0.05310300	-0.76002000	0.38133200
O	-3.79211000	0.55314100	-0.26666500
O	2.49350100	0.30796000	-0.30086900
O	2.29724700	1.55438200	0.19215400
C	1.60614300	2.36187800	-0.77643400
H	1.53481300	3.35215500	-0.32565000
H	2.18362900	2.39989100	-1.70202000
H	0.61261700	1.95151300	-0.96275900
C	1.36566800	-1.16106300	0.63526700
H	1.77562000	-0.86380300	1.59416600
C	2.08732100	-2.27657300	-0.04958800
H(Iso=2)	1.81534900	-2.37313300	-1.10056100

H	3.16417600	-2.11452000	0.01333900
H	1.86058200	-3.22550800	0.45041800

Quinone methide

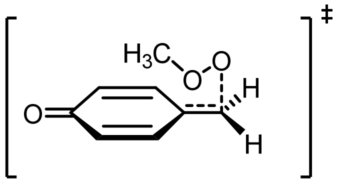

TS frequency: -398.55 cm^{-1}

DFT Enthalpy =	-535.732863	DFT Free Energy =	-535.783535
CBS-QB3 Enthalpy =	-534.888357	CBS-QB3 Free Energy =	-534.939186

0 2

C	-0.45041900	0.01556400	1.30466200
C	-1.62512600	0.65330900	1.08994600
C	-2.45029800	0.36868700	-0.09423100
C	-1.92241900	-0.64414000	-1.02078000
C	-0.74581000	-1.26996000	-0.78982200
H	0.14628200	0.23693500	2.18397800
H	-2.01188800	1.39594500	1.77823400
H	-2.52760500	-0.85911000	-1.89381000
H	-0.36747100	-2.01103500	-1.48681900
C	0.04444400	-0.98077400	0.38440700
O	-3.51757000	0.94655200	-0.29732900
O	2.67913300	-0.49229300	-0.40069300
O	2.86575800	0.72254400	0.16555700
C	2.32234600	1.75042000	-0.68164700
H	2.57876600	2.69011100	-0.19168200
H	2.78056700	1.69207600	-1.67039700
H	1.23952200	1.63716100	-0.75539900
C	1.26119700	-1.60444700	0.60106700
H	1.55936800	-2.44792300	-0.00747300
H	1.78040200	-1.48053800	1.54147900

α-Phenyl quinone methide

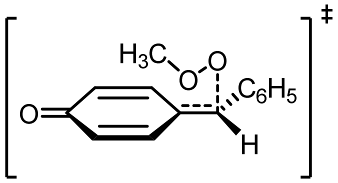

TS frequency: -402.30 cm^{-1}

DFT Enthalpy =	-766.755867	DFT Free Energy =	-766.817105
CBS-QB3 Enthalpy =	-765.505029	CBS-QB3 Free Energy =	-765.566515

0 2

C	-1.89032800	0.09559400	1.29464700
C	-3.12461000	-0.41571900	1.05421500
C	-3.38043800	-1.28738200	-0.09710400
C	-2.23050000	-1.54453000	-0.97119900
C	-1.00156200	-1.03593100	-0.70607100
H	-1.72179100	0.73966500	2.15186100
H	-3.96923100	-0.20500000	1.69996900
H	-2.42003700	-2.14989200	-1.85015900
H	-0.18124800	-1.21224600	-1.39047500
C	-0.76423800	-0.21750900	0.45599700
O	-4.49728600	-1.75866600	-0.32623600
O	0.44753100	2.00158600	-0.43831200
O	-0.41405500	2.94963100	0.02603000
C	-1.51043600	3.10038300	-0.89012800
H	-2.11788700	3.90688800	-0.47774100
H	-1.13187600	3.37089900	-1.87777700
H	-2.08628900	2.17530200	-0.94328800
C	0.48710600	0.34883200	0.76322600
H	0.50190800	0.99508000	1.63430800
C	1.80803000	-0.16826200	0.35892500
C	2.90966300	0.70367600	0.31959400
C	2.02497200	-1.53145200	0.10150100
C	4.17609500	0.23238500	-0.00086800
H	2.75077900	1.75638800	0.51387900
C	3.29756500	-2.00149700	-0.21010800
H	1.20442500	-2.23271700	0.18282000
C	4.37500200	-1.12170700	-0.27019700
H	5.01184000	0.92183300	-0.03819800
H	3.44678700	-3.05888800	-0.39663500
H	5.36515400	-1.48899900	-0.51499800

α,α-Dicyano quinone methide

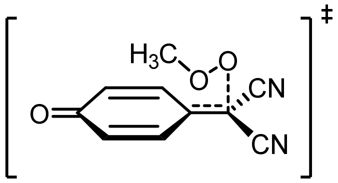

TS frequency: -542.88 cm^{-1}

DFT Enthalpy = -720.237352 DFT Free Energy = -720.295868

CBS-QB3 Enthalpy = -719.117482 CBS-QB3 Free Energy = -719.176214

0 2

C	0.83015500	-0.24888200	1.22207900
C	2.18218300	-0.40167600	1.18813400
C	2.99332500	0.20127600	0.12747100
C	2.27035500	0.97597300	-0.88541200
C	0.92054100	1.11485800	-0.83988000
H	0.24604300	-0.69708500	2.01687600

H	2.71204900	-0.96906000	1.94382200
H	2.86607000	1.43187500	-1.66704400
H	0.40059200	1.68977000	-1.59733900
C	0.15785800	0.51272700	0.21508100
O	4.21927000	0.06234400	0.08912300
O	-1.92198000	-0.88086500	-0.81429600
O	-1.43169000	-2.06967000	-0.37278100
C	-0.49348300	-2.59992400	-1.32892600
H	-0.21092900	-3.57319600	-0.92793000
H	-0.98168100	-2.70840000	-2.29832700
H	0.37848600	-1.94970600	-1.40895200
C	-1.27228000	0.61198900	0.22782500
C	-1.90898300	1.59701500	-0.60617000
C	-1.97693400	0.32168600	1.44856500
N	-2.52349700	0.09573400	2.43873100
N	-2.41248300	2.38584900	-1.28017100

α,α-Diphenyl quinone methide

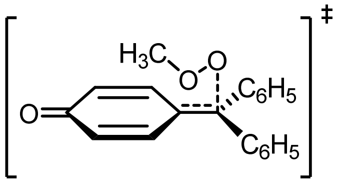

TS frequency: -391.52 cm^{-1}

DFT Enthalpy = -997.772343 DFT Free Energy = -997.843173

CBS-QB3 Enthalpy = NA CBS-QB3 Free Energy = NA

0 2

C	-1.92557400	-1.01056600	-0.17496800
C	-3.26022800	-0.96971100	-0.42906000
C	-3.91493100	0.24459700	-0.91652100
C	-3.04049700	1.40168700	-1.10320100
C	-1.71075500	1.34915900	-0.82418300
H	-1.47497300	-1.91764000	0.20545200
H	-3.89546400	-1.83433200	-0.27331200
H	-3.50298400	2.30090600	-1.49371900
H	-1.09371000	2.21998800	-1.00091800
C	-1.08473000	0.14412100	-0.35432400
O	-5.12743500	0.28789300	-1.15558600
O	0.17722700	0.14248200	1.96974700
O	-0.44633200	-0.92893800	2.55352800
C	-1.62749000	-0.48132300	3.23475900
H	-2.05987700	-1.38023000	3.67644600
H	-1.35505400	0.23211200	4.01589000
H	-2.32647300	-0.02779300	2.53076000
C	0.31601700	0.06460600	-0.06309300
C	1.02541200	-1.24131100	-0.25051900
C	2.04693500	-1.64270100	0.62216300

C	0.74684600	-2.03898700	-1.36963700
C	2.75133600	-2.81802200	0.39183200
H	2.25918300	-1.03563200	1.49146700
C	1.46289500	-3.21104800	-1.60473200
H	-0.02126700	-1.73213000	-2.06924100
C	2.46361900	-3.60660300	-0.72238500
H	3.52722600	-3.12158200	1.08557100
H	1.23659600	-3.81099600	-2.47886500
H	3.01737600	-4.52148100	-0.90074600
C	1.17921200	1.28054900	-0.14597700
C	0.88024500	2.44606400	0.58003400
C	2.31719600	1.28110300	-0.96728700
C	1.68887700	3.57267900	0.47807800
H	0.02977700	2.44874800	1.24733300
C	3.11401600	2.41618800	-1.08024300
H	2.57165900	0.39212100	-1.53007200
C	2.80485500	3.56514900	-0.35664600
H	1.44855000	4.45747400	1.05666700
H	3.98060400	2.39912800	-1.73134600
H	3.43161100	4.44606800	-0.43742000

2,6-di-tert-butyl-α-methyl quinone methide

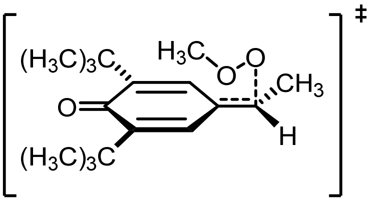

DFT Enthalpy =	-997.772343	DFT Free Energy =	-997.843173
CBS-QB3 Enthalpy =	-887.939513	CBS-QB3 Free Energy =	-888.017065

0 2

C	-1.00465500	0.76255500	-0.76239200
C	0.13238800	1.38576400	-0.34924700
C	1.31173400	0.54477100	-0.00229100
C	1.19618100	-0.93777900	-0.11591500
C	0.01582300	-1.46896900	-0.53401500
H	-1.88237500	1.34016200	-1.02229700
H	-0.08919300	-2.54082100	-0.61955800
C	-1.11877500	-0.66264600	-0.88487400
O	2.36666800	1.06594500	0.37644100
O	-3.42021900	-1.41390300	0.46258000
O	-3.60068300	-0.26068900	1.15123300
C	-2.80652000	-0.27765700	2.34892500
H	-3.04732000	0.65097000	2.86750800
H	-3.07948900	-1.14121600	2.95864800
H	-1.74656500	-0.31168700	2.09248100
C	-2.33402400	-1.20292200	-1.31004400
H	-3.07133200	-0.49097700	-1.66401400

C	0.23934600	2.91457100	-0.23697300
C	1.35298100	3.43651800	-1.17857800
H	1.40402600	4.52802400	-1.11776300
H	2.32384700	3.02775900	-0.90581000
H	1.13843800	3.16861400	-2.21756400
C	0.56009800	3.31697500	1.22400500
H	1.51279300	2.90245800	1.54730900
H	0.60885900	4.40739500	1.30275000
H	-0.22411100	2.96809600	1.90307800
C	-1.07675600	3.60943300	-0.63630400
H	-1.35152700	3.40470700	-1.67506000
H	-1.91117700	3.31339700	0.00602000
H	-0.95426300	4.69090500	-0.53750100
C	2.40995700	-1.81160300	0.23974700
C	3.60211500	-1.46099200	-0.68505300
H	3.90775000	-0.42437200	-0.55894500
H	4.45315900	-2.10709100	-0.44850500
H	3.33908300	-1.62433700	-1.73458700
C	2.81096800	-1.58504900	1.71871200
H	3.65763900	-2.23015600	1.97293500
H	3.09761600	-0.55050000	1.89585000
H	1.98352500	-1.84071600	2.38784400
C	2.10651900	-3.31195600	0.06275300
H	1.29323300	-3.64781800	0.71279100
H	1.84960300	-3.56126000	-0.97094800
H	2.99568400	-3.88981600	0.32677500
C	-2.57160000	-2.62116500	-1.71743000
H	-1.97391100	-3.33178300	-1.14692000
H	-3.62472600	-2.87262700	-1.58318100
H	-2.33006600	-2.74722400	-2.77952100

Truncated dimer

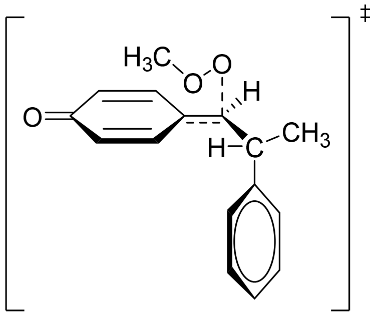

TS frequency: -343.37 cm^{-1}

DFT Enthalpy = -845.342813 DFT Free Energy = -845.411134

CBS-QB3 Enthalpy = -843.960367 CBS-QB3 Free Energy = -844.028986

0 2

C	0.96314500	-0.76857400	-0.75517100
C	1.83634300	-1.75262400	-1.08048400

C	2.84270300	-2.24399300	-0.12820700
C	2.84016200	-1.60372700	1.19251200
C	1.96163900	-0.62042300	1.49770000
H	0.22717400	-0.43962100	-1.47830900
H	1.82743000	-2.22622800	-2.05555400
H	3.57672000	-1.95858700	1.90398000
H	1.98057100	-0.15155200	2.47654100
C	0.97599200	-0.15653500	0.55037100
O	3.63885800	-3.13567600	-0.42560300
O	1.35833300	2.52336000	0.56707000
O	1.45649800	2.81734500	-0.75543500
C	2.78161800	2.51858200	-1.22898300
H	2.79283600	2.84364900	-2.26985300
H	3.51406800	3.07645700	-0.64293800
H	2.97189500	1.44691100	-1.15775400
C	0.11111800	0.87785900	0.91263100
C	-1.10441000	1.33029600	0.13304000
H	-0.76303100	1.59726800	-0.87257200
H	0.14440000	1.17923200	1.95527200
C	-1.73255800	2.58771200	0.76472600
H	-2.57633300	2.93237100	0.16356300
H	-2.10800700	2.36997500	1.76906300
H	-0.99437700	3.38714100	0.83252300
C	-2.14961600	0.22465600	-0.01552300
C	-2.75441500	-0.00316100	-1.25515700
C	-2.56682600	-0.54188100	1.07849300
C	-3.74636600	-0.97085000	-1.40242800
H	-2.44737600	0.58550300	-2.11425100
C	-3.55574100	-1.51108400	0.93481700
H	-2.11460600	-0.38633700	2.05227600
C	-4.14944200	-1.72962700	-0.30691000
H	-4.20104800	-1.13240100	-2.37351500
H	-3.86193900	-2.09794800	1.79366400
H	-4.91795600	-2.48583000	-0.41895600

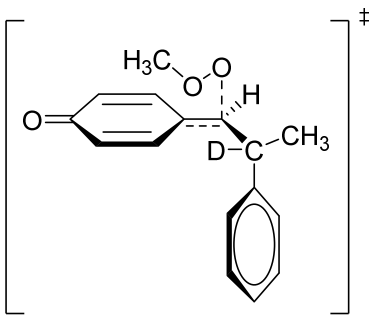

TS frequency: -342.98 cm^{-1}

DFT Enthalpy = -845.346182 DFT Free Energy = -845.414668

CBS-QB3 Enthalpy = -843.963699 CBS-QB3 Free Energy = -844.032487

0 2

C	-0.96314500	-0.76857400	0.75517100

C	-1.83634300	-1.75262400	1.08048400
C	-2.84270300	-2.24399300	0.12820700
C	-2.84016200	-1.60372700	-1.19251200
C	-1.96163900	-0.62042300	-1.49770000
H	-0.22717400	-0.43962100	1.47830900
H	-1.82743000	-2.22622800	2.05555400
H	-3.57672000	-1.95858700	-1.90398000
H	-1.98057100	-0.15155200	-2.47654100
C	-0.97599200	-0.15653500	-0.55037100
O	-3.63885800	-3.13567600	0.42560300
O	-1.35833300	2.52336000	-0.56707000
O	-1.45649800	2.81734500	0.75543500
C	-2.78161800	2.51858200	1.22898300
H	-2.79283600	2.84364900	2.26985300
H	-3.51406800	3.07645700	0.64293800
H	-2.97189500	1.44691100	1.15775400
C	-0.11111800	0.87785900	-0.91263100
C	1.10441000	1.33029600	-0.13304000
H(Iso=2)	0.76303100	1.59726800	0.87257200
H	-0.14440000	1.17923200	-1.95527200
C	1.73255800	2.58771200	-0.76472600
H	2.57633300	2.93237100	-0.16356300
H	2.10800700	2.36997500	-1.76906300
H	0.99437700	3.38714100	-0.83252300
C	2.14961600	0.22465600	0.01552300
C	2.75441500	-0.00316100	1.25515700
C	2.56682600	-0.54188100	-1.07849300
C	3.74636600	-0.97085000	1.40242800
H	2.44737600	0.58550300	2.11425100
C	3.55574100	-1.51108400	-0.93481700
H	2.11460600	-0.38633700	-2.05227600
C	4.14944200	-1.72962700	0.30691000
H	4.20104800	-1.13240100	2.37351500
H	3.86193900	-2.09794800	-1.79366400
H	4.91795600	-2.48583000	0.41895600

C–H HAT TS

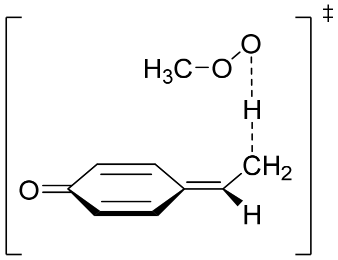

TS frequency: -1730.60 cm^{-1}

DFT Enthalpy =	-575.024938	DFT Free Energy =	-575.079085
CBS-QB3 Enthalpy =	-574.107997	CBS-QB3 Free Energy =	-574.162329

0 2

C	-1.14668300	0.73565700	-1.29790700
C	-2.35174300	0.13149800	-1.17956800
C	-2.83426100	-0.36669800	0.11469100
C	-1.93355800	-0.15590400	1.25912000
C	-0.73319400	0.45387200	1.12070100
H	-0.80415000	1.09162600	-2.26472700
H	-0.10187700	0.59827000	1.98997600
C	-0.26967500	0.92344600	-0.16488500
C	0.96361500	1.53579200	-0.35201100
H	1.19470900	1.83671800	-1.37080700
O	-3.92360500	-0.92475300	0.23994500
C	1.99585800	1.73711200	0.61402200
H	2.76765700	0.72750700	0.45679000
O	3.46609600	-0.33417200	0.22629500
O	2.70970900	-1.02291300	-0.69195000
C	2.02052600	-2.09792200	-0.03960800
H	1.31620400	-1.70976200	0.70033700
H	1.48368100	-2.61682600	-0.83454200
H	2.74188200	-2.76485600	0.43645400
H	2.69273200	2.54293100	0.38837900
H	1.72350900	1.69209500	1.66590500
H	-3.00838700	-0.01384600	-2.02935100
H	-2.28887900	-0.51027100	2.21993500

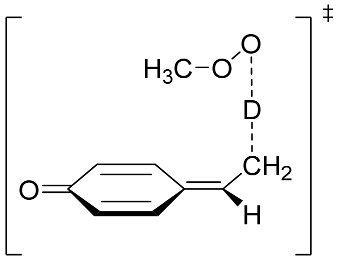

TS frequency: -1323.87 cm^{-1}

DFT Enthalpy =	-575.026511	DFT Free Energy =	-575.080861
CBS-QB3 Enthalpy =	-574.109551	CBS-QB3 Free Energy =	-574.164089

0 2

C	-1.14668300	-0.73565700	1.29790700
C	-2.35174300	-0.13149800	1.17956800
C	-2.83426100	0.36669800	-0.11469100
C	-1.93355800	0.15590400	-1.25912000
C	-0.73319400	-0.45387200	-1.12070100
H	-0.80415000	-1.09162600	2.26472700
H	-0.10187700	-0.59827000	-1.98997600
C	-0.26967500	-0.92344600	0.16488500
C	0.96361500	-1.53579200	0.35201100
H	1.19470900	-1.83671800	1.37080700
O	-3.92360500	0.92475200	-0.23994500
C	1.99585800	-1.73711200	-0.61402200
H(Iso=2)	2.76765700	-0.72750700	-0.45679000
O	3.46609600	0.33417200	-0.22629500
O	2.70970900	1.02291300	0.69195000
C	2.02052600	2.09792200	0.03960800
H	1.31620400	1.70976200	-0.70033700
H	1.48368100	2.61682600	0.83454200
H	2.74188200	2.76485600	-0.43645400
H	2.69273200	-2.54293100	-0.38837900
H	1.72350900	-1.69209500	-1.66590500
H	-3.00838700	0.01384600	2.02935100
H	-2.28887900	0.51027100	-2.21993500

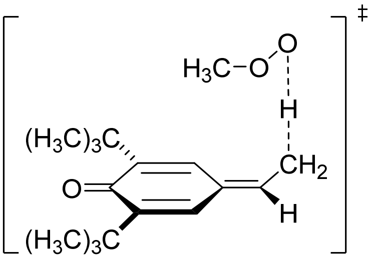

TS frequency: -1706.16 cm^{-1}

DFT Enthalpy = -889.385349 DFT Free Energy = -889.462844

CBS-QB3 Enthalpy = -887.927370 CBS-QB3 Free Energy = -888.005260

0 2

C	-0.17549100	-1.46033500	-0.72444900
C	-1.35084000	-1.00600800	-0.21587200
C	-1.48040600	0.45124500	0.06643800
C	-0.32567600	1.34998300	-0.23635200
C	0.80944100	0.80120200	-0.74474800
H	-0.04121000	-2.51384200	-0.93559500
H	1.65351500	1.43561700	-0.97534300
C	0.94710300	-0.60609700	-1.00274100
C	2.11292300	-1.17048600	-1.51073700
H	2.09227100	-2.24718200	-1.66002900
O	-2.52320600	0.91111800	0.54022000
C	3.35798200	-0.52293100	-1.77829600
H	4.04642700	-0.72016900	-0.72263300

O	4.65202000	-0.97550600	0.39827000
O	3.68396500	-1.60612800	1.14492700
C	3.13463600	-0.68513700	2.09571600
H	2.63020900	0.13884400	1.58485400
H	2.41515600	-1.26612100	2.67410200
H	3.92747700	-0.30268900	2.74172200
H	3.99077400	-1.02175900	-2.51122900
H	3.35443900	0.55910800	-1.88802500
C	-2.53749200	-1.93663600	0.07883900
C	-3.76773200	-1.50386100	-0.75702900
H	-4.59948600	-2.18923200	-0.56753900
H	-4.08491600	-0.49504200	-0.50072600
H	-3.54130600	-1.53972200	-1.82694100
C	-2.88614200	-1.88826700	1.58723900
H	-3.18131100	-0.88651000	1.89282400
H	-3.71330600	-2.57390300	1.79501500
H	-2.03076800	-2.20308800	2.19281500
C	-2.21748500	-3.40014900	-0.28164800
H	-1.99236800	-3.52270200	-1.34507900
H	-1.37724200	-3.79347500	0.29787300
H	-3.08799000	-4.02170300	-0.05799500
C	-0.46263500	2.85739400	0.02880100
C	-0.73052400	3.10809900	1.53391100
H	-0.79909900	4.18425100	1.71978700
H	-1.65970400	2.64086400	1.85326000
H	0.08850700	2.71534600	2.14437300
C	-1.62607700	3.43765600	-0.81387000
H	-2.57623300	2.98228300	-0.54271500
H	-1.69632700	4.51679800	-0.64696300
H	-1.45020300	3.27473100	-1.88146100
C	0.81804900	3.62463100	-0.35286500
H	1.68523100	3.29667700	0.22789400
H	1.05569600	3.52712700	-1.41619000
H	0.67172100	4.68780600	-0.14742600

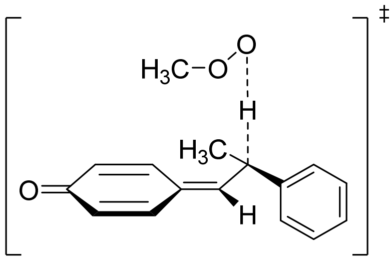

TS frequency: -1557.85 cm^{-1}

DFT Enthalpy =	-845.333200	DFT Free Energy =	-845.402944
CBS-QB3 Enthalpy =	-843.945793	CBS-QB3 Free Energy =	-844.015840

0 2

C	2.25564500	-0.81459400	-1.31202000

C	3.59164400	-1.00867700	-1.27621500
C	4.34095000	-0.94392700	-0.01493100
C	3.53661400	-0.67398800	1.18686700
C	2.19873300	-0.47972300	1.13376700
H	1.72389600	-0.86184400	-2.25735800
H	1.65699900	-0.30200500	2.05014700
C	1.47427800	-0.52309700	-0.12151100
C	0.11527300	-0.32322000	-0.28906400
H	-0.22338500	-0.43776100	-1.31608500
O	5.55794900	-1.10835800	0.03910700
C	-0.95338200	0.11339200	0.59345900
H	-0.98829400	1.32936100	0.26126100
O	-0.99624200	2.63019100	-0.02807000
O	-0.04681900	2.76958300	-1.00881500
C	1.15191800	3.32650200	-0.45026100
H	1.58793500	2.64444600	0.28311000
H	1.82813900	3.45106200	-1.29669400
H	0.93127600	4.29146200	0.01023200
C	-0.78138600	0.19480600	2.11318300
H	-0.60022900	-0.79575900	2.54330900
H	0.03185600	0.86149900	2.40191900
H	-1.69744200	0.58282100	2.56083200
C	-2.32405400	-0.39026600	0.18827000
C	-3.44985900	0.43555600	0.30836200
C	-2.50351700	-1.70092100	-0.27188600
C	-4.71588900	-0.03568000	-0.02300300
H	-3.32776700	1.46076400	0.63874700
C	-3.77214400	-2.17439200	-0.60011000
H	-1.64639200	-2.36002900	-0.35910000
C	-4.88216500	-1.34322800	-0.47742900
H	-5.57354200	0.62163600	0.06581100
H	-3.89129700	-3.19433200	-0.94836100
H	-5.86931600	-1.70967500	-0.73514600
H	4.16560400	-1.21728600	-2.17165600
H	4.07690800	-0.64693800	2.12640000

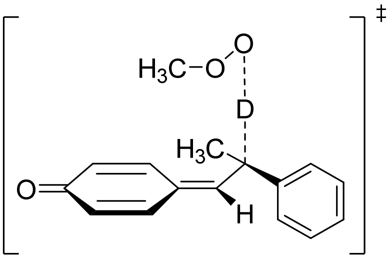

TS frequency: -1188.31 cm^{-1}

DFT Enthalpy = -845.334883 DFT Free Energy = -845.404783

CBS-QB3 Enthalpy = -843.947456 CBS-QB3 Free Energy = -844.017663

0 2

C	2.25564600	-0.81460000	-1.31201700
C	3.59164600	-1.00867400	-1.27621300

C	4.34095300	-0.94391700	-0.01492900
C	3.53661700	-0.67396800	1.18686800
C	2.19873500	-0.47971300	1.13376800
H	1.72389600	-0.86185700	-2.25735300
H	1.65700200	-0.30199000	2.05014700
C	1.47427900	-0.52310100	-0.12150900
C	0.11527300	-0.32322900	-0.28906000
H	-0.22338600	-0.43776900	-1.31608100
O	5.55795100	-1.10835100	0.03911000
C	-0.95338100	0.11338100	0.59346500
H(Iso=2)	-0.98828400	1.32935300	0.26128100
O	-0.99623000	2.63018600	-0.02803300
O	-0.04684100	2.76958500	-1.00881000
C	1.15192200	3.32648100	-0.45029000
H	1.58795200	2.64441300	0.28306200
H	1.82811900	3.45103800	-1.29674300
H	0.93131000	4.29144100	0.01021800
C	-0.78138800	0.19477900	2.11318900
H	-0.60022100	-0.79578900	2.54330400
H	0.03184800	0.86147600	2.40193400
H	-1.69744800	0.58278000	2.56084200
C	-2.32405400	-0.39026600	0.18826700
C	-3.44985500	0.43556000	0.30836100
C	-2.50352300	-1.70091900	-0.27189500
C	-4.71588700	-0.03566700	-0.02300800
H	-3.32775700	1.46076600	0.63875200
C	-3.77215300	-2.17438100	-0.60012300
H	-1.64640200	-2.36003100	-0.35911200
C	-4.88216900	-1.34321200	-0.47744000
H	-5.57353600	0.62165300	0.06580700
H	-3.89131100	-3.19431900	-0.94837900
H	-5.86932200	-1.70965200	-0.73515900
H	4.16560700	-1.21728300	-2.17165300
H	4.07691400	-0.64690600	2.12640000

Cyclization TS

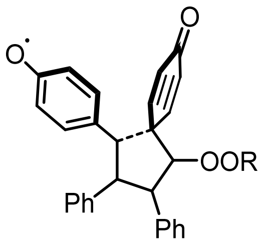

TS frequency: -499.33 cm^{-1}

DFT Enthalpy = -1420.693781 DFT Free Energy = -1420.785775

0 2

C	2.52477800	0.32623000	-1.20000600
C	2.86191300	-0.08519500	-2.49386200

C	4.18286200	-0.06799000	-2.93497400
C	5.19335500	0.37255600	-2.08455700
C	4.87053600	0.79310700	-0.79606300
C	3.54994500	0.76818700	-0.35729100
H	2.07860500	-0.41575400	-3.16960700
H	4.41934200	-0.38902500	-3.94327500
H	6.22260900	0.39436200	-2.42431300
H	5.64965600	1.14411000	-0.12877500
H	3.32358800	1.10077900	0.64807000
C	1.07699300	0.24774100	-0.76059600
H	0.46952200	0.28812300	-1.67089900
C	0.67092300	-1.09412900	-0.04696100
H	0.65363300	-1.85279000	-0.83157400
C	1.66690200	-1.59257100	0.99523500
C	2.57937700	-2.58916400	0.63174500
C	1.72449100	-1.09733000	2.30376700
C	3.52226100	-3.06993500	1.53572400
H	2.55452300	-2.99010800	-0.37577400
C	2.66677500	-1.57487300	3.21237600
H	1.02888700	-0.33557200	2.63548000
C	3.57075600	-2.56274500	2.83161600
H	4.21680900	-3.84291200	1.22690900
H	2.68938300	-1.17590500	4.22030300
H	4.30139100	-2.93716400	3.53932800
C	-0.73916300	-0.95428100	0.51905600
H	-0.79706600	-0.96093400	1.59962300
C	-1.88913100	-1.49620400	-0.11264900
C	-1.92982300	-1.84238100	-1.50472300
C	-3.09243500	-1.65655900	0.64941200
C	-3.05924900	-2.33018900	-2.08274200
H	-1.03727400	-1.71719000	-2.10744100
C	-4.23688300	-2.13982500	0.09136600
H	-3.07157300	-1.38786800	1.70010700
C	-4.29425500	-2.51748700	-1.31877200
H	-3.09311800	-2.60554800	-3.13052100
H	-5.14585500	-2.26507900	0.66761200
C	-0.91466700	1.22727000	0.42785700
C	-1.83500500	1.49463100	-0.66151300
C	-1.40008500	1.52012000	1.75939000
C	-3.12664700	1.82344000	-0.44470200
H	-1.46715500	1.40671800	-1.67788200
C	-2.68994500	1.84771700	2.00532400
H	-0.69130400	1.45946400	2.58058600
C	-3.66287500	2.00383300	0.91448200
H	-3.81896800	1.99409900	-1.26112000
H	-3.05101700	2.04636100	3.00797000
O	-5.32073900	-2.97071600	-1.84012000
O	-4.83795000	2.29743600	1.12247500
C	0.57891200	1.38519900	0.14153600
H	1.12877900	1.39000900	1.08769000
O	0.86514700	2.59757600	-0.54178400

O	0.69492600	3.66555500	0.44678500
C	0.25785500	4.79959100	-0.28201400
H	0.18826600	5.59072100	0.46769600
H	0.98838900	5.08078400	-1.04674000
H	-0.72194800	4.62892900	-0.73706900

References and notes

1. A. Nishinaga, H. Iwasaki, T. Shimizu, Y. Toyoda, T. Matsuura, *J. Org. Chem.*, 1986, **51**, 2257.
2. H. D. Becker, *J. Org. Chem.*, 1969, **34**, 1211.
3. C. D. Cook, B. E. Norcross, *J. Am. Chem. Soc.*, 1956, **78**, 3797. (b) C. E. Cook, B. E. Norcross, *J. Am. Chem. Soc.*, 1958, **81**, 1176. (c) L. K. Dyall, S. Winstein, *J. Am. Chem. Soc.*, 1972, **94**, 2196. (d) J. A. Richards, D. H. Evans, *J. Electroanal. Chem.*, 1977, **81**, 171.
4. (a) A. P. Krysin, A. N. Fedorenko, Novosibirsk Institute of Organic Chemistry, Siberian Branch, Academy of Sciences of the USSR. Translated from Izvestiya Akademii Nauk SSSR, Seriya Khimicheskaya, No. 7, pp. 1671-1674, July, 1989. (b) T. Matsuura, Y. Kitaura, *Tetrahedron*, 1969, **25**, 4501. (c) Volodkin et al.[Bulletin of the Academy of Sciences of the USSR, Division of Chemical Science (English Translation), 1969, 1467,1472.
5. (a) D. Richter, N. Hampel, T. Singer, A. R. Ofial, H. Mayr, *Eur. J. Org. Chem.*, 2009, 3203. (b) V. Reddy, R. V. Anand, *Org. Lett.*, 2015, **17**, 3390.
6. M. J. Frisch, G. W. Trucks, H. B. Schlegel, G. E. Scuseria, M. A. Robb, J. R. Cheeseman, G. Scalmani, V. Barone, G. A. Petersson, H. Nakatsuji, X. Li, M. Caricato, A. V. Marenich, J. Bloino, B. G. Janesko, R. Gomperts, B. Mennucci, H. P. Hratchian, J. V. Ortiz, A. F. Izmaylov, J. L. Sonnenberg, D. Williams-Young, F. Ding, F. Lipparini, F. Egidi, J. Goings, B. Peng, A. Petrone, T. Henderson, D. Ranasinghe, V. G. Zakrzewski, J. Gao, N. Rega, G. Zheng, W. Liang, M. Hada, M. Ehara, K. Toyota, R. Fukuda, J. Hasegawa, M. Ishida, T. Nakajima, Y. Honda, O. Kitao, H. Nakai, T. Vreven, K. Throssell, J. A. Montgomery Jr, J. E. Peralta, F. Ogliaro, M. J. Bearpark, J. J. Heyd, E. N. Brothers, K. N. Kudin, V. N. Staroverov, T. A. Keith, R. Kobayashi, J. Normand, K. Raghavachari, A. P. Rendell, J. C. Burant, S. S. Iyengar, J. Tomasi, M. Cossi, J. M. Millam, M. Klene, C. Adamo, R. Cammi, J. W. Ochterski, R. L. Martin, K. Morokuma, O. Farkas, J. B. Foresman, and D. J. Fox, Gaussian 16, Revision A.03, Gaussian, Inc., Wallingford CT, 2016.
7. A. D. Becke, *J. Chem. Phys.*, 1993, **98**, 5648.
8. S. Grimme, S. Ehrlich, L. Goerigk, *J. Comput. Chem.*, 2011, **32**, 1456.
9. J. A. Montgomery, Jr., M. J. Frisch, J. W. Ochterski, G. A. Petersson, *J. Chem. Phys.* 1999, **110**, 2822.
10. Rate constants were computed using:

    $k = \frac{RT}{P} \cdot \frac{k_B T}{h} e^{\frac{-\Delta G^{TS}}{RT}}$ where T = 310.15 K
